# Testing general relativity in cosmology

**DOI:** 10.1007/s41114-018-0017-4

**Published:** 2018-12-18

**Authors:** Mustapha Ishak

**Affiliations:** Department of Physics, The University of Texas at Dallas, Richardson, TX 75080 USA

**Keywords:** Tests of relativistic gravity, Theories of gravity, Modified gravity, Cosmological tests, Post-Friedmann limit, Gravitational waves

## Abstract

We review recent developments and results in testing general relativity (GR) at cosmological scales. The subject has witnessed rapid growth during the last two decades with the aim of addressing the question of cosmic acceleration and the dark energy associated with it. However, with the advent of precision cosmology, it has also become a well-motivated endeavor by itself to test gravitational physics at cosmic scales. We overview cosmological probes of gravity, formalisms and parameterizations for testing deviations from GR at cosmological scales, selected modified gravity (MG) theories, gravitational screening mechanisms, and computer codes developed for these tests. We then provide summaries of recent cosmological constraints on MG parameters and selected MG models. We supplement these cosmological constraints with a summary of implications from the recent binary neutron star merger event. Next, we summarize some results on MG parameter forecasts with and without astrophysical systematics that will dominate the uncertainties. The review aims at providing an overall picture of the subject and an entry point to students and researchers interested in joining the field. It can also serve as a quick reference to recent results and constraints on testing gravity at cosmological scales.

## Introduction

For over a century, Einstein’s general relativity (GR) has continued to be an impressive theory of gravity that fits observations from our solar system to the entire cosmological model of the universe. Guided by some key principles, Einstein came to the important realization of a very close relationship between the curvature of spacetime and gravity. Taking into account further requirements, such as coordinate invariance, conservation laws, and limits that must be consistent with Newtonian gravity, he proposed his gravitational field equations (Einstein 1915). Astonishingly, the same simple but powerful equations remain to date the most accurate description of gravitational physics at all scales.

Shortly after that, GR gave birth to the current standard model of cosmology predicting exact solutions with expanding or contracting universes. It allowed the combination of ideas from Friedmann and Lemaître about expanding universes (Friedmann 1922; Lemaître 1931) along with the geometry of homogeneous and isotropic spacetimes of Robertson (1935) and Walker (1937) in order to produce the so-called Friedmann–Lemaître–Robertson–Walker models (FLRW). These models describing the background cosmological evolution were completed by the addition of cosmological perturbation theory to populate them with cosmic structures. Over the years and decades to follow, the FLRW models plus cosmological perturbations benefited from a number of theoretical developments and observational techniques that allowed us to map the whole history of cosmic evolution from very early times to the current stages of the universe as we observe it today.

However, this scientific triumph in cosmology came with two conundrums: dark matter and cosmic acceleration (or dark energy). Indeed, in order for the FLRW model to fit current observations, we first need $\sim$ 25% of the mass-energy content in the universe to be in the form of a pressureless dark matter component that interacts only gravitationally with baryons and light (possibly weakly with baryons as well). The requirement for the presence of such a dark matter component does not come only from cosmology but also from rotation curves of galaxies, gravitational lensing observations, and the requirement of deep initial potential wells that would have allowed the formation of the largest structures that we observe today; see for example Trimble (1987), Bertone et al. (2005), D’Amico et al. (2009), Einasto (2014), Freese (2017) and references therein. The dark matter problem motivated the introduction of modified gravity (MG) theories that would explain such observations by a small modification to Kepler laws such as Modified Newtonian Dynamics (MOND) (Milgrom 1983b), its relativistic generalization known as TeVeS (tensor–vector–scalar) theory (Bekenstein 2004), or other vector–tensor theories. While Dark Matter motivated proposals of some MG models, the main focus of this review is rather on models that attempt to address the question of cosmic acceleration that we describe next.

The second problem in standard cosmology is indeed that of cosmic acceleration and the dark energy associated with it. Two decades ago, two independent groups using supernova measurements found that the universe’s expansion is speeding up rather than slowing down (Riess et al. 1998; Perlmutter et al. 1999). A plethora of complementary cosmological observations have continued since to confirm this result and require that an FLRW model fitting observations must have a genuine or effective dark energy component that would account for $\sim$ 70% of the total energy budget in the universe. In such a universe, the baryons constitute only $\sim$ 5% of this budget. This picture has become the concordance model in cosmology referred to as the Lambda-Cold-Dark-Matter ($\varLambda$CDM) model. This best fit model is spatially flat. $\varLambda$ is the cosmological constant, and its addition to the Einstein’s equations can produce an accelerated expansion of the universe.

The cosmological constant can be cast into the model as an effective cosmic fluid with an equation of state of minus one. This coincides exactly with the equation of state of the vacuum energy associated with zero-level quantum fluctuations. Interestingly, this connects the problem of cosmic acceleration to the problems of the cosmological constant/vacuum energy problems (Weinberg 1989; Carroll et al. 1992; Sahni and Starobinsky 2000; Carroll 2001; Peebles and Ratra 2003; Padmanabhan 2003; Copeland et al. 2006; Ishak 2007). Namely, why is the value measured from cosmology so small compared to the one predicted from quantum field calculations? This is known as the old cosmological constant problem. A second question (the new problem) is why the energy density associated with the cosmological constant/vacuum energy is of the same order of magnitude as the matter density at present cosmic time? (If it were any larger it would have prevented cosmic structure from forming.) Other types of dark energy have been proposed with an equation of state that is very close to minus one and would be not connected to the cosmological constant/vacuum energy. These are for example quintessence models based on a scalar field with kinetic energy and potential terms that can be cast as well into an effective dark energy model with a negative equation of state also close to minus one (Peebles and Ratra 1988; Ratra and Peebles 1988; Caldwell et al. 1998). It is worth noting that most of these dark energy models do not address the cosmological constant problem and may suffer from some form of fine-tuning as well.

Relevant to our review, the question of cosmic acceleration motivated a number of proposals for modified gravity models that could produce such an acceleration without the need for a cosmological constant. Such models are said to be self-accelerating. In most cases, these models do not address the cosmological constant problems and it is hoped that by some mechanism, for example degravitation or some given cancellation, vacuum energy does not contribute to gravitational and cosmological dynamics. However, in some cases, modified gravity models do provide some degravitation mechanism, although not successfully so far. We discuss these further in this review.

Finally, there are also modified gravity models at high energies that have been motivated by the search for quantum gravity and other unified theories of physics which may or may not have any consequences at cosmological scales.

While the rapid growth of the subject of deviations from GR and MG models has been motivated by cosmic acceleration/dark energy and to some extent by dark matter, the subject of MG models is an old one. Indeed, just a few years after GR was introduced, Weyl gravity was proposed by Weyl (1918), and so were the theories of Eddington (1924), Cartan (1922b) and Brans and Dicke (1961), and many others. Testing GR and gravity theories within the solar system and using other astrophysical objects have been the subject of intense work with a number of important results over the last five decades or so; see for a review (Will 2014). Impressively, GR fits all these local tests of gravity. In fact, it fits them so well that these tests are commonly referred to as GR local tests. This is very useful to the current cosmological developments, because it has established very stringent constraints at the level of the solar system that any gravity theory must pass. Nevertheless, to address these requirements, some MG models have some gravitational screening mechanisms that allow them to deviate from GR at cosmological scales but then become indistinguishable from it at small scales.

Further motivation for testing GR at cosmological scales is the increasing quantity and quality of available cosmological data. These are indeed good times for cosmology where a plethora of complementary observational data from ongoing and planned surveys will continue to flow for the many decades to come. These include the cosmic microwave background radiation, weak gravitational lensing, galaxy surveys, distances to supernovae, baryon acoustic oscillations, and gravitational waves. A good piece of news is that one can not only combine these data sets to increase their constraining power, but one can also apply consistency tests between such complementary data sets. This would allow one to identify any problems with systematic effects in the data or any problems with the underlying model. Furthermore, nature has also given us a break in cosmology as we have two types of data sets. Indeed, some data sets are sensitive to the geometry and expansion of the universe and some other sets are sensitive to the growth of large-scale structure (i.e., the rate at which structures cluster in the universe). These two sets of observations must be consistent with one another. For testing deviations from GR and constraining MG models, it was realized that MG models can mimic an expansion history of the universe that is identical to that of the concordance $\varLambda$CDM model while they can still have a structure formation history that is different and distinguishable from that of $\varLambda$CDM. It has become common practice that the background expansion is modeled with an effective dark energy with an equation of state close to the minus one value of $\varLambda$CDM. Meanwhile, any departure from GR is constrained by using the growth data from large-scale structure observables.

There are two general approaches that have been developed to test departures from GR at cosmological scales. The first one is where the deviation is parameterized in a phenomenological way with no necessary exact knowledge of the specific alternative theory. The growth equations are modified by the addition of MG parameters that represent the departure from GR. These MG parameters are expected to take values of unity for GR but depart from it for MG models. It is worth noting that such an effective description may not necessarily remain valid at all scales constrained by observations and so must be used with some caution when compared to various observations. The second approach is to choose a specific class of MG models [like the popular *f*(*R*) or DGP models (see Sects. 7.4.1 and 7.5.2)] and derive cosmological perturbations and observables for these models. These are then implemented in cosmological analysis software and used to compare to the data. We cover both approaches in this review. A related question is what one could call a modified gravity model versus a dark energy model. There are some guiding helpful prescriptions that we discuss in the review but the spectrum of models has a grey zone where such a distinction is not unambiguous. We characterize various types of deviations from general relativity and organize MG models accordingly with some illustrative examples.

In this review, we aim at providing an overall current picture of the field of testing gravity at cosmological scales including a selection of recent important results on the subject. The review is meant to provide an entry point for students and researchers interested in the field where they can find summaries and references to further readings. This review can also serve for experienced researchers or other readers to find quickly recent developments or results in the field. As required for the Living Review guidelines, this review is written with the depth and style of a plenary review talk on the subject. It is not meant to replace thorough comprehensive reviews on various parts of this topic and we refer the reader constantly to such specialized reviews as we discuss each sub-topic.

## General relativity (GR)

### Basic principles

Einstein considered some key guiding principles and well-known limits that a successful theory of gravity must obey. At the forefront is the principle of covariance—that is the laws of physics must be independent of any coordinate system. So the right language must be that of tensors or another coordinate independent formulation. Such a successful theory should locally be consistent with special relativity and must inherit its principles including the equivalence of local inertial frames of reference, the universal constancy of the speed of light in vacuum, and the Lorentz-invariance of the theory.

An important part of Einstein’s reflections when he proposed special relativity and then continued to work toward general relativity was about the principles of equivalence. He found guidance in Mach’s ideas about relativity and the nature of inertia (Mach et al. 1905, 1988), although, he had to abandon some of them later on.

From the principle of equivalence between gravity and inertia that we provide below, Einstein developed the important insight that gravity seems to have a privileged status compared to other interactions. That is gravity is equivalent to inertia. The principle of universality of free-fall and gravitational interaction as expressed below in the equivalence principles combined with some insight that gravity is omnipresent in spacetime, led Einstein to formulate gravity as the curvature of spacetime. See various discussions and perspectives in reviews and books, e.g., Will (2014, 2018), d’Inverno (1992), Rindler (2006), Weinberg (1972), Misner et al. (1973) and Carroll (2003).

- **Weak equivalence principle (WEP)** WEP is stated in a variety of formulations. One of them is usually stated as the equivalence between the inertial mass and the gravitational mass which has been been tested to a few parts in $10^{13}$ (Adelberger 2001; Wagner et al. 2012) and a few parts in 10$^{14}$ (Touboul et al. 2017), Will (2014) for WEP test timeline. Einstein then advocated that inertia and gravity must be the same and that an observer inside a “cabin” (with no windows) at rest in the presence of a gravitational acceleration will not be able to distinguish that situation from one where the “cabin” is on a rocket moving up with the exact opposite acceleration. The WEP is expressed as the universality of the gravitational interaction and free-fall for all particles. For our review, we focus on the notions of universality of free fall and the matter coupling in the context of GR $+$ dark energy versus modified gravity (MG) models following for example Joyce et al. (2016). WEP is satisfied if there exists some spacetime metric (in the Jordan frame) to which all species of matter are universally coupled. Test particles fall then along geodesics of this metric.
- **Einstein equivalence principle (EEP)** The EEP requires the validity of the WEP, and that in all local freely falling frames, the laws of physics reduce to those of special relativity (assuming tidal gravitational forces are absent). It is also customary to add here that the EEP contains the statement that the outcome of any local non-gravitational experiment is independent of where and when it is performed (Will 2014).
- **Strong equivalence principle (SEP)** The SEP extends the universality of free fall of the WEP to massive gravitating objects so it is completely independent of the composition of the objects as well as their gravitational binding energy. Compact objects like Black Holes will also fall along geodesics like test particles (Will 1994, 2014). The SEP extends also the EEP to include all of the laws of physics, gravitational or otherwise.

One more remark is worth mentioning about the relationship between the equivalence principles and the spacetime metric. Let us recall that metric theories of gravity satisfy the following properties, see for example Will (2014): (i) a symmetric metric exists, (ii) test particles follow geodesics of such a metric, and (iii) in local reference frames, the non-gravitational laws of physics are those of special relativity. From this definition, it follows that metric theories obey the EEP. It also encourages one to imply that theories that obey the EEP are metric theories, e.g., Will (2014).

We conclude this subsection by commenting on a few other notions that guided Einstein in formulating his equations of the gravitational field. The geometrical nature of GR and the principles it is built upon are certainly far from the Newtonian gravity based on forces and potentials, not to mention the notions of absolute space and other shortcomings that had to be abolished. However, it is interesting to remark that the notion of spacetime and its metric to explain gravity can be compared to the notion of the gravitational potential field in space created by massive objects. However, there is a major difference, in GR there is no gravitational potential or gravity that is added on the top of spacetime, but gravity is curvature of spacetime itself. This was a major insight that Einstein got from his EEP principle. In fact, he knew well that GR must have Newtonian gravity as a limit in the weak regime and that provided to him many hints on how to formulate the field equations that we provide in the next section.

### Einstein field equations (EFEs) and their exact solutions

In addition to the principles above, Einstein used the fact that, in the weak field limit, the gravitational field equations must locally reduce to those of Newtonian gravity where the metric tensor components would be related to the gravitational potential and the field equations must reduce to Poisson equations. From the latter, he imposed that the curvature side of the equations must contain only up to second order derivatives of the metric and must also be of the same tensor rank as the energy-momentum tensor. This naturally led Einstein to consider the Ricci tensor, derived from contracting twice the Riemann curvature tensor, but there was a little bit more into it. Indeed, he knew that the equations must satisfy conservation laws and thus must be divergence-free. While the vanishing of the divergence of the matter-energy source side of the equations is assured by energy conservation laws and continuity equations, on the curvature side, the Ricci tensor is not divergence-free so more work was required. For that, Einstein built precisely the tensor that holds his name which, by the Bianchi identity, is divergence-free hence complies with conservation laws, as it should. Some technical or historical entire books or articles have been devoted to what led Einstein to derive his equations and we refer the reader to the extended study by Janssen et al. (2007) and references therein.

With no further discussion, the Einstein’s field equations (EFEs) read

1 $$\begin{aligned} G_{\mu \nu }+\varLambda g_{\mu \nu }=8\pi G T_{\mu \nu }, \end{aligned}$$

where $G_{\mu \nu }\equiv R_{\mu \nu }-\frac{1}{2}g_{\mu \nu }R$ is the Einstein tensor representing the curvature of spacetime, $R_{\mu \nu }$ is the Ricci tensor, *R* the Ricci scalar, $g_{\mu \nu }$ is the metric tensor, and $\varLambda$ is the cosmological constant. For brevity we use units such that $c=1$ throughout. On the RHS, the source (content) of spacetime is represented by the energy momentum tensor

2 $$\begin{aligned} T_{\mu \nu }=(\rho +p)u_{\mu }u_{\nu }+pg_{\mu \nu }+q_{\mu }u_{\nu }+u_{\mu }q_{\nu }+\pi _{\mu \nu }, \end{aligned}$$

where $u^{\mu }$ is the tangent velocity 4-vector (e.g., the tangent field to the cosmic fluid particle world-lines) normalized by $u_{\mu }u^{\mu }=-1$, $\rho$ is the relativistic mass-energy density, *p* is the isotropic pressure, $q^{\mu }$ the energy flux, and $\pi _{\mu \nu }$ is the trace-free anisotropic pressure or stress, all relative to $u^{\mu }$. The quantities $\rho$, *p*, $q_{\mu }$, and $\pi _{\mu \nu }$ are functions of time and space. We use the signature $(-,+,+,+)$ and a 3$+$1 decomposition of spacetime unless stated otherwise.

In standard cosmology, it is assumed that the cosmic fluid is well-described by a perfect fluid (i.e., $q_{\mu }=0$ and $\pi _{\mu \nu }=0$) at the cosmic background level which accounts for baryons, dark matter, radiation and a cosmological constant or another dark energy component. The energy-momentum tensor then reduces to

3 $$\begin{aligned} T_{\mu \nu }=({\bar{\rho }}+\bar{p})u_{\mu }u_{\nu }+\bar{p}g_{\mu \nu }, \end{aligned}$$

where the last three terms of (2) are set to zero, and the over bar means average over space of quantities and are now functions of time only. However, at the perturbation level, the velocity field contributes to the heat flux and neutrinos, for example, generate anisotropic shear at early times in the universe.

It is not widely known that the EFEs have over 1300 exact solutions that have been derived over the last century, see for example the classical compilation book by Stephani et al. (2003) and also Online Interactive Geometric Databases equipped with a live tensor component calculator (Ishak and Lake 2002). These solutions are based on symmetries of the spacetime and defined forms of the energy momentum source.

While the large number of exact solutions exhibit the richness and mathematical beauty of the field, a number of solutions still lack any physical interpretation (Stephani et al. 2003; Delgaty and Lake 1998; Ishak et al. 2001). Some of these solutions have found direct applications to real astrophysical systems. These include the popular Schwarzschild static spherically symmetric vacuum solution around a concentric mass (Schwarzschild 1916). The solution is often used to model space around Earth, Sun, or other slowly rotating objects where it leads to more accurate predictions than Newtonian gravity, see e.g., Will (2014). The solution is also used to represent the exterior spacetime around a static spherically symmetric black hole. A second well-know exact solution is that of Kerr (1963) representing the vacuum space around an axially symmetric rotating compact object or black hole. Next, several other static spherically symmetric non-vacuum solutions such as the Tolman family of solutions (Tolman 1939) and the Buchdahl solutions (Buchdahl 1967) have been used to model the interior of compact astrophysical objects such as Neutron stars (Lattimer and Prakash 2007). Finally, some solutions have found applications in cosmology. These include, for example, the isotropic and homogeneous Friedmann–Lemaître–Robertson–Walker (FLRW) solutions (Friedmann 1922; Lemaître 1931; Robertson 1935; Walker 1937), the inhomogeneous Lemaître–Tolman–Bondi solutions (Lemaître 1933; Tolman 1934; Bondi 1947), the inhomogeneous Szekeres models (Szekeres 1975), the anisotropic Bianchi models (Ellis and MacCallum 1969), and others (Ellis and van Elst 1999).

Einstein’s Equations of general relativity connected naturally the isotropic and homogeneous geometry of space given by the Robertson–Walker metric to the cosmic fluid substratum described by a perfect fluid, giving birth to the standard model of cosmology that we describe in the next section.

It is important to note, and in particular in the context of this review, that while Einstein derived his equations from the principles and approach discuss above, the field equations also derive immediately from a variational principle where the action for the curvature sector is simply the Ricci scalar. This was derived simultaneously by Einstein and Hilbert and the curvature part of the action bears their names. The GR action with a cosmological constant term reads

4 $$\begin{aligned} S_{\textit{GR}}=\int d^{4}x \sqrt{-g} \left[ \frac{R- 2 \varLambda }{16 \pi G}+{\mathcal {L}}_{M}\right] , \end{aligned}$$

where *g* is the determinant of the metric tensor and ${\mathcal {L}}_{M}$ is the Lagrangian for the matter fields. Variations of Eq. (4) with respect to the metric, $g_{\mu \nu }$, gives the field equations (1) above. Modified gravity models are often introduced at the level of the action.

Finally, with regards to this review, it is worth clarifying that modifications to GR mean also that the above exact solutions are not anymore valid and need to be replaced by their homologous solutions in the modified theory. For cosmology, an FLRW metric is often used but then leads to modified dynamical equations often referred to as modified Friedmann’s equations.

## The standard model of cosmology

### The homogeneous cosmological background

#### FLRW metric and Friedmann’s equations

From the nearly isotropic large scale observations around us and the assumption that it should not look any different from another point in the universe (i.e., the cosmological principle), one can infer that the universe can be described by a spacetime that is globally isotropic and thus homogeneous. The geometry is then described by the metric of Friedmann–Lemaître–Robertson–Walker (FLRW) with line element

5 $$\begin{aligned} ds^2=-dt^2+a^2(t)\left( \frac{dr^2}{1-kr^2}+r^2(d\theta ^2+\sin ^2\theta d\phi ^2)\right) , \end{aligned}$$

where *a*(*t*) is the expansion scale factor representing the time-dependent evolution of the spatial part of the metric (surfaces of constant *t*), and $k\in \{-1,0,+1\}$ determines the geometry of these spatial sections: negatively curved, flat, or positively curved, respectively.

The EFEs (1) solved for the FLRW metric (5) and a perfect fluid source energy momentum tensor (3) give the dynamical Friedmann equations. The first equation derives from time-time components of the EFEs as

6 $$\begin{aligned} \frac{\dot{a}^2}{a^2}=H(t)^2=\frac{8\pi G}{3}{\bar{\rho }} +\frac{\varLambda }{3}-\frac{k}{a^2}, \end{aligned}$$

where an *overdot* denotes the derivative with respect to the cosmic time *t*, and we isolated on the LHS the Hubble parameter defined as,

7 $$\begin{aligned} H(t)\equiv \frac{\dot{a}(t)}{a(t)}. \end{aligned}$$

This allows us to define a first cosmological parameter, the Hubble constant as $H_0=H(t_0)$ where $t_0$ is the present time. It is common to use instead the normalized parameter $h \equiv H_0/$(100  km $\mathrm s^{-1}$ Mpc$^{-1}$). As usual, in the spatially flat case, the scale factor can be normalized such that its present value $a_0=a(t_0)\equiv 1$. We recall that in spatially curved space, one cannot normalize simultaneously the spatial curvature and the scale factor. The cosmological redshift is related to the scale factor by $1 + z = a_0/a$.

The second Friedmann equation derives from the combination of the space-space component and the time-time component of the EFEs, and can be written as an acceleration/deceleration equation as follows

8 $$\begin{aligned} \frac{\ddot{a}}{a}\, = -\frac{4\pi G}{3}\left( {\bar{\rho }}\, +\, 3\bar{p} \right) \, +\, \frac{\varLambda }{3}. \end{aligned}$$

It is sometimes more convenient to replace the radial coordinate, *r*, by the comoving coordinate $\chi$ using $d\chi \equiv {dr}/{\sqrt{1-kr^2}}$ so that the line element reads

9 $$\begin{aligned} ds^2=-dt^2+a^2(t)\left( d\chi ^2+f_{K}^2(\chi ) \left( d\theta ^2+\sin ^2\theta d\phi ^2\right) \right) , \end{aligned}$$

where

10 $$\begin{aligned} f_K(\chi )=\left\{ \begin{array}{ll} \sin (\chi ) &{}\quad k=+1 \\ \chi &{}\quad k=0 \\ \sinh (\chi ) &{}\quad k=-1 \end{array}.\right. \end{aligned}$$

Finally, it is also sometimes convenient to change the coordinate (cosmic) time to the conformal time defined as $d\tau \equiv {dt}/{a(t)}$ so the line element now reads

11 $$\begin{aligned} ds^2=a^2 (\tau ) \left[ -d\tau ^2 + d\chi ^2+f_{K}^2(\chi ) \left( d\theta ^2+\sin ^2\theta d\phi ^2\right) \right] . \end{aligned}$$

The Friedmann equations and the FLRW metric provide a description of the homogeneous universe and its dynamics serving as a basis to study the propagation of light, the expansion history, distance measures, and the energy budget of the universe.

Again, with regards to modifications to GR, the Friedmann’s equations above, i.e., (6) and (8), are modified and so are all the observables and distance measurements described below that build on these equations. For example, in relation to cosmic acceleration, the cosmological constant term can be replaced by extra terms coming from the modification and that could play a similar role to it. However, as we already mentioned in the introduction, some of these models are able to fit well the expansion and background observations so any further distinction will have to come from the growth of structure constraints and observables.

#### Cosmic mass-energy budget, dark energy and cosmic acceleration

In general relativity, conservation laws are given by the vanishing of the covariant derivative of the energy momentum tensor, i.e., $T^{\mu \nu }_{\;\;\;\;\; ;\nu }=0$. This provides the continuity equation

12 $$\begin{aligned} \dot{{\bar{\rho }}}+3 \frac{\dot{a}}{a}({\bar{\rho }}+\bar{p})=\dot{{\bar{\rho }}}+3 \frac{\dot{a}}{a}{\bar{\rho }}(1+w)=0, \end{aligned}$$

where in the last step we used the equation of state variable, *w*, defined as

13 $$\begin{aligned} \bar{p}=w{\bar{\rho }}. \end{aligned}$$

It follows from the continuity Eq. (12), that for a matter (baryon and dark matter) dominated epoch (i.e., $w=0$) ${\bar{\rho }}_m \propto a^{-3}$, for a radiation dominated epoch (i.e., $w=1/3$) ${\bar{\rho }}_r \propto a^{-4}$, and for a cosmological constant (i.e., $w=-1$) $\rho _{\varLambda }$ is a constant, while for a dynamical dark energy with $w_{de}$,

14 $$\begin{aligned} {\bar{\rho }}_{de} = {\bar{\rho }}_{de}^{0} a^{-3(1+w_{de})}. \end{aligned}$$

In models of dynamical dark energy, $w_{de}$ is another cosmological parameter that is allowed to be different from $-1$ in cosmological analyses. It can also be allowed to vary in redshift (or scale factor) in which case it can, for example, take the form $w(a)=w_0+w_a(1-a)$ known as CPL parameterization (Chevallier and Polarski 2001; Linder 2003). Other parameterizations for *w* have been introduce in order to fit other dark energy or modified gravity models. Alternatively, the equation of state can also be binned in the redshift.

It is trivial to observe from the second Friedmann equation (8) that a cosmic effective dark energy fluid with an equation of state $w_{de}=p_{de}/\rho _{de}<-1/3$ gives an accelerated expansion. This is the case for a cosmological constant. The field equations of GR have no difficulty in mathematically producing an accelerated expansion, but the real challenge is to figure out what is the physical nature of such an effective dark energy fluid.

So far, most analyses are consistent with the value of $w=-1$ of a cosmological constant with shrinking error bars around it; see for example DES Year-1 cosmological parameter paper (Abbott et al. 2018b) where combining most available data sets gave $w_{de}= -1.00{\,}^{+0.04}_{-0.05}$. Although the latest data from Planck and Planck combined with other data sets was found to slightly favor $w_{de}$ values slightly smaller than $-1$ (Ade et al. 2016b). However, current data do not yet significantly constrain the $w_0$ and $w_a$ parameters for a time-varying equation of state of DE.

In order to describe the energy budget in the universe as measured from observations, we first need to describe the critical density of the universe evaluated today, noted as $\rho _{crit}^0$. This will serve as a reference density and is determined from the first Friedmann equation (6) in a spatially flat universe with no cosmological constant. That is:

15 $$\begin{aligned} \rho _{crit}^0= & {} \frac{3H^2_0}{8\pi G} \nonumber \\= & {} 1.9\times 10^{-29}h^2 {\mathrm{grams}} \,\,{\mathrm{cm}}^{-3} \nonumber \\= & {} 2.8\times 10^{11}h^2 {\mathrm{M}}_{\odot } {\mathrm{Mpc}}^{-3}. \end{aligned}$$

The last line is given in solar masses, $\mathrm{M}_{\odot }$, per megaparsec cubed. We can now use this reference density to express the density parameters today for different species as the ratio

16 $$\begin{aligned} \varOmega _i^0=\frac{{\bar{\rho }}_i^0}{\rho _{crit}^0}. \end{aligned}$$

This defines 3 other cosmological parameters with their values today as for example estimated from Planck and other data sets (Ade et al. 2016a): $\varOmega _b^0\approx 0.05$ for baryonic matter, $\varOmega _dm^0 \approx 0.26$ for cold dark matter, $\varOmega _{\varLambda }^0 \approx 0.69$ for a cosmological constant, and a tiny curvature “density” parameter $|\varOmega _k^0\equiv -k/H_0^2| < 0.01$. These numbers characterize the standard spatially flat Lambda-Cold-Dark-Matter ($\varLambda$CDM) concordance model.

The Friedmann equation (6) can be re-written in terms of these density parameters and the scale factor as

17 $$\begin{aligned} H^2(a)=H^2_0 \left[ \varOmega _m^0 a^{-3}+ \varOmega _r^0 a^{-4}+ \varOmega _k^0 a^{-2}+\varOmega _{de}^0 a^{-3(1+w)} \right] , \end{aligned}$$

where we use $\varOmega _m^0 \equiv \varOmega _b^0+\varOmega _c^0$ and recall that $\varOmega _r^0\approx 10^{-4}$ and is so negligible at the present time. So when evaluated today for a spatially flat universe with a cosmological constant, $\varLambda$, Eq. (17) reduces to simply $\varOmega _m^0+\varOmega _{\varLambda }^0=1$.

#### Cosmological distances

Another useful background information to cover is that of distances in cosmology. We start with the physical distance or proper distance (e.g., Weinberg 1972), defined for example by integrating the line element (9) at a given instant along a radial direction so that $dt=d\theta =d\phi =0$

18 $$\begin{aligned} d_{phys}(t)={a(t)} \int ^{\chi }_0 d\chi '={a(t)}\chi . \end{aligned}$$

This is the distance that would be instantaneously measured if we used a gigantic ruler from us to a remote object. In Weinberg (1972), this is equivalently defined from (9) as

19 $$\begin{aligned} d_{prop}(t)=\int ^{r}_0 \sqrt{g_{rr}}dr'={a(t)}\int ^{r}_0 \frac{dr'}{\sqrt{1-kr'^2}}={a(t)}\chi . \end{aligned}$$

This distance is time dependent so a radial comoving distance is often used as

20 $$\begin{aligned} \chi =\frac{d_{phys}}{{a(t)}}. \end{aligned}$$

In the spatially flat case, with the normalization of $a\equiv 1$ today, the comoving distance is normalized to be equal to the proper distance today. Also, the normalized comoving distance to a galaxy with redshift *z* (or $a=1/(1+z)$) is thus given from Eq. (9) as

21 $$\begin{aligned} \chi =\int ^{t_{today}}_{t}\frac{dt'}{a(t')}= \int ^{1}_{a}\frac{da'}{a'^2 H(a')}. \end{aligned}$$

Now, astronomers define other distances that can be measured by different methods. First, the angular diameter distance is defined for an object that has a typical diameter size, $\mathcal {D}$, and an angular observed size, $\delta \theta$ as (Ellis 1973; Ellis and van Elst 1999)

22 $$\begin{aligned} d_A\equiv & {} \frac{\mathcal {D}}{\delta \theta }=\frac{\sqrt{g_{\theta \theta }}d\theta }{\delta \theta }\nonumber \\= & {} {a(t)}f_K(\chi ), \end{aligned}$$

where we have used the metric (9) and $f_K(\chi )$ is given by (10). Furthermore, the comoving angular diameter distance is defined as

23 $$\begin{aligned} d_{AC}\equiv \frac{d_A}{{a(t)}}=f_K(\chi ), \end{aligned}$$

so in a spatially flat cosmology, $\chi$ is also referred to as the comoving angular diameter distance.

Finally, for an object with luminosity, *L*, and flux, *F*, measured here at the observer [for example on a Charged-Coupled Device (CCD)], the luminosity distance, $d_L$, is defined from the relation

24 $$\begin{aligned} F \equiv \frac{L}{4\pi d_L^2}. \end{aligned}$$

From photon conservation, the flux measured at observer can be written in terms of the metric functions of (9) and the source redshift as Ellis and van Elst (1999)

25 $$\begin{aligned} F=\frac{L}{4\pi (1+z)^2 r_G^2}, \end{aligned}$$

where $r_G\equiv a(t_0) f_K(\chi )$ is called the galaxy area distance. Furthermore, two effects need to be considered. The first is that photons are redshifted by a factor $(1+z)$, and the second effect is that there is a time dilation due to cosmic expansion providing a second factor $(1+z)$.

Now, comparing Eqs. (24) and (25), and using $r_G$, the luminosity distance is given by

26 $$\begin{aligned} d_L(z)=f_K(\chi )(1+z). \end{aligned}$$

$d_L$ is thus related to the angular diameter distance, $d_A$, by

27 $$\begin{aligned} d_L=d_A(1+z)^2. \end{aligned}$$

This is Etherington’s reciprocity theorem (or distance-duality relation), which is true when the number of photons traveling on null geodesics is conserved.

### The inhomogeneous lumpy universe and the growth of large-scale structure

#### Large-scale structure and cosmological perturbations

The universe we observe at large scales is rather full of clusters and superclusters of galaxies. Such a picture is mathematically realized by applying linear perturbations to Einstein’s equations in an FLRW background. Sufficiently large scales are considered so linear perturbations are a valid description.

This is done by adding to the metric tensor a small perturbation tensor. Then computing the Einstein tensor to the first order. At the same time, the energy momentum tensor is also linearly perturbed. The Einstein equations then give the usual background Friedmann equations (184) plus additional equations governing the evolution of the perturbations (see, e.g., Carroll 2003; Peter 2013 for a pedagogical introductions and also some of the seminal references Bardeen 1980; Kodama and Sasaki 1984). An insightful approach to these linear perturbations is to decompose the components of the symmetric metric tensor perturbations according to how they transform under spatial rotations. The 00-component of the metric perturbation tensor is a scalar, the three 0i-components (or equally the three i0-components) constitute a vector, and the remaining nine ij components form a symmetric spatial tensor of rank two. This is known as the SVT decomposition of linear perturbations. The three parts transform only into components of the same type under spatial rotations. In GR, the scalar modes are, for example, associated with matter density fluctuations and used for large scale structure studies, tensor modes are associated with gravitational radiation used, for example, for primordial gravitational waves, while vector modes decay in and are usually ignored. Last, in addition to this decomposition, one needs to specify a gauge choice where the components of the perturbations can be different in the corresponding coordinate system, see e.g., Carroll (2003) and Peter (2013) for pedagogical discussions. Modification to gravity can be implemented at the level of scalar mode perturbations as we discuss further below or at the level of tensor modes as in, e.g., Saltas et al. (2014), Pettorino and Amendola (2015), Dubovsky et al. (2010), Raveri et al. (2015), Amendola et al. (2014) and Lin and Ishak (2016).

In this review, we will focus scalar perturbations. The perturbed spatially flat FLRW metric reads in, for example, the conformal Newtonian gauge as

28 $$\begin{aligned} ds^2=a(\tau )^2[-(1+2\varPsi )d\tau ^2+(1-2{\varPhi })dx^idx_i], \end{aligned}$$

where $x_i$’s are the comoving coordinates, and $\tau$ the conformal time defined further above. $\varPhi$ and $\varPsi$ are the gravitational scalar potentials describing the scalar mode of the metric perturbations.

We consider subhorizon scales with $k \gg aH$. In many analyses and papers on testing gravity at cosmological scales, the perturbed equations are often specialized to the quasi-static limit or approximation. This means that the time evolution of the gravitational potentials is assumed to be small compared to the Hubble time so one can assume the derivatives of the potentials to be zero for sub-Hubble-horizon scales. For scalar–tensor theories, this approximation also means that one neglects the time derivatives of the fluctuations in the scalar field at scales below the scalar perturbation sound horizon. More on this approximation or its limits can be found in, e.g., Noller et al. (2014), Sawicki and Bellini (2015) and Pogosian and Silvestri (2016).

The first-order perturbed Einstein equations in Fourier space give two equations that describe the evolution of the two scalar gravitational potentials, e.g., Ma and Bertschinger (1995). The combination of the time-time and time-space perturbed equations provides a Poisson equation for the potential $\varPhi$. The second equation includes the two potentials and comes from the traceless space-space components. The two equations read (in the quasi-static approximation for the potentials)

29 $$\begin{aligned} k^2{\varPhi }= & {} -4\pi G a^2\sum _i {\bar{\rho }}_i \delta _i \end{aligned}$$

30 $$\begin{aligned} k^2(\varPsi -\varPhi )= & {} -12 \pi G a^2\sum _i {\bar{\rho }}_i(1+w_i)\sigma _i, \end{aligned}$$

where ${\bar{\rho }}_i$ and $\sigma _i$ are the density and the shear stress, respectively, for matter species denoted by the index *i*. $\delta _i$ is the gauge-invariant, rest-frame overdensity for matter species, *i*. Its evolution describes the growth of inhomogeneities. It is defined by

31 $$\begin{aligned} \delta _i = \delta _i +3\mathcal {H}\frac{q_i}{k}, \end{aligned}$$

where $\mathcal {H} ={a}'/a$ is the Hubble factor in conformal time (where $'$ is for differentiation with respect to conformal time), and for species *i*,

32 $$\begin{aligned} \delta _i=\frac{\rho _i-{\bar{\rho }}_i}{{\bar{\rho }}_i} \end{aligned}$$

is the fractional overdensity; ${{\bar{\rho }}_i}$ is the background average density; $q_i$ is the heat flux related to the divergence of the peculiar velocity, $\theta _i$, by

33 $$\begin{aligned} \theta _i=\frac{k\ q_i}{1+w_i}. \end{aligned}$$

From conservation of the energy-momentum in the perturbed matter fluid, these quantities for uncoupled fluid species or the mass-averaged quantities for all the fluids evolve as, e.g., Ma and Bertschinger (1995):

34 $$\begin{aligned} {\delta }'= & {} -k q +3(1+w){\varPhi }'+3\mathcal {H}\left( w-\frac{\delta P}{\delta \rho }\right) \delta \end{aligned}$$

35 $$\begin{aligned} \frac{{q}'}{k}= & {} -\mathcal {H}(1-3w)\frac{q}{k}+\frac{\delta P}{\delta \rho }\delta +(1+w)\left( \varPsi -\sigma \right) . \end{aligned}$$

Combining these two equations, one obtains the evolution equation of $\delta$ as

36 $$\begin{aligned} {\delta }' = 3(1+w)\left( {\varPhi }'+\mathcal {H}\varPsi \right) +3\mathcal {H}w\delta -\left[ k^2+3\left( \mathcal {H}^2-{\mathcal {H}}'\right) \right] \frac{q}{k}-3\mathcal {H}(1+w)\sigma . \end{aligned}$$

Equations (29), (30), (34), and (35) above are coupled to one another; their combinations, along with the evolution equations for the scale factor $a(\tau )$, can provide a full description of the growth history of structures in the universe.

#### Growth factor and growth rate of large-scale structure

Now, specializing the above equations to the case of matter (baryons plus cold dark matter) at late time, we can set $w=\delta P/\delta \rho =\sigma =0$. Also using the quasi-static approximation (i.e., ${\varPhi }'=0$), Eq. (34) reduces to

37 $$\begin{aligned} \delta '_m=-kq=-\theta . \end{aligned}$$

Next, taking its derivative and using Eq. (35) as well as the two Poisson equations (29) and (30), we write

38 $$\begin{aligned} {\delta }_m''+\mathcal {H}{\delta }_m'-4 \pi G a^2 {\bar{\rho }} \delta _m=0. \end{aligned}$$

In cosmic time, this reads,

39 $$\begin{aligned} \ddot{\delta }_m+2{H}\dot{\delta }_m-4 \pi G {\bar{\rho }} \delta _m=0. \end{aligned}$$

This time evolution equation for $\delta$ has a solution with decaying and growing modes. We are interested in the growing modes (denoted with a $+$ subscript) that gave the structures that we observe today in the universe. One thus defines $D_{+}(t)$ as the linear growth factor of perturbations relating the overdensity $\delta (t)$ at some given time *t* to its value at some initial time $t_i$. That is

40 $$\begin{aligned} \delta (t)=\frac{D_{+}(t)}{D_{+}(t_i)} \delta (t_i), \end{aligned}$$

where ${D_{+}(t_i)}$ and $\delta (t_i)$ are constants set by initial conditions. The growth factor is often properly normalized as $G(z)\equiv D(a)/a$.

A paramount quantity in probing the growth of large scale structure is the growth rate, defined as the derivative of the logarithm of the growth factor with respect to the logarithm of the scale factor, i.e.,

41 $$\begin{aligned} f(a)\equiv \frac{d \ln D}{d \ln a}. \end{aligned}$$

As we will discuss further, some observations, such as Redshift Space Distortions (RSD), are directly sensitive to this function (or its product with the amplitude of matter fluctuation, $\sigma _8(a)$). The growth differential equation (39) above can be rewritten in terms of the growth rate (41) where the effect of modification to gravity can be encapsulated in an effective gravitational constant $G_{\mathrm{eff}}$ or a modified gravity parameter $\mu (k,a)$ (see Sect. 5.2 further) and thus re-written as:

42 $$\begin{aligned} \frac{df}{d \ln a}+f^2+\left( \frac{\dot{H}}{H^2}+2\right) f=\frac{3}{2}\frac{G_{\mathrm{eff}}^{\psi }}{G}\varOmega _m \equiv \frac{3}{2}\,\mu \, \varOmega _m \end{aligned}$$

(for GR, $G_{\mathrm{eff}}=G$ and $\mu =1$ , recovering the standard expression).

**Fig. 1:**
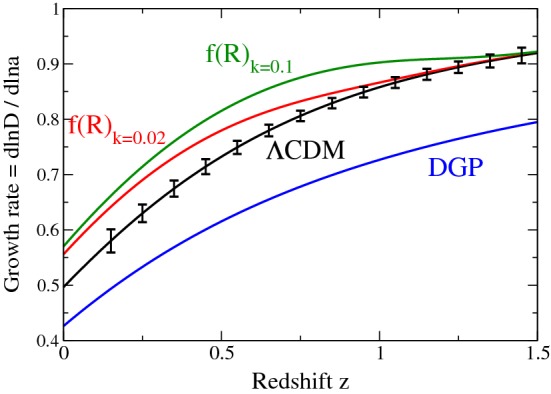
Growth rate of matter density fluctuations, *f*(*z*). Theory prediction curves are shown for: the $\varLambda$CDM model; the Dvali–Gabadadze–Porrati braneworld model (the self-accelerating branch, see Sect. 7.5.2) (Dvali et al. 2000); and the *f*(*R*) (see Sect. 7.4.1) modified gravity model (Hu and Sawicki 2007a) [model with $c=3$ from Linder (2009)]. Note that the growth in *f*(*R*) models is scale-dependent so the authors show predictions at two wavenumbers, $k=0.02\,\hbox {h}\,{{\mathrm{Mpc}}^{-1}}$ and $k=0.1\,\hbox {h}\,{{\mathrm{Mpc}}^{-1}}$. Also shown are the error bars projected from a future galaxy spectroscopic redshift survey designed with DESI survey specifications (Aghamousa et al. 2016) Image reproduced with permission from Huterer et al. (2015), copyright by Elsevier

For illustration, we reproduce Fig. 2 from Huterer et al. (2015) (Fig. 1 here) where it is shown how the function *f*(*z*) can be a discriminator for various gravity theories.

#### Correlation function and matter power spectrum

The galaxy correlation function is a measure of the degree of clustering in a spatial or angular distribution of galaxies. If $\delta _g(\mathbf {r})$ represents the galaxy overdensity with respect to an expected mean density then the correlation function is given by the 2-point function

43 $$\begin{aligned} \xi (\mathbf {r_1},\mathbf {r_2}) \equiv \langle \delta _g(\mathbf {r_1})\delta _g(\mathbf {r_2}) \rangle , \end{aligned}$$

where $\langle \dots \rangle \,$ denotes the ensemble average. The galaxy correlation function can be further understood as follows (Baugh 2000): Let’s consider two volume elements, $dV_1$ and $dV_2$ separated in space by $r_{12}$. The 2-point correlation can be defined as the excess probability, in comparison with a random distribution, of finding a galaxy in $dV_1$ and another in $dV_2$. That is:

44 $$\begin{aligned} d P = \bar{n}^2\left[ 1+\xi (r_{12})\right] dV_1 dV_2, \end{aligned}$$

where $\bar{n}$ is the mean galaxy number density. Due to the assumption of isotropy and homogeneity, the vector notation is dropped and only the distance $r_{12}$ has been kept.

A closely related quantity is the galaxy power spectrum which is defined as the Fourier transform of the correlation function as

45 $$\begin{aligned} P_g(k)= & {} \int \xi (r)e^{i\mathbf{k\cdot r}}d^3r,\end{aligned}$$

46 $$\begin{aligned} \xi (r)= & {} \int P(k)e^{-i\mathbf{k\cdot r}} \frac{d^3k}{(2\pi )^3}. \end{aligned}$$

Note that we have again dropped the vector notation in the argument of $P_g(k)$ and $\xi (r)$ due to the statistical isotropy and homogeneity. In other words, they are only functions of the magnitudes of $\mathbf{k}$ and $\mathbf{r}$. In this case, it is assumed that one of the two galaxies is at the origin and the other one is at a distance *r*. It is worth noting that for a Gaussian random field, the power spectrum contains all the statistical information of the field which explains its wide use in cosmological studies.

The correlation function can be measured from a galaxy survey using estimators taking into account observational subtleties (Landy and Szalay 1993). Its theoretical counterpart is calculated from using the model predicted matter power spectrum that we discuss next. However, we use now the term matter because we refer to the dark matter field and its fluctuation, $\delta (\mathbf {k},z)$, which is traced by the galaxy fluctuation modulo some bias factor. The matter power spectrum, *P*(*k*, *z*), is defined by

47 $$\begin{aligned} \langle \delta (\mathbf{k},z)\delta (\mathbf{k'},z)\rangle = (2\pi )^3P(k,z)\ \delta _D^3(\mathbf{k}-\mathbf{k}'), \end{aligned}$$

where $\delta _D^3$ is the delta function of Dirac. *P*(*k*, *z*) is determined from theoretical grounds as we discuss next.

The standard picture of structure formation in the universe is that structures have grown by gravitational infall and clustering from primordial small fluctuations in the matter density field. These seed fluctuations would have originated from microscopic quantum fluctuations that have been blown up to macroscopic scales by cosmic inflation (Guth 1981; Bardeen et al. 1983; Albrecht and Steinhardt 1982). These primordial fluctuations would be scale invariant and described by the power spectrum (Harrison 1970; Peebles and Yu 1970; Zeldovich 1972)

48 $$\begin{aligned} P(k) \propto k^{n_s}. \end{aligned}$$

with $n_s\approx 1$. This is consistent with current observations finding that $n_s=0.9652 \pm 0.0062$, see e.g., Ade et al. (2016a) and Spergel et al. (2003).

The matter power spectrum today has evolved from this primordial spectrum while subject to a number of physical processes. During the radiation-dominated epoch, perturbations outside the horizon grow as the square of the expansion scale factor while those inside the horizon do not grow. This is due to the radiation pressure in the primordial plasma acting against gravity and preventing gravitational infall. Furthermore, as the universe expands, modes entering the horizon are also frozen. This happens until the time of matter-radiation dominance equality where modes inside the horizon can then grow. Accordingly, the scale of the horizon at this matter-radiation equality is marked in the distribution of density fluctuations and appears as a turn-over in the shape of the matter power spectrum, see e.g., Peacock (1999) and Dodelson (2003). This and other processes about mode behaviors are formulated in the so-called transfer function, *T*(*k*), (Bardeen et al. 1986; Sugiyama 1995; Eisenstein and Hu 1998). The primordial power spectrum is also enhanced by the growth factor of structure, *G*(*z*) as described in Sect. 3.2.2. In sum, the matter power spectrum today can be written as a product of the components discussed above plus a primordial amplitude determined by observations:

49 $$\begin{aligned} P(k,z)=A_s\,k^{n_s}\,T^2(k)\,G^2(z). \end{aligned}$$

In a last step, we need to connect the galaxy and matter power spectra. For that, we recall that galaxies trace the distribution of dark matter in the universe so the galaxy overdensity also traces the matter overdensity. However, this tracing is subject to some subtle galaxy bias that can be non-local and nonlinear encoding various processes and physics of structure formation, see for example discussion in Percival (2013) and references therein. On large scales, it is often assumed that one has a linear bias defined via $\delta _g(z,k)= b (z,k) \,\delta _m(z,k)$. Additionally, as we discuss in some detail in Sect. 4.3, peculiar motion of galaxies adds distortions that can be accounted for via the factor $f(z)\mu ^2$ where $\mu$ is the cosine of the angle to the line of sight. Consequently, the galaxy power spectrum can be written as

50 $$\begin{aligned} P^s_{gg}(k,\mu ,z)=A_s\,k^{n_s}\,T^2(k)\,G^2(z)\left[ b(z,k)+f(z)\mu ^2\right] ^2. \end{aligned}$$

Finally, the linear matter power spectrum above under-predicts power on small scales, and must be modified to the nonlinear matter power spectrum $P_{nl}$ to include nonlinear effects on small scales using simulations or fitting formulas for specific class of models, e.g., Peacock and Dodds (1996) and Smith et al. (2003) for $\varLambda$CDM and Zhao (2014), Hojjati et al. (2011) and Zhao et al. (2009) for *f*(*R*) MG models (see Sect. 7.4.1). The presence of screening mechanisms also complicates the picture for nonlinear modes in MG. There have been some recent interesting developments on simulation codes for MG models. Winther et al. (2015) (and references therein) presents a comparative analysis of MG N-body codes. See also Valogiannis and Bean (2017), Winther et al. (2017) where a Comoving Lagrangian Acceleration (COLA) approach was used. This last method uses fewer time-steps and resources and trades some accuracy at small scales to obtain more efficiency. A parameterization for modified gravity on nonlinear cosmological scales was also proposed in Lombriser (2016).

Relevant to our review, deviations from general relativity can affect the transfer function *T*(*k*), the growth factor $G^2(z)$, and the growth rate *f*(*z*). These can be reflected on the shape and amplitude of the galaxy power spectrum as a function of redshift and scale with some degeneracies. We discuss in the next section various observational probes, surveys and techniques that constrain and connect to the galaxy power spectrum.

## Cosmological probes of gravity theory

A well-appreciated “break” that nature has given us in cosmology is that we have two categories of measurements and probes that we can use. One category of probes constrains the expansion history and geometry of the universe via, for example, distance measurements and expansion rate. The second category constrains the growth and history of structure formation and clustering over space and time in the universe. Not only can we combine them, we can also contrast them for consistency. Indeed, combining probes from the two categories allows one to break further degeneracies between cosmological parameters and to tighten significantly the constraints, while contrasting their constraints can reveal systematics in some data sets or the need of some extensions to the underlying model. It is worth noting that some probes are sensitive to both the expansion and the growth such as CMB and weak lensing, however, for probing modifications to GR, it is rather the growth constraints that are the most useful.

Modifications to gravity change the Friedmann equations and the functions derived from them for distance and expansion observables. We give in Sect. 7 examples for some MG models. However, as we show there as well, the modified terms in the Friedmann equations can be cast into effective dark energy density and pressure leading to an effective equation of state. A number of MG models can then have an expansion history that is indistinguishable from that of $\varLambda$CDM (or a quintessence model closed to it), thus fitting cosmological distance and expansion observations equally well with the $\varLambda$CDM. However, such models can still exhibit a growth of structure that is different from that of $\varLambda$CDM so growth data can then be used as a discriminator between the theories. For this reason, studies testing GR at cosmological scales then focused on deviations from GR (or MG models) that can mimic well the expansion history of $\varLambda$CDM but can still be distinguished from it using the growth rate of structure. For that, most studies assume a $\varLambda$CDM (or a quintessence *w*CDM) background model and then use the growth probes to constrain any deviation from GR. It has been argued though that one should implement and use both expansion and growth explicitly modified functions for consistency. Also, the background can be used to test GR based on spatial curvature consistency, see e.g. Zolnierowski and Blanchard (2015).

We briefly overview various probes of gravity below and refer the reader to corresponding review articles in each sub-section. We start with probes of cosmic geometry and expansion and then follow with various probes of the growth of large-scale structure in the universe.

### Probes of cosmic geometry and expansion

Bearing in mind the strategy described above, probes of expansion and geometry have been very useful in constraining tightly background cosmological parameters such as the density parameters, the Hubble constant, the true or effective equation of state of dark energy, and then setting the stage for growth probes to constrain any deviation from GR at cosmological scales.

#### Standard candles: type Ia supernova

One of the first compelling evidences for cosmic acceleration came from Supernovae type Ia (SN Ia) observations (Riess et al. 1998; Perlmutter et al. 1999). After some corrections, SN Ia can be considered as good standard candles with an average absolute bolometric magnitude of $M_{B}\approx -19.3$; see for example Phillips (1993). The ratio of their apparent brightness to their intrinsic one can provide a measure of their luminosity distance while their redshift can be measured independently from spectroscopy. The theoretical model’s function $d_L(z)$ (or *m*(*z*)) are then fit to the data points after further corrections on the data, see for example Hamuy et al. (1996), Riess et al. (1998), Perlmutter et al. (1999) and references therein. These and other similar plots are known as the popular Hubble plots. SN Ia Hubble plots provide relative measurements of distances that can be calibrated using low redshift distance measurements such as Cepheid variable stars in the host galaxies building a distance ladder. A more practical function to use for distance estimation in cosmological analyses is the distance modulus

51 $$\begin{aligned} \mu (z)=m(z)-M=5 \log D_L + 25, \end{aligned}$$

where *M* is an effective absolute magnitude degenerate with the Hubble constant, $H_0$ and $D_L$ is the luminosity distance in units of Mpc given, for example, for a spatially flat $\varLambda$CDM universe by

52 $$\begin{aligned} D_L(z)=\frac{(1+z)}{H_0}\int ^{z}_0\frac{dz'}{\sqrt{\varOmega _m^0 (1+z')^{3}+ \varOmega _{\varLambda }^0 }}. \end{aligned}$$

$D_L(z)$ for spatially curved universes follows straightforwardly from Eqs. (26), (10), (21) and (17). Supernova data combined with other distance probe data sets can put tight constraints on background cosmological parameters. For example, supernova constraints on present time density parameters $\varOmega _m^0$ and $\varOmega _{\varLambda }^0$ have a degeneracy direction that is orthogonal to that from CMB constraints so when combined together they provide tight constraints on these parameters, see e.g., Spergel et al. (2003). We list here a number of projects and popular compilations of supernova data that we will refer to in this review including: Supernova Legacy Survey (SNLS) compilation (Conley et al. 2011); Union2.1 compilation (Suzuki et al. 2012); Joint Light Curve Analysis (JLA) constructed from SNLS, SDSS and several low-redshift SN samples, e.g., Betoule et al. (2014); Pan-STARRS sample, e.g., Rest et al. (2014); and most recently the Pantheon Sample compiled from a number of the above and other surveys which was provided in Scolnic et al. (2017).

#### Standard rulers: angular distance to CMB last scattering surface and baryon acoustic oscillations

The very early universe was made of a hot and dense plasma of electrons, baryons, mixed with a pressure-less dark matter component. Photons were trapped with this plasma via Thompson scattering. This is sometimes referred to as the baryon-photon fluid. As the universe expanded and cooled down, electrons and protons formed neutral hydrogen atoms. This is called recombination and happened at approximately 380,000 years after the Big Bang corresponding to a redshift of about 1090 (Ade et al. 2016a; Spergel et al. 2003). Shortly after that, photons decoupled from the matter and traveled freely in the universe constituting the relic background radiation that we observe today as the CMB.

Before decoupling, the baryon-photon fluid was subject to gravitational infall toward the center of overdense regions (dominated by dark matter) but then pushed back outward by the building pressure of the photons. This process created spherical sound oscillations in the plasma fluid traveling at a sound speed $c_s$ that depends on the baryons and photon density parameters. The largest comoving distance that such sound waves could have traveled from the Big Bang time to decoupling time is denoted here as $r_{s,\mathrm{com},\mathrm{dec}}$ and can be calculated as follows

$$\begin{aligned} r_{s,\mathrm{com},\mathrm{dec}}= & {} \int ^{t_\mathrm{dec}}_{0}\frac{c_sdt}{a} \\= & {} \frac{c}{\sqrt{3}}\int ^{t_\mathrm{dec}}_{0}\frac{dt}{a\sqrt{1+(3\varOmega _b)/4(\varOmega _{\gamma })a}}\\= & {} \frac{c}{\sqrt{3}H_0}\int ^{a_\mathrm{dec}}_{0}\frac{da}{\sqrt{\varOmega _r+a\varOmega _m}\sqrt{1+(3\varOmega _b)/4(\varOmega _{\gamma })a}} \end{aligned}$$

For example, if we use the values from Ade et al. (2016a) as follows: $\varOmega _{b} = 0.0492$, $\varOmega _m=0.3156$, $\varOmega _{\gamma } = 5.45\times 10^{-5}$, $\varOmega _{r} = 9.16\times 10^{-5}$ for baryon, matter, photon, and radiation (photons $+$ neutrinos) density parameters, respectively; $H_0=67.3$  km $\mathrm s^{-1}$ Mpc$^{-1}$and $z_\mathrm{dec}=1090$; then Eq. (53) above gives $r_{s,\mathrm{com},\mathrm{dec}}=144.7$ Mpc.

The corresponding physical scale is given by $r_{s,\mathrm{dec}}=a_\mathrm{dec}\times r_{s,\mathrm{com},\mathrm{dec}}=0.133$ Mpc and is called the crossing sound horizon at time of recombination. It corresponds to the largest scale at which an acoustic oscillation can be present in the baryon-photon fluid. After decoupling, these standing acoustic waves remained imprinted in the CMB temperature maps as well as in the distribution of matter structure in the universe. It constitutes a “standard ruler” that can be measured in the universe while taking into account the expansion scale factor (or redshift).

For the CMB, this standard ruler and the angular diameter distance from the observer to the CMB last scattering surface can be combined to give the angular size of the sound horizon on such a surface as

53 $$\begin{aligned} \theta _s\approx \frac{r_s}{d_A^{sls}}. \end{aligned}$$

This angle is particularly sensitive to the density and spatial curvature parameters, thus providing a good constraints on the geometry of the universe. This is related to the position of the CMB acoustic peaks (e.g., $\ell \approx \pi /\theta _s$ for the first peak). Planck has put a remarkably tight constraints on this angle as $\theta _s=(1.04106 \pm 0.00031)\times 10^{-2}$ (Ade et al. 2016a). A concise description of how the distance to last scattering using the crossing sound horizon can be found in for example Wijenayake and Ishak (2015) and more detail in Bond et al. (1997).

On the side of Baryons, part of the pattern is the presence of shells of overdense regions with comoving radius equal to the sound crossing horizon. This pattern is called the Baryon Acoustic Oscillations (BAO) and was indeed detected in various galaxy surveys as we cite further below. In BAO geometry, one is dealing with a spherical shell of matter so one can use the standard ruler along the line of sight (longitudinal) as well as in the transverse direction.

For the line-of-sight part, one can write from the line element of spacetime

54 $$\begin{aligned} H(z) = \frac{\delta z}{\delta \chi _{\parallel }}. \end{aligned}$$

One can measure $\delta z$ from spectroscopy in the survey while $\delta \chi _{\parallel }$ is the standard ruler, so one can constrain the Hubble function *H*(*z*) at some effective redshift.

For the transverse part, one can use the small angle approximation for the angle subtended by the standard ruler $\delta \chi _{\bot }$ as

55 $$\begin{aligned} d_A(z)=\frac{\delta \chi _{\bot }}{\delta \theta }, \end{aligned}$$

where $\delta \theta$ is measured from the survey while $\delta \chi _{\bot }$ is the known standard ruler so one can derive the angular diameter distance $d_A(z)$ at the effective redshift used.

Some analyses like Gaztañaga et al. (2009) and Chuang and Wang (2012) have used this approach and made very low-signal-to-noise detection because extremely large volumes are necessary for a 2D correlation function (Beutler et al. 2011). But a number of other analyses, e.g., Cole et al. (2005), Beutler et al. (2011), Blake et al. (2011b) and Anderson et al. (2012) made much stronger detections using rather a 1D correlation function and an effective projected distance defined as

56 $$\begin{aligned} D_V(z) \equiv \left[ (1+z)^2 d_A^2(z)\frac{cz}{H(z)} \right] . \end{aligned}$$

In such analyses, what is fit to the data is then the ratio

57 $$\begin{aligned} d_z=\frac{r_s(z_{\mathrm{drag}})}{D_V(z)}, \end{aligned}$$

where $r_s(z_{drag})$ is specifically the comoving crossing sound horizon when baryons became dynamically decoupled from photons. This can be understood as after photons last scattering, the baryons encountered a baryon drag epoch until redshift of about 1060 (Ade et al. 2016a). Other variations or definitions of useful effective distances like (56) have been defined and used in literature (Bassett and Hlozek 2010; Aubourg et al. 2015).

A number of measurements of BAO have been made and have become very useful in constraining the background geometry providing important complementary data to that of CMB and SN measurements. These include measurements of the BAO effective projected distance (or other measures) by for example the SDSS at $z_{\mathrm{eff}}=0.15$ (Eisenstein et al. 2005; Ross et al. 2015), the 2-degree-Field Galaxy Survey (2dFGRS) at $z_{\mathrm{eff}}=0.32$ (Cole et al. 2005), BOSS LOWZ at $z_{\mathrm{eff}}=0.32$ and CMASS at $z_{\mathrm{eff}}=0.57$ (Anderson et al. 2014), the 6dFGS measured at $z_{\mathrm{eff}}=0.106$ (Beutler et al. 2011), and WiggleZ survey at $z_{\mathrm{eff}}=0.6$ (Blake et al. 2011b).

#### Local measurements of the Hubble constant or measurements of H(z)

The Hubble constant, $H_0$, is one of the oldest cosmological parameters describing the rate of expansion of the Universe and entering all distance and geometry measurements of the universe.

A direct measurement of the local Hubble constant is possible using the cosmic distance ladder (e.g., Freedman and Madore 2010). Once this local measurement is accomplished, it can serve as a prior to further cosmological analyses. This is in particular useful if one wants to fix the background cosmology to that of a fiducial $\varLambda$CDM while allowing for the growth parameter to vary. This is useful in the case of models that can mimic a $\varLambda$CDM expansion but can still have a distinct growth rate of structure, like for example some *f*(*R*) models (see Sect. 7.4.1).

Furthermore, other cosmological probes such as the CMB infer the value of the Hubble constant by assuming and using a cosmological model. Therefore the comparison of the local measurement with that of the CMB provides an important consistency test for the underlying model. This highlights the importance of such a local measurement and we report here some of the values of the local measurements of $H_0$.

We list here some measurements of $H_0$. First, using the Hubble Space Telescope (HST) Key Project and Cepheid calibration of distances to 31 galaxies and other calibrated secondary distance indicators (Type Ia and Type II Supernovae), Freedman et al. (2001) reported $H_0 = 72 \pm 8$  km $\mathrm s^{-1}$ Mpc$^{-1}$. A decade later, Riess et al. (2011) used HST new camera observations of over 600 Cepheids in host galaxies of 8 Type Ia SN. This allowed the authors to calibrate the SN magnitude-redshift relation and to obtain a much more precise value of $H_0 = 73.8 \pm 2.4$  km $\mathrm s^{-1}$ Mpc$^{-1}$. Efstathiou (2014) used different outlier rejection criteria for the Cepheids and obtains $H_0 = 70.6 \pm 3.3$  km $\mathrm s^{-1}$ Mpc$^{-1}$. He also obtained $H_0 = 72.5 \pm 2.5$  km $\mathrm s^{-1}$ Mpc$^{-1}$when the H-band period-luminosity relation is assumed to be independent of metallicity using other combined distance anchors. Freedman et al. (2012) used HST with further calibrations from the Spitzer Space Telescope to measure $H_0 = 74.3 \pm 1.5 \,{\mathrm{(statistical)}}\, \pm 2.1\, {\mathrm{(systematic)}}$  km $\mathrm s^{-1}$ Mpc$^{-1}$. Most recently, Riess et al. (2016), used four geometric calibration methods of Cepheids to obtain $73.24\pm 1.74$  km $\mathrm s^{-1}$ Mpc$^{-1}$.

It is worth noting here that a tension seems to persist between the local measurement values and the lower value obtained from Planck, i.e., $H_0=66.93 \pm 0.62$  km $\mathrm s^{-1}$ Mpc$^{-1}$. This tension has been the subject of numerous discussions in recent literature offering different perspectives (Bernal et al. 2016; Lin and Ishak 2017a, b; Luković et al. 2018; Wang et al. 2017; Haridasu et al. 2017; Zhang et al. 2018; Gómez-Valent and Amendola 2018; Abbott et al. 2018a). As we discuss further below in some of the sub-sections (see e.g., Sects. 9.7 and 9.9), some authors find that some modified gravity models reduce or alleviate the tension in the Hubble parameter (see e.g., Barreira et al. 2014a; Belgacem et al. 2018b).

However, other approaches have been used to determine local measurement of $H_0$. Some time ago, Gott et al. (2001) developed and used a median statistics method that provides an alternative of $\chi ^2$ likelihood methods and requires fewer assumptions about the data. They found at that time a median value of $H_0=67$ km $\mathrm s^{-1}$ Mpc$^{-1}$with $\pm 2$ km $\mathrm s^{-1}$ Mpc$^{-1}$statistical errors (95% CL) and $\pm 5$ km $\mathrm s^{-1}$ Mpc$^{-1}$statistical errors (95% CL) from using 331 measurements of $H_0$ from by Huchra’s compilation. Some time later Chen and Ratra (2011) used the same method and the final compilation of Huchra with 553 measurements finding a median of $H_0 = 68 \pm 5.5$ km $\mathrm s^{-1}$ Mpc$^{-1}$(at 95% CL) including statistical and systematics uncertainties. Most recently, Chen et al. (2017) used rather the Hubble function *H*(*z*) with 28 measurements at intermediate redshifts $0.07\le z \le 2.3$ in order to determine the local Hubble constant, $H_0$. They find for the spatially flat and non-flat $\varLambda \mathrm {CDM}$ model, $H_0=68.3^{+2.7}_{-2.6}$. The authors stress that this value is consistent with the low value obtained with the previous work using the median statistics. They also note that this value is consistent with the low value measured by Planck while it includes the high value from local measurement in the previous paragraph within the 2$\sigma$ bound. Further work using, *H*(*z*), was carried (Moresco et al. 2016; Farooq et al. 2017; Yu et al. 2018) where the authors put constraints on a cosmological deceleration-acceleration transition with various levels of confidence. Capozziello et al. (2014) made some first developments to constrain *f*(*R*) models using the cosmological deceleration-acceleration transition redshift. They required that the model reduces to $\varLambda \mathrm {CDM}$ at $z=0$ but they parametrize possible departures from it at higher redshifts in terms of a two-parameter logarithmic correction. They found that the transition in this model happens at a redshift consistent with using type Ia supernova apparent magnitude data and Hubble parameter measurements. Finally, Gómez-Valent and Amendola (2018) followed on the *H*(*z*) approach using cosmic chronometers, Type Ia supernovae, Gaussian processes and a novel Weighted Polynomial Regression method to find $H_0=67.06\pm 1.68$ km $\mathrm s^{-1}$ Mpc$^{-1}$which is in agreement with low values and in 2.71-$\sigma$ tension with the local measurement of Riess et al. They also determine a more conservative value of $H_0=68.45\pm 2.00$ which is still about 2-$\sigma$ tension with the value from Riess et al. further above. With future precise data from for example, GAIA, and other experiments, one will hopefully get to the bottom of these tensions.

### Weak gravitational lensing

Trajectories of photons traveling to us from remote galaxies get deflected along the line of sight by matter overdensities in the intervening medium. This is called gravitational lensing. Depending on the positions of the sources and lenses relative to the observer, these deflections can result in strong, intermediate, or weak lensing. Strong and intermediate lensing provides spectacular multiple images such as Einstein rings and crosses (Cabanac et al. 2005; Belokurov et al. 2009), giant arcs, and arclets (Hennawi et al. 2008). Less impressive but so abundant, weak lensing consists of tiny distortions to the shapes of millions and millions of galaxies that can be accounted for using statistical techniques and turned into a powerful cumulative signal which probes the cosmology of the intervening deflector medium including any modification to gravity theory at cosmological scales.

Weak lensing at cosmological scales, also called cosmic shear, is quantified by the shear of images that tend to transform circular shapes into elliptical ones and is represented by the complex-quantity $\gamma$, and the convergence, $\kappa$, that represents the magnification of these images. In this weak regime, the two effects are very small, of the order of a few percent at most and equal, thus used interchangeably. To linear order, the shear is a good approximation to the reduced shear that is determined from the measured shapes (ellipticies) of galaxy images and on scales typically used in weak lensing analyses to date, see e.g., reviews Bartelmann and Schneider (2001) and Kilbinger (2015).

Cosmic shear surveys measure ellipticities and positions of galaxies in the sky and then build from them pairs and triplets called 2- and 3-point correlation functions that can be compared to theoretical models using the lensing power spectrum and bispectrum that are derived from the formalism as follows (we use a mixture of steps from Kilbinger 2015; Troxel and Ishak 2015).

The mean convergence can be written as a weighted projection of the overdensities along the line of sight

58 $$\begin{aligned} \kappa (\varvec{\theta }) = \frac{3 H_0^2 \varOmega _{\mathrm{m}}}{2 c^2} \int \limits _0^{\chi _{_{\mathrm{H}}}} {\mathrm{d} \chi } \frac{g(\chi )}{a(\chi )} f_K(\chi ) \, \delta (f_K(\chi ) \varvec{\theta }, \chi ), \end{aligned}$$

where $\chi _{_{\mathrm{H}}}$ is is the comoving coordinate at the horizon, $f_K(\chi )$ is given by Eq. (10), and $g(\chi )$ is defined as

59 $$\begin{aligned} g(\chi ) = \int \limits _\chi ^{\chi _{_{\mathrm{H}}}} \mathrm{d} \chi ^\prime \, n(\chi ^\prime ) \frac{f_K(\chi ^\prime - \chi )}{f_K(\chi ^\prime )}, \end{aligned}$$

and represent the lensing efficiency at a distance $\chi$. The convergence 2-point correlation functions is constructed as

60 $$\begin{aligned} \langle \kappa (\varvec{\theta _1}) \kappa (\varvec{\theta _2}) \rangle , \end{aligned}$$

where again $\langle \,\,\rangle$ denotes the ensemble average. Now, the convergence scalar field can be decomposed into multipole moments of the spherical harmonics as

61 $$\begin{aligned} \kappa (\varvec{\theta })=\sum _{lm}\kappa _{lm}Y^m_l(\varvec{\theta }), \end{aligned}$$

where

62 $$\begin{aligned} \kappa _{lm}=\int d\hat{\theta } \kappa (\varvec{\theta },\chi ) Y^{m*}_l(\varvec{\theta }). \end{aligned}$$

The convergence power spectrum $P_\kappa (\ell )$ is then defined by

63 $$\begin{aligned} \left\langle \kappa _{lm} \kappa _{l'm'} \right\rangle =\delta _{ll'} \delta _{mm'} P_\kappa (\ell ). \end{aligned}$$

In the Limber approximation (Limber 1953), it is given by Kaiser (1992, 1998) and Jain and Seljak (1997):

64 $$\begin{aligned} P_\kappa (\ell ) = \frac{9}{4} \, \varOmega _{\mathrm{m}}^2 \left( \frac{H_0}{c} \right) ^4 \int _0^{\chi _{_{\mathrm{H}}}} \mathrm{d} \chi \, \frac{g^2(\chi )}{a^2(\chi )} P_\delta \left( k = \frac{\ell }{f_K(\chi )}, \chi \right) , \end{aligned}$$

where $P_\delta \left( k = \frac{\ell }{f_K(\chi )}, \chi \right)$ is the 3D nonlinear matter power spectrum (Sect. 3.2.3).

As we discuss further below, modifications to gravity will alter the growth factor function and the matter power spectrum (49) as well as Weyl potential Eq. (88). A generalization of the above steps to the convergence 3-point correlation, $\langle \kappa (\varvec{\theta _1}) \kappa (\varvec{\theta _2}) \kappa (\varvec{\theta _3}) \rangle$, provides the convergence bispectrum

65 $$\begin{aligned} B_{\kappa }(\ell _1,\ell _2,\ell _3)&=\int _0^{\chi _{_{\mathrm{H}}}}d\chi \frac{W^3(\chi )}{f_K(\chi )^4(\chi )}\nonumber \\&\quad B_{\delta }\left( k_1=\frac{\ell _1}{f_K(\chi )},k_2=\frac{\ell _2}{f_K(\chi )},k_3=\frac{\ell _3}{f_K(\chi )};\chi \right) , \end{aligned}$$

where we encapsulated the other factors into the $W(\chi )$ as follows,

66 $$\begin{aligned} W(\chi )&=\frac{3}{2}H_0^2\frac{\varOmega _m}{a(\chi )}\int _{\chi }^{\chi _{_{\mathrm{H}}}}d\chi ' n(\chi ')f_K(\chi )\frac{f_K(\chi '-\chi )}{f_K(\chi ')}, \end{aligned}$$

and $B_{\delta }(k_1=\frac{\ell _1}{f_K(\chi )},k_2=\frac{\ell _2}{f_K(\chi )},k_3=\frac{\ell _3}{f_K(\chi )};\chi )$ is the 3D matter bispectrum.

Next, we describe a few more steps on how comparison to observed ellipticities of galaxies is performed. We note that the ellipticity is also represented as a complex number field just like the shear. For a galaxy with intrinsic ellipticity $\epsilon ^{\mathrm{int}}$, cosmic shear modifies this ellipticity [via combination with the reduced shear Kilbinger (2015)] such that the observed ellipticity in the weak-lensing regime is given by

67 $$\begin{aligned} \varepsilon \approx \varepsilon ^{\mathrm{int}} + \gamma . \end{aligned}$$

If we average over a large number of galaxies, we expect the averaged first term to drop due to the assumed random intrinsic ellipticity of galaxies (any residual is usually put into a noise term) so the observed ellipticity components can be used as an estimator of the complex shear, i.e., $\gamma = \left\langle \varepsilon \right\rangle$.

Additionally, galaxies also have intrinsic alignments that provide signals contaminating the lensing signal. These intrinsic alignments are due to processes of galaxy formation in the gravitational field. They need to be isolated and mitigated for weak lensing to reach its full potential. See the following reviews for this topic (Troxel and Ishak 2015; Kirk 2015).

In practice, the two components of the shear can be identified as a tangential component with respect to the 1-axis, i.e., $\gamma _t=-\gamma _1$, and a cross-component, i.e., $\gamma _\times =-\gamma _2$, obtained by a rotation of an angle $+\pi /4$ from the tangential component. These components are used to build 2-point correlators that can be combined to construct two practical and often-used 2-point correlations from observations as follows (Miralda-Escude 1991),

68 $$\begin{aligned} \xi _+(\theta )&= \langle \gamma _{\mathrm{t}} \gamma _{\mathrm{t}} \rangle (\theta ) + \langle \gamma _\times \gamma _\times \rangle (\theta ); \quad \nonumber \\ \xi _-(\theta )&= \langle \gamma _{\mathrm{t}} \gamma _{\mathrm{t}} \rangle (\theta ) - \langle \gamma _\times \gamma _\times \rangle (\theta ) . \end{aligned}$$

The explicit corresponding weighted estimators from ellipticities can be found in for example Kilbinger (2015).

Finally, in order to compare the correlation functions above to their theoretical counterparts, the shear 2-point correlations are related to the convergence power spectrum as follows

69 $$\begin{aligned} \xi _+(\theta )&= \frac{1}{2\pi } {\displaystyle \int } \mathrm{d} \ell \, \ell \,\mathrm{J}_0(\ell \theta ) P_\kappa (\ell ),\nonumber \\ \xi _-(\theta )&= \frac{1}{2\pi } {\displaystyle \int } \mathrm{d} \ell \, \ell \,\mathrm{J}_4(\ell \theta ) P_\kappa (\ell ), \end{aligned}$$

where $J_n(x)$ are the n-th order Bessel function of the first kind.

Finally, it is worth mentioning that cosmic shear analyses perform a powerful technique called tomography where the data is split into redshift bins. This strongly probes the growth rate of large scale structure. With tomography, the 2-point correlation functions between two bins i and j is specialized as

70 $$\begin{aligned} \xi _{\pm }^{ij}(\theta )&= \frac{1}{2\pi } {\displaystyle \int } \mathrm{d} \ell \, \ell \,\mathrm{J}_{0/4}(\ell \theta ) P_\kappa ^{ij}(\ell ), \end{aligned}$$

where the corresponding power spectrum is given by

71 $$\begin{aligned} P_\kappa ^{ij}(\ell ) = \frac{9}{4} \, \varOmega _{\mathrm{m}}^2 \left( \frac{H_0}{c} \right) ^4 \int _0^{\chi _{\mathrm{lim}}} \mathrm{d} \chi \, \frac{g^i(\chi )g^j(\chi )}{a^2(\chi )} P_\delta \left( k = \frac{\ell }{f_K(\chi )}, \chi \right) . \end{aligned}$$

**Fig. 2:**
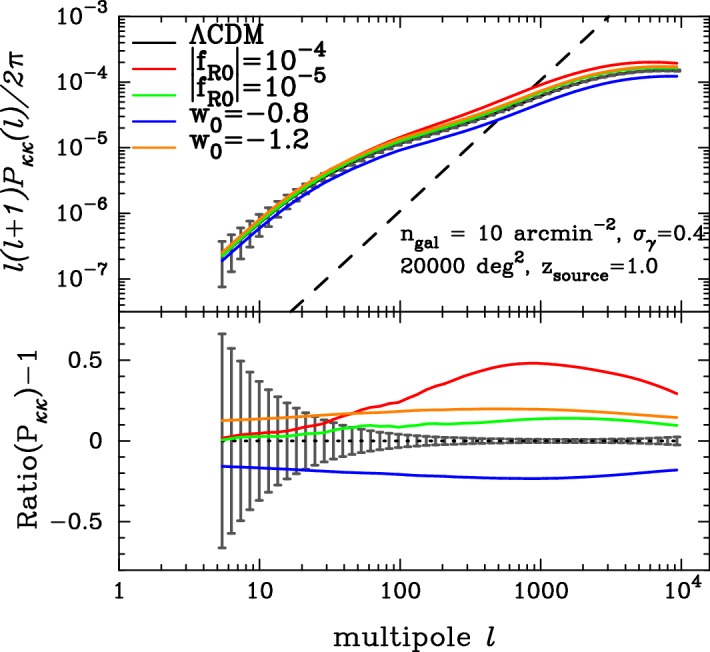
Top panel: convergence power spectra for *f*(*R*) models (see Sect. 7.4.1), dynamical dark energy models and the $\varLambda$CDM standard model. Error bars are for the survey indicated on the figure—sky coverage of 20,000 square degrees with a galaxy density number of 10 per arcminutes squared. The dashed line corresponds to the shot noise term of auto power spectrum. Bottom panel: Ratio between $\varLambda$CDM model and *f*(*R*) models or *w*CDM models Image reproduced with permission from Shirasaki et al. (2016), copyright by the authors

Modifications to gravity are constrained by weak lensing via the growth factor function and any other changes in the matter power spectrum (49) as well as the modifications to the Weyl potential equation (88). The latter change is usually captured phenomenologically by the addition of the MG parameter factor, $\varSigma (k,\chi )^2$, in the integrand of equation (71). This highlights the sensitivity and importance of WL surveys in testing deviations from GR. We reproduce here the right-top panel of Fig. 1 from Shirasaki et al. (2016) (Fig. 2 here) comparing convergence power spectra of two *f*(*R*) models, two dynamical dark energy models and the standard $\varLambda$CDM model.

Recent cosmic shear surveys have already provided us with several analyses to constrain modification to GR or some classes of MG models that we discuss further below. These include, CFHTLenS (Heymans et al. 2013; Simpson et al. 2013), KIDS (Joudaki et al. 2017; Hildebrandt et al. 2017), and KIDS+2dFLenS (Amon et al. 2017; Joudaki et al. 2018). It is expected that LSST (https://www.lsst.org/; LSST Dark Energy Science Collaboration 2012) and WFIRST (https://wfirst.gsfc.nasa.gov/; Spergel et al. 2015), and Euclid (http://sci.esa.int/euclid/; Amiaux et al. 2012) will be particularly effective in constraining beyond $\varLambda$CDM model including deviations from GR and a number of classes of MG theories (Jennings et al. 2012; Xu 2015; Kwan et al. 2012; Tsujikawa 2015; Bellini et al. 2016; Okumura et al. 2016).

### Galaxy surveys: clustering and redshift space distortions (RSD)

In the recent years, a wealth of cosmological information has been provided to us from spectroscopic redshift surveys such as SDSS, BOSS, 2dF, 6dF and WiggleZ. From galaxy redshift surveys one can measure the isotropically averaged galaxy power spectrum or the galaxy correlation function and thus put constraints on cosmological parameters as well as MG parameters and models. This can be done via constraints on various factors in the galaxy power spectrum (50) discussed in Sect. 3.2.3. For example, we reproduce Fig. 2 from Barreira et al. (2014a) (see Fig. 4 here) showing in the bottom panel the data points from the SDSS-DR7 Luminous Red Galaxy host halo power spectrum of Reid et al. (2010) against Galilean MG models and $\varLambda$CDM with massive neutrinos (Barreira et al. 2014a).

Additionally, there are Lyman-$\alpha$ surveys (sub-surveys) that can determine the frequency, density and temperature of matter clouds containing neutral hydrogen between the observer and remote quasars. Each spectrum gives information about multiple structures along the line of sight and that traces the distribution and growth of matter along the line of sight, see for example Weinberg et al. (2003), McDonald et al. (2006) and Font-Ribera et al. (2013).

In regards to testing deviations from GR using galaxy redshift surveys, it seems that “the good comes from the bad”. Indeed, observations along the line of sight are also subject to distortions due to the fact that we make measurements in the redshift space and then convert them to the real space. It turns out that these distortions are a rich source of cosmological information which has at its forefront the redshift space distortions (RSD) that are very sensitive to the growth rate of structure and the gravity theory governing such a growth. We briefly describe below some aspects of the RSD formalism and refer the reader to specialized reviews on the topic (Samushia et al. 2014; Blake et al. 2011a; Hamilton 1998; Percival and White 2009; Percival 2013) and references therein.

Redshifts to remote cosmic objects such as galaxies are distorted by peculiar velocities of these objects with respect to the Hubble flow. These peculiar velocities follow large-scale infall of matter toward over dense regions in the cosmic web and by that they can trace the growth rate of large-scale structure. The distortions can be observed in the redshift space as two main effects. The first one is due to random peculiar velocity distribution of galaxies in clusters that produce a Doppler effect stretching out a cluster of galaxies in the radial direction on redshift maps. This radial stretching points to the observer and was dubbed by the “fingers-of-god” (FoG) effect, see e.g., the seminal papers by Kaiser (1987) and Hamilton (1998). See also earlier work by Jackson (1972). The FoG effect happens at relatively smaller nonlinear scales. The second effect happens on larger scales where the peculiar velocities are not random but directed coherently toward the center of overdense regions (center of mass of clusters). It is a subtle blend of effects that combine to produce a flattening of the distribution on larger scales on redshift survey maps, sometimes dubbed as the “pancakes-of-god”, see e.g., Hamilton (1998), Percival and White (2009) and Percival (2013). The related equations are as follows.

A point in the redshift space can be related to the real space by

72 $$\begin{aligned} \mathbf {s}({\mathbf{r}} ) = {\mathbf{r}} + v_r ({\mathbf{r}} ) {\hat{\mathbf{r}}}, \end{aligned}$$

where $v_r$ is the peculiar velocity projected in the radial direction. Next, we recall the linearized continuity equation

73 $$\begin{aligned} \beta \delta _m + \bar{\nabla } \cdot \bar{v} = 0 \, \end{aligned}$$

where *v* is the matter velocity field, $\beta (z) \equiv f(z)/b(z)$ and *b*(*z*) is the galaxy bias .

Using the Jacobian between the redshift and real spaces, conservation of galaxy number in the two spaces, the continuity equation and a few steps, it is straightforward to derive (Kaiser 1987; Hamilton 1998)

74 $$\begin{aligned} \delta ^s_g(k) = \left( 1+\beta \mu ^2 \right) \delta ^r_g(k), \end{aligned}$$

where the $\mu$ is the cosine of the angle with the line of sight.

Using (74) and a linear galaxy bias, the corresponding power spectra are related as follows

75 $$\begin{aligned} P^s_g(k,\mu ,z)= & {} b(z)^2\left[ 1 + \beta (z) \mu ^2 \right] ^2 P_m^r(k,z) \end{aligned}$$

76 $$\begin{aligned}= & {} \left[ b (z) + f(z) \mu ^2 \right] ^2 P_m^r(k,z) \, , \end{aligned}$$

where in the last line, we split *b*(*z*) and *f*(*z*) on purpose and note that from the matter power spectrum on the right comes its amplitude, e.g., $\sigma _8$ that is then degenerate with *f*(*z*) in such a measurement. This illustrates why RSD surveys probe $b(z)\sigma _8$ and $f(z)\sigma _8$, unless the degeneracies are broken by other means.

Equation (75) gives the linear RSD at large-scales,^1^ while the nonlinear FoG effect can be modeled by a damping factor multiplying the power spectrum and often chosen to be an exponential (Lorentzian) or Gaussian form Percival and White (2009)

78 $$\begin{aligned} F_{\mathrm{Lorentzian}}(k,\mu ^2)= & {} [1+(k\sigma _p\mu )^2]^{-1}, \end{aligned}$$

79 $$\begin{aligned} F_{\mathrm{Gaussian}}(k,\mu ^2)= & {} \exp [-(k\sigma _p\mu )^2]. \end{aligned}$$

It is then customary to multiply Eqs. (75) and (78) to combine the effect with caution though about some limitations and the need for some accurate simulations as discussed in for example Percival and White (2009). Indeed, other combined models including contributions from nonlinear effects and numerical simulations are used to fully explore RSD modeling and observations and we refer the reader to the following RSD reviews in the literature (Hamilton 1998; Percival and White 2009; Percival 2013) and references therein.

**Fig. 3:**
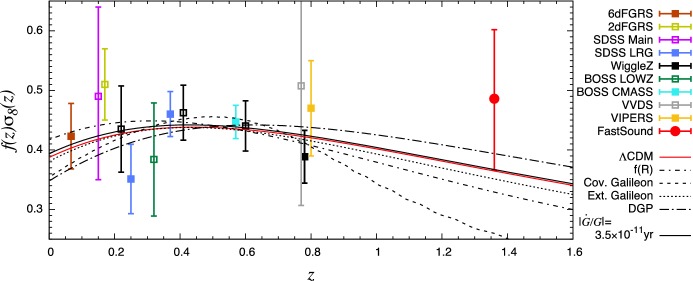
Growth rate $f(z)\sigma _8(z)$ measurements for redshift range $0<z<1.55$ and theoretical predictions from standard GR-$\varLambda$CDM model and MG models *f*(*R*) (see Sect.7.4.1), covariant Galileons (see Sect. 7.3.1), extended Galileons, DGP (see Sect. 7.5.2), and models with varying gravitational constant. The constraint obtained from Subaru FastSound sample at $1.19<z<1.55$ (Okumura et al. 2016) is plotted as the big red point. The other results include the 6dFGS, 2dFGRS, SDSS main galaxies, SDSS LRG, BOSS LOWZ , WiggleZ, BOSS CMASS, VVDS, and VIPERS surveys at $z<1$. Predicted $f\sigma _8$ from GR-$\varLambda$CDM with the amplitude determined by minimizing their $\chi ^2$ is shown as the red solid line. The data points used for the $\chi ^2$ minimization are denoted as the filled-symbol points. The other curves are predictions from MG models as indicated on the right Image reproduced with permission from Okumura et al. (2016), copyright by the authors

Finally, it is worth noting that measurement of RSD are degenerate with another effect called the Alcock–Paczynski effect (Alcock and Paczynski 1979) which is caused by the conversion of angles and redshifts measured in redshift space to physical distances and Hubble function in the real space. If the theoretical cosmological model used is significantly different from the true model then further distortions are introduced in this process. These can be confused with the RSD effects and need to be accounted for. This results in a further multiplicative expression to Eq. (75) with one or two more parameters. See for example, treatments and discussions in Ballinger et al. (1996), Simpson and Peacock (2010), Samushia et al. (2012) and Montanari and Durrer (2012). This is well summarized in the following equation from Raccanelli et al. (2015):

80 $$\begin{aligned} P_g^{\mathrm{s}}(k',\mu ',\alpha _\bot ,\alpha _{||},\mathbf{p})= \frac{(b+\mu '^{2}f)^2}{\alpha _{\bot }^2\alpha _{||}} P_m^{\mathrm{r}}\left[ \frac{k'}{\alpha _\bot }\sqrt{1+\mu ^{'2}\left( \frac{1}{F^2}-1\right) }\right] , \end{aligned}$$

where $\mathbf{p}$ are the cosmological parameters of the real-space power-spectrum and the primed quantities are the observed quantities that have been introduced here to distinguish them from the real quantities as follows: $k'$ and $\mu '$ are the observed wavevector and angle; their relation to the real quantities is given by $k'_{||}=\alpha _{||}k_{||}$, $k'_\bot =\alpha _\bot k_\bot$, $\mu '=\frac{k_{||}'}{\sqrt{k_{||}'+k_\bot '}}$; $F=\alpha _{||}/\alpha _\bot$, with $\alpha _{||}=\frac{H^{\mathrm{fid}}}{H^{\mathrm{real}}}$ and $\alpha _\bot =\frac{D^{\mathrm{real}}}{D^{\mathrm{fid}}}$ the ratios of angular and radial distances between the fiducial and real cosmological models, see Raccanelli et al. (2015).

An important aspect of RSD analyses is to use measurements of the correlation function from galaxy redshift surveys and then compare them to galaxy theoretical power spectrum or its Legendre decomposition in order to estimate $f\sigma _8$ and $b\sigma _8$ at different effective redshifts.

For our review, we stress that modifications to gravity enter into the $f(z)\sigma ^8$ term in Eq. (80) and also into the $G^2(z)$ contained in the matter power spectrum. RSD measurements are thus very important in constraining deviations from GR affecting Poisson equation (29). While current error bars on measurements are still too large to exclude a number of contenders to GR, RSD is considered one of the most promising probes of gravity theories and has been used in a number of analysis as we discuss further below. For example, it has been shown in Okada et al. (2013) that RSD can already exclude some covariant Galileon MG models (see Sect. 7.3.1) to high level of confidence (Okada et al. 2013). We reproduce Fig. 17 from Okumura et al. (2016) (see Fig. 3) for a number of $f\sigma _8$ measurements to date along with GR-$\varLambda$CDM and five MG models (see discussion in Sect. 6.2).

Current RSD data include for example measurements from 6dFGS (Beutler et al. 2012), 2dFGRS (Cole et al. 2005), SDSS LRG (Samushia et al. 2012), BOSS LOWZ (Tojeiro et al. 2012), BOSS CMASS (Anderson et al. 2014), VVDS (Guzzo et al. 2008), VIPERS (de la Torre et al. 2013), WiggleZ Dark Energy Survey (Blake et al. 2012; Parkinson et al. 2012), and Subaru FMOS galaxy redshift survey (FastSound) (Okumura et al. 2016). A compilation of 34 points with corrections from model dependencies can be found in Nesseris et al. (2017). It is worth noting that when using $f \sigma _8$ data to constrain modified gravity models, one has to make sure no assumptions of the $\varLambda \mathrm {CDM}$ model are kept in the data points due to calibration using $\varLambda \mathrm {CDM}$ mocks. See for example the following papers that performed validation analyses of $f \sigma _8$ constraints in MG models and pointed out to possible biases (Taruya et al. 2014; Barreira et al. 2016; Bose et al. 2017).

In addition to linear scales, RSD and velocity power spectra were shown to be a promising probe of deviations from gravity. Jennings et al. (2012) used large volume N-body simulations to study dark matter clustering in redshift space in *f*(*R*) modified gravity models (see Sect. 7.4.1). The nonlinear matter and velocity fields were resolved to a high level of accuracy over a broad range of scales for f(R) models. The analysis found significant deviations from the clustering signal in GR, with an enhanced boost in power on large scales and stronger damping on small scales in the *f*(*R*) models at redshifts z below 1. In particular, they found that the velocity power spectrum is a strong discriminator between *f*(*R*) and GR suggesting that the extraction of the velocity power spectrum from future galaxy surveys is a promising method to constrain deviations from GR. See also (Hellwing et al. 2014) on the galaxy velocity field and a signature of MG.

It is worth mentioning here that almost a decade ago RSD already attracted a lot of attention after a study in Guzzo et al. (2008) using the VIMOS-VLT Deep Survey (VVDS) measured the anisotropy parameter $\beta (z=0.77) = 0.70 \pm 0.26$, which corresponds to a growth rate of structure $f(z=0.77) = 0.91 \pm 0.36$ consistent with GR and $\varLambda$CDM, but with too large errors leaving room for other possibilities. We present recent constraints from RSD on gravity in Sect. 6.2.

### Cosmic microwave background radiation

This relic radiation that we call the CMB is among the most powerful cosmological probes. Not only does it constrain the background geometry (as discussed in Sect. 4.1.2) but it also constrains the growth of structure in the universe. The information in the CMB is expressed into temperature and polarization power spectra. These spectra have primary anisotropies that were imprinted at the surface of last scattering and also secondary anisotropies that happen later while the CMB photons are traveling in the intervening medium.

CMB spectra provide via their primary anisotropies a powerful probe of the early universe to constrain cosmological parameters. It is complementary to other geometry probes such as supernova and BAO that probe the later times. CMB by itself can already tightly constrain background parameters such as the Hubble constant, the matter density and the effective dark energy density parameters. In combination with other probes, it can also tightly constrain an effective dark energy equation of state.

Most relevant to dark energy and modification to GR at cosmological scales, are the secondary anisotropies that constrain scalar mode perturbations and the growth of large-scale structure. These are the Integrated Sachs–Wolfe–Effect (ISW) that affect the spectrum at small multipoles (large angular scales) (Sachs and Wolfe 1967; Kofman and Starobinskij 1985), Lensing of the CMB (Blanchard and Schneider 1987; Cole and Efstathiou 1989; Linder 1997; Seljak 1996) that affects the spectrum progressively at high multipoles (small angular scales), and the Sunyaev–Zel’dovich (SZ) effect at even higher multipoles (smaller angular scales). We review the former two effects in the next sub-sections.

Finally, it is worth mentioning that a general practice in using CMB in analysis where geometry constraints are compared to growth constraints, the spectra are split into low and high multipoles as follows. Low multipoles ($\ell < 30$) are used to constrain the growth while the higher multipoles ($30 \le \ell \le 2508$) are more sensitive to the background geometry via the position of the acoustic peaks and are used for that.

#### Integrated Sachs–Wolfe (ISW) effect

The Integrated Sachs–Wolfe (ISW) effect is a secondary anisotropy in the CMB temperature fluctuations that is caused by time variations in the gravitational potentials (Sachs and Wolfe 1967; Kofman and Starobinskij 1985; Rees and Sciama 1968). In this review, we focus on the late-time ISW that can be caused by a Dark Energy component or a modification to gravity that can effect the evolution of the potentials associated with large-scale structures and voids. Namely, CMB photons traveling to us encounter potential wells due to large structures. They gain energy while falling down the potential wells but then lose it back while climbing out of them except for a small difference left due to a stretching in the potential well caused by repulsive Dark Energy or Modified gravity that happened during the photons’ journey through the potential. This results in a net gain in energy for the photons coming out of the potential’s well. The opposite scenario happens to photons when they travel across large voids (potential hills) causing a net loss in their energy. The effect is given by

81 $$\begin{aligned} \frac{\delta T}{T}(\hat{n})= -\int ^{\eta _{*}}_{\eta _{0}}d\eta \frac{\partial (\varPsi +\varPhi )}{\partial \eta } \end{aligned}$$

where *T* is the CMB temperature, $\eta _{*}$ is the conformal time at CMB surface and $\eta _{0}$ at the observer. We note that spatial curvature can also cause such a variation (Kamionkowski 1996) but we assume here spatial flatness in accordance with current observational constraints.

The ISW effect modifies the CMB temperature power spectrum at the largest angular scales with multipoles $\ell \le 10$ affecting the height of the left tail of the spectrum. The first detections of the ISW effect were done by cross-correlating the WMAP CMB temperature data with galaxy density surveys, see for example Boughn and Crittenden (2004), Fosalba et al. (2003), Nolta et al. (2004), Corasaniti et al. (2005), Padmanabhan et al. (2005), Vielva et al. (2006) and Giannantonio et al. (2012) and later on by cross-correlating Planck with large scale structure data (Ade et al. 2014b, 2016d). Other methods using stacking of CMB fields at coordinates coinciding with known superstructures have also led to detection, see for example Granett et al. (2008), Pápai et al. (2011) and Ade et al. (2014b). The ISW was also detected through the ISW-lensing bispectrum using Planck data only (Ade et al. 2016d).

**Fig. 4:**
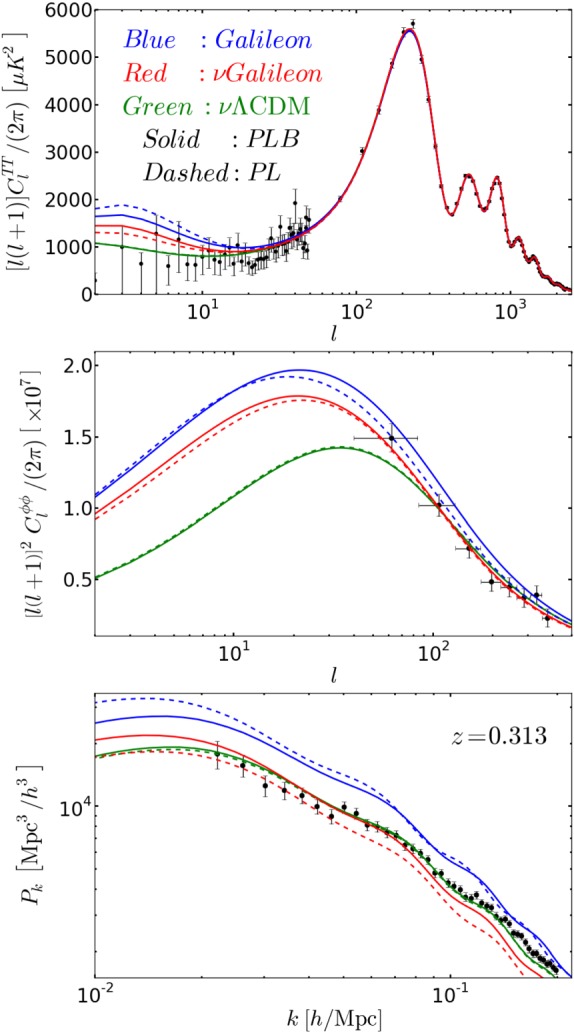
These plots illustrate the differences between $\varLambda \mathrm {CDM}$ and Galileon models (see Sect. 7.3.1), with and without massive neutrinos. The Galileon models have background Friedmann equations that contain a scalar-field energy density contribution that generates late time cosmic acceleration and has an evolution consistent with observations and thus similar to that of a $\varLambda \mathrm {CDM}$ model. The Galileon scalar field here also affects linear perturbations and is not coupled to matter. The effect of the Galileon field considered here is focused on large-scale structure. The Top: CMB temperature power spectra showing the ISW effect at low multipoles. Middle: CMB lensing potential spectra. Bottom: linear matter power spectra. The models plotted in dashed lines indicate their best fit models to Ade et al. (2014c) temperature data, WMAP9 polarization data (Hinshaw et al. 2013), and Planck-2013 CMB lensing (Ade et al. 2014d). They note these as PL models. The solid lines indicate their best fits to CMB data (i.e., PL) plus BAO measurements from 6dF, SDSS DR7 and BOSS DR9. They note these as PLB models. The models correspond to best-fitting base Galileon modified gravity model (in blue), ${\nu } {{\mathrm{Galileon}}}$ (in red) and ${\nu } \varLambda {{\mathrm{CDM}}}$ (in green). For the last two models, the authors added massive neutrino. In the upper and middle panels, the data points show the power spectrum measured by the Planck satellite (Ade et al. 2014c). In the lower panel, the data points show the SDSS-DR7 Luminous Red Galaxy power spectrum of Reid et al. (2010), but scaled down to match the amplitude of the best-fitting ${\nu } {{\mathrm{Galileon}}}$ (PLB) model (Barreira et al. 2014a). We refer to this figure from various parts of the text Image reproduced with permission from Barreira et al. (2014a), copyright by APS

By changing the gravitational potentials [as in (88)] and their time evolution (growth), MG models affect the ISW and change the very-left end of the CMB power spectrum. We reproduce Fig. 2 from Barreira et al. (2014a) (see Fig. 4 here) where the top panel shows the ISW effect for various Galileon MG models (see Sect. 7.3.1) and the $\varLambda$CDM model augmented by massive neutrinos. As we discuss further in Sect. 9.7, such an effect played a major role in ruling out the cubic Galileon models and putting very stringent constraints on the quartic and quintic ones. It is worth noting though that since the ISW effect enters only on the largest angular scales, its constraining power is limited by cosmic variance. However, cross-correlating CMB with large-scale structure tracers such as galaxies enhances its measurement significance and usefulness as we listed above.

As we describe further below, the ISW effect has been used extensively to constrain deviations from GR in conjunction with other data sets and plays a central role in obtaining such constraints.

#### CMB lensing

Just as in cosmic shear, CMB photons traveling to us from the surface of last scattering are subject to deflections by large-scale structure and mass concentrations along the intervening medium. These deflections change the trajectories of photons and affect the CMB temperature and polarization maps observed in the form of very small distortions that can be statistically collected and analyzed from high-precision CMB experiments (Blanchard and Schneider 1987; Cole and Efstathiou 1989; Linder 1997; Seljak 1996). This lensing smears out the CMB temperature power spectrum and produces non-guaussianities in the temperature and polarization maps, generating 3- and 4-point correlations (Bernardeau 1998; Zaldarriaga and Seljak 1999; Okamoto and Hu 2003), and converting E-mode polarization of the CMB photons into lensing B-mode (Zaldarriaga and Seljak 1998). CMB lensing and its effects have been measured by various experiments (Hanson et al. 2013; van Engelen et al. 2012; Keisler et al. 2015; Ade et al. 2014f, g, e; van Engelen et al. 2015; Das et al. 2011; Ade et al. 2014d, 2016c). For example, Planck-2015 measured the CMB lensing potential to an overwhelming 40-$\sigma$ confidence level (Ade et al. 2016c).

These deflections and the resulting observed lensed CMB are sensitive to the distribution and growth rate of large-scale structures and their associated gravitational potential. Modification to the gravitational potential due to deviations from general relativity are thus reflected on the CMB Lensing and can be used to constrain MG parameters and models.

CMB lensing can be understood as a remapping of CMB temperature (or polarization) as follows. The lensed CMB temperature, noted as $\tilde{T}({\hat{\mathbf {n}}})$ in a direction ${\hat{\mathbf {n}}}$, is given by the unlensed temperature, $T({\hat{\mathbf {n}}}') = T({\hat{\mathbf {n}}}+ {\varvec{\alpha }})$ in the deflected direction ${\hat{\mathbf {n}}}'={\hat{\mathbf {n}}}+ {\varvec{\alpha }}$. ${\varvec{\alpha }}$ is the deflection angle that is expressed at lowest order as ${\varvec{\alpha }}= \nabla \psi _{_{\mathrm{L}}}$ where $\psi _{_{\mathrm{L}}}$ is the lensing potential, see e.g., Lewis and Challinor (2006). The latter is the result of an integration along the line of sight of the gravitational potential from the surface of last scattering all the way to us as an observer, that is

82 $$\begin{aligned} \psi _{_{\mathrm{L}}}({\hat{\mathbf {n}}}) \equiv -2 \int _0^{\chi _*} {\text {d}}\chi \, \frac{f_K(\chi _*-\chi )}{f_K(\chi _*)f_K(\chi )} \varPsi _{\mathrm{w}}(\chi {\hat{\mathbf {n}}}; \tau _0 -\chi ), \end{aligned}$$

where $\chi _*$ is the conformal distance to the surface of last scattering; $\tau _0 -\chi$ is the conformal time at which the photon was at position $\chi {\hat{\mathbf {n}}}$; $\varPsi _{\mathrm{w}}(\chi {\hat{\mathbf {n}}}; \tau )\equiv (\varPsi +\varPhi )/2$ is the Weyl gravitational potential at conformal distance $\chi$, in direction ${\hat{\mathbf {n}}}$, and at conformal time $\tau$;

Following a similar procedure as in Sect. 4.2, the power spectrum of the CMB lensing potential, for a spatially flat cosmology and in the Limber approximation (Limber 1953) is given as (see, e.g., Lewis and Challinor 2006)

83 $$\begin{aligned} C_l^{\psi _{_{\mathrm{L}}}\psi _{_{\mathrm{L}}}} = \frac{8\pi ^2}{l^3} \int _0^{\chi _*} \chi {\text {d}}\chi \, \mathcal {P}_\varPsi (l/\chi ;\tau _0-\chi ) \left( \frac{\chi _*-\chi }{\chi _*\chi }\right) ^2. \end{aligned}$$

The lensing potential power spectrum probes the matter power spectrum and its evolution and is thus sensitive to its amplitude, growth and how modification to GR affects these quantities. For example, it is very sensitive to modification to the second perturbed Eq. (30). For example, we reproduce Fig. 2 from Barreira et al. (2014a) (Fig. 4 here) where the middle panel shows how CMB lensing power spectra for Galileon MG models (see Sect. 7.3.1) versus $\varLambda$CDM model plus massive neutrinos.

It is worth pointing out Hojjati and Linder (2016) where the authors showed that CMB Lensing will be particularly useful in constraining modified gravity models, massive neutrino models, or other new physical models that are scale dependent. Such signatures will show up in the CMB lensing power spectrum and provide an additional means to constrain MG models and other models beyond *w*CDM. They show that the shapes of the deviations of the CMB lensing power spectra from that of a $\varLambda$CDM model are fairly distinct between the various scale-dependent physical origins. They highlight the role of arcminute resolution polarization experiments such as ACTpol, POLARBEAR/Simons Array, and SPT-3G, as well as the next generation CMB-S4 will be able to distinguish between these models.

A number of analyses of CMB Lensing have provided already useful constraints on various cosmological parameters, see for example Hanson et al. (2013), van Engelen et al. (2012), Keisler et al. (2015), Ade et al. (2014f, 2014g, 2014e), van Engelen et al. (2015), Das et al. (2011) and Ade et al. (2014d, 2016c). We will provide in Sect. 6 further below, various constraints on deviations from GR and MG models based on CMB Lensing.

## Formalisms and approaches to testing GR at cosmological scales

Modifications to GR at cosmological scales have been often proposed at the level of the action and its Lagrangian or at the level of the perturbed Einstein’s equations. Accordingly, formalisms for deviations from GR in this context have been developed at these two levels as we discuss in the following sub-sections.

### Effective field theory (EFT) approach to dark energy and modified gravity

The EFT approach to dark energy and modified gravity is often referred to as a “Unified” approach to dark energy since it includes in its action a broad spectrum of single field scalar–tensor dark energy and modified gravity models. It was applied first to inflation models using a Lagrangian derived from an EFT expansion (Cheung et al. 2008) and then to dark energy by for example Gubitosi et al. (2013), Bloomfield et al. (2013), Gleyzes et al. (2013) and Creminelli et al. (2009).

The approach is based on constructing a Lagrangian that includes the scalar terms for a perturbed FLRW metric assuming a single field dark energy models with operators up to a given dimension and those that are invariant under spatial diffeomorphisms. This EFT formulation is also done in the unitary gauge where the foliations of constant time coincide with the hypersurfaces of uniform scalar field. This gauge allows one to write the action only in terms of the metric and its derivatives with no scalar field perturbations appearing there, however it brings limitation of a background-dependent EFT approach compared with the covariant EFT approach of, e.g., Weinberg (2008) and Bloomfield and Flanagan (2012). The action satisfying the above restrictions, that is up to quadratic order in the perturbations, and contains only operators that lead to at most second-order equations of motion, takes the following form in the Jordan frame (Gubitosi et al. 2013; Bloomfield et al. 2013; Gleyzes et al. 2013; Creminelli et al. 2009):

84 $$\begin{aligned} S= & {} \int d^4x \sqrt{-g} \bigg \{ \frac{m_0^2}{2} \varOmega (t) R+ \varLambda (t) - c(t) \delta g^{00} \nonumber \\&+\, \frac{M_2^4 (t)}{2} (\delta g^{00})^2 - \frac{\bar{M}_1^3 (t)}{2} \delta g^{00} \delta K^\mu _\mu - \frac{\bar{M}_2^2 (t)}{2} (\delta K^\mu _\mu )^2 \nonumber \\&-\, \frac{\bar{M}_3^2 (t)}{2} \delta K^i_j \delta K^j_i + \frac{\hat{M}^2 (t)}{2} \delta g^{00} \delta R^{(3)} \nonumber \\&+ \,m_2^2(t)\left( g^{\mu \nu }+n^{\mu } n^{\nu }\right) \partial _{\mu }(g^{00})\partial _{\nu }(g^{00}) \bigg \}\nonumber \\&+\, S_{m} [g_{\mu \nu }, \chi _i] \ , \end{aligned}$$

where $m_0^{-2} = 8\pi G$ is the reduced Planck mass; $\delta g^{00}$ is the perturbation of the time-time component of the inverse metric; $\delta {K}{^\mu _\nu }$, $\delta K$ are the perturbation of the extrinsic curvature and its trace; $\delta R^{(3)}$ is the perturbation of the three dimensional spatial Ricci scalar of constant-time hypersurfaces; $n^{\mu }$ is the 4-vector normal to the constant-time hypersurfaces; and $S_m$ is the action for all matter fields $\chi _i$ minimally coupled to the metric $g_{\mu \nu }$.

The coefficients $M^i_j(t)$ are functions of time and have dimensions of mass. The functions *c*(*t*) and $\varLambda (t)$ (not to be confused with the cosmological constant) can be re-expressed in terms of the function $\varOmega (t)$ and background functions such as the Hubble and density parameters by using the FLRW background evolution equations. Thus, the theories covered by action (84) can be specified by the following 7 functions of time:

85 $$\begin{aligned} \left\{ \varOmega ,\bar{M}_1^3,\bar{M}_2^4,\bar{M}_3^2,M_2^4,\hat{M}^2, m_2^2\right\} \end{aligned}$$

plus one function describing the background evolution such as the Hubble function.

It is worth mentioning that the EFT approach covers both the background evolution and the linear perturbations of the metric so it provides equations and parameterization that can be compared to the background evolution as well as the growth of large-scale structure observations. However, in order to compare effectively the whole set to observations, one needs to do further useful parameterizations of the functions (85). For example, for Horndeski models (Horndeski 1974), these functions are mapped to the so-called $\alpha _x$ parameterization (Bellini and Sawicki 2014) which is then connected to the physical aspect of the theory as we discuss in Sect. 7.3.1 further below. See also another informative reconstruction of Horndeski from EFT of dark energy in Kennedy et al. (2017).

**Table 1:** The EFT formalism covers a number of different theories of dark energy and modified gravity

Model parameter	$\varOmega$	$\varLambda$	*c*	$M_2^4$	$\bar{M}_1^3$	$\bar{M}_2^2$	$\bar{M}_3^2$	$\hat{M}^2$	$m_2^2$
Corresponding operator	R		$\delta g^{00}$	$(\delta g^{00})^2$	$\delta g^{00} \delta {K}{^\mu _\mu }$	$(\delta {K}{^\mu _\mu })^2$	$\delta {K}{^\mu _\nu } {K}{^\nu _\mu }$	$\delta g^{00} \delta R^{(3)}$	$\frac{\tilde{g}^{ij}}{a^2} \partial _i g^{00} \partial _j g^{00}$
$\varLambda$CDM	1	$\checkmark$	0	–	–	–	–	–	–
Quintessence	1/$\checkmark$	$\checkmark$	$\checkmark$	–	–	–	–	–	–
*f*(*R*)	$\checkmark$	$\checkmark$	0	–	–	–	–	–	–
*k*-essence	1/$\checkmark$	$\checkmark$	$\checkmark$	$\checkmark$	–	–	–	–	–
Galileon (Kinetic Braiding)	1/$\checkmark$	$\checkmark$	$\checkmark$	$\checkmark$	$\checkmark$	–	–	–	–
DGP	$\checkmark$	$\checkmark$ $\dagger$	$\checkmark$ $\dagger$	$\checkmark$ $\dagger$	$\checkmark$	–	–	–	–
Ghost Condensate	1/$\checkmark$	$\checkmark$	0	–	–	$\checkmark$	$\checkmark$	–	–
Horndeski	$\checkmark$	$\checkmark$	$\checkmark$	$\checkmark$	$\checkmark$	$\checkmark$ $\dagger$	$\checkmark$ $\dagger$	$\checkmark$ $\dagger$	–
Hořava–Lifshitz	1	$\checkmark$	0	–	–	$\checkmark$	–	–	$\checkmark$

This table provides the list of coefficient functions (85), related Lagrangian operators entering the theory from the action (84) and some selected known cases in the literature (this table was adapted by Linder et al. 2016 from Bloomfield et al. 2013). Horndeski theories are also known as generalized Galileons. Reproduced with permission from Table I of Linder et al. (2016), copyright by IOP

$\checkmark$, Operator is necessary

–, Operator is not included

1, 0, Coefficient is unity or vanishes exactly

1/$\checkmark$, Minimally and non-minimally coupled versions of this model exist in the literature

$\dagger$, Coefficients marked with a dagger are linearly related to other coefficients in that model by numerical coefficients

The EFT action (84) is general enough to include broad classes of dark energy and modified gravity such as the Horndeski 1974 or generalized Galileons (Deffayet et al. 2009a), beyond Horndeski models (Zumalacarregui and García-Bellido 2014; Gleyzes et al. 2015a, b), Hořava–Lifshitz gravity in its low energy limit (Hořava 2009b; Kase and Tsujikawa 2014), ghost condensate models (Hamed et al. 2004), and DGP braneworld models (Dvali et al. 2000). We reproduce Table I from Linder et al. (2016) (Table 1 here) that shows the list of the function parameters (85), the corresponding terms in the Lagrangian operators of the action (84), and some gravity theories with the terms they involve from the EFT Lagrangian.

While the EFT approach can be praised for its clear theoretical motivation and systematic nature, it has the disadvantage of requiring the use of a large number of parameters and functions. This number overwhelms the limited constraining power of current cosmological data. Nevertheless, some of the coefficients can be set to zero or can be shown to be interrelated in the case of some known dark energy or modified gravity models so one can reduce the number of parameters to a practical one. This of course affects the primary motivation of the EFT approach in providing a systematics method but the hope is that as more orthogonal and precise data sets become available in the future this method will reach its aimed goals. Also, the effectiveness of the EFT approach was questioned in Linder et al. (2016) stating that the EFT functions used do not have a simple time dependence that can be fit to observations for different cosmic eras, but as they state, one can nevertheless gain some general characteristics of such dependencies for early and late time limits of cosmic evolution.

Most recently, Lagos et al. (2016, 2018) followed on a previous effort of the Parameterized-Post-Friedmann formalism of Baker et al. (2013) in order to extend the EFT formalism to cover beyond scalar–tensor theories. The general approach they proposed recovers the standard $\alpha$-parameterization of Bellini and Sawicki (2014) for Horndeski models (see Sect. 7.3.1) but also applies to beyond-Horndeski models, vector–tensor theories, and tensor–tensor theories. In each of the more complicated theories, the formalism considers a few additional $\alpha _x$-parameters for up to 12 parameters in the most general case. We refer the reader to their papers for more information.

Due to its broad application, the EFT approach has been implemented in several Einstein–Boltzmann solvers and Markov-Chain–Monte-Carlo codes to analyze CMB and other datasets, see for example Hu et al. (2014b), Bellini et al. (2018) and references therein, as well as our discussion in Sect. 11.

### Modified growth parameters

We discussed in Sect. 3.2 how the growth of large scale structure can be described by the two equations (29) and (30) derived from linear perturbations of the Einstein’s Field Equations. Now, the effect of deviations from GR on the growth of large structure can be encapsulated in two parameters added to these equations. These are then often called the modified growth or Modified gravity (MG) equations. Usually, one of the MG parameters modifies the coupling between the gravitational potential and the energy-density source while the other parameter quantifies the difference between the two gravitational potentials. There are various related parameterizations notations and we review some of the most commonly used ones in the literature.

One pair of such parameters is given by *Q*(*k*, *a*) and *R*(*k*, *a*) as follows, see e.g., Caldwell et al. (2007), Amendola et al. (2008) and Bean and Tangmatitham (2010):

86 $$\begin{aligned} k^2{\varPhi }= & {} -4\pi G a^2\sum _i {\bar{\rho }}_i \delta _i \, Q(k,a) \end{aligned}$$

87 $$\begin{aligned} k^2(\varPsi -R(k,a)\,\varPhi )= & {} -12 \pi G a^2\sum _i {\bar{\rho }}_i(1+w_i)\sigma _i \, Q(k,a), \end{aligned}$$

where each matter specie is denoted by the index *i*, ${\bar{\rho }}_i$ is the corresponding mass-energy density, $\delta _i$ is the rest-frame overdensity, and $\sigma _i$ is the shear stress. *Q*(*k*, *a*) and *R*(*k*, *a*) are scale and time dependent and both take the value of unity in GR.

The parameter *Q*(*k*, *a*) represents a modification to the “Poisson equation” (29) (see comments in Dossett et al. 2011b), while the parameter *R*(*k*, *a*) quantifies the inequality between the two potentials referred to as the gravitational slip (Caldwell et al. 2007) (at late times, when anisotropic stress is negligible, Eq. (87) gives $R=\varPsi \!/\!\varPhi$). Caldwell et al. (2007) noted the slip parameter as $\varPsi = (1 + \varpi )\varPhi$ based on a cosmological extension to the PPN formalism, see e.g., Will (2014).

In order to avoid a strong degeneracy between the parameters *Q*(*k*, *a*) and *R*(*k*, *a*), Eqs. (86) and (87) can be combined to introduce another MG parameter as follows (see, e.g., Amendola et al. 2008):

88 $$\begin{aligned} k^2(\varPsi +\varPhi ) = -8\pi G a^2\sum _i {\bar{\rho }}_i \delta _i \,\varSigma (k,a)\, -12 \pi G a^2\sum _i {\bar{\rho }}_i (1+w_i)\sigma _i \, Q(k,a), \end{aligned}$$

where

89 $$\begin{aligned} \varSigma (k,a) \equiv \frac{Q(k,a)[1+R(k,a)]}{2}. \end{aligned}$$

The parameter $\varSigma (k,a)$ enters the equation for the Weyl potential defined earlier (i.e., $\varPsi _{\mathrm{w}}\equiv (\varPsi +\varPhi )/2$) which affects the propagation of light. The parameter is thus directly constrained by some observations such as weak gravitational lensing. Just like the parameters *Q* and *R*, $\varSigma$ takes unity in general relativity.

A second pair of MG parameters often used in the literature is where a modification to Eq. (29) is done indirectly by defining a modified field equation containing the parameter $\mu (k,a)$ plus a gravitational slip parameter, $\eta (k,a)$ (Zhao et al. 2009, 2010; Hojjati et al. 2011; Caldwell et al. 2007; Amendola et al. 2008). The modified growth equations then read:

90 $$\begin{aligned} k^2\varPsi= & {} -4\pi G a^2\sum _i {\bar{\rho }}_i \delta _i \, \mu (k,a). \end{aligned}$$

91 $$\begin{aligned} \frac{\varPhi }{\varPsi }= & {} \eta (k,a). \end{aligned}$$

The generalization of these two equations for non-zero shear can be found in, for example, equations (13) and (14) of Hojjati et al. (2011). Again, $\varSigma (k,a)$ is defined from their combination as

92 $$\begin{aligned} \varSigma (k,a)\equiv \frac{\mu (k,a)[1+\eta (k,a)]}{2} \end{aligned}$$

Similarly, these parameters have a scale and time dependencies and take the value of unity for GR.

A third notation is one that associates MG parameters with effective gravitational constants in the growth equations (see, e.g., Tsujikawa 2007; Song and Koyama 2009; Linder 2017) so that the modified Poisson equations take the form

93 $$\begin{aligned} k^2\varPsi= & {} -4\pi G_{\mathrm{eff}}^{\varPsi } a^2\sum _i {\bar{\rho }}_i \delta _i \end{aligned}$$

94 $$\begin{aligned} k^2(\varPsi +\varPhi )= & {} -8\pi G_{\mathrm{eff}}^{\varPsi +\varPhi } a^2\sum _i {\bar{\rho }}_i \delta _i. \end{aligned}$$

Equation (93) governs the coupling between the gravitational potential for non-relativistic particles to the source density fluctuation while Eq. (94) governs the coupling of the gravitational potential for relativistic particles to the source density fluctuation and affects geodesics of relativistic particles such as light propagation and gravitational lensing. Often $G_{\mathrm{eff}}^{\varPsi }$ is dubbed as $G_\mathrm{matter}$ and $G_{\mathrm{eff}}^{\varPsi +\varPhi }$ as $G_\mathrm{light}$.

It is worth concluding this sub-section by providing the relationships between the different parametrizations above during matter domination and assuming zero anisotropic stress

95 $$\begin{aligned} \mu =Q R = \frac{G_{\mathrm{eff}}^{\varPsi }}{G}=\frac{G_\mathrm{matter}}{G},&\quad \eta = \frac{1}{R} \end{aligned}$$

96 $$\begin{aligned} \varSigma = \frac{Q(1+R)}{2} =\frac{G_{\mathrm{eff}}^{\varPsi +\varPhi }}{G}= \frac{G_\mathrm{light}}{G}&\quad \mu \eta = Q . \end{aligned}$$

A more extended discussion of the relationship between MG parameters can be found in Daniel et al. (2010).

Finally, on super-horizon scales $k\ll a H$ and for adiabatic perturbations, there are further useful constraints from coordinates invariance that apply to GR and also MG theories (Bertschinger 2006). These provide a consistency relation between the two gravitational potential which reduces the two independent functions (MG parameters) above to only one parameter. The consistency relation plus the MG parameter $\eta (a)$ can be used to characterize deviation from GR at super-horizon scales. In other words, at these long wavelength, $\eta (a)$ is the only important degree of freedom for MG gravity (Bertschinger 2006; Bertschinger and Zukin 2008; Hu and Sawicki 2007b).

### Evolution of MG parameters in time and scale

Departures from general relativity can evolve in time and/or scale and this has been included in parametrizations and studies. Mainly two approaches have been used in doing so. The first method employs generic functional forms, while the second uses binning in redshift and scale. A third method combines the two previous ones into a hybrid method.

- **Functional forms for time and scale evolution**: For example, Bean and Tangmatitham (2010) used:
  97 $$\begin{aligned} X(k,a) = \left[ X_0 e^{-k/k_c}+X_\infty (1-e^{-k/k_c})-1\right] a^s +1, \end{aligned}$$
  where *X* denotes, for example, *Q* or *R*. $Q_0$ and $R_0$ are the present-day asymptotic superhorizon values while $Q_\infty$ and $R_\infty$ are the present-day asymptotic subhorizon values of *Q*(*k*, *a*) and *R*(*k*, *a*). $k_c$ is a comoving transition scale. The time evolution is given by $a^s$. It was noted though in, for example Zhao et al. (2010), Song et al. (2011) and Dossett et al. (2011a), that such a functional exponential form causes a too strong dependence of MG parameters on the exponent *s* and can exacerbate tensions between GR and data (Dossett et al. 2011a). It was found in these papers that a binning method in redshift avoids this problems. The model parameters that can be used to detect deviations from GR are now: $Q_0$, $R_0$, $Q_\infty$, $R_\infty$, $k_c$, and *s*. The parameters *s* and $k_c$ take the values $s=0$ and $k_c=\infty$ in GR and the other parameters reduce to unity. The constraints on $\varSigma (k,a)$ can then be derived using Eq. (89).
  In a similar way, the parameters, $\mu$ and $\eta$ have also been allowed to evolve, for example, in redshift. In Dossett et al. (2011a), the two parameters have a redshift dependence transitioning to constant values below some redshift, $z_s$, and then take the GR value of unity following a hyperbolic tangent function with a transition width, $\delta z$:
  98 $$\begin{aligned} \mu (z)= & {} \frac{1-\mu _{0}}{2}\Big (1 + \tanh {\frac{z-z_s}{\delta z}}\Big ) + \mu _{0}, \end{aligned}$$
  99 $$\begin{aligned} \eta (z)= & {} \frac{1-\eta _0}{2}\Big (1 + \tanh {\frac{z-z_s}{\delta z}}\Big ) + \eta _{0}. \end{aligned}$$
  The parameter $\varSigma (z)$ then follows from Eq. (96) above.
  Functional forms for MG parameters have been discussed to be less flexible than binning or hybrid methods in Dossett et al. (2011a) and Daniel et al. (2010).
- **Time and scale binning method of MG parameters**: An example of binning MG parameters in time (redshift) and scale is provided in Dossett et al. (2015). Two scale bins are defined as $k\le 0.01\,h$ Mpc$^{-1}$ and $k>0.01\,h$ Mpc$^{-1}$. These are crossed with two other bins in redshift defined by $0<z\le 1$ and $1<z\le 2$. In order to assure for the transition between the bins to be continuous and for numerical implementation stability, the following transition functions have been used:
  100 $$\begin{aligned}&X(k,a) =\frac{1}{2}\big (1 + X_{z_1}(k)\big )+\frac{1}{2}\big (X_{z_2}(k) - X_{z_1}(k)\big )\nonumber \\&\tanh {\frac{z-1}{0.05}}+\frac{1}{2}\big (1 - X_{z_2}(k)\big )\tanh {\frac{z-2}{0.05}}, \end{aligned}$$
  with
  101 $$\begin{aligned} X_{z_1}(k)= & {} \frac{1}{2}\big (X_2+X_1\big )+\frac{1}{2}\big (X_2-X_1\big )\tanh {\frac{k-0.01}{0.001}}, \nonumber \\ X_{z_2}(k)= & {} \frac{1}{2}\big (X_4+X_3\big )+\frac{1}{2}\big (X_4-X_3\big )\tanh {\frac{k-0.01}{0.001}}, \end{aligned}$$
  where *X* takes the values *Q* or $\varSigma$ so in this parameterization a total of eight MG parameters are varied, $\varSigma _i$ and $Q_i$, $i=1,2,3,4$. Again, all these parameters take a value of unity in GR.
- **Hybrid methods for MG parameters**: Finally, the implementation of MG parameters can be optimized to take advantage of each of the two methods above. For that, hybrid methods have been employed in order to keep a functional form for the scale dependence while using bins of redshift for the time evolution as follows (Dossett et al. 2015). The redshift bins are similarly given by Eq. (100) above while the scale dependence is given the form:
  102 $$\begin{aligned} X_{z_1}(k)= & {} X_1 e^{-\frac{k}{0.01}}+X_2\left( 1-e^{-\frac{k}{0.01}}\right) , \nonumber \\ X_{z_2}(k)= & {} X_3 e^{-\frac{k}{0.01}}+X_4\left( 1-e^{-\frac{k}{0.01}}\right) . \end{aligned}$$
  This gives again eight MG parameters, $\varSigma _i$ and $Q_i$, $i=1,2,3,4$ to be constrained by observations.
- **f(R) guided time and scale parametrization**: Guided by *f*(*R*) formalism (see Sect. 7.4.1), Bertschinger and Zukin (2008) suggested a phenomenological time and scale parametrization as follows:
  103 $$\begin{aligned} \mu (a,k)= & {} \frac{1+\alpha _1k^2a^s}{1+\alpha _2k^2a^s} \end{aligned}$$
  104 $$\begin{aligned} \eta (a,k)= & {} \frac{1+\beta _1k^2a^s}{1+\beta _2k^2a^s}, \end{aligned}$$
  To construct such a parameterization, the authors required GR to hold at early times, so that $s>0$. They also noted that this parametrization describes *f*(*R*) theories with $|f_R|\ll 1$ for $\alpha _1=\frac{4}{3}\alpha _2=2\beta _1=\beta _2=4f_{RR}/a^{2+s}$. $(\alpha _1,\alpha _2,\beta _1,\beta _2)$ are arbitrary constants with $\alpha _2$ and $\beta _2$ positive so $\mu$ and $\gamma$ remains finite for all k. $\alpha _1$ must be positive as well to assure that $\mu$ is positive and gravity is attractive.
- **Using rational functions of** $k^2$ **and five functions of time**: Silvestri et al. (2013) showed that for local theories of gravity with one scalar degree of freedom with up to second order equation of motion and in the quasi-static approximation, the two MG parameter $\mu (k,a)$ and $\eta (k,a)$ can be written as rational functions of $k^2$ with at most 5 functions of time in all generality as follows:
  105 $$\begin{aligned} \eta (a,k)= & {} \frac{p_1(a)+p_2(a)k^2}{1+p_3(a)k^2}, \end{aligned}$$
  106 $$\begin{aligned} \mu (a,k)= & {} \frac{1+p_3(a)k^2}{p_4(a)+p_5(a)k^2}. \end{aligned}$$
  They note that even if this parametrization has been derived for the quasi-linear limit, it is expected to work fine at the near- and super-horizon scales since $\eta (a,k \rightarrow 0)=p_1(a)\ne 1$. They also note that $\mu (a,k\rightarrow 0)=1/p_4(a)\ne 1$ should be of no-consequences on observables and that super-horizon perturbations will have an evolution consistent with the background expansion (Silvestri et al. 2013). See also discussions for this type of rational functions in De Felice et al. (2011) and for higher order in the wavenumber in Vardanyan and Amendola (2015).

**Table 2:** The layout of the binned parametrizations

	Redshift bins	
Scale bins	$0.0<z\le 1,\,1.5$	$1,\,1.5 <z \le 2,\,3$
$0.0 < k \le k_x$	$\mu _{1},\,\varSigma _{1}$	$\mu _{2},\,\varSigma _{2}$
$k_x< k< \infty$	$\mu _{3},\,\varSigma _{3}$	$\mu _{4},\,\varSigma _{4}$

Specifically, for the first two binned methods this involves using $\{\mu _{1},\,\varSigma _{1}\}$ for the $0<z\le 1$ and $0.0<k\le k_x$ bin, $\{\mu _{2},\,\varSigma _{2}\}$ for the $1<z\le 2$ and $0.0<k\le k_x$ bin, $\{\mu _{3},\,\varSigma _{3}\}$ for the $0<z\le 1$ and $k_x<k< \infty$ bin, and $\{\mu _{4},\,\varSigma _{4}\}$ for the $1<z\le 2$ and $k_x<k< \infty$ bin, and the third binned method uses $\{\mu _{1},\,\varSigma _{1}\}$ for the $0<z\le 1.5$ and $0.0<k\le k_x$ bin, $\{\mu _{2},\,\varSigma _{2}\}$ for the $1.5<z\le 3$ and $0.0<k\le k_x$ bin, $\{\mu _{3},\,\varSigma _{3}\}$ for the $0<z\le 1.5$ and $k_x<k< \infty$ bin, and $\{\mu _{4},\,\varSigma _{4}\}$ for the $1.5<z\le 3$ and $k_x<k< \infty$ bin. Table reproduced with permission from Dossett et al. (2015), copyright by APS

### The growth index parameter $\gamma$

Another approach to use the linear growth of structure to constrain deviations from general relativity is by defining the growth index parameter as follows. In some pioneering early work for a matter-dominated universe, the growth function *f* was shown to be well-approximated by the following ansatz (Peebles 1980; Fry 1985; Lightman and Schechter 1990):

107 $$\begin{aligned} f \equiv \varOmega _m^\gamma \end{aligned}$$

where $\gamma$ is the growth index parameter. Peebles (1980) introduced the approximation $f(z=0)\approx \varOmega _0^{0.6}$ for matter dominated models. After that, Fry (1985) and Lightman and Schechter (1990) proposed more accurate approximations for such a model, i.e., $f(z=0) \approx \varOmega _0^{4/7}$.

Later on, the work was extended to dark energy models (GR-wCDM) with a slowly varying equation of state by Wang and Steinhardt (1998) deriving the following expression:

108 $$\begin{aligned} \gamma (\varOmega _m,w)=\frac{3(1-w)}{5-6w}+\frac{3}{125} \frac{(1-w)(1-3w/3}{(1-6w/5)^2(1-12w/5)}(1-\varOmega _m) \end{aligned}$$

with an asymptotic early value of $\gamma _\infty ^{w\mathrm{CDM}}=3(1-w)/(5-6w)$ reducing to the well known $\varLambda$CDM model value of $\gamma ^{\mathrm{LCDM}}=\frac{6}{11}=0.545$.

Linder (2005) extended this growth index approach to modified gravity theories and pointed out that it can be used as a discriminator between quintessence dark energy models and modified gravity models. For example, for the DGP model (see Sect. 7.5.2) has a growth index parameter of $\gamma ^{\mathrm{DGP}}=\frac{11}{16}=0.68$ (Lue et al. 2004; Linder 2005) and thus is clearly distinct from the value of the $\varLambda$CDM model. Indeed, despite some dispersion of $\gamma ^{w\mathrm{CDM}}$ for various values of *w* and also some dispersion of $\gamma ^{\mathrm{DGP}}$ for various values of $\varOmega _{m}(a)$, such fluctuations do not overlap and $\gamma$ remains a good discriminator for gravity theories, see e.g., Linder and Cahn (2007), Gong (2008), Polarski and Gannouji (2008) and Ishak and Dossett (2009) for spatially flat models and Gong et al. (2009) and Mortonson et al. (2009) for curved models.

Moreover, the growth index can be allowed to vary in redshift and provides more stringent constraints on gravity theories (Polarski and Gannouji 2008; Ishak and Dossett 2009). For example, Polarski and Gannouji (2008) proposed a redshift dependent parameterization of the form

109 $$\begin{aligned} \gamma (z)=\gamma _0 + \gamma '\,z, \end{aligned}$$

where $\gamma '\equiv \frac{d\gamma }{dz}(z=0)$. The study showed the usefulness of a variable growth index to distinguish between dark energy models and modified gravity models (Polarski and Gannouji 2008). Ishak and Dossett (2009) and Wu et al. (2009) proposed a redshift dependent parameterization that covers a wide range of redshift highlighting that the sign of the slope $\gamma (z)$ can provide further discrimination between gravity theories.

### The $E_G$-parameter test

Zhang et al. (2007) proposed a measure they called $E_G$ to test deviations from GR’s gravitational potentials in a way that is insensitive to the galaxy bias. The idea is to use a ratio of the galaxy–galaxy lensing angular cross power spectrum over the velocity–galaxy cross power spectrum. We use here a mixture of notation from Zhang et al. (2007) and Leonard et al. (2015) to describe this quantity. The corresponding estimator was defined in the original paper (Zhang et al. 2007) as

110 $$\begin{aligned} \hat{E}_{G}(\ell , \delta \ell )=\frac{C_{\kappa g}(\ell , \delta \ell )}{3H_{0}^2a^{-1}\sum \limits _{\alpha } j_{\alpha }(\ell , \delta \ell )P^{\alpha }_{vg}}, \end{aligned}$$

where $C_{\kappa g}(\ell , \delta \ell )$ is the galaxy–galaxy lensing cross-power spectrum in bins of $\delta \ell$; $P^{\alpha }_{vg}$ is the galaxy–velocity cross-power spectrum between $k_{\alpha }$ and $k_{\alpha +1}$; and $f_{\alpha }(\ell , \delta \ell )$ is a weighting function defined accordingly. The corresponding expectation value is then given by:

111 $$\begin{aligned} E_{G}(\ell )=\left[ \frac{\nabla ^2(\varPsi +\varPhi )}{3H_0^2a^{-1}f\delta _M}\right] _{k=\ell /\bar{\chi },\bar{z}} \end{aligned}$$

where *f* is the linear growth rate of structure, $\delta _M$ is the matter overdensity field, $\bar{\chi }$ is the comoving distance corresponding to redshift $\bar{z}$. For GR $\varLambda$CDM, $E_G$ is independent of length scale and is given by Zhang et al. (2007)

112 $$\begin{aligned} E_{G}=\frac{\varOmega _M(z=0)}{f(z)}. \end{aligned}$$

The scale independence holds for *w*CDM models with large-sound speed and negligible anisotropic stress like Quintessence. It also holds for some modified gravity models like DGP (see Sect. 7.5.2) but not for other MG models. The scale dependence of $E_G$ can be used as a further discriminator between MG models (Zhang et al. 2007).

It is also worth providing a second definition of $E_G$ motivated by observations as given by Reyes et al. (2010)

113 $$\begin{aligned} E_G(R)=\frac{\varUpsilon _{gm}(R)}{\beta \varUpsilon _{gg}(R)}, \end{aligned}$$

where *R* is the transverse separation from the lens-galaxy; $\varUpsilon _{gm}(R)$ and $\varUpsilon _{gg}(R)$ are the galaxy-matter and galaxy-galaxy annular differential surface densities respectively, see e.g., Baldauf et al. (2010). By construction, these are correlation functions that do not include any contribution from length scales smaller than some cut-off $R = R_0$. This second definition in Eq. (113) provides a ratio that is practically similar to the information content of Eq. (110) and also factors out the galaxy bias. Most recently, Leonard et al. (2015) provided further insights on how theoretical uncertainties such as scale dependence of the bias, projection effects, and cut-off scale can affect measurements of $E_G$ using future high precision probes and the conclusions that can be drawn from them. We present further below in Sect. 6.3 some constraints on the $E_G$ measure from recent data.

We conclude this sub-section with some recent findings about the $E_G$ measure from Amon et al. (2017) using the deep imaging data of KiDS with overlapping spectroscopic regions from 2dFLenS, BOSS DR12 and GAMA. The authors find that changing the metric potentials by as much as 10% produces smaller differences in the $E_G$ predictions than changing the value of $\varOmega _m^0$ between the values prefered by Planck and KiDS. They conclude that for this statistic to achieve its aim, the current tensions in cosmological parameters between Planck and large scale structure must be resolved first.

### Parameterized post-Friedmann formalism

It appears that the parametrized post-Friedmann (PPF) formalisms at cosmological scales (Hu and Sawicki 2007b; Baker et al. 2013) has not yet reached the same popularity that its homologous, the parameterized post-Newtonian (PPN), has received when testing GR at solar system levels or binary systems (Will 2014). This could be attributed perhaps to the context and the level of maturity of other methods developed to deal with the specific problems for which each formalism has been introduced. There are at least two major developments in PPF formalisms (Hu and Sawicki 2007b; Baker et al. 2013) but also a number of previous developments such as in Bertschinger (2006), Caldwell et al. (2007), Amin et al. (2008), Pogosian et al. (2010) and Baker et al. (2011). It is also worth noting that the PPF work of Baker et al. (2013) was followed by some of the same authors and others in Lagos et al. (2016, 2018) where the approach was changed to an EFT one as we comment at the end of this subsection.

While inspired by PPN, PPF needs to be formulated to account for cosmological Hubble scales where the exact form of the linearized metric is unknown and the redshift dependence must be taken into account. Therefore, PPF uses rather functions of the redshift and scale and is based on the parameterization of the perturbed field equations instead of the spacetime metric (Baker et al. 2013; Amendola et al. 2013a). We provide a very brief overview below and refer the reader to the original papers (Hu and Sawicki 2007b; Baker et al. 2013).

The first one was proposed in Hu and Sawicki (2007b) where the authors discuss super-horizon, quasi-static and nonlinear regimes of modified gravity with a particular attention to the transitions between them. They construct a PPF formalism for linear perturbations in MG models that joins the super-horizon regime and the sub-horizon quasi-static regime. They propose PPF functions that make the bridge between these two regimes at a scale parameterized by the Hubble length. They defined three functions and one parameter as follows:

- The metric ratio
  114 $$\begin{aligned} g(\ln a,k_H) \equiv {\varPhi -\varPsi \over {\varPhi } +\varPsi }, \end{aligned}$$
  where $k_H \equiv k/aH$ is the wavenumber in units of the Hubble parameter. Note that in terms of the post-Newtonian parameter $\eta = {\varPhi }\!/\varPsi$, $g= (\eta -1)/(\eta +1)$.
  The expansion history H and the metric ratio g define completely super-horizon scalar metric fluctuations for adiabatic perturbations.
- The function $f_\zeta (\ln a)$ expressing the super-horizon relationship between the metric and density, see Eqs. (16)–(19) in Hu and Sawicki (2007b). As noted there, the exact form of $f_\zeta (\ln a)$ is rarely important for observable quantities. That is the case, for example, for the galaxy redshift surveys and gravitational lensing. Only observable quantities that depend on the comoving density scales beyond the quasi-static regime are affected by $f_\zeta (\ln a)$.
- The function $f_G(\ln a)$ that parameterizes a possible time-dependent modification of the Newton constant in the quasi-static regime. It is defined from the Poisson equation
  115 $$\begin{aligned} k^2 \varPsi _{\mathrm{w}} = {4\pi G \over 1+f_G} a^2 {\bar{\rho }}_m \delta _{m}, \end{aligned}$$
  where $\varPsi _{\mathrm{w}}$ is the Weyl potential defined earlier.
- The parameter $c_\varGamma$ that characterizes the relationship between the transition scale and the Hubble scale. As shown in Hu and Sawicki (2007b), the interpolation between the super-horizon regime and the quasi-static regime is given by
  116 $$\begin{aligned} \left( 1+ c_\varGamma ^2 k_H^{2}\right) \left[ \varGamma ' +\varGamma + c_\varGamma ^2 k_H^{2 }\left( \varGamma -f_G\varPsi _{\mathrm{w}}\right) \right] = S, \end{aligned}$$
  where $\varGamma$ is added to the modified Poisson equation (115) in order to match the super-horizon scale behavior
  117 $$\begin{aligned} k^2 [\varPsi _{\mathrm{w}} + \varGamma ] = 4\pi G a^2 \rho _m\varDelta _m, \end{aligned}$$
  and where *S* is the source for the equation of motion of $\varGamma$ (Hu and Sawicki 2007b).

For MG models affecting cosmic evolution after matter radiation equality, these 3 functions governing the relations for the metric, the density and the velocity, plus the usual transfer functions specify fully the linear observables of the model.

They provided two examples, one for a *f*(*R*) theory model (see Sect. 7.4.1) and another for a DGP theory model (see Sect. 7.5.2). We reproduce their example for the former here. The square of the Compton length (inverse mass) in units of the Hubble length for *f*(*R*) is proportional to

118 $$\begin{aligned} B\equiv & {} {f_{RR} \over 1+f_R} {R'}{H \over H'}, \end{aligned}$$

where $^{\prime }=d/d\ln a$ and $f_{RR}= d^2 f/dR^2$. The metric ratio parameter $g \rightarrow -1/3$ below the Compton length scale. They determine that the PPF metric ratio as $k_H\rightarrow 0$ is given by

119 $$\begin{aligned} g(\ln a, k_H=0)= g_{\mathrm{SH}}(\ln a) = {\varPhi -\varPsi \over {\varPhi } + \varPsi }, \end{aligned}$$

and

120 $$\begin{aligned} f_\zeta = c_\zeta g \end{aligned}$$

with $c_\zeta \approx -1/3$. They take for the transition to the quasi-static regime the interpolating function

121 $$\begin{aligned} g(\ln a,k)&= { g_{\mathrm{SH}} + g_{\mathrm{QS}}(c_{g}k_H)^{n_{g}} \over 1+ (c_{g}k_H)^{n_{g}}}, \end{aligned}$$

where $g_{\mathrm{QS}}=-1/3$. They find that $c_g=0.71 B^{1/2}$ and $n_g=2$ where they used $\varOmega _m=0.24$ and $w_{\mathrm{eff}}=-1$.

Last, they find that $f_R$ is the function that rescales the effective Newton constant and the quasi-static transition happens near the horizon scale. The two statements correspond to

122 $$\begin{aligned} f_G = f_R, \qquad c_\varGamma =1. \end{aligned}$$

The second PPF formalism was proposed in Baker et al. (2013) taking into account the recent exploding development in the area of dark energy and modified gravity models. A concise summary of the formalism was also given in Amendola et al. (2013a) and we follow that presentation here. Baker et al. (2013) start with scalar perturbations of the Einstein field equations of the form

123 $$\begin{aligned} \delta G_{\mu \nu } \;=\; 8\pi G\,\delta T_{\mu \nu }+\delta U_{\mu \nu }^{\mathrm {metric}}+\delta U_{\mu \nu }^{\mathrm {d.o.f}}+\mathrm {\ gauge\ invariance\ fixing\ terms}, \end{aligned}$$

where $\delta T_{\mu \nu }$ is the perturbed stress-energy tensor of cosmic fluids. $\delta U_{\mu \nu }^{\mathrm {metric}}$ contains new terms from metric perturbations due to modified gravity that constitute terms beyond those coming from $\delta G_{\mu \nu }$ in GR. $\delta U_{\mu \nu }^{\mathrm {d.o.f.}}$ contains terms from scalar perturbations of new degrees of freedom due to modified gravity. For example, such terms can come from perturbations of the scalar field from scalar–tensor theories or scalar modes from vector or tensor fields in MG models.

Baker et al. (2013) then considered the expansion of $\delta U_{\mu \nu }^{\mathrm {metric}}$ in terms of two gauge-invariant perturbation variables. The first is simply the standard gauge-invariant Bardeen potentials, $\hat{\varPhi }$. The second is a combination of the two Bardeen potentials as follows: ${\hat{\varGamma }}=1/k (\dot{\hat{\varPhi }}+{\mathcal {H}}{\hat{\varPsi }})$. They provided then the equations further below where $\delta U_{\mu \nu }^{\mathrm {metric}}$ is expressed as a linear combination of $\hat{\varPhi }$, ${\hat{\varGamma }}$ and their derivatives keeping the gauge-invariance of the field equations. The coefficient of such terms are then part of the PPF function set. They also expressed $\delta U_{\mu \nu }^{\mathrm {d.o.f.}}$ for the new degrees of freedom in terms of gauge-invariant potentials $\{{\hat{\chi }}_i\}$ with also coefficients providing other PPF functions. They write then the expanded four components of the perturbed field equations Eq. (123), where 22 PPF parameters where used as functions of time (redshift).

The set of PPF parameters covers super-horizon and sub-horizon scales but the set simplifies significantly in the quasi-static regime reducing to what could be encapsulated in one of the pairs of parameters discussed in Sect. 5.2. It was argued in, for example Amendola et al. (2013a), that in such a regime, which is relevant to weak lensing surveys and galaxy surveys, such a minimal subset is more practical to compare with observation but Baker et al. (2013) explains that such a PPF formalism can extend to horizon scales and can serve for comparisons to large-scale CMB modes contributions to the ISW effect and lensing-ISW cross-correlations, well beyond the quasi-static approximation (Hu et al. 2013; Hu 2008).

Most recently, some of the authors of Baker et al. (2013) and others commented in Lagos et al. (2016, 2018) that the expanded four components of the perturbed field equations with PPF parameters in Baker et al. (2013) contain a lot of free functions because the parameterization is built directly at the level of the field equations. In other words, the coefficients PPF parameters are not all independent. To remove some of the redundancies, Lagos et al. (2016, 2018) built a corresponding parametrization at the level of the action which they call the EFT of cosmological perturbations. As a result, the maximum needed number of parameters drops to 12 in this EFT parameterization compared to 22 in the EFT formalism above. This provides an extension to the scalar–tensor EFT approach that we discussed in Sect. 5.1.

Finally, we conclude this section by a most recent work of Clifton and Sanghai (2018) where the authors proposed a set of 4 parameters to model minimal deviations from GR (metric theories) that can be used to cover scales at solar systems, galactic, and cosmological scales all the way to super-horizon. Two of the parameters are the well-known effective gravitational constant ($\mu$) and the slip parameter (that they note $\zeta$). They apply consistency relations in order to connects the behavior of these parameters between small and large scales. They show that using these conditions, $\mu$ and $\zeta$ can be expressed on small and large scales using 4 parameters $\{\alpha ,\gamma ,\alpha _c,\gamma _c\}$. The first two parameters are the same as the PPN parameters but allowed to vary at cosmological scales while the two other are specific to cosmological evolution and enters the two Friedmann equations. They refer to the set as PPNC. It will be interesting to see applications of this set to currently available data.

### Remarks on transition to nonlinear scales

A legitimate question is to ask if the various parametrizations and approaches discussed above could deal (or be extended to deal) in some way with nonlinear scales. A related question is if any parametrizations can deal with the nonlinear scales then can they reflect accurately any screening mechanism (see Sect. 8) at work in models.

First, the phenomenological MG parameterization using $\mu$, $\eta$, $\varSigma$ and other related parameters have been proposed based on the linearly perturbed Einstein equations so they are constrained to only linear scales by construction. Most recently, Clifton and Sanghai (2018) proposed a scheme (or parametrization) that is argued to link between MG parametrization at small scales and large scales. The idea is based on two parameters they put between quote marks as the “slip” and the “effective Newton’s constant” that can be written in terms four functions of time. Two of these four functions are a direct generalization of the usual $\alpha$ and $\gamma$ parameters from PPN formalism at small scales, see e.g., Will (2014). This development uses concepts of averaging small scales to larger scales. This very recent proposal came in a short paper and is at a very early stage at the moment of writing this review. It will be interesting to follow further development of this work and any clarifications on how it could deal with any screening mechanisms and other relevant questions.

Second, when considering the measure $E_G$ at nonlinear scales, it was observed in Leonard et al. (2015) that there was a difference between $E_G(\ell )$ as given by Eq. (111) and $E_G(R)$ as given by Eq. (113). They state that while $E_G(\ell )$ is defined in Fourier-space and includes only linear scales, that is not necessarily the case for $E_G(R)$ which is defined in real space and scales are not separated in an easy way. They found that the inclusion of non-linearities in the correlation function used into $E_G(R)$ do not cause the measure to deviate from the expected GR value at small scales. They attribute this to fact that nonlinearities enter into $\varUpsilon _{gm}(R)$ and $\varUpsilon _{gg}(R)$ (i.e., the galaxy-matter and galaxy-galaxy annular differential surface densities) via the same combination of correlation function terms, so they effectively cancel out from the ratio. It remains an open question whether such a behavior is also expected for modified gravity models.

Third, the PPF formalism of Hu and Sawicki (2007b) was proposed with a prescription on how to derive the nonlinear matter power spectrum in modified gravity theories that should in principle capture the screening mechanism as well. The prescription is based on the assumption that such a nonlinear power spectrum should reduce to that of GR on small scales. The fitting formula they proposed is as follows

124 $$\begin{aligned} P(k,z)=\frac{P_{\mathrm{non-GR}}(k,z)+c_{\mathrm{nl}}\varSigma ^2(k,z)P_{\mathrm{GR}}(k,z)}{1+c_{\mathrm{nl}}\varSigma ^2(k,z)}, \end{aligned}$$

where $P_{\mathrm{GR}}$ is for the nonlinear power spectrum in a GR-$\varLambda$CDM model that has the same expansion history as that of the modified gravity model under consideration. $P_{\mathrm{non-GR}}$ is for the nonlinear power spectrum in this modified gravity but without the screening mechanism necessary to recover GR on small scales. In other words, the fitting formula corrects the MG power spectrum to fit GR at small scales. The weighting function,

125 $$\begin{aligned} \varSigma ^2(k,z)\equiv \frac{k^3}{2\pi ^2}P_{\mathrm{lin}}(k,z), \end{aligned}$$

represents the degree of nonlinearity and governs the degree of screening efficiency. $P_{\mathrm{lin}}$ is the linear power spectrum in the modified gravity model. The $c_{nl}$ are coefficient (but can also be time-dependent) to control the scale of the effect. See, e.g., Hu and Sawicki (2007b).

Koyama et al. (2009) did further fitting using the PPF formalism with prescription above and added an exponent *n* on the right of Eq. (125). They found that $n=1$ for DGP and $n=1/3$ for *f*(*R*) provide good fits to N-body simulations of the models up to $k\sim 0.5$ h/Mpc. They also determined values for $c_{nl}$ in their fitting work. Zhao et al. (2011) used an exponent *n* as a function of *k* and 3 parameters. They extended the good fit to N-body simulations up to $k=10$ h/Mpc for *f*(*R*) models. These two studied and others found that the Chameleon mechanism at work was accurately reproduced by the implementation of this prescription.

Lombriser et al. (2014) and Lombriser (2014) combined the spherical collapse model, the halo model, linear perturbation theory, quasi-nonlinear interpolation motivated by the $c_{nl} \varSigma ^2(k,z)$ above and one-loop perturbations in order to derive a description of nonlinear the nonlinear matter power spectrum of f(R) gravity with chameleon screening on scales of up to $k\sim 10$ h/Mpc. This encouraged Lombriser (2016) to push further the method above of combining the perturbative approach with one-halo contributions obtained from a generalized modified spherical collapse model. The author proposed a parametrization based on the spherical collapse that enters into effect as one transitions into the deep nonlinear regime. The formalism he proposed allows one to encode different screening mechanisms at work in scalar–tensor theories. This sophisticated parametrization is then combined with generalized perturbative approaches to give a formalism that constitutes a nonlinear extension to the linear PPF formalism discussed above. For a detailed description, see Lombriser (2016).

Finally, there have been some recent proposals of extending the EFT formulation of the dark energy to nonlinear scales such as in, e.g., Cusin et al. (2018) for the Vainshtein mechanism, or to develop post-Newtonian–Vainshtein formalism that can be connected to it, see e.g., McManus et al. (2017) and Bolis et al. (2018). It was highlighted in Lombriser et al. (2018) that the EFT formulation of dark energy they explore in their paper can be connected to the nonlinear parameterization developped in Lombriser (2016). The topic of expanding the EFT formulation of dark energy to nonlinear regime is a subject of interest in the most recent literature and is to be followed very closely.

## Constraints and results on MG parameters (i.e., deviations from GR) from current cosmological data sets

In this section we describe current results on testing MG phenomenological parameters from cosmology. These are only a subset of selected available papers and results in the literature. We aimed here to focus on some of the recent results, or in some cases, on less recent constraints but those that helped exclude substantial regions of MG parameter spaces. We organize this section by the parameterizations described above and then by probes and surveys.

### Constraints on modified growth parameters

#### Constraints from Planck CMB, ISW, CMB lensing, and other data sets

We start with the XIVth paper of the Planck 2015 data release (Ade et al. 2016b) that was dedicated to dark energy and modified gravity models beyond $\varLambda$CDM (we hereafter refer to the paper as Planck2015MG). The authors used Planck CMB temperature, polarization and CMB lensing data sets combined with several other data sets as follows. They defined Planck low-$\ell$ data their temperature and polarization multipoles with $\ell \le 29$ (noted therein as “lowP”), and also the high-$\ell$ temperature-only data (noted Planck-TT) with $30 \le \ell \le 2500$. They also used their CMB lensing data which is sensitive to dynamical dark energy and late-time modification to gravity (Ade et al. 2016c). Planck2015MG considered BAO as the primary data set to be combined with CMB in order to break degeneracies among cosmological parameters constrained by the background evolution and used data from Ross et al. (2015), Anderson et al. (2014) and Beutler et al. (2011). They used supernova data from the (JLA) compilation (Betoule et al. 2013, 2014). They also used a local measurement of the Hubble constant, $H_0=70.6\pm 3.3$  km $\mathrm s^{-1}$ Mpc$^{-1}$, from Efstathiou (2014) who reanalyzed the results of Riess et al. (2011). For constraints on the growth-rate of large scale structure, Planck2015MG used constraints on $f\sigma _8$ from the RSD data compilation of Samushia et al. (2014) (see references therein) as well as weak lensing data from the CFHTLenS survey using the 2D data of Kilbinger et al. (2013) and the tomography data from only blue-galaxies in order to avoid any intrinsic alignment contamination present in the red-galaxies (Heymans et al. 2013).

For MG parameters, Planck2015MG constrained $\mu (k,a)$, $\eta (k,a)$, and $\varSigma (k,a)$ as defined earlier in Eqs. (90), (91), and (92) but added to them specific time and scale dependencies. They defined a parametrization that is similar to that described in (104) (Bertschinger and Zukin 2008) for the quasi-static regime but which is more general and covers a wider range of scales (Ade et al. 2016b). For the time evolution they considered two cases, one where the dependence is expressed via the effective dark energy density $\varOmega _\mathrm{{DE}}(a)$, and a second case where the scale factor appears directly in the parametrization. They also split the time evolution using $E_{ij}$ constants, $i,j-1,2$ to represent early and late time evolution. The $E_{ij}$ parameters are constrained from the data and the parameters $\mu$, $\nu$ and $\varSigma$ are reconstructed from them.

**Fig. 5:**
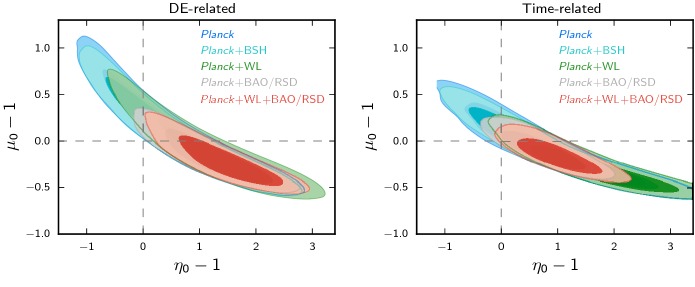
Contour plots for marginalized posterior distributions for 68% and 95% C.L for the two parameters $\{\mu _0-1,\eta _0-1\}$ at the present time with no scale dependence. On the left, time dependence is considered via the effective dark energy density parameter. On the right panel, time evolution is considered by direct inclusion of the scale factor. Results discussed in text of Sect. 6.1.1. The label *Planck* stands for *Planck*TT+TEB Figure reproduced with permission from Ade et al. (2016b)

However, Planck2015MG found that the current data can not meaningfully constrain the scale dependent MG parameters and that the inclusion scale dependence have very little effect on the $\chi ^2$ value of the best fit. Therefore their main MG parameter analysis was carried out without scale dependence except for a small illustrative example.

**Fig. 6:**
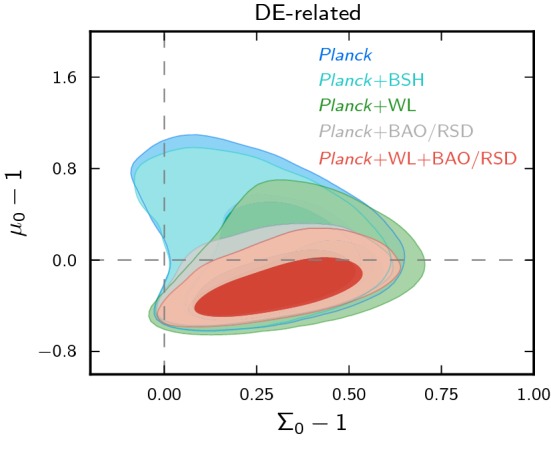
Contour plots for marginalized posterior distributions for 68% and 95% C.L for the two parameters $\{\mu _0-1,\eta _0-1\}$ at the present time with no scale dependence. The time dependence is considered via the effective dark energy density parameter. $\varSigma$ is obtained from Eq. (92). Results discussed in text of Sect. 6.1.1. In the labels, *Planck* stands for *Planck*TT+TEB Figure reproduced with permission from Ade et al. (2016b)

We reproduce here their Fig. 14 (see Fig. 5 here), their Fig. 15 (see Fig. 6) and their Table 6 (see Table 3 here) showing constraints on $\mu (k,a)$, $\eta (k,a)$, and $\varSigma (k,a)$ from various combinations of Planck and other data sets. Note that Planck2015MG use on their figures or tables *Planck* to refer to the combination Planck TT $+$ lowP data. We expanded that in the header of Table 3 for clarity.

Their reproduced Figs. 5 and 6 show that while, $\mu (k,a)$, $\eta (k,a)$, and $\varSigma (k,a)$ are close to their GR value of 1, some tension with GR is present and they provide some explanations for the source of such tension. This is indicated by the dashed horizontal and vertical lines in Fig. 5. In case (1) above, with time evolution based on effective $\varOmega _{\mathrm{DE}}(a)$, the tension is at the 2$\sigma$ level for Planck TT $+$ lowP data and rises above 2$\sigma$ when the constraints are tightened by adding the BAO $+$ RSD data. The tension reaches 3$\sigma$ level for Planck TT $+$ lowP $+$ WL $+$ BAO $+$ RSD combination. For case (2), with time evolution depending directly on *a*, there is less tension. It goes from 1-$\sigma$ for Planck TT $+$ lowP data to 2-$\sigma$. They commented that the latter increase from 2 to 3-$\sigma$ in the tension is mainly driven by the additional external data sets and so is the goodness of the fit of the models with the two additional MG parameters that show an improvement that ranges from $\delta \chi ^2=-6.3$ when using Planck $+$ lowP to $\delta \chi ^2=-10.8$ when combining Planck TT $+$ lowP $+$ WL $+$ BAO $+$ RSD, compared to the $\varLambda$CDM.

**Table 3:** Marginalized mean values and 68 % C.L. errors on cosmological parameters and the MG parameters $\{\mu _0-1,\eta _0-1\,\varSigma _0-1\}$ at the present time with no scale dependence

Parameter	Planck TT $+$ lowP	Planck TT $+$ lowP $+$ BSH	Planck TT $+$ lowP $+$ WL	Planck TT $+$ lowP $+$ BAO/RSD	Planck TT $+$ lowP $+$ WL $+$ BAO/RSD	Planck TT,TE, EE $+$ lowP $+$ BSH
$\mu _0-1$	$0.07^{+0.24}_{-0.51}$	$0.04^{+0.22}_{-0.48}$	$-\,0.14^{+0.13}_{-0.34}$	$-\,0.17^{+0.14}_{-0.23}$	$-\,0.21^{+0.12}_{-0.21}$	$0.06^{+0.23}_{-0.48}$
$\eta _0-1$	$0.70\pm 0.94$	$0.72\pm 0.90$	$1.36^{+1.0}_{-0.69}$	$1.23\pm 0.62$	$1.45\pm 0.60$	$0.60\pm 0.86$
$\varSigma _0-1$	$0.28\pm 0.15$	$0.27\pm 0.14$	$0.34^{+0.17}_{-0.14}$	$0.29\pm 0.13$	$0.31\pm 0.13$	$0.23\pm 0.13$
$\tau$	$0.065\pm 0.021$	$0.063\pm 0.020$	$0.061^{+0.020}_{-0.022}$	$0.062\pm 0.019$	$0.057\pm 0.019$	$0.060\pm 0.019$
$H_0$ $\left( \frac{\mathrm{km}}{s\,\mathrm{Mpc}}\right)$	$68.5\pm 1.1$	$68.17\pm 0.58$	$69.2\pm 1.1$	$68.26\pm 0.69$	$68.55\pm 0.66$	$67.90\pm 0.48$
$\sigma _8$	$0.817^{+0.034}_{-0.055}$	$0.816^{+0.031}_{-0.051}$	$0.786^{+0.021}_{-0.037}$	$0.792^{+0.021}_{-0.025}$	$0.781^{+0.019}_{-0.023}$	$0.816^{+0.031}_{-0.051}$

The time dependence is considered via the effective dark energy density parameter. $\varSigma$ is obtained from Eq. (92). Results discussed in text of Sect. 6.1.1. Table reproduced from Ade et al. (2016b)

Planck2015MG comment that the tension above can be understood from their Fig. 1 showing that the best fit power spectrum Planck TT $+$ lowP prefers models with slightly less power in the CMB at large scales (i.e., ISW effect) and models with a higher CMB lensing potential when compared to the $\varLambda$CDM model. They state that this point corroborates with the fact that MG parameters departing from GR values are found to be degenerate with the lensing amplitude parameter $A_L$. This is simply a non-physical scaling parameter to check how the CMB power spectrum is affected by lensing. It should be equal to 1 for consistency. Calabrese et al. (2008) found that $A_L$ is not equal to 1 when using the $\varLambda$CDM model, but Planck2015MG find that if MG parameters are allowed to vary then $A_L$ becomes consistent with unity again but then MG parameters move away from their $\varLambda \mathrm {CDM}$ value. However, Planck2015MG points out that CMB lensing analysis from the 4-point function of Ade et al. (2016c) is consistent with $A_L=1$ and in agreement with $\varLambda \mathrm {CDM}$ with no requirement of a higher lensing potential. Therefore, when Planck2015MG use this CMB Lensing data, the MG parameter confidence contours are shifted to regions where the tensions above are removed (fall to 1-$\sigma$ for CMB data only and below 2-$\sigma$ for all data combined). GR and $\varLambda$CDM provides a good fit then. It is worth noting though that recent work confirms some tension between Planck temperature and polarization data versus Planck CMB Lensing data (Motloch and Hu 2018).

Their Fig. 16 and Table 7 provide a summary of the tensions with and without CMB Lensing where they present the tension using departure from the line of maximum degeneracy between the two MG parameters.

Their Table 6 (Table 3) shows the corresponding marginalized mean values and the 65% CL errors on the MG parameters for each combination of data sets. This shows the explicit constraints on MG parameters and the tensions reported above. As commented in Planck2015MG, the addition of the BAO $+$ SN $+$ H does not improve significantly the MG constraints while the RSD data does provide a noticeable improvement, as expected. Finally, as shown in their Fig. 18, the current available data is not able to provide useful constraints when the scale dependence of the MG parameters is included in the analysis.

#### Constraints on MG parameters from mainly weak lensing data

*KIDS-450 + other data sets*

Joudaki et al. (2017) conducted a detailed analysis to test extensions to the standard $\varLambda$CDM cosmological model including constraints on deviations from GR using weak lensing tomography using 450 deg$^2$ of imaging data from the Kilo Degree Survey (KiDS) (Hildebrandt et al. 2017). The authors also used the Planck temperature and polarization measurements on large angular scales ($\ell \le 29$) using low-$\ell$ (TEB likelihood) and temperature only (TT) at smaller scales (PLIK TT likelihood) (Ade et al. 2016a). They explored if any of the extensions to the standard model could alleviate the tension reported in Hildebrandt et al. (2017) between KiDS and Planck constraints. The extent and sources of these tensions has been put into question though by Efstathiou and Lemos (2018).

They used the parameterization *Q*(*k*, *z*) and $\varSigma (k,z)$ as in (86) and (88), and binned in scale and redshift similar to Table 2, with transitions at $\mathrm{k}= 0.05 \mathrm{h}\, {\mathrm{Mpc}}^{-1}$ and $\mathrm{z} = 1$. They used as lensing statistics, the correlations functions in Eq. (70). They included in their analysis all of the key lensing systematics such as intrinsic alignments of galaxies and baryonic effects by modeling them and adding the corresponding parameters to be also constrained by the data. They used for the MG part of their analysis the ISiTGR software (Dossett et al. 2011b) which is a modified version of CosmoMC and CAMB (Lewis and Bridle 2002; Lewis et al. 2000) (see Sect. 11.1).

**Fig. 7:**
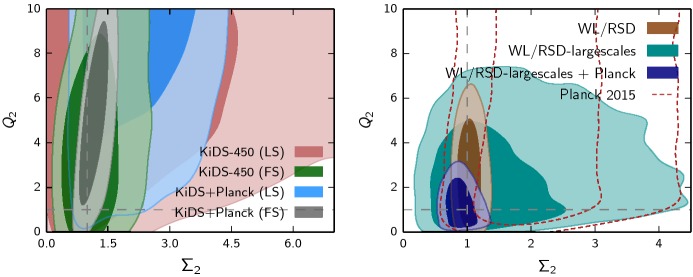
Left: Marginalized posterior contours (inner 68% CL, outer 95% CL) in the $Q_2 - \varSigma _2$ space for KiDS with fiducial angular scales shown in green (labeled by ‘FS’), KiDS keeping only the largest angular scales shown in pink (labeled by ‘LS’), and combined with Planck in grey and blue, respectively. The indices represent the combination of MG bins, such that $z < 1$ and $k > 0.05~h~{\mathrm{Mpc}}^{-1}$. The intersection of the dashed lines give the GR prediction (i.e., $Q = \varSigma = 1$). Reproduced with permission from Fig. 13 in Joudaki et al. (2017). Right: In addition to the cases described on th left, the constraints include galaxy–galaxy lensing correlation with cosmic shear in WL and RSD data as described in the text. ’Large-scale cuts’ mean that small scales have been excluded because of no adequate modeling for generic MG deviations in the nonlinear regime that can be utilized here. Again, the intersection of the horizontal and vertical lines is the GR prediction (i.e., $Q = \varSigma = 1$)

We reproduce the right panel of their Fig. 13 (see left panel of Fig. 7 here) showing the constrains on $Q_2$ and $\varSigma _2$. As shown on the figure, KiDS constraints are consistent with GR and are mainly sensitive to $\varSigma _2$ as expected for lensing constraints. The authors report that this is also the case for the other 6 $Q_i$ and $\varSigma _i$ parameters. Furthermore, using $\chi ^2$ and other Bayesian tests, they find that the data has no significant preference for the model with additional MG parameters compared to $\varLambda$CDM. The tension between Planck and KiDS goes away but they attribute that to the weakening in the constraints due to the additions of 8 MG parameters. They conclude that their data (combined with Planck) has no preference for a deviation from GR. They found instead that a model with a dynamical dark energy and a time-evolving equation of state is moderately preferred by the data and alleviates the tension between their data and Planck.

In a subsequent study (Joudaki et al. 2018), the authors combined KiDS lensing tomography data and the overlapping areas from two spectroscopic redshift galaxy clustering surveys: 2dFLenS (Blake et al. 2016a) and BOSS (Dawson et al. 2013; Anderson et al. 2014). The same Planck data as above was used again. They performed cosmological parameter constraints including MG parameters using three large-scale structure measurements: cosmic shear tomography, galaxy-galaxy lensing tomography, and redshift-space distortions (RSD) in the form of redshift-space multipole power spectra (Taylor and Hamilton 1996). This provided the analysis with significantly more constraining power and tightening of constraints on all parameters. However, this tightening of constraints also made the tension between large-scale constraints and Planck at the 2.6$\sigma$ level. They found that models with MG parameters could resolve the discordance in the linear/large-scale case, but are not favored by model selection. The same result stands for extended models with massive neutrinos, curvature or evolving dark energy. The big plus for constraints on MG parameters in their analysis comes from the complementarity between cosmic shear that is sensitive to the sum of the two potentials via light deflection, i.e., $\varPsi +\varPhi$, and the redshift space distortions that are sensitive to the potential $\varPsi$ via the matter growth of large scale structure. They use the same bins in redshift and scale for MG parameters as above and keep the background cosmology as a $\varLambda$CDM one.

We reproduce the right panel of their Fig. 11 (in the right panel of our Fig. 7) showing the new constraints in $Q_2-\varSigma _2$ plane. These two parameters are in the second bin in redshift (i.e., $z<1$) and second bin in length-scale (i.e., $k>0.05\,\hbox {h}\,\mathrm{Mpc}^{-1}$). One can see a significant improvement in the constraints in the right panel compared to the left which highlights the importance of adding the RSD data and the galaxy-galaxy lensing correlation to cosmic shear data, as the authors stress in their conclusion.

For this WL $+$ RSD combined analysis, they find $Q_2 = 2.8^{+1.1}_{-2.0}$ and $\varSigma _2 = 1.04^{+0.11}_{-0.14}$, while for KiDS only in Joudaki et al. (2017) $\varSigma _2 = 1.23^{+0.34}_{-0.70}$ and unconstrained $Q_2$ within its prior range. These and all other constraints on the six other modified gravity parameters are all consistent with the GR values of unity. The tightest constraints in this analysis come from combining cosmic shear, galaxy–galaxy lensing correlation, RSD and Planck $Q_2 = 1.28^{+0.41}_{-1.00}$ and $\varSigma _2 = 0.90^{+0.14}_{-0.18}$. As they comment, these are conservative results since only large-scale cuts are used which are found consistent with Planck. This is a good improvement from the previous analysis above with large-scale ‘KiDS cosmic shear +Planck’ constraints where $Q_2 > 2.2$ (restricted by the upper bound prior) and $\varSigma _2 = 2.13^{+0.58}_{-1.10}$. The authors conclude that as we will have more overlap between KiDS and 2dFLenS/BOSS, we will be able to obtain more stringent constraints using the data combination used here.

*CFHTLenS + other data sets*

Some years earlier, Simpson et al. (2013) used combined structure growth data from the CFHTLenS tomographic cosmic shear survey (Heymans et al. 2013; Benjamin et al. 2013), the WiggleZ Dark Energy Survey (Blake et al. 2012), and redshift space distortions from the 6dFGS (Beutler et al. 2012) to constrain MG parameters and deviations from the Newtonian potentials. They also used background data for $H_0$ from Riess et al. (2011), BAO data from Anderson et al. (2012), and Padmanabhan et al. (2012), as well as CMB temperature (TT) and polarization (TE) with data from WMAP7 (Komatsu et al. 2011).

They used a slightly modified parametrization so that our $\mu (k, a)$ and $\varSigma (k, a)$ in (91) and (92) are replaced by $[1 + \mu (k, a)]$ and $[1 + \varSigma (k, a)]$ respectively and now taking 0 value in the GR case instead of 1. They modeled the time-evolution of the MG parameter to scale with the background effective dark energy density as:

126 $$\begin{aligned} \varSigma (a) = \varSigma _0 \frac{\varOmega _\varLambda (a)}{\varOmega _\varLambda } \, , \, \, \, \, \mu (a) = \mu _0 \frac{\varOmega _\varLambda (a)}{\varOmega _\varLambda } \, , \end{aligned}$$

where $\varOmega _\varLambda \equiv \varOmega _\varLambda (a=1)$ is today’s value so that $\mu _0$ and $\varSigma _0$ represent today’s values of $\mu (a)$ and $\varSigma (a)$ as well, respectively.

They used measurements constraints on $(f\sigma _8,F)$ from the WiggleZ and 6dFGS surveys where *F*(*z*) represents the amplitude of the Alcock–Paczynski effect degenerate with the RSDs as we discussed in Sect. 4.3. These measurements are from three effective redshift slices from the WiggleZ $z = 0.44$, 0.60, and 0.73, with $\sigma _8(z) = (0.41 \pm 0.08, 0.39 \pm 0.06, 0.44 \pm 0.07)$ and $F = (0.48 \pm 0.05, 0.65 \pm 0.05, 0.86 \pm 0.07)$ plus a fourth data point of $f \sigma _8 = 0.423 \pm 0.055$ at a lower redshift $z=0.067$ from the 6dFGS with negligible sensitivity to the Alcock–Paczynski distortion.

In their analysis they considered the $\varLambda$CDM, the flat and non-flat wCDM models all augmented with the MG parameters $\mu _0$ and $\varSigma _0$. In all cases, they found no indication of departure from general relativity on cosmological scales. They put the following limits on MG parameters: $\mu _0 = 0.05 \pm 0.25$ and $\varSigma _0 = 0.00 \pm 0.14$ for a flat $\varLambda$CDM background model. They note that these correspond to deviations in the present-day Newtonian potential and spatial curvature potential of $\delta \varPsi /\varPsi _{\textit{GR}} = 0.05 \pm 0.25$ and $\delta {\varPhi } / \varPhi _{\textit{GR}} = -0.05 \pm 0.3$ respectively, with significant correlations between the errors. When they allow for *w* to vary for the background, these constraints change to $\mu _0 = -\,0.59 \pm 0.34$ and $\varSigma _0 = -\,0.19 \pm 0.11$. They also constrained the growth index parameter to $\gamma =0.52 \pm 0.09$ for a $\varLambda$CDM background model, thus in agreement with the GR value of $6/11=0.545$.

#### Constraints on MG parameters from various probes and analyses

Peirone et al. (2017b) perform an extensive analytical and numerical analysis of the MG parameters $\varSigma$ and $\mu$ or equivalently $G_\mathrm{light}/G$ and $G_\mathrm{matter}/G$. They consider Horndeski models that are broadly consistent with background and perturbation tests of gravity and the cosmic expansion history with late time acceleration. They also take into account the recent result from GW170817 and its counterpart GRB170817A, setting $c_T=c$. They confirm a conjecture they made in their earlier work (Pogosian and Silvestri 2016) about MG parameters in Horndeski models, that is $(\varSigma -1)(\mu -1)\ge 0$ (that is the two factors must be of the same sign) must hold in viable Horndeski models in the quasi-static approximation. They also discussed in their previous work (Pogosian and Silvestri 2016) consistency relations between the two MG parameters that, if broken would exclude some sub-classes of Horndeski models (e.g., $\varSigma \ne 1$ would rule out all models with a canonical form of kinetic energy). They remark that while the results of Ade et al. (2016b) indicate $\mu <1$ and $\varSigma >0$ are not statistically significant, however, if such values will hold in more precise experiments in the future that would rule out all Horndeski models. In the latter paper, they show that requiring no ghosts and no gradient instabilities prevents from having values within the $\varSigma -1>0$ and $\mu -1<0$ range. They also examined the conjectured condition versus the Compton wavelengths considered. They also found that observations from background expansion also put constraints on gravitational coupling which in turn re-enforces the conjecture limits. They also test the validity of the quasi-static approximation in Horndeski models finding that it holds well at small and intermediate scales but fails at $k\le 0.001$ h/Mpc. They conclude in their analysis that despite the stringent result from GW, there remain Horndeski models with non-trivial modifications to gravity at the level of linear perturbations and large scale structure. They stress the complementarity of different approaches used to constrain modification to GR and the practicality of using the phenomenological $\varSigma$ and $\mu$ parameterization and their consistency relations, see also Pogosian and Silvestri (2016).

Another analysis of these self-consistency relations between MG parameters and growth rate in Horndeski models was performed by Perenon et al. (2017). They considered accelerating Horndeski models with $-1.1 \le w_{\mathrm{eff}} \le -\,0.9$ and classified them according to their early or late time effects as follows. Late-time dark energy where both dark energy energy momentum tensor and non-minimal gravitational couplings are negligible at early times. Early-time dark energy where the dark energy momentum tensor is at work even at early times but non-minimal coupling happens at late time only. Finally, they call early modified gravity where both dark energy momentum and non-minimal gravitational couplings are also present at early time during matter domination. They proposed a convenient way to represent the viability of the models using two diagnostic planes: the $\mu (z)-\varSigma (z)$ and the $f(z)\sigma _8(z)-\varSigma (z)$ planes. They derived the following conclusions from their detailed analysis in the first plane. If model-independent measurements find either (i) $\varSigma -1<0$ at redshift zero or (ii) $\mu -1<0$ with $\varSigma -1>0$ at high redshifts ($z>1.5$) or (iii) $\mu -1>0$ with $\varSigma -1<0$ at high redshifts, Horndeski theories are ruled out. In the second plane, they found that: (i) If $f\sigma _8$ is found to be larger than that of $\varLambda$CDM model at $z>1.5$ then early dark energy models are ruled out. On the opposite case (for $f\sigma _8$), (ii) measuring $\varSigma <1$ will rule out late dark energy models, while, (iii) $\varSigma >1$, it is the early modified gravity case as described earlier in this paragraph that is allowed.

**Fig. 8:**
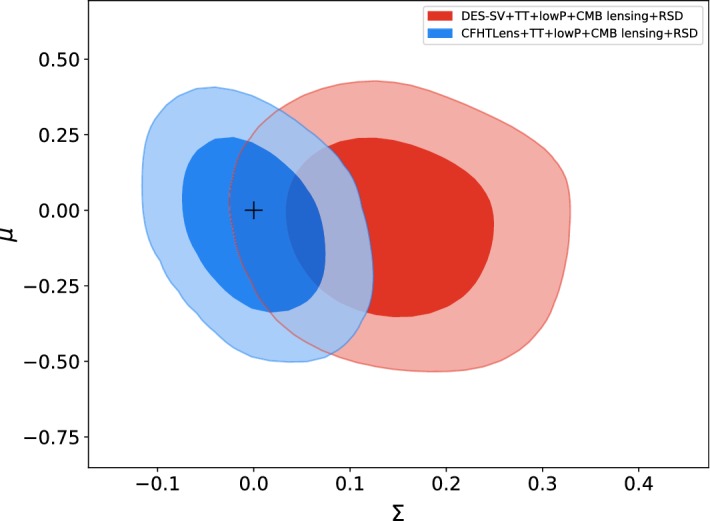
Contour plots for 68% and 95% CL on MG parameters $\varSigma$ and $\mu$ combining *Planck* CMB data (TT $+$ lowP $+$ CMB lensing), RSD data from BOSS DR12 and 6dFGS, and cosmic shear data from CFHTLenS in blue and DES-SV in red. The cross point represent the GR values (0,0) according to the authors’ definitions and show that GR is consistent with the data sets used. The combination for the contours in blue gives among the tightest current constraints on MG parameters as: $\varSigma = -0.01_{-0.04}^{+0.05}$ and $\mu = -0.06 \pm 0.18$ (68 % confidence level) Figure reproduced with permission from Ferté et al. (2017)

Ferté et al. (2017) performed an analysis to constrain the two MG parameters but using the definitions $[1 + \mu (a)]$ and $[1 + \varSigma (a)]$ to enter in the Poisson and lensing equations instead of $\mu (a)$ and $\varSigma (a)$ so taking 0 values in the GR case instead of 1. They also used Eq. (126) to model their time evolution scaling with the effective dark energy density parameter with no scale dependence since current data cannot constrain their scale dependence. They use CMB measurements from Planck, cosmic shear from CFHTLenS and DES science verification data, and RSD from BOSS DR12 and the 6dF galaxy survey. They derived constraints using this combination of probes but including either CFHTLenS or DES-SV separately for weak lensing finding the results shown in their Fig. 10 (reproduced here as Fig. 8). The constraining power of CFHTLens is larger leading to tighter constraints. In the DES-SV data, they also marginalized over the amplitude intrinsic alignment of galaxies finding a positive value leading to a higher value of $\varSigma$ as shown on the figure. The constraints found using CMB Planck (TT $+$ lowP $+$ CMB Lensing), RSD data (BOSS DR12$+$), and CFHTLenS cosmic shear are: $\varSigma = -0.01_{-0.04}^{+0.05}$ and $\mu = -0.06 \pm 0.18$ (68 % confidence level) which are among tightest current constraints on MG parameters. GR is consistent with these tightened bounds, although there is still room for deviations from it, in particular, for the $\mu$ parameter. The authors then perform some forecast analysis for improvement using five years of DES and LSST data showing substantial improvement on the parameters and in particular $\varSigma$ that we present in Sect. 13.

### Constraints on $f \sigma _8$ from galaxy surveys and RSD measurements 

Alam et al. (2017a) presented cosmological constraints from galaxy clustering data of the completed SDSS-III BOSS survey. The study used combined galaxy samples with 1.2 million galaxies. The spectroscopic survey used BAO methods to measure the angular diameter distance and the Hubble parameter. Most relevant to testing gravity, the survey constrained the growth of structure using the combination $f\sigma _8$ from RSD measurements. In this concluding analysis of SDSS-III BOSS, they combined individual measurements from seven previous companion SDSS papers into a set of consensus values for the angular diameter distance, the Hubble parameter and $f\sigma _8$ at 3 redshifts: $z=0.38,0.51, \,\mathrm{and }\,0.61$.

The method they employed to test deviations from GR was not based on using directly any MG parameters. They instead defined two parameters that rescale $f\sigma _8$ as follows:

127 $$\begin{aligned} f \sigma _8 \rightarrow f \sigma _8 [A_{f \sigma _8}+B_{f \sigma _8}(z-z_p)] \end{aligned}$$

with a redshift pivot $z_p=0.51$ (Alam et al. 2017a). GR will have $A_{f \sigma _8}=1$ and $B_{f \sigma _8}=0$.

**Fig. 9:**
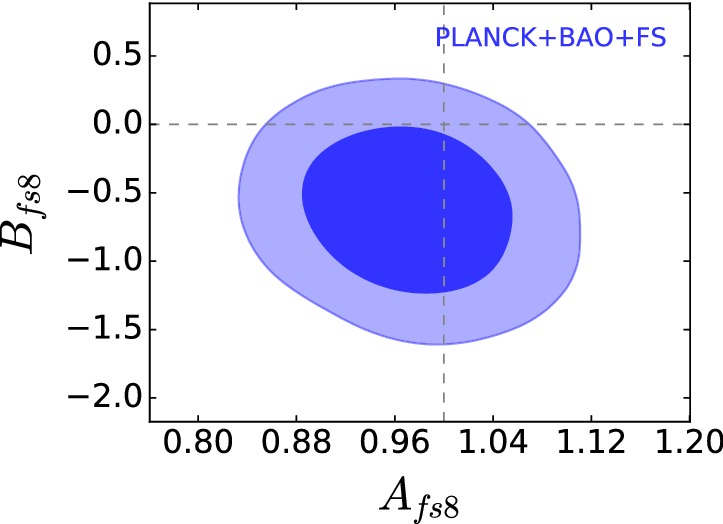
68% and 95% confidence contours on modification of the growth function in the $\varLambda$CDM cosmological model using the form $f \sigma _8 \rightarrow f \sigma _8 [A_{f \sigma _8}+B_{f \sigma _8}(z-z_p)]$ with a redshift pivot $z_p=0.51$. The results are consistent with the predictions of general relativity: $A_{f\sigma _8}=1$, $B_{f\sigma _8}=0$ (dashed grey lines). As explained in the text, the analysis used temperature and polarization data from Planck 2015, and a set of consensus values for BAO and RSD using full shape measurements (FS) from SDSS DR12 Figure reproduced with permission from Alam et al. (2017a)

They combined their RSD and BAO measurements along with temperature and polarization data from Planck-2015 (Ade et al. 2016a). For a $\varLambda$CDM background model and a redshift independent rescaling, they find $A_{f \sigma _8} = 0.96\pm 0.06$, so a growth amplitude value that is consistent with GR. When they allow for a redshift-dependent variation, they find $A_{f \sigma _8} = 0.97\pm 0.06$ and $B_{f \sigma _8} = -0.62\pm 0.40$. This is a 1.5-$\sigma$ deviation from a zero GR-value so they considered this as not statistically significant and concluded that their results are consistent with GR. They also found very little changes in these values when they allow for the equation of state *w* and the spatial curvature parameter to vary. We reproduce their Fig. 20 (as Fig. 9 here) showing consistency with GR of the two rescaling parameters (on the left). They also provide there (on the right panel) 11 measurements of $f\sigma _8$ from their work and other surveys as listed in the caption. We note that they used only BOSS RSD data in the results for $f \sigma _8$ above as they state other data come from a variety of analysis and modeling approaches but are nevertheless consistent with those of BOSS within the error bars shown. The authors note that the current growth measurements of $f \sigma _8$ reaffirm the validity of GR. It is worth noting though that some other MG models such as nDGP (see Sect. 7.5.2) or ${ RR }$ non-local gravity are still also consistent with RSD data due to the large error bars.

Another recent analysis in de la Torre et al. (2016) used RSD and galaxy-galaxy lensing from the final data set of VIMOS Public Extragalactic Redshift Survey (VIPERS) (de la Torre et al. 2013) combined with CFHTLenS data (Heymans et al. 2013) at a redshift range of at $0.5<z<1.2$. The joint analysis obtained measurements of $f\sigma _8(0.6)=0.48\pm 0.12$ and $f\sigma _8(0.86)=0.48\pm 0.10$. The galaxy-galaxy lensing does not add any improvement in constraining these values but alleviates the degeneracies with galaxy bias and $\sigma _8$. This allows the constraints to be separated as $\left[ f(0.6),\sigma _8(0.6)\right] =[0.93\pm 0.22,0.52\pm 0.06]$ and $\left[ f(0.86),\sigma _8(0.86)\right] =[0.99\pm 0.19,0.48\pm 0.04]$ in consistency with GR but again with errors bars large enough to allow for other MG models.

Most recently, Okumura et al. (2016) made a high redshift ($z\sim 1.4$) measurement of $f\sigma _8$ using the FastSound survey using the Subaru Telescope. They obtained $f(z)\sigma _8(z)=0.482\pm 0.116$ at $z\sim 1.4$ after marginalizing over the galaxy bias parameter $b(z)\sigma _8(z)$. The background expansion was fixed to that of a $\varLambda$CDM model and using the RSD measurements on scales above $8\,h^{-1}{\mathrm{Mpc}}$. This is a first measurement above redshift 1 and corresponds to $4.2\sigma$ detection of RSD. As shown in their Fig. 17 (Fig. 3 here), this high redshift measurement is consistent with GR but models such as covariant or extended Galileons (see Sect. 7.3.1), *f*(*R*) (see Sect. 7.4.1) and other MG models with varying gravitational constant were all found outside the 1-$\sigma$ bound. The figure shows the importance of high redshift RSD measurement in strongly constraining these models in the future. They note the combination of low-*z* and high-*z* RSD measurements will be useful in constraining gravity models without relying on CMB data.

Nesseris et al. (2017) gathered a compilation of 34 data points where they made corrections for model dependence. In order to avoid overlap and maximize independence of the data-points, they also constructed a sub-sample from this compilation that they call the ‘Gold’ growth data set with 18 data-points. They determine the best fit *w*CDM from the growth evolution equation using the gold data set and find it in 3-$\sigma$ tension with the best fit Planck-15/$\varLambda$CDM model parameters *w*, $\varOmega _m^0$ and $\sigma _8$. They found that the tension disappears if they allow for the evolution of the effective gravitational constant.

Finally, Kazantzidis and Perivolaropoulos (2018) constructed an extended compilation of 63 data points of $f\sigma _8$ published between 2006 and 2013, They correct the data for the fiducial model and find that using the whole set gives a best fit $\varOmega ^0_m-\sigma _8$ that is in a 5-$\sigma$ tension with the Planck-2015 $\varLambda \mathrm {CDM}$ parameter values. However, they show that the tension drops to below 1-$\sigma$ when they use the 20 most recent values while using the 20 earliest data gives a 4.5-$\sigma$ tension. They find that the drop in the tension using the recent data is due to the fact that these are at high redshift with large enough errorbars that accommodate GR and many other theories. They argue that it is more effective to obtain more data at redshift below 1 and with higher precision to be able to distinguish more effectively between gravity theories.

**Fig. 10:**
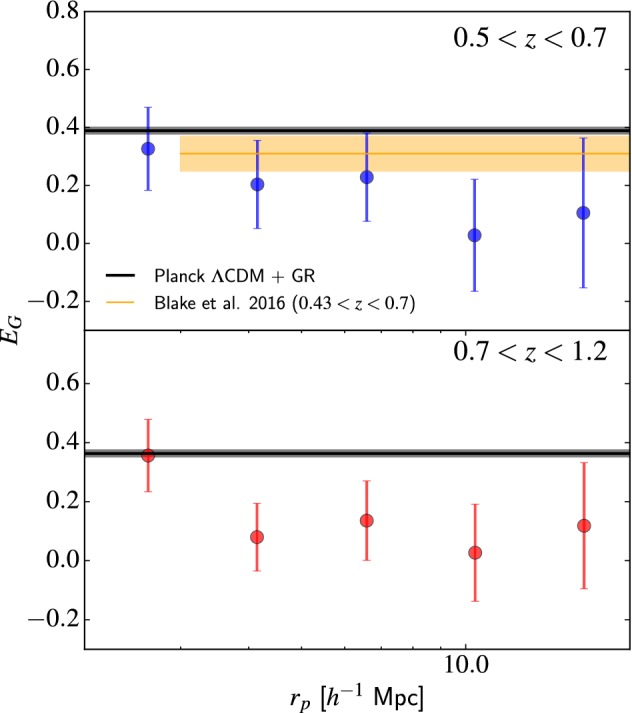
$E_G$ parameter as function of scale in redshift range $0.5<z<0.7$ (top panel) and $0.7<z<1.2$ (bottom panel) as measured in de la Torre et al. (2016). In the two panels, the solid curves and shaded areas around them correspond to the prediction and the $68\%$ uncertainty (thin grey) band for GR with a $\varLambda \mathrm {CDM}$ background set to the best fit model of TT $+$ lowP $+$ lensing Planck 2015 (Ade et al. 2016a). In the top panel, the horizontal (light brown) stripe shows the averaged $\smash {\overline{E}_G}$ over the range $3\,\hbox {h}^{-1} \mathrm{Mpc}<r_p<50\,\hbox {h}^{-1} \mathrm{Mpc}$ from Blake et al. (2016b) at redshift $0.43<z<0.7$ Figure reproduced with permission from de la Torre et al. (2016). As they comment there, this measurements of $E_{G}$ are slightly lower ($1-2\sigma$) than expected for the standard $\varLambda$CDM model of GR and one way to remedy to this is to lower the matter-density parameter. Figure reproduced with permission from Alam et al. (2017a)

### Constraints on $E_G$

Reyes et al. (2010) provided one of the first measurements of $E_G$ at redshift $z=0.3$ finding $E_G=0.39\pm 0.06$ in agreement with GR-$\varLambda$CDM value of $0.408 \pm 0.029$ although the uncertainties did not exclude some other possible alternative gravity theories such as *f*(*R*) (see Sect. 7.4.1) that predict a range of $E_G$ between 0.328 and 0.365. Nevertheless, the measured value was in a 2.5-$\sigma$ tension with the TeVeS models. The authors used 70,205 luminous red galaxies (LRGs) from the SDSS survey (Eisenstein et al. 2005) and the RSD measurement from this sample from Tegmark et al. (2006) of $\beta = 0.309\pm 0.035$ on large scales and at $z = 0.32$. The authors used galaxy-galaxy lensing and galaxy clustering of the LRG sample on Mpc scales and at this effective redshift. They used the slightly different version of $E_G$ as present in Eq. (113).

A more recent measurement of $E_G$ was achieved in Blake et al. (2016b) using deep and overlapping imaging and spectroscopic datasets by combining the Red Cluster Sequence Lensing Survey (RCSLenS) (Hildebrandt et al. 2016), the Canada-France-Hawaii Telescope Lensing Survey (CFHTLenS) (Heymans et al. 2013), the WiggleZ Dark Energy Survey (Blake et al. 2011c) and the Baryon Oscillation Spectroscopic Survey (BOSS) (Font-Ribera et al. 2014). They converted their measurements of galaxy-galaxy lensing, galaxy clustering and redshift space distortions into galaxy-matter annular differential surface densities ready to be used for the second definition of $E_G$ as given by (113). They found $E_G=0.48 \pm 0.10$ at $z=0.32$ and $E_G=0.30 \pm 0.07$ at $z=0.57$ when averaging over scales $10<R<50\,\hbox {h}^{-1}\mathrm{Mpc}$. These are both consistent with the perturbed GR-$\varLambda$CDM values of $E_G = 0.41$ and 0.36 at these respective redshifts. This confirms again GR but the uncertainties are still wide enough to allow for other MG theories. Next, a high-redshift measurement of $E_{G}$ came from de la Torre et al. (2016) who combined redshift space distortions from VIPERS and galaxy–galaxy lensing using the same portion of the sky from CFHTLenS. They found $E_G(z = 0.6) = 0.16 \pm 0.09$ and $E_G(z = 0.86) = 0.09 \pm 0.07$, when $E_{G}$ is averaged over scales above 3 Mpc/h. We reproduce their figure 17 as Fig. 10 here. As they comment, this measurements of $E_G$ gives values that are slightly lower than expected for the standard $\varLambda$CDM model of GR, but the results are consistent with GR within $1-2\sigma$.

Another interesting value of $E_G$ comes from Pullen et al. (2016) where the authors combined measurements of CMB lensing and galaxy velocity field. Unlike previous measurements of $E_G$, this one used CMB lensing instead of galaxy–galaxy lensing. The authors state that this will be less sensitive to contamination by intrinsic alignments of galaxies and will allow for the largest scale measurement of $E_G$ averaging over scales up to 150 $\,\hbox {h}^{-1}$ Mpc. They used cross-correlations of the Planck CMB lensing map with the SDSS III CMASS galaxy sample along with the CMASS galaxy auto-power spectrum and RSD. They used a definition of $E_G$ adapted to these probes [see their Eqs. (3) and (15)]. They find $E_G(z = 0.57) = 0.243 \pm 0.060$ (stat) $\pm 0.013$ (sys) The authors note that this measurement is in tension with GR at a level of 2.6-$\sigma$. Taking cosmological values from Planck-2015 and BOSS BAO, the GR value at $z=0.57$ is $0.402 \pm 0.012$. The authors noted that small tensions with GR start only when considering scales above 80 Mpc/h. They also comment that some deficit at very large scale in the CMB-Lensing galaxy cross power spectrum is present so they do not consider this as an indication of significant deviation from GR.

Alam et al. (2017b) combined data from BOSS CMASS sample DR11 galaxy clustering, CFHTLenS lensing and RSD of $\beta$ measurement from BOSS. They found $E_G(z=0.57)=0.42\pm 0.056$ which is in agreement (at 13% level) with the prediction of GR, $E_G(z=0.57)=0.396\pm 0.011$, using the Planck 2015 cosmological parameters. They corrected their results for systematics effects including scale dependence bias affecting its complete cancellation, difference in lensing and clustering windows and redshift weighting, intrinsic alignment of galaxies on lensing, cosmic variance, calibration bias in lensing, and limitations due to the choice of cutoff scale $R_0$. They run simulations and found that these theoretical observational systematic errors are smaller than the statistical errors in the measurement.

Amon et al. (2017) used the deep imaging data of the KiDS survey combined with overlapping spectroscopic areas from 2dFLenS, BOSS DR12 and GAMA surveys. They found $E_G(z = 0.267) = 0.43 \pm 0.13$ from using GAMA, $E_G(z = 0.305) = 0.27 \pm 0.08$ from using (BOSS LOWZ $+$ 2dF Low Z) and $E_G(z = 0.554) = 0.26 \pm 0.07$ from using (CMASS $+$ 2dF High Z). The results are consistent with GR with a $\varLambda$CDM background and linear perturbations. However, they found that their result and other measurements of $E_G$ favor a lower value of the matter density $\varOmega _m^0$ than the one preferred by Planck. They caution that the statistic $E_G$ is very sensitive to such a tension in the cosmological parameters which can have more effect than a deviation in GR and a change of as much as 10% in the gravitational potentials.

Most recently, Singh et al. (2018) used galaxy clustering from BOSS LOWZ sample with galaxy lensing from SDSS finding $\left<E_G\right>=0.37^{+0.036}_{-0.032}$ (statistical) $\pm 0.026$ (systematic) which is consistent with the GR predicted value (0.46) using Planck $\varLambda \mathrm {CDM}$ parameters and when both statistical and systematic errors are considered. Then they used BOSS LOWZ and Planck CMB lensing finding $\left<E_G\right>=0.43^{+0.068}_{-0.073}$ (stat). This is statistically consistent with SDSS galaxy lensing result and also with GR predictions. They found $\left<E_G\right>=0.39^{+0.05}_{-0.05}$ (stat) when using the CMASS sample and CMB lensing. The result is consistent with the GR prediction of 0.40 at the higher redshift of the CMASS sample. They also split the LOWZ sample into two redshift samples and found results on $E_G$ that are consistent with GR predictions at $2.5\sigma$ level (stat) or better. They found that nonlinear corrections and systematic effects can introduce errors $\sim 1$–2% so below the statistical errors while shear calibration and photometric uncertainties add another $\sim 5\%$ error for the SDSS galaxy lensing.

## Types of modifications to GR at cosmological scales and corresponding MG models

### Cartan–Weyl–Lovelock theorem

General relativity is based on well-defined principles and physical requirements discussed in Sect. 2, most of which, are encapsulated in the structure of the Einstein tensor and field equations. Einstein used guidance from such principles and requirements to shape and propose his theory (Einstein 1915). After that, Cartan (1922a), Weyl (1922), Vermeil (1917), and Lovelock (1971, 1972) worked on a succession of assertions and theorems about the uniqueness of Einstein’s tensor and the field equations. These led ultimately to the Lovelock theorem (Lovelock 1971, 1972) that can be summarized as follows, e.g., Ishak (2007) and Berti et al. (2015):

*In a spacetime of four dimensions, the only divergence free tensor of valence two that is constructed only from the metric tensor and its derivatives up to second order, and preserves diffeomorphism invariance, is the Einstein tensor plus a cosmological constant term.*

**Fig. 11:**
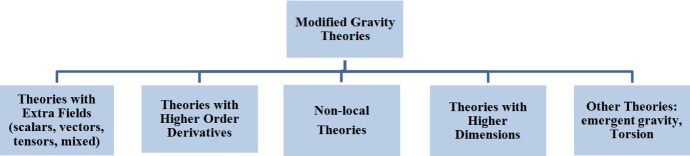
Various categories of modified gravity (MG) theories according to the principle or requirement they violate. It is worth noting that some models can belong to more than one category here like for example some higher dimensional models that have extra fields

Theories that deviate from GR can, in most cases, be delineated into categories according to what principle or requirement they violate. A modification to GR can thus happen by allowing (i) extra fields, (ii) higher-order metric derivatives, (iii) extra dimensions, (iv) non-locality or violation of Lorentz-invariance, see Fig. 11. Accordingly, MG models can be classified into the sub-categories described in the sub-sections that follow.

However, as mentioned already in the introduction, the scope of this review is to give an overview of the current status of testing GR at cosmological scales rather than providing a review of MG models. Therefore, we only provide some outlines of models of interest or models making a good illustrative case for a given category, while we refer the reader in each case to other specialized reviews in the literature. We refer the reader to some of the thorough reviews of MG models including (Clifton et al. 2012; Joyce et al. 2015; Berti et al. 2015) and references therein.

### Modified gravity versus dark energy

A question that keeps coming back in the community is what is the distinction between dark energy and modified gravity models. How to distinguish between the two as a cause of cosmic acceleration. There is more than one answer to this question but with some possible clear guidelines and prescriptions that can be set.

Joyce et al. (2016) and possibly others, propose to use the strong equivalence principle (SEP) (see Sect. 2.1) to draw a distinction between GR $+$ dark energy models versus MG models. They suggest to call any model that satisfies the SEP as a dark energy model and any model that violates SEP to be an MG model. They state that, heuristically, the SEP forbids the presence of a fifth force which motivates the use of such a discriminant. They state that using the SEP to make this distinction can be motivated further by the conjecture that GR is the only metric theory that satisfy the SEP, see Will (2014). They then state that a more pragmatic distinction is to rather use directly the presence (or not) of a fifth force to identify a model as being an MG model (or not) but with some grey zone as observed in for example Kunz and Sapone (2007).

Next, Amendola et al. (2013a) provided a phenomenological prescription to this question. First, they point to the simple case of quintessence that is straightforwardly referred to as dark energy model. In such a model, the scalar field is minimally coupled to curvature (see Sect. 7.3.1). In quintessence models, the scalar field also has a standard kinetic energy and the scalar potential represents the only functional degree of freedom. However, things get more ambiguous when moving beyond quintessence. The difficulty is that different models can have the same observables (Kunz and Sapone 2007). Also, some modified field equations can be recast into GR with extra source terms. Additionally, some scalar field dark energy models such as k-essence can have perturbations and clustering that can change the Poisson equation and induce a modified gravity parameter *Q*(*k*, *a*) signaling a deviation from GR. Therefore, they suggested and used the following prescription:

- Standard dark energy models: the scalar field here is non-minimally coupled to curvature in the Einstein’s equations and has standard kinetic energy. The dark energy has no clustering on sub-horizon with a sound speed equal to the speed of light. Quintessence is a well-known example or perhaps definition.
- Clustering dark energy: In this case dark energy has fluctuations and can cluster on sub-horizon scales. These perturbations in the dark energy modify the Poisson equation (86) by inducing an MG parameter $Q(k,z)\ne 0$. But in this case, no gravitational slip is allowed. That is $\eta (k,a)=0$ and the clustering dark energy does not cause any anisotropic shear. A good example is k-essence (Armendáriz-Picón et al. 2000, 2001). This is also the case for the no-slip gravity (Linder 2018).
- Modified gravity models: These are models where the Field equations are changed leading to changes in the Poisson equations with non vanishing slip parameter $\eta (k,a)$. These are characterized by the presence of fifth force and violate the SEP. Particles and bodies do not follow geodesics of the physical metric in the Einstein frame. This includes for example *f*(*R*) (see Sect.7.4.1), DGP (see Sect. 7.5.2), non-minimal coupled scalar–tensor theories and “dark energy” models with anisotropic clustering.

Amendola et al. (2013a) chose to follow the common practice of calling modified gravity models where GR is modified or where dark energy clusters. In other words, the last two items above. So models with $Q=\eta =1$ are dark energy models while if any of them departs from unity then it is an MG model. Of course, as they stress, this is not meant to be a fundamental classification but rather a convenient and useful phenomenological prescription.

### Modified gravity theories with extra fields

**Fig. 12:**
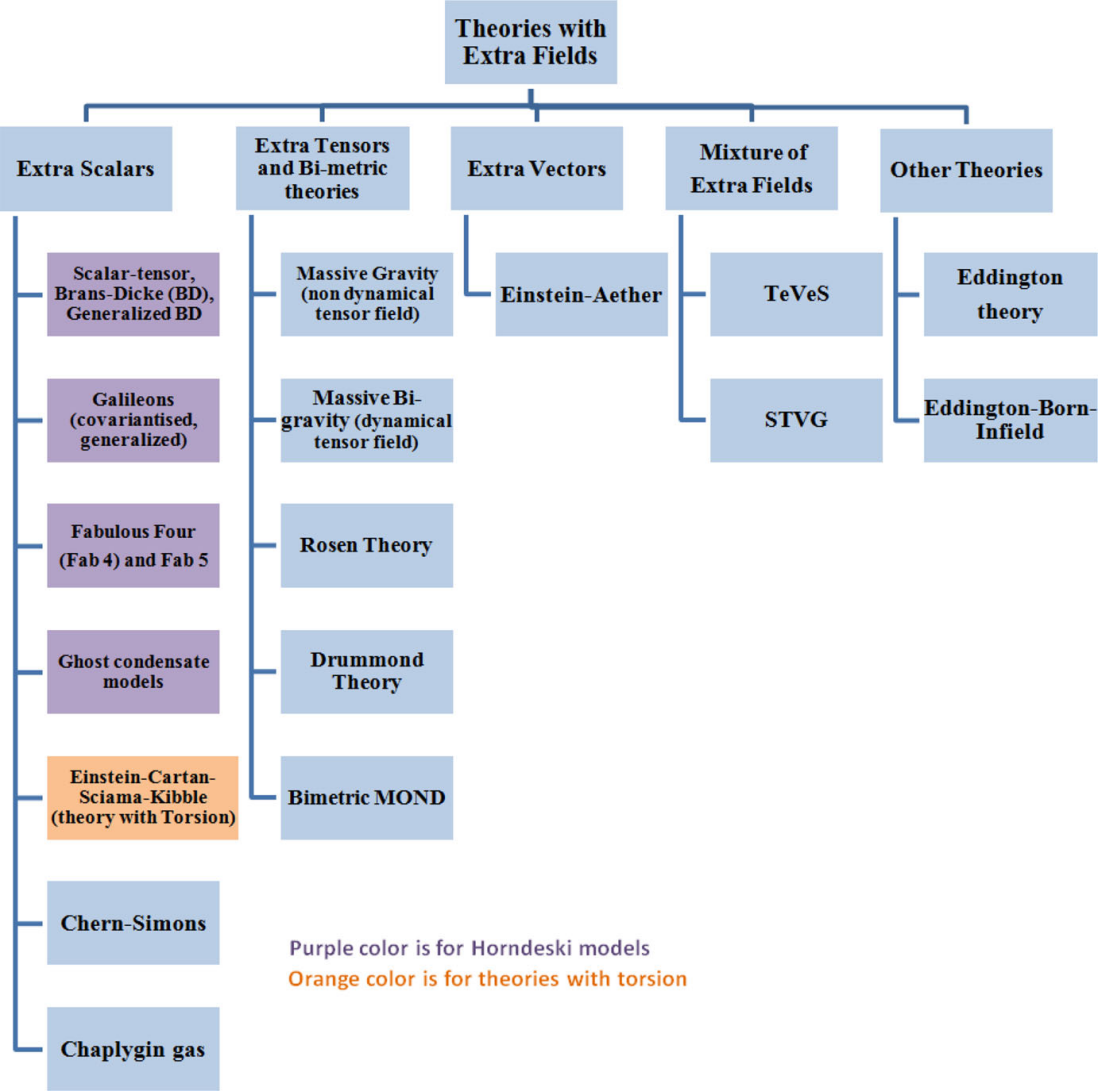
Extra-fields modified gravity models. Purple color is for Horndeski models and orange is for models with torsion. This table follows the models as listed in Clifton et al. (2012)

In this category, the modification comes from adding scalar, vector or tensor field(s) to the metric. Figure 12 provides examples of models for each sub-category and we provide below some illustrative examples for each sub-category.

#### Theories with extra scalar field

Scalar–tensor theories have been extensively studied in the literature from a theoretical point of view as well as comparison to observations, see for example Fujii and Maeda (2007) and references therein. Here a dynamical scalar field is added to the metric tensor hence the popular name. Let’s survey the following examples.

*Illustrative example 1: Generalized Jordan–Fierz–Brans–Dicke (GJFBD)*

The GJFBD models have been very popular as scalar–tensor theories of gravity physics at various regimes, see e.g., the reviews Will (2014), Clifton et al. (2012) and Koyama (2016). In cosmology, the interest recently shifted to Galileon (see Sect. 7.3.1) and Horndeski models because they can provide self-accelerating models. The Lagrangian for the GJFBD models can be written as,

128 $$\begin{aligned} \mathcal {L} = \frac{1}{16 \pi } \sqrt{-g} \left[ \phi R-\frac{\omega (\phi )}{\phi } \nabla _\mu \phi \nabla ^{\mu }\phi -2 \varLambda (\phi )\right] + \mathcal {L}_m(\psi _{\mathrm{m}}, g_{\mu \nu }), \end{aligned}$$

where $\omega (\phi )$ is a coupling function, $\varLambda (\phi )$ is a potential or a function generalizing the cosmological constant, and $\mathcal {L}_m(\psi _{\mathrm{m}}, g_{\mu \nu })$ is the Lagrangian of the matter field $\psi _{\mathrm{m}}$.

Variation of (128) with respect to the metric gives the first set of field equations,

129 $$\begin{aligned} \phi G_{\mu \nu } +\left[ \square \phi + \frac{1}{2}\frac{\omega }{\phi } (\nabla \phi )^2+ \varLambda \right] g_{\mu \nu } - \nabla _\mu \nabla _\nu \phi -\frac{\omega }{\phi } \nabla _\mu \phi \nabla _\nu \phi = 8\pi T_{\mu \nu }. \end{aligned}$$

while variations with respect to the scalar field provides, after some steps, the remaining equations,

130 $$\begin{aligned} (2 \omega +3) \square \phi + \omega ' (\nabla \phi )^2 +4 \varLambda -2 \phi \frac{d\varLambda }{d\phi } = 8 \pi T. \end{aligned}$$

The action (128) is written in the Jordan frame where the scalar field is non-minimally coupled to the Ricci curvature scalar. It is assumed that there exist in this frame a metric $g_{\mu \nu }$ to which all matter species are universally coupled and the particles follow geodesics of this metric. The scalar field does not couple directly to the matter fields.

One can transform (128) to the Einstein frame using a conformal transformation $g_{\mu \nu } =A(\phi )^2 \bar{g}_{\mu \nu }$ and by redefining the scalar field. In such an Einstein frame the scalar field is now minimally coupled to the Ricci scalar of $\bar{g}_{\mu \nu }$. However, the scalar field is directly coupled to the matter fields and test particles do not follow geodesics of $\bar{g}_{\mu \nu }$. The scalar field acts as an effective potential and isolated test particles feel a universal 4-acceleration.

A popular sub-case of the theory is the Jordan–Fierz–Brans–Dicke (JFBD) theory (Brans and Dicke 1961; Will 1994) obtained by setting $\omega$ as a constant noted as the Brans–Dicke coupling parameter $\omega _{_{_{\mathrm{BD}}}}$ and setting $\varLambda =0$, so (128) reduces to

131 $$\begin{aligned} \mathcal {L} = \frac{1}{16 \pi } \sqrt{-g} \left[ \phi R-\frac{\omega _{_{\mathrm{BD}}}}{\phi } \nabla _\mu \phi \nabla ^{\mu }\phi \right] + \mathcal {L}_m(\psi _{\mathrm{m}}, g_{\mu \nu }) \end{aligned}$$

where the Brans–Dicke field gives an effective gravitational constant. The theory approaches general relativity when $\omega \rightarrow \infty$.

Exact solutions for spherically symmetric vacuum in Brans–Dicke theory have been derived and compared to solar system observations, see for example Will (2014). The Cassini–Huygens mission (Bertotti et al. 2003) sets the constraints $\omega _{\mathrm{BD}}>40,000$ so Brans–Dicke must be very close to GR. Unless there is a successful screening mechanism at work at small scales, this bound makes it difficult for Brans–Dicke theories to depart from GR at cosmological scales. For example, Bisabr (2012) discuss Chameleon screened Generalized Brans–Dicke cosmology. However, as we discuss in Sect. 8.1, Wang et al. (2012) showed that such Chameleon screened models cannot explain cosmic acceleration unless we add a cosmological constant to them.

As for the cosmology of JFBD, the field equations for an FLRW metric and a perfect fluid source give, the following Friedmann equations:

132 $$\begin{aligned} H^2= & {} \frac{8 \pi {\bar{\rho }}}{3 \phi } - \frac{k}{a^2} - H \frac{\dot{\phi }}{\phi } + \frac{\omega }{6} \frac{\dot{\phi }^2}{\phi ^2} \end{aligned}$$

133 $$\begin{aligned} \frac{\ddot{\phi }}{\phi }= & {} \frac{8 \pi }{\phi } \frac{({\bar{\rho }}-3\bar{P})}{(2 \omega +3)} -3 H \frac{\dot{\phi }}{\phi }, \end{aligned}$$

where over-dots are for derivatives with respect to proper time. The general solutions to the Brans–Dicke equations above have been fully explored in e.g., Gurevich et al. (1973) and Barrow (1993).

In addition to the background equations, linear perturbations have been worked out in Nariai (1969), Wu et al. (2010), Nagata et al. (2002) and Chen and Kamionkowski (1999) so the theory can be compared to large scale structure and CMB data. For the perturbed FLRW metric (28) in the Newtonian conformal gauge, a dust source, scalar field perturbation $\phi =\phi _0+\delta \phi$, and assuming the quasi-static approximation, the following scalar perturbation equations are obtained, e.g., Koyama (2016):

134 $$\begin{aligned} \nabla ^2 \varPsi= & {} 4 \pi G a^2 \delta \rho - \frac{1}{2} \nabla ^2 \delta \phi , \end{aligned}$$

135 $$\begin{aligned} (3 + 2 \omega _{\mathrm{BD}}) \nabla ^2 \delta \phi= & {} - 8 \pi G a^2 \delta \rho , \end{aligned}$$

136 $$\begin{aligned} {\varPhi } - \varPsi= & {} \delta \phi . \end{aligned}$$

The perturbations of the scalar field act as an effective anisotropic stress producing a slip between the two potentials. Inserting (135) into (134) shows that the presence of the second term in Eq. (134) is equivalent to a modification to the Newton gravitational constant.

*Illustrative example 2: Galileon and Covariant Galileon models*

Galileon models were introduced by Nicolis et al. (2009) with some inspiration from DGP models (see Sect. 7.5.2) and aiming to go beyond their limitations. The models are constructed such that their action in flat spacetime is invariant under the following Galilean shift symmetry for the scalar field (Nicolis et al. 2009)

137 $$\begin{aligned} \partial _{\mu } \phi \rightarrow \partial _{\mu } \phi + c_{\mu }, \end{aligned}$$

where $c_{\mu }$ is a constant vector. It turns out that with such conditions, the most general Lagrangian, that gives second order derivative equations of motion, has only 3 terms, in addition to the scalar field term and its canonical kinetic term. The terms are Nicolis et al. (2009), Deffayet et al. (2009b):

138 $$\begin{aligned} {\mathcal {L}}_1^{\mathrm{gal}}= & {} \phi , \end{aligned}$$

139 $$\begin{aligned} {\mathcal {L}}_2^{\mathrm{gal}}= & {} -\frac{1}{2}(\partial \phi )^2, \end{aligned}$$

140 $$\begin{aligned} {\mathcal {L}}_3^{\mathrm{gal}}= & {} -\frac{1}{2}(\partial \phi )^2 \Box \phi , \end{aligned}$$

141 $$\begin{aligned} {\mathcal {L}}_4^{\mathrm{gal}}= & {} -\frac{1}{2} (\partial \phi )^2 \left[ (\Box \phi )^2-(\partial _\mu \partial _\nu \phi )^2\right] , \end{aligned}$$

142 $$\begin{aligned} {\mathcal {L}}_5^{\mathrm{gal}}= & {} -\frac{1}{4}(\partial \phi )^2 \left[ (\Box \phi )^3 -3\Box \phi (\partial _\mu \partial _\nu \phi )^2 +2(\partial _\mu \partial _\nu \phi )^3 \right] . \end{aligned}$$

The corresponding equations of motion can be found in Nicolis et al. (2009). A concise discussion on how this Lagrangian or other equivalent forms give only second order derivative equations of motion plus other properties of the models can be found in Koyama (2016), Clifton et al. (2012) and Nicolis et al. (2009). Galileon models can also result from conformal invariance (Nicolis et al. 2009; Creminelli et al. 2013) or from the action of a brane in a higher dimensional spacetime (de Rham and Tolley 2010). Galileon models can also be constructed with more than one scalar field (Trodden and Hinterbichler 2011; Trodden 2015; de Rham 2012), or in terms of vector fields (Tasinato 2014; Heisenberg 2014; Hull et al. 2014).

The next logical thing that was considered in literature was to convert Galileon models to curved spacetime. It was shown though that simply turning partial derivatives into covariant derivatives (covariantization) causes the appearance of third order derivatives in the equation of motion with the associated ghost fields (Deffayet et al. 2009a). To eliminate such higher order terms, Deffayet et al. (2009b) introduced counter terms to write the covariant Galileon action as:

143 $$\begin{aligned} {\mathcal {L}}_3= & {} -\frac{1}{2}(\nabla \phi )^2 \Box \phi , \end{aligned}$$

144 $$\begin{aligned} {\mathcal {L}}_4= & {} \frac{1}{8} (\nabla \phi )^4 R - \frac{1}{2}(\nabla \phi )^2 \left[ (\Box \phi )^2-(\nabla _\mu \nabla _\nu \phi )^2\right] , \end{aligned}$$

145 $$\begin{aligned} {\mathcal {L}}_5= & {} -\frac{3}{8} (\nabla \phi )^4 G^{\mu \nu } \nabla _\mu \nabla _\nu \phi \nonumber \\&- \frac{1}{4} (\nabla \phi )^2 \left[ (\Box \phi )^3 -3\Box \phi (\nabla _\mu \nabla _\nu \phi )^2 +2(\nabla _\mu \nabla _\nu \phi )^3 \right] . \end{aligned}$$

Models from Lagrangian up to ${\mathcal {L}}_3$ are referred to as Cubic Galileons, up to ${\mathcal {L}}_4$ as Quartic, and up to ${\mathcal {L}}_5$ as Quintic. The models are self-accelerating with no need for a cosmological constant. An example of a concise practical formulation of the models to compare to cosmological data can be found in for example Barreira et al. (2014b).

The covariant Galileon equations of motion are second and only second order derivatives. They are considered to be a subclass of the Horndeski models. A further generalization of the covariant Galileons to include zeroth and first order derivative equations of motion was carried out in Deffayet et al. (2009a) leading to the Horndeski action (147) (Horndeski 1974).

It is worth mentioning that Gleyzes et al. (2015a, b) have shown that the addition of the counter terms in the covariantization of Galileon action is not strictly necessary to obtain healthy models. The equations of motion can thus be still cast into second order due to further constraints. In fact, these led to the proposal of the so-called beyond Horndeski models (Zumalacarregui and García-Bellido 2014; Gleyzes et al. 2015a, b).

*Illustrative example 3: Horndeski models and beyond (* $\alpha _x$ *parameterization)*

This is the most general single-field scalar–tensor theory with second-order derivative equations of motion in (3$+$1) dimensions. A while ago, Horndeski (Horndeski 1974) derived the corresponding general Lagrangian and field equations but such work went quiet for sometime until their re-discovery within studies of generalized Galileon models, e.g. Deffayet et al. (2009a, b, 2011) and Kobayashi et al. (2011), finding that the generalized covariant Galileon models are equivalent to Horndeski models. Most recently, Horndeski models have been extensively studied analytically and phenomenologically but fully constraining them observationally remains a challenge, e.g. Ade et al. (2016b) due to their large number of parameters.

The Horndeski action is given by, e.g., Horndeski (1974), Deffayet et al. (2011) and Kobayashi et al. (2011)

146 $$\begin{aligned} S = \int d^4x \sqrt{-g} \left[ \sum _{i=2}^{5}{\mathcal {L}}_{i} + {\mathcal {L}}_M(g_{\mu \nu },\psi _{\mathrm{m}}) \right] \end{aligned}$$

where

147 $$\begin{aligned} {\mathcal {L}}_{2}= & {} K(\phi ,X),\nonumber \\ {\mathcal {L}}_{3}= & {} -G_{3}(\phi ,X)\Box \phi ,\nonumber \\ {\mathcal {L}}_{4}= & {} G_{4}(\phi ,X)\, R+G_{4X}\,[(\Box \phi )^{2}-(\nabla _{\mu }\nabla _{\nu }\phi ) \,(\nabla ^{\mu }\nabla ^{\nu }\phi )],\nonumber \\ {\mathcal {L}}_{5}= & {} G_{5}(\phi ,X)\, G_{\mu \nu }\,(\nabla ^{\mu }\nabla ^{\nu }\phi ) \nonumber \\&-\,\frac{1}{6}\, G_{5X}\,[(\Box \phi )^{3}-3(\Box \phi )\,(\nabla _{\mu }\nabla _{\nu }\phi ) \,(\nabla ^{\mu }\nabla ^{\nu }\phi )\nonumber \\&+\,2(\nabla ^{\mu }\nabla _{\alpha }\phi )\,(\nabla ^{\alpha }\nabla _{\beta }\phi ) \,(\nabla ^{\beta }\nabla _{\mu }\phi )]\ , \end{aligned}$$

where *K* and $G_{3}$–$G_{5}$ are functions of the scalar field $\phi$ and that of its kinetic energy, $X=-\partial ^{\mu }\phi \partial _{\mu }\phi /2$, *R* is the Ricci scalar, $G_{\mu \nu }$ is the Einstein tensor. $G_{iX}$ and $G_{i\phi }$ are the partial derivatives of $G_{i}$ with respect to *X* and $\phi$, respectively. The four functions, *K* and $G_{3}$–$G_{5}$ characterize completely this class of theories. The corresponding equations of motion can be found in Bellini and Sawicki (2014), Deffayet et al. (2011) and Kobayashi et al. (2011). It is worth noting that there are no a-priori mass or energy scales that are associated with the functions $G_{3}$–$G_{5}$ that would put them in some hierarchical order. When a model is specified, these functions may feature a mass scale that will determine at what scale they contribute to the dynamics. This mass scale is usually chosen so the terms have an effect at cosmological scales. The appearance of such mass scales differ though from one model to another within the Hordeski models. A brief discussion for Galileon models can be found after Eq. 3 in Baker et al. (2018).

A physically meaningful parameterization for the Horndeski models was introduced by Bellini and Sawicki (2014) from applying and specializing the EFT approach discussed in Sect. 5.1 to this class of models. First, for Horndeski models, the following relations between the functions of the EFT action (84) must hold:

148 $$\begin{aligned} m_2^2=0; \ 2{\hat{M}}^2 = {\bar{M}}^2_2 =-{\bar{M}}^2_3. \end{aligned}$$

Consequently, the nine EFT functions can be replaced by four functions of time only noted as $\alpha _M, \alpha _K, \alpha _B$ and $\alpha _T$ plus the effective Planck mass $M_*^2$ and an additional function of time for the background such as for example *H*(*a*) (Bellini and Sawicki 2014). These 5 functions of time and the effective Planck mass fully characterize the linear dynamics of the Horndeski models.

The relationships between the $\alpha _x$ parameters and the set of EFT functions of (85) have been provided in Bellini and Sawicki (2014) and can be summarized as follows:

149 $$\begin{aligned} \textit{M}_*^2= & {} m_0^2\varOmega + \bar{\textit{M}}_2^2 ; \end{aligned}$$

150 $$\begin{aligned} \textit{M}_*^2 H \alpha _M= & {} m_0^2 \dot{\varOmega } + \dot{\bar{\textit{M}}}_2^2; \end{aligned}$$

151 $$\begin{aligned} \textit{M}_*^2 H^2 \alpha _K= & {} 2c + 4 \textit{M}_2^4; \end{aligned}$$

152 $$\begin{aligned} \textit{M}_*^2 H \alpha _B= & {} -m_0^2 \dot{\varOmega } -\bar{\textit{M}}_1^3; \end{aligned}$$

153 $$\begin{aligned} \textit{M}_*^2 \alpha _T= & {} -\bar{\textit{M}}_2^2 . \end{aligned}$$

The authors also provided a connection between the physical properties of the theory and the $\alpha _x$ parameters as follows:

- $\alpha _K$: quantifies the *kineticity* of the scalar field originating from the presence of its kinetic energy term in the Lagrangian. For example, minimally coupled models such as quintessence or k-essence all have a scalar field kinetic term and thus $\alpha _K \ne 0$. On the other hand *f*(*R*) (see Sect. 7.4.1) or *f*(*G*) models have no such a term and thus $\alpha _K = 0$. In the general Horndeski models, $\alpha _K$ receives contributions from the Lagrangian functions *K*, $G_3$, $G_4$ and $G_5$, see Appendix A in Bellini and Sawicki (2014).
- $\alpha _T$: quantifies the excess of tensor (gravity waves) speed from the speed of light (i.e., $c^2_T-1$) and thus the deviation of gravitational waves speed from that of light. This also affects the coupling between the matter and the Newtonian potential resulting in anisotropic stress regardless of scalar perturbations. In the general Horndeski models, $\alpha _T$ receives contributions from the functions $G_4$ and $G_5$.
- $\alpha _B$: quantifies the *braiding* or mixing of the kinetic terms of the scalar field and the metric. Can cause dark energy clustering. $\alpha _B = 0$ for minimally coupled models of dark energy such as quintessence and k-essence but non-zero for all modified gravity models, i.e., all models where a fifth force is present (Pogosian and Silvestri 2016). $\alpha _B$ receives contributions from the functions $G_3$, $G_4$ and $G_5$ in Horndeski models.
- $\alpha _M$: quantifies the *running rate* of the effective Planck mass. It is generated by a restricted non-minimal coupling. It creates anisotropic stress. $\alpha _M = -\alpha _B \ne 0$ for *f*(*R*) models. $\alpha _M = 0$ for minimally coupled models of dark energy models such a quintessence and k-essence.

It is worth noting that because the $\alpha _x$-parameterization can be connected very well to physical properties of the of the models, it can then serve well the task of assessing the stability criteria of the models, see for example a recent discussion in Kennedy et al. (2018) and references therein.

Later on, Gleyzes et al. (2015b, 2015a) added to Eqs. (149)–(153) and the $\alpha _x$ parameterization the following relation

154 $$\begin{aligned} M_*^2 \alpha _H = 2 \hat{M}^2 - \bar{M}_2^2, \end{aligned}$$

where the authors introduced $\alpha _H\ne 0$ to parameterize a deviation from Horndeski models. Their formalism thus included viable models with a single scalar field but with higher-order equations of motion referred to as beyond-Horndeski models. However, the authors showed how internal constraints in the theory assures that it is free of Ostrogradski instabilities. Some of beyond Horndeski models are obtained by a disformal transformation. However, see some reservation and discussion in Crisostomi et al. (2016a) about the beyond-Horndeski characterization.

It is worth recalling here the definitions of conformal and disformal transformations of the metric given by

155 $$\begin{aligned} \bar{g}_{\alpha \beta } \; = \; A(\phi ,{X}) \, {g}_{\alpha \beta } \, + \, B(\phi ,{X})\, \partial _\alpha \phi \, \partial _\beta \phi \end{aligned}$$

where ${X} \equiv -\frac{1}{2}{g}^{\alpha \beta }\partial _\alpha \phi \, \partial _\beta \phi$. The first term on the right of (155) represents a conformal transformation rescaling the metric tensor. The second term is a pure disformal transformation stretching the metric in the direction given by $\partial _\alpha \phi$.

Further efforts continued to explore models beyond Horndeski. Remarkably, Langlois and Noui (2016a, b) identified the degeneracy conditions that assure that the theory is free from Ostrogradsky ghost even if their equations of motion have higher order derivatives. This allowed Langlois and Noui (2016a) and Crisostomi et al. (2016a) to identify viable beyond-Horndeski theories and even new classes of ghost free degenerate higher order theories in Langlois and Noui (2016a), Crisostomi et al. (2016b), Ben Achour et al. (2016a, b) and Crisostomi et al. (2017). The models introduced in Langlois and Noui (2016a) are now known as the degenerate higher derivative theories beyond Horndeski (DHOST), as dubbed in Langlois et al. (2017a) and the concise review (Langlois 2017). They generalize Horndeski and beyond-Horndeski models and are the most general class of ghost-free scalar–tensor theories. In these theories, it was shown in the vacuum in absence of matter coupling that the presence of a special degeneracy of the Lagrangian ensures the absence of ghosts even if the equations of motion are higher order. Also, if the matter coupling is disformal then it can not break this degeneracy but that is not the case for minimal coupling of matter. This is an interesting class of models that remain to be studied and compared to cosmological observations. We discuss some constraints on these models from neutron-star-merger event GW170817 and GRB170817A in Sect. 10.

If the coupling of matter is disformal (it can be minimal of course), then it could not break the degeneracy, and the ghost is indeed absent.

We reproduce for illustration purposes, Table 1 from Bellini and Sawicki (2014) where the $\alpha _x$ functions are given for known dark energy or modified gravity models (Table 4).

**Table 4:** Parameter functions $\alpha _{i}$ for various dark energy and modified gravity model sub-classes of Horndeski models

Model class	$\alpha _{\mathrm{K}}$	$\alpha _{{\text {B}}}$	$\alpha _{{\text {M}}}$	$\alpha _{{\text {T}}}$
$\varLambda { CDM}$	*0*	*0*	*0*	*0*
Cuscuton $(w_{X}\ne -1)$ (Afshordi et al. 2007b)	0	0	0	0
Quintessence (Ratra and Peebles 1988; Wetterich 1988)	$(1-\varOmega _{\text {m}})(1+w_{X})$	0	0	0
k*-essence*/perfect fluid (Armendáriz-Picón et al. 2000, 2001)	$\frac{(1-\varOmega _{\text {m}})(1+w_{X})}{c_{\text {s}}^{2}}$	0	0	0
Kinetic gravity braiding (Deffayet et al. 2010; Kobayashi et al. 2010b)	$\nicefrac {m^{2}\left( n_{m}+\kappa _{\phi }\right) }{H^{2}M_{\text {Pl}}^{2}}$	$\nicefrac {m\kappa }{HM_{\text {Pl}}^{2}}$	0	0
Covariant Galileon cosmology (Chow and Khoury 2009)	$-\nicefrac {3}{2}\alpha _{\text {M}}^{3}H^{2}r_{\text {c}}^{2}e^{2\phi /M}$	$\nicefrac {\alpha _{\text {K}}}{6}-\alpha _{\text {M}}$	$\nicefrac {-2\dot{\phi }}{HM}$	0
Imperfect fluid scalar–tensor (Sawicki et al. 2013)	$\nicefrac {\dot{\phi }^{2}K_{,\dot{\phi }\dot{\phi }}e^{-\kappa }}{H^{2}M^{2}}$	$-\alpha _{\text {M}}$	$\nicefrac {\dot{\kappa }}{H}$	0
Metric *f*(*R*) (Carroll et al. 2004; Song et al. 2007a)	0	$-\alpha _{\text {M}}$	$\nicefrac {B\dot{H}}{H^{2}}$	0
MSG/Palatini *f*(*R*) (Carroll et al. 2006; Vollick 2003)	$-\nicefrac {3}{2}\alpha _{\text {M}}^{2}$	$-\alpha _{{\text {M}}}$	$\nicefrac {2\dot{\phi }}{H}$	0
*f*(*G*) (Carroll et al. 2005; De Felice et al. 2010a)	0	$\frac{-2H\dot{\xi }}{M^{2}+H\dot{\xi }}$	$\frac{\dot{H}\dot{\xi }+H\ddot{\xi }}{H\left( M^{2}+H\dot{\xi }\right) }$	$\frac{\ddot{\xi }-H\dot{\xi }}{M^{2}+H\dot{\xi }}$

Simple dark energy models are described mostly by one or two Functions while covariant Galileons and *f*(*G*) require three. Reproduced with permission from Table 1 of Bellini and Sawicki (2014), copyright by IOP

*Other scalar–tensor theories*

An interesting scalar–tensor theory is that of Mimetic gravity that was originally proposed as mimetic dark matter in Chamseddine and Mukhanov (2013), see specialized review (Sebastiani et al. 2016). It was extended to produce inflation and late-time cosmic acceleration as well as to address cosmological or astrophysical singularities (Chamseddine et al. 2014; Chamseddine and Mukhanov 2017a, b; Ben Achour et al. 2017). The theory and its extensions can be constructed from the action, e.g., Langlois et al. (2018)

156 $$\begin{aligned} S[\tilde{g}_{\alpha \beta },\phi ] = \int d^4x \, \sqrt{ -g} \, {\mathcal {L}}(\phi ,\partial _\alpha \phi , \nabla _{\alpha }\!\nabla _\beta \phi \, ; g_{\alpha \beta }) \, , \end{aligned}$$

where the variation must be taken with respect to scalar field $\phi$ and the auxiliary metric $\tilde{g}_{\alpha \beta }$ which is related to the physical metric by a non-invertible disformal transformation,

157 $$\begin{aligned} g_{\alpha \beta } \; = \; {\tilde{A}}(\phi ,\tilde{X}) \, \tilde{g}_{\alpha \beta } \, + \, {\tilde{B}}(\phi ,{\tilde{X}})\, \partial _\alpha \phi \, \partial _\beta \phi \, ,\qquad \tilde{X} \equiv \tilde{g}^{\alpha \beta }\partial _\alpha \phi \, \partial _\beta \phi . \end{aligned}$$

The original mimetic dark matter theory had the Einstein–Hilbert term for $g_{\alpha \beta }$ as Lagrangian so in (156) it would depend only on $g_{\alpha \beta }$ and not on $\phi$ explicitly. We refer the reader to the review Sebastiani et al. (2016) for various formulations and discussions.

Dutta et al. (2018) performed a dynamical analysis of the theory showing that Mimetic Gravity can have successive radiation and matter dominated epochs followed by an accelerating phase. Interestingly, the dark matter and dark energy parameter have the same order of magnitude thus addressing the cosmic coincidence problem. These and other features were also stressed in Chamseddine et al. (2014) and references therein. Mirzagholi and Vikman (2015) introduced a novel simple mechanism to produce mimetic DM during radiation epoch. Perhaps the most interesting overall feature of Mimetic Gravity is that of a possible unified scenario for inflation, dark matter and dark energy.

However, a very recent study Langlois et al. (2018) showed that mimetic gravity theories can be viewed/formulated as degenerate higher-order scalar theories (DHOST) (Langlois and Noui 2016a) with an extra local gauge symmetry. They study linear perturbations about a homogeneous and isotropic background for all mimetic theories and find that they have either gradient instabilities or an Ostrogradsky type of instability in the scalar sector coupled to matter. The matter they included was in the form of k-essence scalar field. It will be interesting to see further development on this particular point and if there are ways around it in this unifying scenario of the dark sector.

Another interesting scalar–tensor theory is the ghost condensation model as proposed by Hamed et al. (2004). A scalar ghost field is added but the theory is kept stable because the terms in the action push the kinetic terms to a fixed condensation value avoiding instability. The theory has spontaneous breaking of Lorentz invariance. In such a theory, the ghost condensate field plays a role in the gravitational sector that is similar to that of the Higgs field in particle physics. That is gravitational fields propagating through the ghost condensate scalar field acquire a mass just like particles acquire mass while propagating through the Higgs field. The ghost condensate field fills space in the universe and is equivalent to a fluid with the same equation of state, $w=-1$, as a cosmological constant, and thus can drive the observed cosmic acceleration. But, such a ghost condensate fluid has physical scalar excitations and can be described as an effective field theory. The theory has interesting features such as attractive or repulsive gravity and has been used for problems in inflation, dark matter and cosmic acceleration (Hamed et al. 2004). The models lead to an interesting cosmological phenomenology including Friedmann equations that can be fit to observations and scalar perturbations that lead to a growth equation with additional terms that can be compared to large scale structure observations (Mukohyama 2006; Krause and Ng 2006).

Charmousis et al. (2012) proposed what they called the $F_{ab}$ four scalar–tensor theory and its cosmology in Copeland et al. (2012). Interestingly, this theory proposed a self-tuning mechanism that screens the contribution of the cosmological constant to curvature through phase transition. The name is given because the theory is based on 4 specific terms from the Horndeski action they call Fab Four (in analogy with the Beatles, they named the terms as John, Paul, George, and Ringo). The fabulous aspect is the self-tuning screening of the cosmological constant with a way around Weinberg’s no-go theorem (Weinberg 1989) by allowing the scalar field to break Poincaré invariance on the self-tuning vacua. However, it was argued in Appleby et al. (2012) and Linder (2013) that such models fail to provide a viable cosmic evolution for the whole cosmic history. Furthermore, these models have been ruled out by the requirement of $c_T=c$ from the neutron star merger event GW170817/GRB170817A.

Afshordi et al. (2007a, b) introduced a scalar–tensor model they call Cuscuton that is based on the infinite sound speed limit of k-essence model fluid (Armendáriz-Picón et al. 1999). k-essence is a model where the late-time acceleration is caused by the kinetic energy of the scalar field and not its potential energy as is the case in quintessence dark energy models (this the special case of Eq. (147) where only the second is present and the function *K* depends only on *X* there). k-essence field has perturbations which cause a change in the GR Poisson Eq. (86) (i.e., $Q(k,z)\ne 0$) but no gravitational slip, i.e., $\eta (k,z)=1$. Afshordi et al. (2007a) show that Cuscuton model is causal and perturbations do not introduce any additional dynamical degree of freedom but just obey only a constraint equation. They state that the model can be viewed as an effective modification of gravity on large scales. They also remark that this is the only modification of Einstein gravity that does not introduce any additional degrees of freedom and is not conformally equivalent to GR. They studied two models with specific potentials finding that one can mimic $\varLambda$CDM expansion history but has some early time departure from it. The second model has an expansion history similar to that of DGP (see Sect. 7.5.2) but is consistent with ISW effect from WMAP data. Cuscuton modes have a free potential and constraining them using observations will depend on the choice of such a potential.

Finally, it is worth including a different type of scalar–tensor theory known as the Einstein–Cartan–Sciama–Kibble theory. It constitutes an interesting development in gravity theories in which the torsion tensor is not vanishing and the affine connection is not symmetrical (Cartan 1922b, 1923, 1924; Sciama 1962, 1964; Kibble 1961). The torsion is related to the angular momentum (spin) of matter and the theory differs from GR only when the spin effects are important. Hehl and Von Der Heyde (1973) evaluated that for electron the density that will make the spin effect relevant is $\sim 13^{38}\,\mathrm{Kg/m}^{3}$ and $\sim 13^{45}\,\mathrm{Kg/m}^{3}$ for neutrons. These high densities can exist in the dense early universe but not any later during cosmic evolution. We refer the reader to the reviews (Hehl et al. 1974; Trautman 2006; Desai and Poplawski 2016).

#### Extra vector field(s)

*Illustrative example: Generalized Einstein-Aether theories*

In this theory, a unit timelike vector is added to the tensor metric. The vector field provides a preferred reference frame and constitutes an Aether-like field making the violation of Lorentz invariance. Gasperini (1987) first revived the idea of an Aether-like theory with the use of a scalar field and a preferred reference frame. Kostelecky and Samuel (1989) developed framework for spontaneous Lorentz-symmetry breaking in higher dimensions that served for many purposes later. Jacobson and Mattingly (2001, 2004) and Eling et al. (2004) followed a decade later by proposing a theory where in addition to the metric, a unit timelike vector field is added to the theory providing a direction of time and a preferred frame breaking Lorentz invariance under boosts. This was then generalized further by Zlosnik et al. (2007, 2006). We outline some aspects of the field equations and cosmology in this theory following Zlosnik et al. (2007, 2006) and Meng and Du (2012).

The action for Generalized Einstein-Aether theory is given by

158 $$\begin{aligned} S=\int d^{4}x \sqrt{-g} \left[ \frac{R}{16 \pi G}+{\mathcal {L}}_{A}+{\mathcal {L}}_{M}\right] , \end{aligned}$$

where the additional ${\mathcal {L}}_{A}$ term is the Lagrangian for the vector field, $A^\alpha$ given by Zlosnik et al. (2007, 2006):

159 $$\begin{aligned} {\mathcal {L}}_{A}= & {} \frac{M^{2}}{16 \pi G} {\mathcal {F}}({\mathcal {K}})+\frac{1}{16 \pi G} \lambda (A^{\alpha }A_{\alpha }+1)\nonumber \\ {\mathcal {K}}= & {} M^{-2} {\mathcal {K}}^{\alpha \beta }_{\phantom {\alpha \beta } \gamma \sigma } \nabla _{\alpha }A^{\gamma }\nabla _{\beta }A^{\sigma }\nonumber \\ {\mathcal {K}}^{\alpha \beta }_{\phantom {\alpha \beta } \gamma \sigma }= & {} c_{1}g^{\alpha \beta }g_{\gamma \sigma }+c_{2}\delta ^{\alpha }_{\gamma }\delta ^{\beta }_{\sigma }+ c_{3}\delta ^{\alpha }_{\sigma }\delta ^{\beta }_{\gamma }-c_{4} A^{\alpha }A^{\beta }g_{\gamma \sigma }, \end{aligned}$$

where $c_{i}$ are dimensionless constants and *M* is a coupling constant with mass dimension and typical scale value of the order of $H_0$ for cosmological purposes. $\lambda$ is a Lagrange multiplier to ensure the vector field is unit time-like, i.e., $A^{\alpha }A_{\alpha }=-1$. The ${\mathcal {F}}({\mathcal {K}})$ is a free function. For the case of linear Einstein-Aether theory it is simply equal to ${\mathcal {K}}$.

Variation of the action (158) with respect to $g^{\alpha \beta }$ and $A^{\beta }$ respectively gives

160 $$\begin{aligned} G_{\alpha \beta }= & {} \tilde{T}_{\alpha \beta }+8 \pi G T^{\mathrm{matter}}_{\alpha \beta } \end{aligned}$$

161 $$\begin{aligned} \nabla _{\alpha }({\mathcal {F}}' J^{\alpha }_{\phantom {\alpha }\beta })= & {} 2\lambda A_{\beta }, \end{aligned}$$

where $\tilde{T}_{\alpha \beta }$ is the energy-momentum tensor for the vector field, ${\mathcal {F}}'=\frac{d{\mathcal {F}}}{d{\mathcal {K}}}$, and $J^{\alpha }_{\phantom {\alpha }\sigma }=2 {\mathcal {K}}^{\alpha \beta }_{\phantom {\alpha \beta } \sigma \gamma }\nabla _{\beta }A^{\gamma }$. For ${\mathcal {K}}^{\alpha \beta }_{\phantom {\alpha \beta } \gamma \sigma }$ given by (159), $\tilde{T}_{\alpha \beta }$ is given by Zlosnik et al. (2007, 2006)

162 $$\begin{aligned} \tilde{T}_{\alpha \beta }=\frac{1}{2}\nabla _{\sigma } \left[ {\mathcal {F}}' \left( J_{(\alpha }^{\phantom {\alpha }\sigma }A_{\beta )}-J^{\sigma }_{\phantom {\sigma }(\alpha }A_{\beta )}-J_{(\alpha \beta )}A^{\sigma }\right) \right] -{\mathcal {F}}' Y_{(\alpha \beta )}+\frac{1}{2}g_{\alpha \beta }M^2{\mathcal {F}}+\lambda A_{\alpha } A_{\beta }, \end{aligned}$$

where the $$_{(\dots )}$$ denotes symmetry with respect to the indices. $$Y_{\alpha \beta }$$ is given for the particular choice of (159) (but setting $$c_4=0)$$ by

163 $$\begin{aligned} Y_{\alpha \beta }=-c_{1}\left[ (\nabla _{\nu }A_{\alpha })(\nabla ^{\nu }A_{\beta })- (\nabla _{\alpha }A_{\nu })(\nabla _{\beta }A^{\nu })\right] . \end{aligned}$$

Next, we outline some aspects of the cosmological evolution in the theory. We consider the general FLRW metric (5), the unit time-like vector

164 $$\begin{aligned} A^{\alpha }=(1,0,0,0). \end{aligned}$$

For the matter source, we consider a perfect fluid with velocity field $u^{\alpha }$ and energy momentum tensor given by

165 $$\begin{aligned} T^{\mathrm{matter}}_{\alpha \beta }=\rho u_{\alpha }u_{\beta }+p (u_{\alpha }u_{\beta }+g_{ \alpha \beta }). \end{aligned}$$

The results are as follows (Zlosnik et al. 2007):

166 $$\begin{aligned} \nabla _\beta A^{\beta }= & {} 3 H \nonumber \\ {\mathcal {K}}= & {} 3\alpha \frac{H^2}{M^2}, \end{aligned}$$

where $\alpha \equiv c_{1}+3c_{2}+c_{3}$ and $H\equiv \dot{a}/a$ is the usual Hubble parameter. As shown in Zlosnik et al. (2007, 2006), the energy momentum tensor, (162), also takes the form of a perfect fluid with effective state variables given by

167 $$\begin{aligned} {\bar{\rho }}_{A}=3\alpha H^2\left( {\mathcal {F}}'-\frac{\mathcal {F}}{2 \mathcal {K}}\right) \end{aligned}$$

and

168 $$\begin{aligned} \bar{p}_{A}=3\alpha H^2\left( -\frac{2}{3}{\mathcal {F}}'+\frac{\mathcal {F}}{2\mathcal {K}}\right) -\alpha \dot{ \mathcal {F}}'H-\alpha {\mathcal {F}}'\frac{\ddot{a}}{a}, \end{aligned}$$

satisfying the energy conservation equation $\dot{\rho }_A+3H(\rho _A+p_{A})=0$.

Next, the field equations give the modified Friedmann equations (Zlosnik et al. 2007, 2006)

169 $$\begin{aligned} \left( 1-\alpha {\mathcal {F}}'+\frac{1}{2} \frac{\alpha {\mathcal {F}}}{\mathcal {K}}\right) H^2+\frac{k}{a^2}= & {} \frac{ 8\pi G}{3}{\bar{\rho }} \end{aligned}$$

170 $$\begin{aligned} \frac{d}{dt}(-2H+\alpha {\mathcal {F}}'H)+\frac{2k}{a^2}= & {} 8\pi G({\bar{\rho }}+\bar{p}). \end{aligned}$$

We can see that additional terms are present from the function ${\mathcal {F}}({\mathcal {K}})$ and its derivatives that can be encapsulated to play the role of an effective cosmological constant due to the presence of the Aether field vector. The theory also contains a modified effective gravitational constant. Specific examples can be found in Zlosnik et al. (2006), Zuntz et al. (2010) and Lim (2005) where specific choices of ${\mathcal {F}}({\mathcal {K}})=\gamma (-{\mathcal {K}})^n$ can lead to late time acceleration with $n=0$ corresponding to a $\varLambda \mathrm {CDM}$ model. Meng and Du (2012) proposed other models leading to other effective dark energy models. Battye et al. (2017b) also developed a designer approach to generalized Einstein-Aether to mimic any *w*CDM background. Instead of specifying a specific ${\mathcal {F}}({\mathcal {K}})$ function, other parameters such as *w* and $\varOmega _{\mathrm{de}}$ can be specified to find a functional form for ${\mathcal {F}}({\mathcal {K}})$. This essentially amounts to solving the generalized Friedmann equations (169) and (170).

Linear perturbations for generalized Einstein-Aether theory have been worked out in, e.g., Zuntz et al. (2010), Armendáriz-Picón et al. (2010) and Battye et al. (2017b) taking into account perturbations of the metric and the vector field. This gives modified Poisson equations where the vector field leads to a different source for the Poisson equations and also induces a slip between the two gravitational potentials. This provides a means to test the models using large scale structure as well as CMB and to distinguish them from the $\varLambda \mathrm {CDM}$ model.

*Other vector–tensor theories*

Some of the first vector–tensor theories were those of Will and Nordtvedt (1972) where the authors derived and explored the models within an extended PPN formalism. Other vector–tensor theories include the recent generalized Proca theories where the vector field is promoted to a Proca massive vector field with ghost free models and accelerating cosmologies (Heisenberg 2014; De Felice et al. 2016a, c; Beltrán Jiménez and Heisenberg 2017; Heisenberg et al. 2016). A number of other developments on vector–tensor theories can be found in Beltrán Jiménez et al. (2013, 2016a, 2017), Heisenberg (2014), Tasinato (2014), Allys et al. (2016a), Beltrán Jiménez and Heisenberg (2016, 2017), Heisenberg et al. (2016), Kimura et al. (2017), De Felice et al. (2016a, b), Emami et al. (2017), Hull et al. (2014, 2016), Allys et al. (2016b) and Nakamura et al. (2017). A concise review on generalized Proca theories can be found in Heisenberg (2017).

#### Extra vector and scalar fields

*Illustrative example: TeVeS theory*

A Tensor–Vector–Scalar theory known as TeVeS in the literature was introduced by Bekenstein in Bekenstein (2004) as a relativistic generalization of Modified-Newtonian-Dynamics (MOND) theory (Milgrom 1983a, b). MOND and TeVeS both aim at addressing some observations such as the flat rotation curves of galaxies without the need for Dark Matter. MOND has been criticized for not fitting other astrophysical observations but see discussion and debate in for example Scott et al. (2001), Foreman and Scott (2012) and McGaugh (2011).

TEVES provides a more complex theory where the additional vector field could for example cause a stronger gravitational infall of baryons during the early universe epoch and thus alleviates the need for dark matter to create strong gravitational potential wells, see e.g., Dodelson and Liguori (2006).

The TeVeS action is commonly written in two frames and we follow that here (a single frame formulation can be found in Zlosnik et al. 2006). The gravitational fields are written in the Einstein frame (sometime also referred to as the Bekenstein frame for this specific theory) while the matter fields are written in the frame of the physical metric, $g_{\mu \nu }$. The three gravitational fields of the theory are the Bekenstein metric tensor, $\tilde{g}_{\mu \nu }$, the Sanders vector field, $A_\mu$, and the scalar field, $\phi$. The matter metric is related to the Bekenstein metric by  Bekenstein (1993)

171 $$\begin{aligned} g_{\mu \nu } = e^{-2\phi }\tilde{g}_{\mu \nu } - 2\sinh (2\phi )A_\mu A_\nu . \end{aligned}$$

The TeVeS theory is defined by the sum of the following four actions:

1. For the metric field,
  172 $$\begin{aligned} S_{\tilde{g}} = \frac{1}{16\pi G}\int d^4x \; \sqrt{-\tilde{g}}\; \tilde{R}, \end{aligned}$$
  where *G* is the bare gravitational constant related to Newton’s constant, $G_N$, via the solution to the quasistatic spherically symmetric solution of the TeVeS field equations (Bekenstein 1993). See also Clifton et al. (2012) for a concise discussion.
2. For the vector field,
  173 $$\begin{aligned} S_A = -\frac{1}{32\pi G} \int \mathrm {d}^4 x\sqrt{-\tilde{g}} [K_B F_{\mu \nu } F^{\mu \nu } -2\lambda (A_{\mu } A^{\mu } + 1)], \end{aligned}$$
  where $F_{\mu \nu } \equiv 2\tilde{\nabla }_{[\mu } A_{\nu ]}$, $F^{\mu \nu } =\tilde{g}^{\mu \alpha } \tilde{g}^{\nu \beta } F_{\alpha \beta }$, $A^{\mu } = \tilde{g}^{\mu \nu }A_{\nu }$, $\lambda$ is a Lagrange multiplier to ensure $\tilde{g}^{\mu \nu } A_{\mu } A_{\nu } = -1$, and $K_B$ is a dimensionless constant related to the vector field. $K_B$ constitutes one of the additional parameters of the TeVeS models.
3. For the scalar field,
  174 $$\begin{aligned} S_{\phi } = -\frac{1}{16\pi G} \int \mathrm {d}^4 x \sqrt{-\tilde{g}} [\mu (\tilde{g}^{\mu \nu } - A^{\mu }A^{\nu }) \tilde{\nabla }_{\mu }\phi \tilde{\nabla }_{\nu }\phi + V(\mu )], \end{aligned}$$
  where $\mu$ is a dimensionless non-dynamical scalar field. $V(\mu )$ is a free function which typically depends on a scale $l_B$ (this is a second parameter of the TeVeS model). The action for the scalar field is constructed such that TeVeS theory has a MOND non-relativistic limit, under some conditions and for some specific forms of the function $V(\mu )$. For example, the function in Bekenstein (2004) is given by
  175 $$\begin{aligned} \frac{\mathrm {d}V}{\mathrm {d}\mu } = -\frac{3}{32\pi l_B^2 \mu _0^2} \frac{\mu ^2(\mu -2\mu _0)^2}{\mu _0-\mu }, \end{aligned}$$
  where $\mu _0$ is a dimensionless constant (the third parameter of the TeVeS model) and leads to a MOND limit. Similarly, other more general functions leading to MOND can found in Bourliot et al. (2007), Sanders (2006) and Angus et al. (2006).
4. For the matter fields, $\psi _{\mathrm{m}}$,
  176 $$\begin{aligned} S_m = \int \mathrm {d}^4 x \sqrt{-g} \mathcal {L}[g, \psi _{\mathrm{m}}, \partial \psi _{\mathrm{m}}]. \end{aligned}$$
  where here the matter frame metric is used. We note that if arbitrary matter fields (including for instance fermions) are allowed and a Lagrangian that can depend on the matter field derivative, then by covariance that Lagrangian would also need to involve the derivative of the metric as well.

The corresponding field equations for the metric tensor, the vector field and the scalar field are given respectively by:

177 $$\begin{aligned} \tilde{G}_{\mu \nu }= & {} 8\pi G\left[ T_{\mu \nu } + 2(1 - e^{-4\phi })A^{\alpha }T_{\alpha (\mu } A_{\nu )}\right] \nonumber \\&+\, \mu \left[ \tilde{\nabla }_\mu \phi \tilde{\nabla }_\nu \phi - 2 A^\alpha \tilde{\nabla }_\alpha \phi \; A_{(\mu }\tilde{\nabla }_{\nu )}\phi \right] + \frac{1}{2}\left( \mu V' - V\right) \tilde{g}_{\mu \nu }\nonumber \\&+\, K\left[ F^\alpha _{\;\;\mu } F_{\alpha \nu } - \frac{1}{4} F^{\alpha \beta } F_{\alpha \beta } \tilde{g}_{\mu \nu }\right] - \lambda A_\mu A_\nu , \end{aligned}$$

178 $$\begin{aligned} K \tilde{\nabla }_\alpha F^\alpha _{\;\;\mu }= & {} -\lambda A_\mu - \mu A^\nu \tilde{\nabla }_\nu \phi \tilde{\nabla }_\mu \phi + 8\pi G (1 - e^{-4\phi })A^{\nu }T_{\nu \mu } , \end{aligned}$$

and

179 $$\begin{aligned} \tilde{\nabla }_\mu \left[ \mu \hat{g}^{\mu \nu } \tilde{\nabla }_\nu \phi \right]= & {} 8\pi G e^{-2\phi }\left[ g^{\mu \nu } + 2e^{-2\phi } A^{\mu }A^\nu \right] T_{\mu \nu }. \end{aligned}$$

In addition to the field equations, the theory has two constraints. The first is the usual timelike constraint on the vector field, i.e., $A^\alpha A_\alpha =-1$. This is obtained by varying the action with respect to the Lagrange multiplier, $\lambda$. The second constraint fixes the non-dynamical field ,$\mu$, in terms of the other fields in the theory. It derives from varying the action with respect $\mu$. The above field equations and constraints are all used in what follows.

Next, we proceed to describe some aspects of the cosmology of TeVeS. Some studies based on the homogeneous and isotropic FLRW metric can be found in Bekenstein (2004), Diaz-Rivera et al. (2006), Bourliot et al. (2007), Ferreira et al. (2008), Zhao (2007) and Hao and Akhoury (2009). We follow here (Xu et al. 2015; Clifton et al. 2012) and give some key cosmological equations for a spatially flat FLRW background. The metric in conformal time and the matter frame reads

180 $$\begin{aligned} \mathrm {d}s^2 = a^2(\tau ) (-\mathrm {d}\tau ^2 + \mathrm {d}r^2), \end{aligned}$$

while in the Einstein frame,

181 $$\begin{aligned} \mathrm {d}\tilde{s}^2 = b^2(\tau ) (-e^{-4\phi }\mathrm {d}{\tau }^2 + \mathrm {d}{r}^2 ). \end{aligned}$$

The two scale factors *a* and *b* are related by the disformal relation $a=be^{-\phi }$.

The Friedmann equation in the Einstein frame is given by Skordis (2006):

182 $$\begin{aligned} 3\left( \frac{b^\prime }{b}\right) ^2=a^2 \bigg [ \frac{1}{2}e^{-2\phi }\left( \mu \frac{dV}{d\mu }+V\right) +8\pi G e^{-4\phi }{\bar{\rho }} \bigg ], \end{aligned}$$

where ${\bar{\rho }}$ is the matter energy density. It should be noted that the vector field does not contribute to the dynamic of an FLRW background which is then completely described by the scalar field evolution equation

183 $$\begin{aligned} \phi ^{\prime \prime } = \phi ^\prime \bigg ( \frac{a^\prime }{a}-\phi ^\prime \bigg ) - \frac{1}{U} \bigg [ 3\mu \frac{b^\prime }{b}\phi ^\prime + 4\pi Ga^2e^{-4\phi }({\bar{\rho }}+3\bar{P}) \bigg ], \end{aligned}$$

where $U \equiv \mu +2\frac{dV}{d\mu }/\frac{d^2 V}{d\mu ^2}$ and $\bar{P}$ denotes the pressure from the matter sources (but not the scalar field).

In the matter frame, the physical Hubble parameter is defined as usual as $H \equiv \frac{a^\prime }{a^2}$. The corresponding equivalent of the Friedmann equation is then given by, see e.g. Skordis (2006)

184 $$\begin{aligned} 3H^2 = 8\pi G_{\mathrm {eff}} ({\bar{\rho }} + {\bar{\rho }}_{\phi }), \end{aligned}$$

where the effective gravitational constant is given by

185 $$\begin{aligned} G_{\mathrm {eff}} = G \frac{e^{-4\phi }}{\left( 1 + \frac{\mathrm {d}\phi }{\mathrm {d}\ln {a}}\right) ^2}, \end{aligned}$$

the energy density of the scalar field is given by

186 $$\begin{aligned} {\bar{\rho }}_{\phi } = \frac{1}{16\pi G} e^{2\phi } \left( \mu \frac{dV}{d\mu } + V\right) \end{aligned}$$

and its pressure by

187 $$\begin{aligned} \bar{P}_\phi = \frac{e^{2\phi }}{16\pi G}\left( \mu \frac{dV}{d\mu } - V \right) . \end{aligned}$$

An effective density fraction can be defined as $\varOmega _{\phi } = \frac{{\bar{\rho }}_{\phi }}{{\bar{\rho }} + {\bar{\rho }}_{\phi }}$. When the function *V* takes the form of Eq. (175), the scalar field energy density is found to track the matter energy density (Dodelson and Liguori 2006; Skordis et al. 2006; Skordis 2009) with

188 $$\begin{aligned} \varOmega _{\phi } = \frac{(1+3w)^2}{6(1-w)^2 \mu _0}, \end{aligned}$$

where *w* is the equation of state of the background matter field and the scalar field contribution is always subdominant since $\mu _0$ is of the order of $10^2$.

Adding a constant to the free function *V*, is equivalent to adding a cosmological constant to the effective Friedmann equation (184) and thus producing cosmic acceleration.

Finally, a concise description of the perturbation equations in TeVeS can be found in, e.g., Skordis (2006, 2008) and Skordis et al. (2006) and we refer the reader to those. Mainly, the matter overdensity and velocity field keep the same evolution equations as in GR but are supplemented by perturbation equations for the scalar and vector fields (Skordis 2006, 2008; Skordis et al. 2006). However, an important difference exists in the processes of growth of structures between $\varLambda$CDM and TeVeS. In $\varLambda$CDM, baryons fall after decoupling into deeper potential wells caused by dark matter. But in TeVeS, it is rather the rapidly growing perturbations of the vector field that drives the growth of perturbations. Such a difference in the processes leads to differences in the growth rate of baryon perturbations as well as the amplitude of their peculiar velocity power spectrum, see Skordis et al. (2006), Dodelson and Liguori (2006) and Xu et al. (2015). Unlike the $\varLambda$CDM model, the growth rate in TeVeS is scale dependent which provides a further test to constrain the models (Skordis 2006, 2008; Skordis et al. 2006; Xu et al. 2015).

TeVeS shows how adding a scalar and vector field to the metric tensor can add further complexity and sophistication to gravity, However, the theory has been recently found to be in tension with latest large scale structure and CMB data sets, e.g., Reyes et al. (2010) and Xu et al. (2015), although often disputed by its proposers, e.g., Bekenstein and Sanders (2012) and Milgrom (2017).

*Other scalar–vector–tensor theories*

Moffat (2006) proposed a scalar–tensor–vector gravity (STVG) theory, also referred to as MOG that allows the gravitational constant *G*, a vector field with coupling $\omega$, and the vector field mass $\mu$ to vary in space and time. This theory has modified equations of motion for test particles that have a modified gravitational acceleration law that can fit rotation curves of galaxies and also data from clusters of galaxies without the need for dark matter. The theory is consistent with solar system tests of gravity and is ghost free. Gravitational waves and electromagnetic waves both travel on null geodesics of the metric with equal speeds so the theory is not ruled out by the GW event GW170817 and its electromagnetic counterpart GRB170817A (Green et al. 2017). The theory is reported in Moffat and Toth (2011) to fit gravitational lensing of observations and to be consistent with some cosmological observations with no need of dark matter, however oscillations of the matter power spectrum in MOG are not suppressed (Moffat and Toth 2011). It remains to be tested against full LSS data or CMB data.

#### Extra tensor fields

Last but not least, it turned out that adding an extra metric tensor to GR can be a very lucrative extension. For example, a first accomplishment in doing so was to achieve a gravity theory where the graviton has an effective mass or a resonance (massive gravity) (Fierz and Pauli 1939; de Rham and Gabadadze 2010; de Rham et al. 2011; Hassan and Rosen 2012a). Moreover, some of such theories can provide self-accelerating cosmological models with no need for a cosmological constant. These massive gravity theories change the coupling between curvature of spacetime and its source and the idea behind generating cosmic acceleration is that gravity is weakened at the graviton’s mass Compton wavelength which is comparable to Hubble scales.

On more point which is worth highlighting is that such massive gravity theories allow for proposals of degravitation mechanisms of the cosmological constant (Arkani-Hamed et al. 2002; Dvali et al. 2003b). The idea is that the massive graviton acts as a high-pass filter with filter scale, *L*, set by the inverse of the mass of the graviton. Sources with wavelengths $\ll L$ pass the filter and gravitate normally. However, sources with wavelengths $\gg L$, like the cosmological constant, are filtered out leading to their degravitation (Dvali et al. 2007). This and other related ideas are very interesting but unfortunately so far there is no realistic realization of such a degravitation mechanism.

We outline below some selected aspects of two illustrative examples of these tensor–tensor or bimetric theories, list some other models, and refer the reader to the specialized reviews (de Rham 2014; Hinterbichler 2017; Clifton et al. 2012).

*Illustrative example 1: massive gravity*

The idea goes back at least to the early attempts of Fierz and Pauli (Fierz and Pauli 1939) in simply deriving a theory of gravity with a massive graviton. Fierz and Pauli considered a non-dynamical background flat metric $\eta _{\alpha \beta }$ (Minkowski) and a dynamical linear perturbation, $h_{\alpha \beta }$ resulting in the dynamical metric

189 $$\begin{aligned} g_{\alpha \beta }=\eta _{\alpha \beta }+h_{\alpha \beta }. \end{aligned}$$

They derived and added a (PF)-term at linear order to the Einstein–Hilbert action that generates the massive graviton as follows (Fierz and Pauli 1939)

190 $$\begin{aligned} {\mathcal {L}}_{FP}=m^2 \left[ h^{\mu \nu }h_{\mu \nu }-(\eta ^{\mu \nu }h_{\mu \nu })^2\right] , \end{aligned}$$

where *m* is the mass parameter. They showed that this term is the only linear-order term that leads to no-ghost mode at this order. Therefore, the Fierz–Pauli is the unique consistent linear theory of massive gravity.

However, at nonlinear order, the story is different. The action with an FP term can be generalized to nonlinear order as (Boulware and Deser 1972)

191 $$\begin{aligned} S=\frac{1}{16\pi G} \int d^4x \sqrt{-g}R(g)+\frac{m^2}{4} \sqrt{-g} \left[ g^{\mu \nu }g^{\alpha \beta }-g^{\mu \alpha } g^{\nu \beta }\right] h_{\mu \nu } h_{\alpha \beta }. \end{aligned}$$

It was shown by Boulware and Deser (1972) that the Fierz–Pauli theory at nonlinear order acquires a scalar ghost mode and is thus unstable. Another problem with the theory is known as the van Dam, Veltman, and Zakharov (vDVZ) discontinuity, see van Dam and Veltman (1970) and Zakharov (1970). Namely, that solutions to the theory cannot be continuously connected to their analog GR solutions when the graviton mass is taken to the zero limit, as one would naively expected from the action. To explain, let’s consider the spherically symmetric vacuum solution representing the gravitational field around a concentric mass such as the Sun. Then, taking the limit of the graviton mass going to zero does not give back a solution analog to the GR Schwarzschild solution and is thus inconsistent with local observations such as the deflection angle of light, precession of planets, or light travel time delays.

To deal with these two problems, some possible solutions were proposed in Vainshtein (1972) and Arkani-Hamed et al. (2003) where one could solve two problems with one stratagem. First, in order to deal with ghost modes appearing at higher orders, one would introduce tuned higher order interaction terms that would remove the ghost terms order by order. Second, Vainshtein (1972) suggested his mechanism (see Sect. 8.3) where such higher order interaction terms would serve at small scales to shield additional-field interactions and lead to observations indistinguishable from GR.

A *tour de force* came from de Rham, Gabadadze and Tolley (dRGT) (de Rham and Gabadadze 2010; de Rham et al. 2011) who succeeded in generalizing Fierz–Pauli theory and formulating a stable massive gravity. For that, they considered $g_{\mu \nu }$ and $f_{\mu \nu }$ as the dynamical and non-dynamical metrics, respectively, and wrote the action:

192 $$\begin{aligned} \mathcal {S} = \int d^4 x \frac{\sqrt{-g}}{16 \pi G} R(g)- m^2\int d^4 x \frac{\sqrt{-g}}{8 \pi G} \sum \limits _{n=0}^4 \beta _n e_n \left( \mathbb {X} \right) + \mathcal {S}_M(g_{\mu \nu }, \psi _{\mathrm{m}}),\qquad \end{aligned}$$

where the first part is the usual Hilbert–Einstein term and the third part is the matter action term, while the middle part gives the dRGT terms with $\beta _n$ as arbitrary constants and $e_n$ are functions defined by,

193 $$\begin{aligned} e_0\left( \mathbb {X}\right)= & {} 1, \nonumber \\ e_1\left( \mathbb {X}\right)= & {} \left[ \mathbb {X}\right] ,\nonumber \\ e_2\left( \mathbb {X}\right)= & {} \frac{1}{2} \left( \left[ \mathbb {X}\right] ^2 - \left[ \mathbb {X}^2\right] \right) , \nonumber \\ e_3\left( \mathbb {X}\right)= & {} \frac{1}{6} \left( \left[ \mathbb {X}\right] ^3 -3 \left[ \mathbb {X}\right] \left[ \mathbb {X}^2\right] + 2 \left[ \mathbb {X}^3\right] \right) , \nonumber \\ e_4\left( \mathbb {X}\right)= & {} \det \mathbb {X}, \end{aligned}$$

where $\mathbb {X} \equiv \sqrt{g^{\alpha \beta } f_{\beta \gamma }}$ and $\left[ \mathbb {X} \right]$ is its trace (i.e., $(\mathbb {X}^2)^\alpha \,_\gamma = g^{\alpha \beta } f_{\beta \gamma }$).

The equations of motion can be found in de Rham and Gabadadze (2010) and de Rham et al. (2011). Interestingly, massive gravity can have cosmological solutions that can self-accelerate, however, the cosmological solutions have to be Minkowski type open FLRW with strongly coupled perturbations making them not analyzable by standard methods. There are other cosmological solutions with well-behaved perturbations but they require non-isotropy or preferred directions making them cosmologically less attractive, see e.g., Hinterbichler (2017) and de Rham (2014). It was then realized soon after that it would be interesting to have the second metric to be a non-Minkowski and dynamical like an FLRW metric (Hassan and Rosen 2011, 2012a).

From a cosmological point of view, it turned out that adding a dynamical metric provides a richer phenomenology and the possibility to have stable and viable self-accelerating solutions (Akrami et al. 2015), although in this case there are also bounds and conditions that must hold to avoid further instabilities as we discuss in the next section (Könnig et al. 2014; Lagos and Ferreira 2014; Könnig 2015).

It is worth noting that most recently Heisenberg and Tsujikawa (2017) performed a thorough analysis of perturbations in massive gravity with SO(3) rotation invariance. The models violate Lorentz invariance and it was argued there that this makes it possible to avoid some problems in massive gravity. The models and their cosmology have been studied and reviewed in Dubovsky (2004), Dubovsky et al. (2005), Bebronne and Tinyakov (2007), Blas et al. (2009a), Domènech et al. (2017) and Comelli et al. (2014). It was shown in Dubovsky et al. (2005), Comelli et al. (2014) and Heisenberg and Tsujikawa (2017) healthy models can have late-time self-acceleration. Heisenberg and Tsujikawa (2017) worked out perturbations in and FRLW background and with a perfect fluid source. They found models that have no ghosts nor gradient instabilities for effective dark energy equation of state $w_{\mathrm{DE}} > -1$ and $w_{\mathrm{DE}} < -1$. They also derived expressions for the effective gravitational constant and the slip parameter. Implementation of this formalism into full CMB code and large scale structure will allow for the comparison of these models to current and future cosmological data.

*Illustrative example 2: Bimetric massive gravity or bigravity*

In addition to realizing a massive gravity theory, adding a second dynamical metric has been shown to provide stable self-accelerating cosmological solutions with no need for a dark energy component, see e.g. Hassan and Rosen (2011, 2012a, b), Koennig et al. (2014a, b) and Akrami et al. (2015). These theories have a branch of models that admit a limit in which the Planck mass associated to the second metric is small and any scalar instabilities can be pushed to very early times where they are not observable (Koennig et al. 2014a; Lagos and Ferreira 2014; Cusin et al. 2015a, b, 2016; Akrami et al. 2015; Schmidt-May and von Strauss 2016). Even if in this limit the background evolution becomes indistinguishable from that of the $\varLambda$CDM, Akrami et al. 2015 argue that it provides a technically natural value for the effective cosmological constant.

The action for bimetric massive gravity reads (Hassan and Rosen 2011, 2012a),

194 $$\begin{aligned} \mathcal {S}= & {} \int d^4 x \left( \frac{\sqrt{-g}}{16 \pi G} R(g) + \frac{\sqrt{-f}}{16 \pi G_f} R(f) \right) \nonumber \\&-\, m^2\int d^4 x \frac{\sqrt{-g}}{8 \pi G} \sum \limits _{n=0}^4 \beta _n e_n \left( \mathbb {X} \right) \nonumber \\&+\, \mathcal {S}_M(g_{\mu \nu }, \psi _{\mathrm{m}}), \end{aligned}$$

where here we note the additional action term with the Ricci scalar, *R*(*f*), built out of the second metric, *f*, compared to the action  (192). Variation of Eq. (194) with respect to $g_{\mu \nu }$ and $f_{\mu \nu }$ gives the field equations,

195 $$\begin{aligned}&G_{\mu \nu } + \sum \limits _{n=0}^3 (-1)^n\beta _n g_{\mu \lambda } (Y_n)^\lambda _\nu = \kappa ^2 T^M_{\mu \nu }, \end{aligned}$$

196 $$\begin{aligned}&F_{\mu \nu } +\sum \limits _{n=0}^3 (-1)^n\beta _{4-n} f_{\mu \lambda } (Y_n)^\lambda _\nu = 0, \end{aligned}$$

where $m^2$ has been absorbed into $\beta _n$, and $8\pi G_f$ was set to 1, following the notation of  Khosravi et al. (2012), Koennig et al. (2014b) and Geng et al. (2017). $G_{\mu \nu }$ and $F_{\mu \nu }$ are the Einstein tensors built from the metrics $g_{\mu \nu }$ and $f_{\mu \nu }$, respectively. $T^M_{\mu \nu }$ is the matter energy-momentum tensor and $(Y_n)^{\lambda }_\nu$ are matrices defined by

197 $$\begin{aligned} Y_0= & {} \mathbb {I}, \nonumber \\ Y_1= & {} \mathbb {X} -\mathbb {I} \left[ \mathbb {X}\right] ,\nonumber \\ Y_2= & {} \mathbb {X}^2 - \mathbb {X} \left[ \mathbb {X}\right] + \frac{1}{2} \mathbb {I}\left( \left[ \mathbb {X}\right] ^2 - \left[ \mathbb {X}^2\right] \right) , \nonumber \\ Y_3= & {} \mathbb {X}^3 - \mathbb {X}^2 \left[ \mathbb {X}\right] + \frac{1}{2} \mathbb {X} \left( \left[ \mathbb {X}\right] ^2 - \left[ \mathbb {X}^2\right] \right) - \frac{1}{6}\mathbb {I} \left( \left[ \mathbb {X}\right] ^3 - 3 \left[ \mathbb {X}\right] \left[ \mathbb {X}^2\right] + 2 \left[ \mathbb {X}^3\right] \right) .\nonumber \\ \end{aligned}$$

Next, the field equations are applied to the FLRW metrics in, e.g. Koennig et al. (2014a, b) and Geng et al. (2017),

198 $$\begin{aligned} ds^2= & {} g_{\mu \nu } dx^{\mu } dx^{\nu } = -dt^2 + {a(t)}^2 dx^i dx_i \end{aligned}$$

199 $$\begin{aligned} ds_f^2= & {} f_{\mu \nu } dx^{\mu } dx^{\nu } = -\frac{\dot{b}^2}{\dot{a}^2} dt^2 + b(t)^2 dx^i dx_i \end{aligned}$$

to obtain the Friedmann-like equations (Geng et al. 2017)

200 $$\begin{aligned} H^2= & {} \frac{1}{3} \left( {\bar{\rho }}_M + \beta _0 + 3 \beta _1 \frac{b}{a} + 3 \beta _2 \frac{b^2}{a^2} + \beta _3 \frac{b^3}{a^3} \right) , \end{aligned}$$

201 $$\begin{aligned} \dot{H}= & {} -\frac{1}{2} \left( {\bar{\rho }}_M + \bar{P}_M + \beta _1 \frac{b}{a}+2\beta _2\frac{b^2}{a^2}+\beta _3{b^3}{a^3}-\beta _1\frac{\dot{b}}{\dot{a}}-2\beta _2\frac{b}{a}\frac{\dot{b}}{\dot{a}}-\beta _3\frac{b^2}{a^2}\frac{\dot{b}}{\dot{a}} \right) ,\nonumber \\ \end{aligned}$$

for $$g_{\mu \nu }$$ and

202 $$\begin{aligned} H^2= & {} \frac{1}{3} \frac{a}{b} \left( \beta _1 + 3 \beta _2\frac{b}{a} + 3 \beta _3 \frac{b^2}{a^2} + \beta _4 \frac{b^3}{a^3} \right) , \end{aligned}$$

203 $$\begin{aligned} H^2+2\frac{H}{H_f}\frac{\ddot{a}}{a}= & {} \left( \beta _2+2\beta _3\frac{b}{a}+\beta _4\frac{b^2}{a^2}+\beta _1 \frac{\dot{a}}{\dot{b}}+2\beta _2\frac{b}{a} \frac{\dot{a}}{\dot{b}}+\beta _3\frac{b^2}{a^2}\frac{\dot{a}}{\dot{b}} \right) , \end{aligned}$$

for $f_{\mu \nu }$, where $H=(\dot{a}/a)$ while $H_{(f)}=(\dot{b}/b)$ is the Hubble constant of $f_{\mu \nu }$, $\rho _M = \rho _r + \rho _m$ is the energy density of the radiation and matter and ($P_M = P_r + P_m$) is the sum of their pressures. $\kappa ^2$ was set to 1. Note that the presence of $\dot{a}$ in (199) allows (202) and (203) to be written using *H*. See also Koennig et al. (2014a, b) where a compact encapsulation of these equations is given. As can be seen from Eqs. (202) and (203), and stressed in Koennig et al. (2014a, b), the background dynamics depend entirely on the Hubble parameter of the metric $g_{\mu \nu }$ and the ratio of the two scale factors.

The $\beta _0$ term represents a cosmological constant term. Koennig et al. (2014a) performed a stability analysis finding that the only single parameter models without instabilities at early times are models with $\beta _2$ or $\beta _4$. They found there are no self-accelerating models (i.e., $\beta _0=0$) with a viable background evolution and stable perturbations on the finite branch. For the infinite branch, they found only models with non-vanishing $\beta _1$ and $\beta _4$ are self-accelerating, viable and stable for all cosmic evolution and they focused their analysis on those models.

Geng et al. (2017), presented a minimum nontrivial case by setting $\beta _2 = \beta _3 = \beta _4 =0$ that we reproduce here for mere illustration purposes. Consequently, Eqs. (202) and (203) reduce to

204 $$\begin{aligned} \frac{b}{a} = \frac{ \beta _1}{3 H^2}, \qquad \mathrm {and} \qquad H_b \equiv \frac{H_f}{H} = 1 - 2 \frac{\dot{H}}{H^2}. \end{aligned}$$

The authors defined an effective energy density and pressure from (200) and (201)

205 $$\begin{aligned} \rho _{\mathrm{DE}}= & {} \beta _0 + 3 \beta _1 \frac{b}{a} = \rho _{\mathrm{DE}}^{(0)} \left( \bar{\beta }_0 + \bar{\beta }_1 \frac{H_0^2}{H^2} \right) , \end{aligned}$$

206 $$\begin{aligned} P_{\mathrm{DE}}= & {} - \beta _0 - \beta _1 \left( 2 \frac{b}{a} + \frac{\dot{b}}{\dot{a}} \right) = \rho _{\mathrm{DE}}^{(0)} \left[ -\bar{\beta }_0 + \bar{\beta }_1 \frac{H_0^2}{H^2} \left( \frac{2\dot{H}}{3H^2} - 1 \right) \right] , \end{aligned}$$

that satisfy the continuity equation, $\rho _{\mathrm{DE}} + 3 H \left( \rho _{\mathrm{DE}} + P_{\mathrm{DE}} \right) = 0$ and where they defined

207 $$\begin{aligned} \bar{\beta }_0 = \frac{ \beta _0 }{ \rho _{\mathrm{DE}}^{(0)}} \quad \mathrm {and} \quad \bar{\beta }_1 = \frac{\beta _1^2 }{ H_0^2 \rho _{\mathrm{DE}}^{(0)}}, \end{aligned}$$

with $\bar{\beta }_0 + \bar{\beta }_1 = 1$ and $\rho _{\mathrm{DE}}^{(0)}$ being the corresponding effective dark energy density at present. They noted that from Eqs. (205) and (206), $e_0 \left( \mathbb {X}\right)$ with the free parameter $\beta _0$ in the action plays the role of an effective cosmological constant. They also analyzed the evolution of the effective dark energy density and found that the model has a phantom-type equation of state, $w_{\mathrm{DE}} < -1$.

Bimetric massive gravity has branches that are not ruled out by current observations, see Sect. 9.4. The structure of the theory gives extra terms in the evolution equation that can be encapsulated as effective dark energy density and negative pressure thus producing late-time cosmic acceleration without the need for a cosmological constant. The models fit well background observational data and some growth (see Sect. 9.4) and constitute a competitor to the $\varLambda$CDM GR model. Comparison of these models to full CMB and large scale structure data is needed.

However, Könnig (2015), Lagos and Ferreira (2014), Koennig et al. (2014a) and Comelli et al. (2012) pointed out to some further instabilities that must to be avoided by requiring some conditions to hold. For example, Higuchi instability can occur in theories with massive spin-2 particles (here the massive graviton) where the mass must satisfy specific bounds in order to avoid modes with negative norm and the appearance of a Higuchi ghost (Higuchi 1987, 1989). Higuchi provided mass bounds for the de Sitter space while Fasiello and Tolley (2012) derived mass bounds for massive gravity in flat FLRW spacetimes, see also Woodard (2007). Higuchi instability and scalar gradient instabilities for cosmological solutions in massive gravity are discussed in Lagos and Ferreira (2014), Cusin et al. (2015a) and Könnig (2015). Könnig (2015) analyzed general models in singly coupled bimetric gravity around a FLRW background and found that all models that are not equivalent to $\varLambda \mathrm {CDM}$ suffer from either gradient or Higuchi instabilities.

Other self-accelerating solutions in massive gravity with inhomogeneous fiducial metric were discussed in Koyama et al. (2011), Gratia et al. (2012) and Khosravi et al. (2013). The physical metric in these solutions is an FLRW and can be flat, however, these solutions were shown to suffer from instabilities as recapitulated in Khosravi et al. (2013).

Perturbation and growth of structure equations for bigravity can be found in for example Koennig et al. (2014a), Kobayashi et al. (2016) and Lagos and Ferreira (2017). It was shown in Koennig et al. (2014a, b) that bimetric gravity has several classes of models with unstable linear perturbations. However, they also found that a particular class of models, named the infinite-branch, has a viable background evolution and stable linear perturbations. The infinite-branch refers simply to a specific evolution of the ratio of the scale factors *b* / *a* of the two metrics of the theory (Solomon et al. 2014; Koennig et al. 2014a, b). Further cosmological constraint studies have since focused on the infinite-branch models of bimetric massive gravity as we discuss those in Sect. 9.4. However, Könnig (2015) showed that the infinite branch suffers from Higuchi instability which compromises its viability. Detailed discussions about massive gravity and bigravity, their phenomenology and cosmology can be found in the following review papers de Rham (2014) and Schmidt-May and von Strauss (2016).

*Other tensor–tensor theories*

Models with an extra 2-rank tensor include Rosen’s theory (Rosen 1940, 1973) with an extra non-dynamical flat metric. The theory is known to pass solar system tests of GR where it is indistinguishable from it (Lee et al. 1976). However, the theory has problems when it comes to pulsar and binary pulsar observations (Lee et al. 1976; Will and Eardley 1977). Namely, the theory allows for states with energy unbound from below and the emission of gravitational waves with negative energy. This would cause an increase of the spin of pulsars that is not compatible with observations of millisecond pulsars as shown in Lee et al. (1976). Similarly, Will and Eardley (1977) found that such a theory predicts large emission of dipole gravitational radiation that will increase the orbital period of the binary pulsar system to a level again inconsistent with observations of such systems.

Another bimetric gravity theory is that of Eddington–Born–Infield (EBI) (Eddington 1924; Bañados 2007, 2008; Banados 2008). It is based on extentions to Eddington theory of affine connections. It combines the metric tensor plus a connection. It was shown in Bañados et al. (2009b) that the connection can be replaced by a corresponding metric and thus expressing the theory as a bimetric gravity with interesting cosmological features that can account for dark matter and dark energy and thus a possible unifying theory (Bañados et al. 2009a, b; Hu 1998). It was shown in Bañados et al. (2009a) that if one wants to keep the unification of dark matter and dark energy in this model, the integrated Sachs–Wolf effect is thus too large and becomes inconsistent with observations. Also, such a theory would also predict an angular power spectrum and galaxy power spectrum that are not consistent with current observations.

Next, Drummond (2001) proposed a tensor–tensor theory formulated using two sets of dynamical tetrads (vierbeins). The theory has a length scale of galactic size. Below such a length scale, it passes the standard test of GR but beyond such a scale it acquires an effective gravitational constant larger than Newton’s constant. The author argues that the transition galactic scale can explain the flat velocity rotation curves of galaxies and can account for an alternative to dark matter. It is not clear from current literature whether this theory suffer from the same constraints as the EBI theory above as very little work has been done on its cosmological constraints.

Gabadadze et al. (2012) proposed models to implement Galileons on curved spacetime by coupling a scalar with the galilean symmetry to a massive graviton. The models can maintain second order equations of motion, maintain the galilean shift symmetries, and allow the background metric to be dynamical. The models can be viewed as an extension of the ghost-free massive gravity, or as a massive graviton-Galileon scalar–tensor theory. They have higher order equations of motion and infinite powers of the field, but are ghost-free. We refer the reader to the original paper. Finally, Milgrom (2009, 2010) proposed a bimetric extension to MOND that reduces to MOND on small scales and the low acceleration regime of the theory. Cosmological aspects of the theory were studied in Clifton and Zlosnik (2010) and Milgrom (2010) finding that it can reproduce an FLRW evolution in the high acceleration limit. Some solutions can have cosmic acceleration due to a cosmological constant term in the theory but with some problems. Namely, Clifton and Zlosnik (2010) found that the solutions that remain in such a high acceleration regime for the entire evolution require either non-baryonic dark matter or extra terms in the original action, or else they fail observational constraints of $\varOmega _{\varLambda }$ and do not predict the right position of the first peak of the CMB temperature spectrum.

### Modified gravity theories with higher-order derivatives

**Fig. 13:**
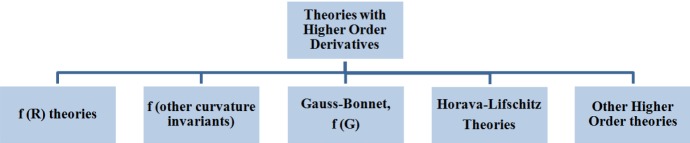
Higher-order-derivatives modified gravity models

Modification to GR can also be realized by allowing for higher order derivatives of the metric to be present in the equations of motion. Such theories can for example be derived from higher-order invariants built from the Riemann curvature tensor and the metric. Shortly after Einstein proposed GR, other theories of gravity using scalar invariants more general than the Ricci scalar were proposed (Weyl 1918). In addition to an interesting phenomenology, it has been argued that the models have theoretical motivations within unification theories of fundamental interactions and within field quantization on curved space-times (Utiyama and DeWitt 1962; Stelle 1977; Birrell and Davies 1984). Figure 13 shows some sub-categories of higher-order derivative theories.

However, the problem with this route is that it leads to theories that have problematic fields with states that admit negative unbound energy when quantized, known as ghost fields (Stelle 1978; Calcagni et al. 2005; Hindawi et al. 1996a, b; Chiba 2005; Navarro and Van Acoleyen 2006; DeFelice et al. 2006; Barth and Christensen 1983; Nunez and Solganik 2005). This is formulated as Ostrogradski’s instability theorem stating that for a nondegenerate Lagrangian which depends on higher derivatives, the Hamiltonian is necessarily unbounded, see Woodard (2007) and Ostrogradski (1850). Dolgov and Kawasaki (2003), Faraoni (2006a) and Seifert (2007) Other instabilities for such theories have also been raised in Frolov (2008).

Luckily, there is yet a limited number of higher-order derivative theories that by some particular construction avoid the presence of ghosts. These include, for example, the popular *f*(*R*), Hořava–Lifschitz, and Gauss–Bonnet theories. Additionally, there has been most recently some developments in models beyond Horndeski with higher-order derivatives but some degeneracies making them ghost-free, see Sect. 7.3.1.

We describe below some aspects of *f*(*R*) and Hořava–Lifschitz theories as two illustrative examples for this category and list further below other selected models.

#### Illustrative example 1: *f*(*R*) theories

These theories derive from using a general function *f*(*R*) in the action instead of simply *R* as is the case in GR. The action reads

208 $$\begin{aligned} S=\int d^4 x \left( \sqrt{-g}f(R) + {16 \pi G}\,\mathcal {L}_m(\psi _{\mathrm{m}}, g_{\mu \nu })\right) , \end{aligned}$$

where $\mathcal {L}_m(\psi _{\mathrm{m}}, g_{\mu \nu })$ is the Lagrangian of the matter field, $\psi _{\mathrm{m}}$. Varying (208) with respect to the metric gives the field equations

209 $$\begin{aligned} f_R R_{\mu \nu } - \frac{1}{2} f g_{\mu \nu } - f_{R;\mu \nu }+g_{\mu \nu } \square f_R= 8 \pi G T_{\mu \nu }, \end{aligned}$$

where $f_R \equiv \partial f(R)/\partial R$ and $\square \equiv \nabla ^\mu \nabla _\mu$ is the d’Alembertian operator. Obviously, when $f(R)=R$, the above reduces to Einstein’s equations. It is worth mentioning that, unlike the case of GR, variation of the action (208) with respect to the metric and the connection independently (known as the Palatini approach) leads to a different set of Field equations and thus different theories.

We have put *f*(*R*) in this section but it is fair to mention that *f*(*R*) models can also be classified under scalar–tensor theories due to the equivalence between this formulation and that involving an additional scalar-field as we show further in this section.

For an FLRW curved background metric, the field equations above generalized Friedmann equations read

210 $$\begin{aligned} H^2= & {} \frac{1}{3 F} \left[ 8 \pi G {\bar{\rho }} - \frac{1}{2} (f-RF)-3 H \dot{F} \right] - \frac{\kappa }{a^2}, \end{aligned}$$

211 $$\begin{aligned} \dot{H}= & {} -\frac{1}{2F} ( 8 \pi G ({\bar{\rho }} +\bar{P}) +\ddot{F} - H \dot{F}) + \frac{\kappa }{a^2}, \end{aligned}$$

where we further note $F\equiv f_R$, the Ricci scalar is given by $R = 6 ( 2 H^2 +\dot{H} + \kappa /a^2 )$, over-dots denote derivatives with respect to cosmic time *t*, and $\kappa$ is the curvature of spatial sections.

*f*(*R*) theories have been shown to exhibit a very interesting cosmological phenomenology as they can have solutions that are self-accelerating and thus produce early time inflation or late-time observed cosmic acceleration with no requirement for a cosmological constant (Nojiri and Odintsov 2007b, 2008a, b, c; Bamba and Odintsov 2008). Furthermore, other proposals aim to provide a unifying scenario by producing early time inflationary acceleration as well as late-time cosmic acceleration (Cognola et al. 2008; Elizalde et al. 2011). Self-acceleration results from a more complex coupling between matter and curvature in such models that can be expressed, as we write below, as conditions on the functions *f*(*R*) and its derivatives.

In the context of cosmic acceleration, one can re-write the generalized Friedmann equations (210) and (211) as those of GR plus additional terms that can be recast into terms associated with state variables of an effective dark fluid as follows

212 $$\begin{aligned} 8 \pi G \rho _{\text {eff}}= & {} \frac{{ RF}-f-6 { H}\dot{F}}{2 F} \end{aligned}$$

213 $$\begin{aligned} 8 \pi G P_{\text {eff}}= & {} \frac{2 \ddot{F} +4 H \dot{F} +f -RF}{2F}. \end{aligned}$$

From the corresponding equation of state,

214 $$\begin{aligned} w = \frac{2 \ddot{F} +4{ H}\dot{F} +f -{ RF}}{{ RF}-f-6 H \dot{F}}, \end{aligned}$$

one can then impose the condition for cosmic acceleration, $w < -1/3$, and find the conditions required on the function *f*(*R*) and its derivatives to produce late-time self-accelerating models.

Some limitations of the emergence of cosmic acceleration in *f*(*R*) models were discussed in Clifton and Dunsby (2015).

At least three self-accelerating *f*(*R*) models have been popular and compared extensively to observations. These are the models of Starobinsky (2007) for early inflation

215 $$\begin{aligned} f(R)=R-\mu R_c \left[ 1- \left( 1+\frac{R^2}{R_c^2} \right) ^{-n} \right] , \end{aligned}$$

Hu and Sawicki (2007a) with

216 $$\begin{aligned} f(R)=R- \frac{ \mu R_c }{1+(R/R_c)^{-2n}}, \end{aligned}$$

and Appleby and Battye (2007) with

217 $$\begin{aligned} f(R)=R+R_c \log \left[ e^{-\mu }+(1-e^{-\mu })e^{-R/R_c} \right] \end{aligned}$$

for late-time acceleration, where $\mu$, *n* and $R_c$ are positive constants.

It is also worth mentioning the designer approach to models of *f*(*R*) as in, for example, Song et al. (2007a), Pogosian and Silvestri (2008) and Nojiri and Odintsov (2006b, 2007a). In this approach, the *f*(*R*) model is reconstructed from a predefined background expansion history such as that given by the Hubble function of the $\varLambda \mathrm {CDM}$. Using this as a requirement for *f*(*R*) Friedmann’s equations gives an inhomogeneous second-order differential equation that can be solved numerically and using specific initial conditions, $f_{R0}$, or a Compton wavelength parameter.

While *f*(*R*) models brought some excitements in the community as being serious contenders to GR, their viable models require the chameleon screening mechanism in order to pass solar system constraints. However, as we discuss in Sect. 8.1, models requiring a chameleon-like screening mechanism were shown in Wang et al. (2012) to fail to produce the observed cosmic acceleration unless added with a cosmological constant. Thus, the models fail to be properly screened and simultaneously possess a self-acceleration feature.

It is worth noting that in order to assure well behaved initial conditions in *f*(*R*) models at early epochs when curvature is high, the following condition is required

218 $$\begin{aligned} \lim _{R\rightarrow \infty } f(R)/R \rightarrow 0. \end{aligned}$$

In this way, any modification to gravity in *f*(*R*) viable models happens well after radiation is negligible. As we will see further below, *f*(*R*) theory can be expressed as a scalar–tensor theory with a scalaron field. As mentioned above, a second common practice used for *f*(*R*) models is to parameterize them using the dimensionless Compton wavelength (of the scalaron) in Hubble units given by Song et al. (2007a) and Hu and Sawicki (2007a)

219 $$\begin{aligned} B\equiv \frac{f_{RR}}{1+f_R}R'\frac{H}{H'}, \end{aligned}$$

where $f_{RR}=\mathrm{d}^2 f/\mathrm{d} R^2$ and ${~}'=\mathrm{d} /\mathrm{d} \ln a$ here. This allows $f_{RR}$ to control the modification to gravity and solutions with a given expansion history can be characterized by $B_0 \equiv B(\ln a=0)$ (Song et al. 2007a). For GR, $B(a)=0$. It is customary for cosmological analyses to constrain the $B_0$ parameter.

Cosmological perturbations for *f*(*R*) have been fully worked out in a number of studies. See for example the reviews (Clifton et al. 2012; De Felice and Tsujikawa 2010) for a summary and references. Using the flat perturbed FLRW metric in Newtonian gauge, the following informative relations can be obtained in the quasi-static approximation (De Felice and Tsujikawa 2010). First, the gravitational potentials are given by

220 $$\begin{aligned} \varPsi \simeq \frac{1}{2F} \left( \delta F-\frac{a^2}{k^2} \kappa ^2 \delta \rho _m \right) , \end{aligned}$$

and

221 $$\begin{aligned} {\varPhi } \simeq -\frac{1}{2F} \left( \delta F +\frac{a^2}{k^2} \kappa ^2 \delta \rho _m \right) . \end{aligned}$$

where $\delta F$ satisfies $(k^2/a^2+M^2)\delta F \simeq \kappa ^2\delta \rho _m/3$ and the mass parameter *M* is given by

222 $$\begin{aligned} M^2 = \frac{F - R F_{R}}{3 F_{R}}. \end{aligned}$$

The modified Poisson equations are given by

223 $$\begin{aligned} \frac{k^2}{a^2}\varPsi \simeq -\frac{\kappa ^2 \delta \rho _m}{2F} \frac{2+3M^2a^2/k^2}{3(1+M^2a^2/k^2)}, \end{aligned}$$

and

224 $$\begin{aligned} \frac{k^2}{a^2}\varPhi \simeq -\frac{\kappa ^2 \delta \rho _m}{2F} \frac{4+3M^2a^2/k^2}{3(1+M^2a^2/k^2)}. \end{aligned}$$

The Weyl potential $\varPsi _{\mathrm{w}} \equiv (\varPhi +\varPsi )/2$ that enters observations of for example gravitational lensing and the ISW effect is given by

225 $$\begin{aligned} \varPsi _{\mathrm{w}} \simeq -\frac{\kappa ^2}{2F} \frac{a^2}{k^2} \delta \rho _m. \end{aligned}$$

The difference of the two potentials is given by

226 $$\begin{aligned} \varPsi -\varPhi = - \frac{8 \pi a^2 ({\bar{\rho }}+\bar{P}) \sigma }{F} - \frac{\delta F}{F} \end{aligned}$$

so that even in absence of shear, there is still a slip parameter between the two potentials due to modification to gravity.

Before we end this section, it is worth showing how *f*(*R*) theories can be formulated in terms of equivalent scalar field actions and what implications that has. First, we observe that the action (208) is equivalent to that of a scalar field as

227 $$\begin{aligned} S=\int d^4 x\sqrt{-g} \left( f(\phi ) + (R- \phi ) \frac{df(\phi )}{d\phi } \right) + {16 \pi G}\, \mathcal {L}_m(\psi _{\mathrm{m}}, g_{\mu \nu }). \end{aligned}$$

Varying with respect to the scalar field $\phi$ gives $(R - \phi ) \frac{d^2f(\phi )}{d\phi ^2}=0$ so $R=\phi$ for all $\frac{d^2f(\phi )}{d\phi ^2}\ne 0$, showing that the action is indeed equivalent to (208).

Next, one can introduce an auxiliary field, $\psi \equiv \frac{df(\phi )}{d\phi }$, and define a potential $V(\psi )$ as the Legendre transform of the function $f(\phi )$ given by

228 $$\begin{aligned} V(\psi )=f(\phi (\psi ))-\phi (\psi ) \psi \end{aligned}$$

so the action (208) can be written in the Jordan frame as

229 $$\begin{aligned} S=\int d^4 x\sqrt{-g} \left( \psi R - V(\psi ) \right) + {16 \pi G}\, \mathcal {L}_m(\psi _{\mathrm{m}}, g_{\mu \nu }). \end{aligned}$$

This action has now taken the well-known form of a non-minimally coupled scalar–tensor theory as discussed in the Generalized Brans–Dicke theory (Sect. 7.3.1) with the parameter $\omega _{\mathrm{BD}}=0$. Importantly, the equations of motion of the theory are second order in the metric derivatives instead of fourth order and no ghost modes are present.

Finally, one can show that the *f*(*R*) action can be transformed to the Einstein frame using a conformal transformation $g_{\mu \nu } =A(\phi )^2 \bar{g}_{\mu \nu }$ and by redefining the scalar field. One can then write

230 $$\begin{aligned} S = \int d^4 x \sqrt{-\bar{g}}\Big ({\bar{R}} - \frac{1}{2} (\partial \phi )^2- \bar{V}(\phi ) \Big ) +16 \pi G \int d^4 x \sqrt{-g} {\mathcal {L}}_m \Big ( \psi _{\mathrm{m}}, A(\phi )^2 \bar{g}_{\mu \nu } \Big ). \end{aligned}$$

Now, the theory appears in this frame as that of a scalar minimally coupled to curvature, however, the scalar field couples now directly to the matter fields.

We refer the reader to the specialized reviews of *f*(*R*) theories and their cosmology in De Felice and Tsujikawa (2010) and Sotiriou and Faraoni (2010) and references therein.

#### Illustrative example 2: Hořava–Lifshitz

Hořava–Lifshitz gravity theory was proposed with the motivation to quantize gravity (Hořava 2009a, b, c). The idea was to provide an ultraviolet (UV) completion of GR at the expense of breaking Lorentz invariance. For this, this theory also belongs to the category of Lorentz breaking theories. Such an invariance is however approximately recovered (i.e., staying below experimental constraints) in the infrared (IR) regime. Following early studies of scalar fields by Lifshitz (1941), Hořava proposed to use an anisotropic scaling between space and time dimensions as

231 $$\begin{aligned} \mathbf {x}\rightarrow l \mathbf {x}; \,\,\,\,t \rightarrow l^z t, \end{aligned}$$

where *z* is called the dynamical critical exponent and the theory is often referred to as Hořava’s gravity at a Lifshitz point *z*. This anisotropic treatment of space and time allowed the theory to avoid the Ostrogradski’s ghost problem by allowing it to have higher order spatial derivatives but no time higher order derivatives.

A convenient formalism to express a theory with such a split between time and space is the Arnowitt–Deser–Misner (ADM) decomposition of spacetime, see e.g., Misner et al. (1973), given by

232 $$\begin{aligned} d s^2 = - N^2 c^2 \, dt^2 + g_{ij}(dx^i + N^i \, dt) (dx^j + N^j \, dt), \end{aligned}$$

where $N(t,x^i)$ is the lapse function and $g_{ij}$ is the 3-space metric. One is restricted to pick a preferred foliation of spacetime due to the anisotropy discussed above. Another important feature, but also source of problems, is that the GR invariance under diffeomorphisms is replaced by the more restrictive foliation preserving diffeomorphisms as

233 $$\begin{aligned} t\rightarrow \tilde{t}(t),\qquad x^i\rightarrow \tilde{x}^i(t,x^i). \end{aligned}$$

The most general action for such a theory with second-order only time derivatives is given by

234 $$\begin{aligned} S=\frac{M_{\mathrm{pl}}^2}{2}\int d^3x d t N \sqrt{g} \left\{ K^{ij} K_{ij} - \lambda K^2 -V(g_{ij},N)\right\} \, , \end{aligned}$$

where $M_{\mathrm{pl}}$ is a constant that can be identified with the Planck mass, $\lambda$ is a dimensionless running coupling constant, and *V* is a potential function depending on the spatial metric, the lapse function and their spatial derivatives. $K_{ij}$ is the extrinsic curvature given by

235 $$\begin{aligned} K_{ij} = {1\over 2N} \left\{ \dot{g}_{ij} - \nabla _i N_j - \nabla _j N_i \right\} , \end{aligned}$$

where an overdot is for differentiation with respect to the time coordinate and $\nabla _i$ is the covariant derivative associated with the spatial metric.

The Hořava–Lifshitz theory can have different versions. One version is said to have a *detailed balance* property and is based on specific symmetry properties of the potential function *V* (Hořava 2009a, b). Hořava proposed *detailed balance* to simplify the theory by reducing the number of curvature invariants needed to describe its formalism. Also, depending on whether the lapse function, *N*, is a function of time or a function of time and space coordinates, the theory is said to be projectable or non-projectable, respectively, with a number of implications including the cosmological evolution.

The original Hořava–Lifshitz theory had a number of problems and various improvements have been proposed, see e.g. Blas et al. (2010, 2011b), Hořava and Melby-Thompson (2010), Zhu et al. (2011, 2012) and Lin et al. (2014). The theory and its improved versions have interesting phenomenological implications in the infra-red (low-energy) regime where, for example, it was shown that an integration constant can play the role of dark matter (Mukohyama 2009a). It was also argued in Appignani et al. (2010) that the presence of a bare geometrical cosmological constant in Hořava–Lifshitz with detailed balance can be used to address the cosmological constant problem, although still with some fine tuning. They do that by cancellation of the negative geometrical cosmological constant term from the theory against the vacuum energy term, leaving only a very small observed value (Appignani et al. 2010).

Cosmology of Hořava–Lifshitz theory has been discussed in a number of other papers including the reviews (Sotiriou 2011; Calcagni 2009; Gong et al. 2010; Misonoh et al. 2017) for the projectable case; (Kobayashi et al. 2010a) for non-projectable case; (Huang and Wang 2011, 2012; Huang et al. 2012) for the projectable case with *U*(1) local symmetry Hořava and Melby-Thompson 2010; and Zhu et al. 2013a, b for the non-projectable case with the same *U*(1) local symmetry.

For cosmology, we provide a few general illustrative results following Sotiriou (2011). Under the foliation preserving diffeomorphisms, the theory is written in a prefered foliation. One can choose

236 $$\begin{aligned} N=1,\quad N^i=0,\quad g_{ij}={a(t)}^2\delta _{ij}, \end{aligned}$$

so that the ADM line element (232) coincides with the FLRW metric

237 $$\begin{aligned} ds^2=-dt^2+a(t)^2 \left[ \frac{dr^2}{1-k r^2}+r^2\left( d\theta ^2+\sin ^2 \theta d\phi ^2\right) \right] . \end{aligned}$$

For the cosmological solution, the difference here between projectable and non-projectable theory manifests itself on the Hamiltonian constraint which is global in the projectable theory and local in the non-projectable one. This subtlety needs to be taken into account when studying the background cosmology dynamics as we delineate below. Indeed, the Hořava–Lifshitz field equations give two generalized Friedmann equations as follows.

In the projectable case, the Hamiltonian constraint is global and gives the first equation as an integral

238 $$\begin{aligned} \int d^3 x a^3 \left\{ \frac{3\lambda -1}{2}\; {\dot{a}^2\over a^2} - {V(a)\over 6} - {8\pi G_N {\rho }\over 3}\right\} =0, \end{aligned}$$

where ${\rho }\equiv -g^{-1/2} \delta S_M/\delta N$ and $S_M$ is the matter action, Of course, for the FLRW metric, the integrand in (238) is a function of time only and gets out of the space integral so it gives Eq. (240) below. But we assume that when Sotiriou (2011) writes (238) in his review, he meant the case where the universe is not globally isotropic and homogeneous so the integrand in (238) does depend on spatial coordinates.

The potential above is given by

239 $$\begin{aligned} V(a) = g_0\, M_{\mathrm{pl}}^2 + {6 g_1 k \over a^2} + {12(3g_2+g_3) k^2 \over M_{\mathrm{pl}}^2\,a^4} + {24(9 g_4 + 3g_5+ g_6) k\over M_{\mathrm{pl}}^4 \, a^6}. \end{aligned}$$

where the $g_{i}$ are dimensionless couplings from the action (Sotiriou 2011).

In the non-projectable case, the Hamiltonian constraint is local so we can get rid of the integral to write (Sotiriou 2011)

240 $$\begin{aligned} \frac{3\lambda -1}{2}\; {\dot{a}^2\over {a}^2} - {V(a)\over 6} = {8\pi G_N {\bar{\rho }}\over 3}. \end{aligned}$$

The second Friedmann equation is the same for the two cases and is given by Sotiriou (2011)

241 $$\begin{aligned} - \frac{3\lambda -1}{2} \; {\ddot{a}\over {a}}= & {} {1\over 2}\frac{3\lambda -1}{2} {\dot{a}^2\over {a}^2} - {1\over 12 {a}^2} {d[V(a)\, {a^3}]\over d {a}} + 4\pi G_N \bar{p}, \end{aligned}$$

where $$\bar{p}\equiv -g^{ij}(2/N\sqrt{g})\delta S_m/\delta g^{ij}$$.

In the non-projectable case, $\dot{a}$ can be eliminated from (241) by use of (240) to write

242 $$\begin{aligned} - \frac{3\lambda -1}{2} \; {\ddot{a}\over {a}}= & {} -{1\over 12 {a}} {d[V({a}) {a}^2] \over d {a}} +{4\pi G_N\over 3} ({\bar{\rho }}+3\bar{p}). \end{aligned}$$

Next, differentiating (240) and subtracting it from (242) gives the usual conservation law as in GR and that will be used further below (Sotiriou 2011)

243 $$\begin{aligned} \dot{{\bar{\rho }}}+3\frac{\dot{a}}{a} ({\bar{\rho }}+\bar{p})=0. \end{aligned}$$

Mukohyama (2009a) argued that the global nature of the Hamiltonian constraints in the projectable case has some specific implications for cosmic evolution. That is Eq. (238) is irrelevant locally inside the Hubble horizon. Therefore, one has to work only with Eq. (241) and ignore (238). Following the argument and integrating Eq. (241) gives

244 $$\begin{aligned} \frac{3\lambda -1}{2}\; {\dot{a}^2\over {a}^2} - {V({a})\over 6} = \frac{8\pi G_N}{3} \left( {\bar{\rho }}+\frac{C(t)}{a^3}\right) , \end{aligned}$$

where the form of *C*(*t*) depends on the conservation law satisfied by the matter source. Using (244) in (241) to eliminate $\dot{{a}}$, one writes (Sotiriou 2011)

245 $$\begin{aligned} - \frac{3\lambda -1}{2} \; {\ddot{a}\over {a}}= & {} -{1\over 12 {a}} {d[V({a}) {a}^2] \over d {a}} +{4\pi G_N\over 3} \left( {\bar{\rho }}+\frac{C(t)}{{a}^3}+3\bar{p}\right) . \end{aligned}$$

Interestingly, now the only difference between the two pairs of equations describing the background cosmological evolution, i.e., [(244) and (245) for the projectable case versus ((240) and (242)) for the non-projectable case] is the presence of the function *C*(*t*).

In the projectable case, if we suppose that the state variables ($\rho$ and *p*) satisfy the conservation equation (243) then *C*(*t*) reduces to a constant and the corresponding term in the Friedmann equations (244) and (245) above play the role of a pressureless dark matter component as shown in Mukohyama (2009a). Last, it is worth noting that in the case of spatially curved geometry, two additional terms are present in the potential *V*(*a*) and thus the Friedmann equations. The first of the last two terms in the potential is referred to as dark radiation and is proportional to $a^{-4}$ and the very last term is associated with a stiff matter and is proportional to $a^{-6}$ (Sotiriou 2011).

Finally, we close the discussion of the Hořava–Lifshitz theory by pointing out to a recent development of what is now called the *healthy extension* theory proposed by Blas et al. (2010). The theory avoids persistent instabilities in the original theory and remains power-counting renormalizable (Blas et al. 2010). The theory reduces in the low-energy limit to a scalar–tensor theory with deviations from GR that can be made small by some choice of the parameter space. This healthy theory admits a solution around a static mass that has a gravitational potential of the same form as the GR Schwarzschild solution with Blas et al. (2010)

246 $$\begin{aligned} \varPsi =\varPhi =-\frac{m}{8\pi M_p^2(1-\alpha /2)r} \end{aligned}$$

with an effective gravitational constant

247 $$\begin{aligned} G_N=[8\pi M_p^2(1-\alpha /2)]^{-1}. \end{aligned}$$

For a cosmological homogeneous background, the theory gives dynamical equations that differ from GR only by the presence of the $\lambda$ coupling. The first Friedmann equation is then given by

248 $$\begin{aligned} H^2=\frac{8 \pi G_{\mathrm{eff-cosmo}}}{3}{{\bar{\rho }}} \end{aligned}$$

with an effective gravitational constant at cosmological scales given by

249 $$\begin{aligned} G_{\mathrm{eff-cosmo}}=\frac{2}{2\pi M^2_p}(3\lambda -1), \end{aligned}$$

so $\lambda =1$ restores GR Friedmannian cosmological evolution.

The observational bound on the deviation from GR is provided from the measurement of the primordial abundance of He$^4$ which gives (Jacobson 2008; Carroll and Lim 2004)

250 $$\begin{aligned} |G_{\mathrm{eff-cosmo}}/G_N - 1| \le 0.13, \end{aligned}$$

thus putting only mild constraints on the parameters $\alpha$ and $\lambda$ of the theory.

Perturbations for the Hořava–Lifshitz theory have been worked out in a number of papers including (Mukohyama 2009b; Gao et al. 2010; Wang et al. 2010a; Wang 2010). The growth equations are different from those of GR and offer the possibility to test these theories using large-scale structure.

Recent reviews, papers and progress reports on Hořava–Lifshitz gravity and cosmology can be found in Wang (2017), Sotiriou (2011), Calcagni (2009) and references therein. Discussions about the implications of Lorentz symmetry violations in Hořava–Lifshitz theory can be found in Sotiriou et al. (2009a, b), Visser (2009), Nikolic (2010), Cai et al. (2009), Charmousis et al. (2009), Li and Pang (2009) and Blas et al. (2009b) while more about its cosmology can be found in Kiritsis and Kofinas (2009), Brandenberger (2009), Mukohyama et al. (2009), Saridakis (2010), Mukohyama (2009a, 2010), Gao et al. (2010), Wang and Wu (2009), Wang and Maartens (2010) and Ferreira and Brandenberger (2012).

#### Other higher order derivative theories

Conformal Weyl gravity has been actively pursued and developed by Mannheim and Kazanas (1991) and Mannheim (2012) but the theory goes way back to the early work of Weyl (1918). The gravitational action is built solely from the Weyl tensor contracted with itself as

251 $$\begin{aligned} S_{CG}=\int d^4 x\sqrt{-g}C_{\alpha \beta \gamma \delta }C^{\alpha \beta \gamma \delta }, \end{aligned}$$

where the Weyl conformal tensor is given by

252 $$\begin{aligned} C_{\alpha \beta \gamma \delta }= & {} R_{\alpha \beta \gamma \delta }+\frac{1}{6}R [g_{\alpha \gamma }g_{\delta \beta }-g_{\alpha \delta }g_{\gamma \beta }]\nonumber \\&+\frac{1}{2}[g_{\alpha \delta }R_{\gamma \beta } +g_{\beta \gamma }R_{\delta \alpha }-g_{\alpha \gamma } R_{\delta \beta }-g_{\beta \delta }R_{\gamma \alpha }]. \end{aligned}$$

Even if the theory contains fourth order derivatives in the metric, it has been argued in Bender and Mannheim (2008), Mannheim (2007) and Pavšič (2013) that it is free of ghosts, although with some further open discussions in Pavšič (2016).

The theory has an interesting phenomenology with a solution for a spherically symmetric field that has a metric component potential with two extra terms compared to that of the GR Schwarzschild’s solution, e.g., Mannheim (2012). It was argued that one of the additional terms can explain galaxy flat rotation curves as an alternative to dark matter while the second term can play the role of a cosmological constant (Mannheim 2006). The theory was often discussed in the context of rotation curves and as an alternative to dark matter (Mannheim and O’Brien 2013; Mannheim 2006), however, it was also argued in, for example, Mannheim (1999, 2012) that the theory could help to address the cosmological constant problem.

Some work has been done showing that the theory passes some solar system tests such as the bending of light, e.g. Cattani et al. (2013) and Sultana and Kazanas (2010), although debated in, e.g. Campigotto et al. (2017). It was also claimed in Yoon (2013) that Mannheim’s conformal gravity potential is problematic as it cannot reduce to a proper Newtonian limit at short distances without singularities in the mass density source. This was refuted in an extended response by Mannheim (2016). It was also found to fit some astrophysical distance tests in Yang et al. (2013) and Diaferio et al. (2011) but was criticized as its Big Bang Nucleosynthesis predictions are not consistent with observations (Knox and Kosowsky 1993). Caprini et al. (2018) investigated very recently the gravitational radiation from Pulsar binary systems in conformal gravity using the system PSR J1012+5307. They found that when fixing the graviton mass in conformal gravity so that the theory fits galaxy rotation curves without dark matter, the gravitational radiation from the system is much smaller than in GR and cannot explain the orbital decay of the binary system. At the cosmology level, more work remains to be done to compare conformal gravity to full data of CMB and large scale structure.

Another theory worth mentioning in this section is that built from the Gauss–Bonnet invariant,

253 $$\begin{aligned} G=R^2-4R^{\alpha \beta }R_{\alpha \beta }+R^{\alpha \beta \gamma \delta }R_{\alpha \beta \gamma \delta }, \end{aligned}$$

constructed from this specific combination of the Ricci scalar squared, the Ricci tensor and Riemann tensor contracted with themselves. Albeit being quadratic in the Riemann and Ricci tensors, the Gauss–Bonnet combination gives equations of motion that are ghost free, e.g., DeWitt (1965), Li et al. (2007) and Akbar and Cai (2006). Furthermore, the graviton itself may still become a ghost in the FLRW background, so further no-ghost conditions must be imposed on the background, see e.g., DeFelice et al. (2006). Some models have been shown to be also free from other instabilities due to superluminal propagations and fit cosmological expansion constraints De Felice and Tsujikawa (2009), Moldenhauer and Ishak (2009) and Moldenhauer et al. (2010). The action is given by

254 $$\begin{aligned} S=\int {d^{4}x \sqrt{-g}\left[ \frac{1}{2}R+f(G)\right] +\int {d^{4}x}\sqrt{-g}L_m}+\int {d^{4}x}\sqrt{-g}L_{\mathrm{rad}}, \end{aligned}$$

where we has set in this sub-section $8 \pi G \equiv 1$, $L_m$ and $L_{rad}$ are the matter and radiation Lagrangians, respectively. Here, the Gauss–Bonnet term is effective at cosmological scales.

Varying the action with respect to the metric gives the field equations

255 $$\begin{aligned}&{8[R_{\alpha \gamma \beta \delta }+R_{\gamma \beta }g_{\delta \alpha }-R_{\gamma \delta }g_{\beta \alpha }-R_{\alpha \beta }g_{\delta \gamma }+R_{\alpha \delta }g_{\beta \gamma } +\frac{1}{2}R(g_{\alpha \beta }g_{\delta \gamma }-g_{\alpha \delta }g_{\beta \gamma })]\nabla ^{\gamma }\nabla ^{\delta }}f_G\nonumber \\&\quad +\,(Gf_G-f)g_{\alpha \beta } + R_{\alpha \beta }-\frac{1}{2}g_{\alpha \beta }R= T_{\alpha \beta }, \end{aligned}$$

where we use the definition $f_G\equiv \frac{\partial {f}}{\partial {G}}$.

Now using the FLRW flat metric and a universe filled with matter and radiation, one derives the generalized Friedmann equation

256 $$\begin{aligned} 3H^2=Gf_G-f{G}-24H^3\dot{f}_{G}+{\bar{\rho }}_{m} +{\bar{\rho }}_{\mathrm{rad}}. \end{aligned}$$

where ${\bar{\rho }}_m$ and ${\bar{\rho }}_{rad}$ are the matter and radiation energy densities, respectively, a dot represents *d* / *dt*. Also, in terms of *H*,

257 $$\begin{aligned} R=6(\dot{H}+2H^2) \end{aligned}$$

and

258 $$\begin{aligned} G=24H^2(\dot{H}+H^2). \end{aligned}$$

Several models were proposed in De Felice and Tsujikawa (2009) and shown to be consistent with observations of supernova magnitude-redshift data, distance to the CMB surface, baryon acoustic oscillations (BAO), and Hubble Key project and age constraints in Moldenhauer and Ishak (2009) and Moldenhauer et al. (2010). However, it was shown in De Felice et al. (2010b) that *f*(*G*) models have generic divergent modes for matter linear perturbations which ruled them out. However, other theories based on using the Gauss–Bonnet invariant in higher dimensions known as Einstein–Gauss–Bonnet models are not ruled out and are still subject to discussions (see Sect. 7.5).

A third theory worth listing here is the Chern–Simons theory which is also based on using specific combination of higher order curvature invariants. The theory can arise from particle physics, string theory or from geometrical considerations. An extensive review of the theory is given by Alexander and Yunes (2009).

Finally, it is worth saying a few more words about models built from more general invariants than the Ricci scalar. A revived interest was raised into them in the early 2000s as some models were shown to exhibit late-time self-accelerating expansion without a dark energy component (Carroll et al. 2005; Easson 2004; Easson et al. 2005). It was also shown in Sotiriou and Liberati (2007), Meng and Wang (2004) and Nojiri and Odintsov (2003) that they can have early-time inflation as well, thereby providing a possible unification scenario for the two accelerating phases. See for example reviews in Lobo (2008), Nojiri and Odintsov (2006a) and references therein. However, previous studies stressed that the models considered were chosen somewhat randomly due to the large spectrum of possible curvature invariants (e.g., Carroll et al. 2005; Dolgov and Kawasaki 2003), and a systematic approach to these models was highly desirable (Faraoni 2006b; Nojiri and Odintsov 2008b). Accordingly, Ishak and Moldenhauer (2009) proposed a systematic method to classify such models based on minimal sets of invariants. They explore an idea based on theorems from the theory of invariants in GR (Debever 1964; Carminati and McLenaghan 1991; Zakhary and Mcintosh 1997). The idea was that curvature invariants are not independent from each other and, for a given algebraic type of the Ricci tensor (see, e.g., the Segre classification Segre 1884; Stephani et al. 2003) and for a given Petrov type of the Weyl tensor (i.e., symmetry classification of space-times)—e.g., Petrov (2000), Pirani (1957), Penrose (1960) and Stephani et al. (2003)—there exists a complete minimal independent set (basis) of these invariants in terms of which all the other invariants may be expressed. As an immediate consequence of the connection made and the proposed approach, the number of independent invariants to consider is reduced from an infinite number to six in the worst case and to only two independent invariants in the case of primary interest of cosmology, i.e., all FLRW metrics.

Although this was an interesting idea for classification of this class of models, the determining factor to limit the number of physically acceptable models came from considering their quantization. It was quickly recognized that only a small subset of such models are free of ghost instabilities as we discussed in Sect. 7.4.

### Modified gravity theories with higher-dimensions

**Fig. 14:**
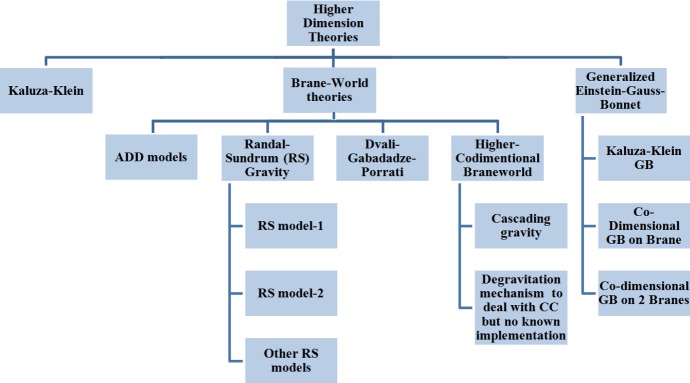
Higher-dimension modified gravity models

This class of models has been popular both in the scientific literature as well as in the media and public scene since extra dimensions beyond the 3+1 dimensions of GR has been the subject of much fantasy and fascination. Mathematically, studies of higher dimensional geometry have a long history going back to Riemannian geometry, a century and a half ago. Additionally, it is also worth mentioning that unification theories of physics such as superstring theory and supergravity require such higher dimensional spaces. Figure 14 shows some sub-categories of gravity theories with higher dimensions.

Accordingly, a number of MG models have been proposed with higher dimensions along with their corresponding cosmologies. We provide here a brief overview following the presentation of Clifton et al. (2012) and refer the reader to this and other MG extensive reviews (Berti et al. 2015; Joyce et al. 2015). After outlining some seminal or major developments on the topic, we present the Dvali–Gabadadze–Porrati (DGP) theory (Dvali et al. 2000) as an illustrative case.

#### Theories with compact dimensions versus braneworld models

One of the first developments of higher dimensional theories is that of Kaluza–Klein that aimed at unifying gravity and electromagnetism by the use of a fifth small (compact) dimension (Nordstrom 2007; Kaluza 1921; Klein 1926a, b). Such an additional dimension is compactified in a way so that the theory is effectively a four dimensional one and it does not alter observations in the solar system or at galactic scales but it may have effects at very tiny scales where it is probed using sub-milliliter gravitational experiments, or at cosmological scales where it may have some observational effects. This idea of compactified dimensions is adopted in string theory and supergravity that use 10 and 11 dimensions respectively but where the additional dimensions are compactified to leave only the usual 3+1 standard dimensions of space and time. Interestingly, Kaluza–Klein theory with one or more additional dimensions generically corresponds to an effective 4 dimensional theory with extra scalar fields such as the dilaton field. A dilaton field is scalar field that appears in theories with higher dimensions and compactification. Also, if the Newton constant or Planck mass are promoted to a scalar field in a given theory then it is a dilaton. A concise discussion about compactification and stabilization of such additional dimensions can be found in Clifton et al. (2012) and references therein.

A second distinct development came from the braneworld approach (Akama 2000; Rubakov and Shaposhnikov 1983; Arkani-Hamed et al. 1998, 1999; Antoniadis et al. 1994) where the extra dimensions can be large or even infinite. In such a scenario the usual 3$+$1 dimensional hypersurface is called the brane and is embedded in a higher dimensional space called the bulk. The extra large dimensions are said to be non universal in the sense that particles and fields are now constrained only to the brane and only gravity is felt through the bulk. The Braneworld approach is well-motivated by string theory and M-theory and their use of D-branes embedded in higher dimensional spaces (Hořava and Witten 1996; Lukas et al. 1999; Antoniadis et al. 1998; Polchinski 1995). This second approach has seen models with interesting cosmological applications that we discuss next, following Clifton et al. (2012).

A seminal proposal came from Arkani-Hamed et al. (1998) who proposed an extra dimension to solve the so-called hierarchy problem between the electro-weak scale and the Planck scale being separated by 16 orders of magnitude. The idea is that such a hierarchy can be related to the hierarchy between the scale of the new dimension introduced and the electro-weak scale. The bulk here is a flat 5 dimensional Minkowski space. This is commonly referred to ad the ADD model for the initials of its proposers Arkani-Hamed, Dimopoulos, and Dvali. The ADD model and the alike have been subject to stringent constraints from solar system tests, see e.g., Overduin (2000).

Following the work of Arkani-Hamed et al. (1998), Randall and Sundrum developed two braneworld models with an anti-de Sitter space as bulk. The first model has two branes (Randall and Sundrum 1999b) and the second model with one brane (Randall and Sundrum 1999a). The first Randal Sundrum model also aimed at solving the hierarchy problem using two branes separated by a 5-dimensional anti-de Sitter space and is well motivated by string and supergravity (Hořava and Witten 1996; Lukas et al. 1999; Duff et al. 2001). However, it is considered incomplete because, on the two branes, the effective theory of gravity is not GR but rather Brans–Dicke gravity with a BD parameter related to the branes and is being not consistent with current observational constraints on the BD parameter (Garriga and Tanaka 2000). The stabilization of moduli in the Randall–Sundrum model using the mechanism of Goldberger and Wise (1999) have been discussed in Lesgourgues and Sorbo (2004), Brümmer et al. (2006) and Dey et al. (2007). The second Randall Sundrum model has only one brane with a positive tension where GR is recovered as an effective theory and thus consistent with observations. Other models based on Braneworld constructions include the Karsh-Randal model (Karch and Randall 2001; Kaloper 1999), the Gregory, Rubakov and Sibiryakov model (Gregory et al. 2000), the asymmetric brane model (Padilla 2005a, b; Stoica et al. 2000), and the Charmousis, Gregory and Padilla model (Charmousis et al. 2007).

The cosmology of braneworld scenarios has been discussed in Binetruy and Langlois (2000), Bowcock et al. (2000), Padilla (2005a), Stoica et al. (2000), Shtanov et al. (2009), Maartens et al. (2001), Niz et al. (2008), Kiritsis et al. (2003), Kiritsis (2005), Umezu et al. (2006), Ichiki et al. (2002), Liddle and Smitha (2003) and cosmological perturbations have been worked out in Maartens (2000), Langlois et al. (2001), Maartens et al. (2000), Copeland et al. (2001), Sahni et al. (2002), Nunes and Copeland (2002), Liddle and Ureña-López (2003), Bridgman et al. (2002, 2001), Gordon and Maartens (2001), Langlois et al. (2000), Gorbunov et al. (2001), Mukohyama (2000a, b, 2001, 2002), Hawking et al. (2000, 2001), Kodama et al. (2000), Langlois (2000), van de Bruck et al. (2000a, b), Koyama and Soda (2000), Kobayashi et al. (2001, 2003), Kodama (2001), Langlois (2001), Deruelle et al. (2001), Brax et al. (2001), Dorca and van de Bruck (2001), Chen et al. (2002), Chung and Freese (2003), Deffayet (2002), Riazuelo et al. (2002), Leong et al. (2002) and Cardoso et al. (2007).

In braneworlds, the difference between the dimension of the bulk and the dimension of the brane is called the co-dimension of the brane. The discussions above are all for a co-dimension of one and that is what has been explored the most in the literature, however other models with higher co-dimensions have been discussed in Arkani-Hamed et al. (1998), Cline et al. (2003), Carroll and Guica (2003), Vinet and Cline (2004), Nilles et al. (2004), Aghababaie et al. (2004), Dvali and Gabadadze (2001), Dvali et al. (2003a, b), Gabadadze and Shifman (2004), Dubovsky and Rubakov (2003), Kaloper and Kiley (2007), Kaloper (2008), de Rham et al. (2008a, 2010), de Rham (2009), de Rham et al. (2009), Minamitsuji (2010), Agarwal et al. (2010), Corradini et al. (2008a, b), Bostock et al. (2004), Charmousis and Papazoglou (2008, 2009), Charmousis et al. (2010), Papantonopoulos et al. (2008, 2007) and Cuadros-Melgar et al. (2009, 2011). Moreover, cascading gravity has been proposed and is based on scenarios where higher dimensional gravity goes through steps cascading from $(4+n)D$ down to 4*D* gravity, as for example one transitions from larger scales to smaller and smaller scales, see for example de Rham et al. (2008a, b), de Rham (2009), Corradini et al. (2008a, b), Kaloper and Kiley (2007) and Kaloper (2008).

These higher co-dimensional or cascading gravity models have been useful in trying to address the cosmological constant problem (Carroll and Guica 2003; Cline et al. 2003; Vinet and Cline 2004; Aghababaie et al. 2004; Dvali et al. 2003b; Gabadadze and Shifman 2004) by for example using a degravitation mechanism for the cosmological constant leading to a “small” dynamical effect as measured by current cosmological observations (Arkani-Hamed et al. 2002).

Finally, we mention here briefly another class of higher dimensional theories called the Einstein–Gauss–Bonnet gravity. It is built in 5 or 6 dimensions and has as an action the Ricci scalar, a higher-D cosmological constant term (not the same as the 4D one), and a Gauss–Bonnet invariant [see Eq. (253)]. A variation of such an action with respect to the metric gives the Einstein equations in a higher dimension plus a Lovelock tensor term (Lovelock 1971; Lanczos 1938). This has found applications in string theory (Gross and Sloan 1987; Mannheim 2012). A concise discussion with examples can be found in Clifton et al. (2012).

#### Illustrative example : Dvali–Gabadadze–Porrati gravity (DGP)

The DGP model is a popular braneworld theory of gravity and is based on a 3$+$1 brane embedded in a five dimensional Minkowski space (Dvali et al. 2000) and is known to have two branches. One is a self-accelerating branch that can produce cosmic acceleration without the need for a cosmological constant or brane tension and has been the subject of much interest earlier in the literature, see extended discussions in Deffayet (2001), Deffayet et al. (2002a) and Lue (2006). This branch is however plagued with instabilities and the presence of ghost degrees of freedom, e.g., Charmousis et al. (2006) and Luty et al. (2003). It also turned out at the end that this self-accelerating branch is not consistent with cosmological observations as we discuss further below. The second *normal* branch (noted as nDGP) does not self-accelerate but has better stability properties than the former branch (Charmousis et al. 2006; Padilla 2007; Gregory et al. 2007; Gorbunov et al. 2006). In order to exhibit acceleration this branch uses a brane tension that leads to a phantom type effective dark energy equation of state, $w_{{\mathrm{EDE}}}<-1$. At the perturbation level, the fifth force in these models is screened by the Vainshtein mechanism, see Sect. 8.3.

We follow here the presentation of Clifton et al. (2012) to describe the model and its cosmology. The action is given by

259 $$\begin{aligned} S = M_5^3 \int d^5 x \sqrt{-\gamma } {\mathcal {R}}+ \int d^4 x \sqrt{-g}\left[ -2 M_5^3 K+\frac{M_4^2}{2} R-\sigma +{\mathcal {L}}_{\text {matter}}\right] , \end{aligned}$$

where ${\mathcal {R}}$ is the Ricci scalar built from the bulk metric $\gamma _{ab}$. $M_4$ and $M_5$ are the Planck scales in the brane and bulk, respectively. $g_{\mu \nu }$ is the metric on the brane, *R* its Ricci scalar, and $K=g^{\mu \nu } K_{\mu \nu }$ is the trace of extrinsic curvature, $K_{\mu \nu }$. $\sigma$ is the tension or bare vacuum energy on the brane. ${\mathcal {L}}_\mathrm{matter}$ is the matter lagrangian.

The two different mass scales give rise to a characteristic scale

260 $$\begin{aligned} r_c\approx \frac{M_4^2 }{M_5^3 }. \end{aligned}$$

At scales shorter than $r_c$, gravity is 4 dimensional and reduces to GR but at scales larger than $r_c$, the 5 dimensional physics is involved and contributes to the dynamics. The field equations can be found in Clifton et al. (2012).

With an FLRW metric for the brane, a Minkowski metric for bulk and zero tension, $\sigma$, one gets first a modified Friedmann equation from the 4D Einstein’s equations (Deffayet 2001; Lue 2003; Dick 2001) as

261 $$\begin{aligned} H^2 + \frac{\kappa }{a^2} - \frac{\epsilon }{r_c}\sqrt{H^2 + \frac{\kappa }{a^2}} = \frac{8\pi G}{3}{\bar{\rho }} , \end{aligned}$$

where $\epsilon =-1$ is for the normal branch, and $\epsilon =1$ is for the self-accelerating branch.

The other components of the Einstein equations combined with (261) above, give the second evolution equations as

262 $$\begin{aligned} 2\frac{dH}{dt} + 3 H^2 + \frac{\kappa }{a^2} = -\frac{ 3 H^2 + \frac{3\kappa }{a^2} - 2 \epsilon r_c \sqrt{H^2 + \frac{\kappa }{a^2}} \; 8\pi G P}{1 - 2 \epsilon r_c \sqrt{H^2 + \frac{\kappa }{a^2}} } . \end{aligned}$$

Although clearly a modified gravity model, the DGP can be formulated using state variables of an effective dark energy

263 $$\begin{aligned} 8\pi G \rho _{E}= & {} \frac{3\epsilon }{r_c} \sqrt{H^2 + \frac{\kappa }{a^2}} , \end{aligned}$$

264 $$\begin{aligned} 8 \pi G P_{E}= & {} -\epsilon \frac{ \frac{dH}{dt} + 3 H^2 + \frac{2\kappa }{a^2} }{ r_c \sqrt{H^2 + \frac{\kappa }{a^2} }}, \end{aligned}$$

and an effective equation of state,

265 $$\begin{aligned} w_{DGP} = \frac{P_E}{\rho _E} = -\frac{ \frac{dH}{dt} + 3 H^2 + \frac{2\kappa }{a^2}}{ 3H^2 + \frac{3\kappa }{a^2} }, \end{aligned}$$

satisfying the conservation law.

$$\begin{aligned} \frac{d\rho _E}{dt} +3H (\rho _E + P_E) \equiv 0. \end{aligned}$$

During the late time self-accelerating epoch $w_E \rightarrow -1$ in (265), mimicking a cosmological constant.

Cosmological linear perturbations for DGP have been worked out in Deffayet (2002). Koyama and Maartens (2006) assumed the small-scale (quasi-static) approximation $k/a\gg r_c \mathcal {H}$ and obtained

266 $$\begin{aligned} - k^2 {\varPhi } = 4 \pi G \left( 1 - \frac{1}{3\beta }\right) {\bar{\rho }} a^2 \delta _M, \end{aligned}$$

and

267 $$\begin{aligned} - k^2 \varPsi = 4 \pi G \left( 1 + \frac{1}{3\beta }\right) {\bar{\rho }} a^2 \delta _M, \end{aligned}$$

where $\beta = 1 + 2 \epsilon H r_c w_E$. It was shown in a number of studies that, unlike the normal branch, the self-accelerating branch of the DGP theory suffers from ghost instabilities (Luty et al. 2003; Charmousis et al. 2006; Gregory et al. 2007; Gorbunov et al. 2006; Koyama 2007).

It is also informative, for comparison with observations, to note a result from Wei (2008) and Ferreira and Skordis (2010) that the linear growth rate index parameter for the DGP model is given by

268 $$\begin{aligned} \gamma = \frac{11}{16} - \frac{7}{5632} \varOmega _{\mathrm{DGP}} + \frac{93}{4096}\varOmega _{\mathrm{DGP}}^2 + O(\varOmega _{\mathrm{DGP}}^3). \end{aligned}$$

Nonlinear growth and simulations in DGP models include the works of Lue et al. (2004), Koyama et al. (2009), Scoccimarro (2009), Chan and Scoccimarro (2009), Schmidt (2009a, b), Khoury and Wyman (2009), Wyman and Khoury (2010), Schmidt et al. (2010), Seahra and Hu (2010), Chan and Scoccimarro (2009), Winther et al. (2017) and Bose et al. (2018).

While the DGP self-accelerating branch models have been now excluded by observation (see Sect. 9.6) and have been shown to be plagued by ghost instabilities, interest continues in the stable normal branch which is often referred to as the nDGP model. Models in this branch are not self-accelerating since the acceleration is due to the brane tension term playing the role of a cosmological constant. nDGP are used as benchmark to develop and test frameworks for MG studies as in for example the nonlinear regime, e.g., Hellwing et al. (2017) and Bose et al. (2018).

### Non-local modified gravity theories

A different approach to gravity has been undertaken sometime ago with the idea to introduce some non-locality aspects. A recent class of such theories is where the fundamental action of gravity is local, but the corresponding quantum effective action is not. Non-local gravity in the recent context of cosmic acceleration has been introduced by Wetterich (1998) with the additional term R$\Box ^{-1}R$ to the Einstein–Hilbert action. Despite interesting features and being ghost-free, the model did not have a viable cosmological evolution. This was followed by a generalization proposed by Deser and Woodard (2007) that made the additional term as $R f(\Box ^{-1}R)$. It is possible to adjust their model so it can fit well the background accelerating expansion with no need for a dark energy component (Woodard 2014), although in a non-predictive way state (Belgacem et al. 2018b). Cosmological perturbations and growth of structure equations have been worked out and the model compared to large scale structure data (Park and Dodelson 2013; Nersisyan et al. 2017; Park 2017). It was found in Park and Dodelson (2013) that the $R f(\Box ^{-1}R)$ is not in agreement with such large-scale structure data but Nersisyan et al. (2017) found the opposite and reported that the model are consistent with such data. This was confirmed in Park (2017) so it is agreed now that the model is consistent with the growth of large-scale structure data. For the moment, the model has been compared only to structure formation data and need further comparison to full CMB and other data sets. As stated in Woodard (2014), their model paved the road for further developments in non-local gravity. A good discussion of non-local gravity and its cosmology was also given in Koivisto (2008) where models similar to Deser and Woodard (2007) were carefully analyzed. The author found that even simple models can drive late-time cosmic acceleration without affecting early time cosmology. Furthermore, Barvinsky proposed a theory with an additional term of the form $R^{\mu \nu }\square ^{-1}G_{\mu \nu }$ (Barvinsky 2003, 2012a, b). The author connects the theory he proposed to the paradigms of dark matter and dark energy. It is hoped to see more detailed developments of these models with observable functions and comparison to data. Most recently, Maggiore and Mancarella (2014) followed the path of non-local gravity but with a different approach where a new mass scale is generated in the IR limit and is associated with the non-local term in the theory. Their theory provides an interesting phenomenology for cosmic acceleration and is found to fit current observations (Maggiore and Mancarella 2014; Maggiore 2014).

#### Illustrative example: ${ RR }$ model

We use here for illustration, the specific model called “${ RR }$” that was proposed in Maggiore and Mancarella (2014) and was based on their earlier work of Maggiore (2014). The quantum effective action derived from the fundamental Einstein–Hilbert action is postulated to have the form

269 $$\begin{aligned} \varGamma _{{{RR}}}=\frac{m_{\mathrm{Pl}}^2}{ 2}\int d^4 x {\sqrt{ -g}}\,\,\left[ R-\frac{1}{6} m^2 R\frac{ 1}{\Box ^2 } R\right] , \end{aligned}$$

where the nonlocal term is assumed to capture non-perturbative infrared effects due to quantum fluctuations, and corresponds, physically, to a dynamical mass generation for the conformal mode. Here $m_{\mathrm{Pl}}^{2}$ is the reduced Planck mass squared and *m* is a mass parameter related to the generated fundamental mass scale $\varLambda _{{RR}}$ by $\varLambda _{{RR}}^4=(1/12)m^2m_{\mathrm{Pl}}^2$. The model has been reviewed in some detail in Maggiore (2016) and Belgacem et al. (2018b) with comparison to available cosmological data in Dirian et al. (2014, 2015, 2016a) and Dirian (2017) as we summarize further below.

Recently, evidence for the emergence of the nonlocal term in (269) has been found by using nonperturbative results from lattice gravity Knorr and Saueressig (2018). Also, as pointed out in Maggiore and Mancarella (2014), analogous nonlocal terms, proportional to $m^2 F^{\mu \nu } \Box ^{-1} F_{\mu \nu }$, have also been postulated to arise in the quantum effective action of QCD, where they reproduce results for the gluon propagator from lattice simulations (Boucaud et al. 2001; Capri et al. 2005; Dudal et al. 2008).

We present the model dynamics and cosmology following (Maggiore 2016; Belgacem et al. 2018b). The model can be written in a local form by the use of two auxiliary fields *U* and *S*, (Maggiore and Mancarella 2014)

270 $$\begin{aligned} U=-\square ^{-1}R\, ,\qquad S=-\square ^{-1}U. \end{aligned}$$

The action can then be re-written by the use of the two Lagrange multipliers $\xi _1,\xi _2$ as follows (Maggiore and Mancarella 2014)

$$\begin{aligned} \varGamma _{{{RR}}}=\frac{m_{\mathrm{Pl}}^2}{2}\int d^4x \sqrt{-g}\, \left[ R\left( 1-\frac{m^2}{6} S\right) -\xi _1(\Box U+R)-\xi _2 (\Box S+U) \right] . \end{aligned}$$

Variation with respect to the metric gives the field equations

271 $$\begin{aligned} G_{\mu \nu }=\frac{m^2}{6} K_{\mu \nu }+8\pi GT_{\mu \nu }\, , \end{aligned}$$

where the tensor $K_{\mu \nu }$ is given in terms of the metric and the auxiliary fields as (Maggiore and Mancarella 2014)

272 $$\begin{aligned} K^\mu _\nu \equiv 2 S G^\mu _\nu - 2 \nabla ^\mu \partial _\nu S + 2 \delta ^\mu _\nu \Box _g S + \delta ^\mu _\nu \partial _\rho S \partial ^\rho U - \frac{1}{2} \delta ^\mu _\nu U^2 - \big ( \partial ^\mu S \partial _\nu U + \partial _\nu S \partial ^\mu U \big ). \end{aligned}$$

Variation with respect to the Lagrange multipliers $\xi _1,\xi _2$ gives the further equations that *U* and *S* must satisfy

273 $$\begin{aligned} \Box U=-R\, ,\qquad \Box S =-U. \end{aligned}$$

This localization thus makes the theory appear as a scalar–tensor theory with two dynamical fields *U* and *S*. However, as discussed in Belgacem et al. (2018b) and references therein, upon quantization, the theory remains ghost-free because there are no quanta associated to these two fields. In fact, the classical instability develops rather on a cosmological timescale producing the late cosmic acceleration with an effective phantom-like dark energy component that is found to be consistent with cosmological background and growth of structure observations.

Again following Belgacem et al. (2018b), we summarize some aspects of ${ RR }$ model cosmology. The field equations (271)–(273) are applied to the flat FLRW metric in Cartesian coordinates

274 $$\begin{aligned} ds^2=-dt^2+a^2(t)d\mathbf x ^2 \end{aligned}$$

and the time evolution is parametrized using $x\equiv \ln a$. In addition to *U*(*x*) and *S*(*x*), the following dimensionless functions are defined

275 $$\begin{aligned} W(x)\equiv H^2(x)S(x),\,\,\,\,h(x)\equiv H(x)/H_0 \end{aligned}$$

where $H(t)=\dot{a}/a$ is the Hubble function with $H_0$ its present value. The background evolution equations are then obtained as (Maggiore and Mancarella 2014)

276 $$\begin{aligned}&h^2(x)=\varOmega _M e^{-3x}+\varOmega _R e^{-4x}+\gamma Y \end{aligned}$$

277 $$\begin{aligned}&U''+(3+\zeta ) U'=6(2+\zeta )\, ,\end{aligned}$$

278 $$\begin{aligned}&W''+3(1-\zeta ) W'-2(\zeta '+3\zeta -\zeta ^2)W= U\, , \end{aligned}$$

where prime here denotes differentiation with respect to *x*, $\gamma \equiv m^2/(9H_0^2)$, $\zeta \equiv h'/h$ and

279 $$\begin{aligned} Y\equiv \frac{1}{2}W'(6-U') +W (3-6\zeta +\zeta U')+\frac{1}{4}U^2. \end{aligned}$$

In the modified Friedmann equation (276), an effective dark-energy density is identified as $\rho _{\mathrm{DE}}=\rho _0\gamma Y$ where $\rho _0=3H_0^2/(8\pi G)$ is the usual critical density given in (15).

As discussed in Maggiore and Mancarella (2014) and Belgacem et al. (2018b), a choice of boundary conditions can be made for the auxiliary fields *U* and *W* that can provide a minimal model with a background evolution that depends on the Hubble constant, $H_0$ the matter density parameter $\varOmega _M$, and one additional parameter that is the mass *m* that replaces the cosmological constant. So the model has the same background parameters as the $\varLambda$CDM model. Interestingly, just like for $\varOmega _{\varLambda }$ in the $\varLambda$CDM model, in the ${ RR }$ model, the flatness condition allows one to derive the mass parameter *m*. The authors then proceeded to fit this minimal model to CMB, BAO, and SN data and found $\varOmega _M\simeq 0.299$ and $h_0\simeq 0.695$ (Dirian 2017). With these values fixed, they then integrated numerically (276)–(278) (Maggiore and Mancarella 2014) and studied the evolution of the corresponding effective dark energy equation of state, $w_{\mathrm{DE}}(x)$. Translating their results into the common CPL parameterization (Chevallier and Polarski 2001; Linder 2003), they found that the model has an effective phantom-like equation of state with $w_0\simeq -1.15$ and $w_a\simeq 0.09$ (Maggiore and Mancarella 2014).

Belgacem et al. (2018b) followed with an interesting discussion relating the evolution of the field *U* to that of the effective dark energy of the model. While it is zero in the radiation dominated era, the field *U* grows in the matter dominated era resulting in the growth of the effective dark energy density as indicated by (279). Moreover, this implies that both the effective dark energy density and its variation are positive resulting in a phantom effective equation of state caused by the ghost-like field *U*. The good news is that the ghost-like feature of the field in this case is only classical with no associated quanta nor instability when quantizing the theory. On the contrary, the classical instability is a plus and would be responsible for the observed late-time cosmic acceleration.

Belgacem et al. (2018b) compared the comoving distances between the minimal ${ RR }$ model and the $\varLambda$CDM and found that for the same values of a fiducial cosmological model these can be different by up to 2.5%, however, if each models is fed its best-fit values of cosmological parameters, the difference can be brought down to below 1% up to redshift 6 (see Fig. 2 there).

Discussions of ${ RR }$ models with other initial conditions than the minimal models discussed above can be found in Maggiore (2016), Belgacem et al. (2016) and Maggiore (2014). It was found that some models can mimic the background with an equation of state that is very close to that a cosmological constant and different from it by less than a 1%, on the phantom side again. This will make it challenging to distinguish such particular models from the $\varLambda$CDM model using observations, although the growth of structure remain to be explored.

Cosmological perturbations for ${ RR }$ models have been worked out in detail in Dirian et al. (2014). They have been recapitulated in Maggiore (2016) and Belgacem et al. (2018b) and we refer the reader to those papers. We provide here a few remarks following Belgacem et al. (2018b). From using the flat FLRW perturbed metric in the Newtonian gauge plus linear perturbations of the auxiliary fields *U* and *W*, with adiabatic initial conditions, the resulting perturbation evolutions were found to be stable (Dirian et al. 2014). They also found in their analysis that, for the minimal ${ RR }$ model, the perturbations are close to those of the $\varLambda$CDM model with a difference below 10% (Dirian et al. 2014). This makes the comparison to data interesting in a sense that it is close to the $\varLambda$CDM and so it is expected to be found in a viable range, but it is also distinct from the $\varLambda$CDM so a comparison to find which model fits better the data will be possible and important.

#### Other non-local gravity theories

Another interesting proposal of non-local gravity is that of Mashhoon, see e.g. Mashhoon (1990, 2008) and Hehl and Mashhoon (2009a, b). Recently, the authors applied the theory to Newtonian cosmology with the aim to model structure formation without the need for dark matter (Chicone and Mashhoon 2016). It will be interesting to see this theory tested using large scale structure and CMB data. A review can be found in Mashhoon (2017).

## Screening mechanisms

Most recent developments and proposals of MG models have been motivated by the problem of cosmic acceleration. Modifications to GR happen in a way to affect cosmological dynamics at large scales and to produce an accelerating expansion. However, any such modification at cosmological scales must survive well-established stringent solar system tests of gravity (Will 2014).

Therefore, MG models must either reduce to GR at small scales, by construction, or must have a mechanism that suppresses any deviation from GR at small scales. These are known as screening mechanisms. Some of them are based on ideas that relate the scalar field potential to the local matter density within planetary systems or galaxies since it is higher than the average cosmological density.

Most MG models generate a fifth force acting at the level of perturbations due to the coupling of the scalar field to the matter in the Einstein frame. We use here the behavior of factors or components of such a fifth force potential to classify the corresponding screening mechanism following one of the classifications of, e.g., de Rham (2012), Jain et al. (2013a) and Joyce et al. (2015). For that, we consider a simplified lagrangian for a scalar field conformally coupled to matter as

280 $$\begin{aligned} {\mathcal {L}} = -\frac{1}{2}Z^{\mu \nu }(\phi , \partial \phi ,\ldots )\partial _\mu \phi \partial _\nu \phi -V(\phi )+\beta (\phi )T^\mu _{\;\mu }~, \end{aligned}$$

where the components of $Z^{\mu \nu }$ contain functions up to second order derivatives associated with self-interactions of the field, and $\beta (\phi )$ is a coupling function to the trace of the energy-momentum tensor, $T^\mu _{\;\mu }$. We can consider non-relativistic pressure-less sources and specifically a point source so that $T^\mu _{\;\mu }=-\rho =-{\mathcal {M}}\delta ^3(\varvec{x})$. The background value of the field, ${\bar{\phi }}$, is determined by this local density. We then consider a field perturbation, $\delta \phi$, around the background value, ${\bar{\phi }}$, with an equation of motion given by

281 $$\begin{aligned} Z({\bar{\phi }})\Big (\ddot{\delta \phi }-c_s^2({\bar{\phi }})\nabla ^2\delta \phi \Big )+m^2({\bar{\phi }})\delta \phi = \beta ({\bar{\phi }}){\mathcal {M}} \delta ^3(\varvec{x}), \end{aligned}$$

where $c_s$ is an effective sound speed of perturbations, $m({\bar{\phi }})$ is the scalar effective mass. Assuming negligible spatial variations for the background field over the scales of interest, the corresponding potential is given by Joyce et al. (2015)

282 $$\begin{aligned} V(r) = -\frac{\beta ^2({\bar{\phi }})}{Z({\bar{\phi }}) c_s^2({\bar{\phi }})}\frac{e^{-\frac{m({\bar{\phi }})}{\sqrt{Z({\bar{\phi }})}c_s({\bar{\phi }})}r}}{4\pi r}{\mathcal {M}} ~. \end{aligned}$$

This Yukawa potential corresponds to a fifth force. This force is Yukawa-suppressed (via the exponential) at some large scales but needs to be suppressed at small scales such as the solar systems and inside galaxies. There are at least three mechanisms to produce such a screening in high density environments and to produce dynamics that complies with local tests of gravity and also do not perturb star motions and distributions in galaxies. We briefly overview them in the next subsections and refer the reader to specific papers and reviews on each mechanism. The topic of screening is extensively covered in the reviews Joyce et al. (2015), Khoury (2010), Jain and Khoury (2010) and Burrage and Sakstein (2018). It was pointed out in (Burrage and Sakstein 2018) that Eq. (282) captures very well how screening may happen in interactions between fundamental particles, but is not particularly effective in explaining how screening happens around more macroscopic sources. For example they explain that the thin-shell mechanisms of chameleon and symmetron models that we will discuss further below are much better described in this equation by making curly M in Eq. (282) as a function of the true mass of the source like in Eq. 2.11 in Burrage and Sakstein (2018).

We use below a classification based on the MG model fifth force, however, other classifications have been proposed. See the review Joyce et al. (2015) for two other classifications of screening mechanisms. One they qualify as more phenomenological and more suited for astrophysical and cosmological observations. It is based on classifying the screening mechanisms in three types where the screening is set by the field itself, its first derivatives, or its second derivatives. The last classification they provide there is based on an effective field theory approach that they present as a unifying description for these mechanisms. The authors provide a large number of examples and organize their review around such screening mechanisms and we refer the reader to their review and references therein.

### Large-mass based screening

In this case, the scalar field mass, $m({\bar{\phi }})$ depends on the environment and causes the change. In a high density region, the scalar field acquires a large mass so the fifth force becomes very short range and highly suppressed as can be seen from the potential (282). At the opposite, in a low density region such as cosmological volume scales, the scalar field becomes light and the fifth force reaches the strength of the gravitational force manifesting itself in the growth perturbation equations (see Sect. 3.2). An example of a field with such a behavior is the aptly named chameleon field (Khoury and Weltman 2004b, a). See also the recent review Burrage and Sakstein (2018) and references therein. We review some aspects of it following Khoury and Weltman (2004a) and Koyama (2016).

We consider a class of MG models using the chameleon mechanism and for which the action can be written in the following form in the Einstein frame

283 $$\begin{aligned} S= \int d^4 x \sqrt{-g} \left[ \frac{1}{16 \pi G} R - \frac{1}{2} (\nabla \phi )^2 - V(\phi ) \right] + S_{m} ( A^2(\phi ) g_{\mu \nu },\psi _m) \end{aligned}$$

where the field is coupled to the matter via the metric $A^2(\phi ) g_{\mu \nu }$. The matter particles do not follow geodesics in this frame and feel a fifth force generated by the scalar field as

284 $$\begin{aligned} F_5=\nabla \ln A(\phi ). \end{aligned}$$

The scalar field dynamics are governed by an effective potential that depends on the local density $T^{\mu }_{\mu }=-\rho$ as

285 $$\begin{aligned} V_{\mathrm{eff}} = V(\phi ) +[A(\phi )-1]\rho . \end{aligned}$$

An example of a typical choice of the potential $V(\phi )$ and the coupling function $A(\phi )$ for the chameleon mechanisms is given by

286 $$\begin{aligned} A(\phi )= & {} 1 + \xi \frac{\phi }{M_{\mathrm{pl}}}, \quad V(\phi ) = \frac{M^{4 +n}}{\phi ^n}. \end{aligned}$$

where *M* is the mass scale parameter. The scalar field dynamics are characterized by the coupling function

287 $$\begin{aligned} \beta =M_{\mathrm{Pl}}\frac{d \ln A}{d \phi } \big |_{\phi =\bar{\phi }} \end{aligned}$$

and its mass around the minimum of the potential $\phi =\bar{\phi }$ given by

288 $$\begin{aligned} m^2 = \left. \frac{d^2V_{\mathrm{eff}}}{d\phi ^2} \right| _{\phi =\bar{\phi }} \end{aligned}$$

Thus the dependence of the scalar field mass on its environment will be determined by an appropriate choice of the potential $V(\phi )$. For example the potential(285) depends explicitly on the density.

To understand a little bit better how this is implemented, let one recall the equation of motion of the scalar field given by

289 $$\begin{aligned} \nabla ^2 \phi = V_{\mathrm{eff,\phi }}( \phi )= V_{,\phi }(\phi ) + 8 \pi G \beta \rho . \end{aligned}$$

Now, following Khoury and Weltman (2004a), one considers a spherically symmetric body with radius $R_c$ and homogeneous density $\rho _c$. The body is assumed to be embedded in a larger environment with homogeneous smaller density $\rho _{\infty }$. This is like the solar system in the galaxy, or a galaxy in the Hubble volume. For the spherically symmetric body, the Eq. (289) becomes

290 $$\begin{aligned} \frac{d^2\phi }{dr^2} + \frac{2}{r} \frac{d\phi }{dr} = V_{,\phi }(\phi ) + 8 \pi G \beta \rho . \end{aligned}$$

with $\rho (r)=\rho _c$ for $r<R_c$ and $\rho (r)=\rho _{\infty }$ for $r>R_c$.

A qualitative discussion followed by a quantitative derivation of the solution to (290) taking into account proper boundary conditions is carried out in some detail in Khoury and Weltman (2004a) giving

291 $$\begin{aligned} \phi (r) = - \left( \frac{3 \delta R_c}{R_c} \right) \frac{2 G M \beta }{r} e^{- m_{\infty } r} + \phi _{\infty }, \end{aligned}$$

where

292 $$\begin{aligned} \frac{\delta R_c}{R_c} = \frac{\phi _{\infty } - \phi _{s}}{6 \beta M_{\mathrm{Pl}}|\varPsi _{N}| } \ll 1, \end{aligned}$$

where $\phi _s$ is the field value which minimizes $V_{\mathrm{eff}}$ inside the source, $\phi _{\infty }$ is another minimum outside the source, $r_{scr}$ delimits the screened area, $R_c$ is the radius outside $r_{scr}$, and $\varPsi _{N}$ is the gravitational potential of the source with $|\varPsi _{N}| = G M/R_c$.

This solution is valid under the thin shell condition (Khoury and Weltman 2004a),

293 $$\begin{aligned} \delta R_c/R_c \ll 1. \end{aligned}$$

**Fig. 15:**
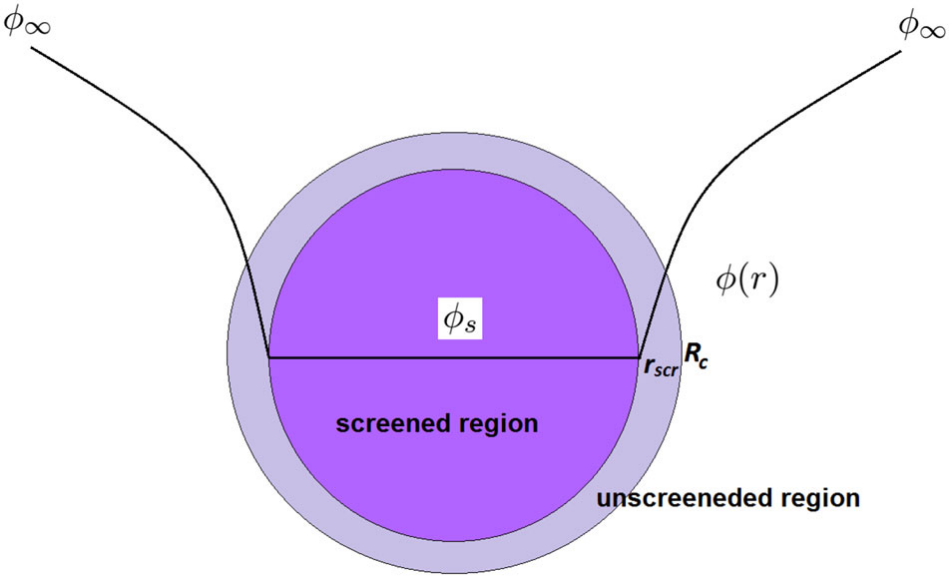
Thin shell profile of the scalar field for the chameleon screening mechanism. Only the mass of the thin shell contributes to the fifth force outside the shell while the force is suppressed inside the shell. See text and Khoury and Weltman (2004a) and Koyama (2016) for discussion

In such a case, only the mass within the thin-shell defined by $\delta R_c$ contributes to the fifth force outside the shell because in the interior of the source the scalar field mass is large and the fifth force is suppressed by the Yukawa exponential realizing the chamelon mechanism. This brings us to an important point stressed in Khoury and Weltman (2004a) and Koyama (2016) which is the gravitational potential profile from a dense region to a less dense region that matters rather than the dense region alone. We depict in Fig. 15 the above picture for the chameleon thin-shell mechanism.

We conclude this sub-section by giving some informative constraints on the chameleon scalar field that can be obtained from applying the screening mechanism and the thin shell condition to the solar system, galactic scale and cosmic scale following Khoury and Weltman (2004a) and Koyama (2016). In order to compare with observations though, we need to consider the Jordan frame where the MG parameters then relate to the coupling parameter $\beta$ in the thin shell condition as follows:

294 $$\begin{aligned} \mu = 1 + 2 \beta _{\mathrm{eff}}^2 \quad \mathrm {and } \quad \eta = \frac{1 - 2 \beta _{\mathrm{eff}}^2}{1 + 2 \beta _{\mathrm{eff}}^2}. \end{aligned}$$

where

295 $$\begin{aligned} \beta _{\mathrm{eff}}^2 \equiv \beta ^2 \frac{3 \delta R}{R}. \end{aligned}$$

For example, taking the sun density as $\rho _{\mathrm{sun}} = 10$ g cm$^{-3}$ and the Milky way density as $\rho _{\mathrm{gal}}=10^{-24}$ g cm$^{-3}$, one can conclude that the scalar field in the vicinity of the sun is suppressed compared to the galaxy. If one assumes the thin shell condition holds and uses Eqs. (294) and (295), one can write $\eta -1 = - 4 \beta _{\mathrm{eff}} ^2$. Now, using Eq. (292), setting $\varPsi _{N \mathrm gal} \approx 10^{-6}$ for the Milky Way’s potential, and employing the constraints $|\eta -1| =(2.1 \pm 2.3) \times 10^{-5}$ (Bertotti et al. 2003), one can derive the constraint on the galaxy scalar field as

296 $$\begin{aligned} \frac{\beta \phi _{\mathrm{gal}}}{M_{\mathrm{Pl}}} < 10^{-11}. \end{aligned}$$

This is a model independent constraint but see (Khoury and Weltman 2004a; Koyama 2016) for model dependent stringent constraints on the scalar field in a cosmological environment and many other examples in Khoury and Weltman (2004a). It was also pointed out in Sakstein (2017) that (296) only applies if one has a point particle and that PPN bound on chameleon screening is actually weaker than previously claimed due to the WEP.

We end this sub-section with a significant result from Wang et al. (2012) who proved two no-go theorems limiting the cosmological impact on chameleon-like mechanisms such as one above but also the symmetron (a scalar field that couples to the ambient matter density) and dilaton (defined in Sect. 7.5.1) mechanisms in the next sub-section. The first theorem states that the Compton wavelength (effective “range”) of such a scalar field can be at most of Mpc scale so it limits its impact on large-scale structure reducing it to nonlinear scales only. So it will have no effect on the linear growth rate of large scale structure and its observables such as RSD, WL or clustering. The second theorem states that, in these theories, the conformal factor relating the Einstein and the Jordan frames and their scale factors is essentially constant in a Hubble time meaning that such theories cannot provide self-acceleration and rather require a form of dark energy for that. In other words, a broad class of chameleon, symmetron, and dilaton screened theories cannot have any significant effect on large scale density perturbations and cannot explain cosmic acceleration. This was quite a strong result leaving then only kinetic-terms based screening mechanisms such as Vainshtein and k-mouflage, discussed further below, for consideration.

### Weak-coupling based screening

In this mechanism, it is the field’s coupling $\beta (\phi )$ in Eq. (282) that depends on the environment. In a dense region such as the solar system, it becomes weak and causes the suppression of the fifth force. However, in low density environments such as at cosmological scales, the coupling strengthens and makes the fifth force of the order of the gravitational force affecting Poisson equations as in Sect. 3.2.

Examples of fields using this mechanism are the dilaton (Damour and Polyakov 1994; Brax et al. 2011) with a typical choice of the potential and coupling functions given by

297 $$\begin{aligned} A(\phi )= & {} 1 + \frac{1}{2 M} (\phi - \bar{\phi })^2, \quad V(\phi ) = V_0 e^{- \phi /M_{\mathrm{pl}}} \end{aligned}$$

and the symmetron (Hinterbichler and Khoury 2010; Olive and Pospelov 2008) with

298 $$\begin{aligned} A(\phi )= & {} 1 + \frac{1}{2 M^2} \phi ^2, \quad V(\phi ) = -\frac{\mu ^2}{2} \phi ^2 + \frac{\lambda }{4} \phi ^4 , \end{aligned}$$

where the action and effective potentials are given by Eqs. (283) and (285), respectively. We refer the reader to the original papers above for these mechanisms and the specialized review (Joyce et al. 2015).

### Large kinetic terms based screening

Another possibility is to make the kinetic function $Z({\bar{\phi }})$ in Eq. (282) dependent on the environment. These are in particular derivatives of the field corresponding to its nonlinear interactions. When such terms becomes large, they can effectively suppress the fifth force as can be seen in (282). Namely, this can happen when the first derivatives of the field become large as in the case of the k-mouflage mechanism (Babichev et al. 2009), see also reviews Brax and Valageas (2014, 2016), or when the second derivatives become important realizing the Vainshtein mechanism (Vainshtein 1972), see also review Joyce et al. (2015).

A typical choice of action that leads to the k-mouflage mechanism is of the form of the Horndeski class of models (146) with only

299 $$\begin{aligned} {\mathcal {L}}_{2} = K(\phi ,X)=X + \frac{\alpha }{4 \varLambda ^4} X^2. \end{aligned}$$

If one considers a solution to a spherically symmetric field around a source with a given gravitational potential then the k-mouflage screening occurs when the first derivative of the gravitational potential exceeds some critical value $\varLambda _c$. We chose the form of (299) just for illustrative purposes as it was shown in Barreira et al. (2015) that it does not pass some solar system and cosmological constraints.

Vainshtein mechanisms can be realized by the typical choice of the Horndeski action with only

300 $$\begin{aligned} {\mathcal {L}}_{2}= & {} K(\phi ,X)=X,\nonumber \\ {\mathcal {L}}_{3}= & {} -G_{3}(\phi ,X)\Box \phi = \frac{1}{\varLambda ^3} X \Box \phi . \end{aligned}$$

The Vainshtein screening occurs when the second derivatives of the gravitational potential exceed some critical value $\varLambda _c^3$.

We provide here a simple illustrative example of how the Vainshtein mechanism works. Following Joyce et al. (2015), we use the cubic Galileon with Lagrangian:

301 $$\begin{aligned} {\mathcal {L}} = -3(\partial \phi )^2 - \frac{1}{\varLambda ^3}\Box \phi (\partial \phi )^2 +\frac{g}{M_{\mathrm{Pl}}}\phi T^\mu _{\;\mu }\ , \end{aligned}$$

where gravitational strength coupling, *g*, is taken of the order unity and $\varLambda$ is the strong-coupling scale of the theory. The Vainshtein mechanism is realized by the $(\partial \phi )^2\Box \phi /\varLambda ^3$ term becoming large compared to the term $(\partial \phi )^2$ near massive objects so that $\partial ^2\phi \gg \varLambda ^3$ is achieved. Varying (301) with respect to $\phi$ gives the equation of motion

302 $$\begin{aligned} 6\Box \phi + \frac{2}{\varLambda ^3} \bigg ( (\Box \phi )^2 - (\partial _\mu \partial _\nu \phi )^2\bigg ) = - \frac{g}{M_{\mathrm{Pl}}} T^\mu _{\;\mu }. \end{aligned}$$

Next, the field is considered around a static point source with $T^\mu _{\;\mu } =-M\delta ^{(3)}(\varvec{x})$ and assumed to have a static spherically-symmetric profile, $\phi (r)$. Equation (302) then becomes  (Nicolis and Rattazzi 2004)

303 $$\begin{aligned} \varvec{\nabla } \cdot \left( 6\mathbf {\nabla }\phi + \hat{r} \frac{4}{\varLambda ^3}\frac{(\mathbf {\nabla }\phi )^2}{r}\right) = \frac{gM}{M_{\mathrm{Pl}}} \delta ^{(3)}(\varvec{x}). \end{aligned}$$

Upon integration, one obtains

304 $$\begin{aligned} 6\phi ' + \frac{4}{\varLambda ^3}\frac{\phi '^2}{r} = \frac{gM}{4\pi r^2 M_{\mathrm{Pl}}}\, . \end{aligned}$$

One can then solve this equation algebraically for $\phi '$ and use the stable solution for which $\phi '\rightarrow 0$ at $r \rightarrow \infty$. This reads

305 $$\begin{aligned} \phi '(r) = \frac{3\varLambda ^3 r}{4} \left( -1+ \sqrt{1 + \frac{1}{9\pi }\left( \frac{r_{\mathrm{V}}}{r}\right) ^3 }\right) \, , \end{aligned}$$

where

306 $$\begin{aligned} r_{\mathrm{V}} \equiv \frac{1}{\varLambda } \left( \frac{gM}{M_{\mathrm{Pl}}}\right) ^{1/3}\ \end{aligned}$$

is the Vainshtein radius.

**Fig. 16:**
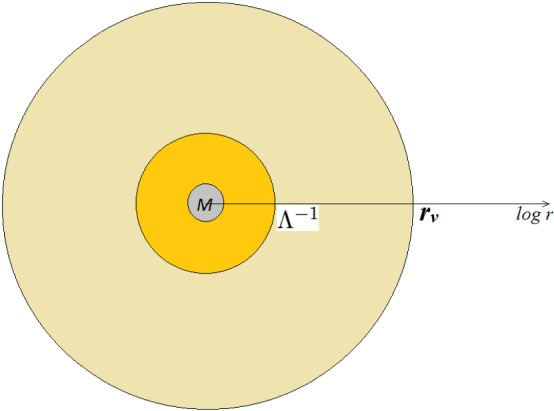
Overall picture of the Vainshtein screening mechanism. There are three regions of interest: ($r\gg r_v$) far outside the Vainshtein radius and away from the source, the fifth force is not screened; ($r \ll r_v$) near the source and below $r_v$, the fifth force is suppressed (this includes $\varLambda ^{-1}\ll r \ll r_v$). $r=\varLambda ^{-1}$ represent the cut-off scale of the theory and is typically very large compared to the Schwarzschild radius

Again following Joyce et al. (2015), we describe how this profile encodes the functioning of the Vainshtein screening mechanism:

- $r \gg r_{\mathrm{V}}$: Far away from the source, the profile goes approximately as $1/r^2$,
  307 $$\begin{aligned} \phi '(r\gg r_{\mathrm{V}}) \simeq \frac{g}{3} \cdot \frac{M}{8\pi M_{\mathrm{Pl}} r^2}. \end{aligned}$$
  The ratio of the Galileon scalar force to the gravitational force is given by
  308 $$\begin{aligned} \left. \frac{F_{\phi }}{F_{\mathrm{gravity}}}\right| _{r\gg r_{\mathrm{V}}}\simeq \frac{g^2}{3}. \end{aligned}$$
  So the gravitational force is enhanced as well-known for DGP models for example.
- $r \ll r_{\mathrm{V}}$: close to the source, the profile  (305) is given by
  309 $$\begin{aligned} \phi '(r\ll r_{\mathrm{V}}) \simeq \frac{\varLambda ^3r_{\mathrm{V}}}{2} \sqrt{\frac{r_{\mathrm{V}}}{r}}\sim \frac{1}{\sqrt{r}}. \end{aligned}$$
  The ratio of the galilean force to the gravitational force is given by
  310 $$\begin{aligned} \left. \frac{F_{\phi }}{F_{\mathrm{gravity}}}\right| _{r\ll r_{\mathrm{V}}} \sim \left( \frac{r}{r_{\mathrm{V}}}\right) ^{3/2}\ll 1, \end{aligned}$$
  so the fifth force is strongly suppressed at distances much smaller than the Vainshtein radius. We provide a schematic picture of the Vainshtein mechanism in Fig. 16.

It is worth ending this section by pointing out a number of papers that have used astrophysical constraints on screening mechanisms some practically ruling out chameleon mechanisms or putting constraints on Vainshtein mechanisms, e.g., Salzano et al. (2017). Studies also provided forecasts on such constraints from future surveys and space-missions including (Sakstein 2017).

## Constraints on MG models from current cosmological data sets

### Constraints on Horndeski and beyond Horndeski models

Planck2015MG used the $\alpha _x$-parameterization of Sect. 7.3.1 in order to put constraints on Horndeski models with a number of restrictions to reduce the number of parameters. This is necessary in view of the relatively limited constraining power of current data sets. They used EFTCAMB (Hu et al. 2014b) so they adapted the parameterization accordingly.

They considered Horndeski models with $\alpha _M = - \alpha _B$, $\alpha _T = \alpha _H = 0$, and $\alpha _K$ fixed by setting $M_2=0$ in Eqs. (149)–(153). So they simply considered non-minimally coupled K-essence type models as in Bellini and Sawicki (2014) with the only free function being $\alpha _M$. As discussed in Sect. 7.3.1, a non-zero $\alpha _M$ parameter represents a non-zero anisotropic stress and a modification of the lensing potential. Additionally, Planck2015MG used the ansatz,

311 $$\begin{aligned} \alpha _M = \alpha _M^{\mathrm{today}} a^\beta \end{aligned}$$

where $\alpha _M^{\mathrm{today}}$ is a constant and $\beta >0$ determines its backward time evolution. In the minimal model they considered, $\alpha _M$ is related to the EFT function $\varOmega (a)$ which after integration gives (Ade et al. 2016b)

312 $$\begin{aligned} \varOmega (a) = \exp \left\{ \frac{\alpha _M^{\mathrm{today}}}{\beta } a^\beta \right\} - 1. \end{aligned}$$

They called this the exponential EFT model while they called a second model with $\beta =1$ and $\varOmega (a)=\varOmega _0\,a$ the linear EFT model. In their Table 5, they give their results from where we extract the following representative constraints:

- For the linear EFT case: $\alpha _M^{\mathrm{today}} < 0.052$ (95% confidence level) for the Planck TT $+$ lowP data set combination and $\alpha _M^{\mathrm{today}} < 0.043$ (95% confidence level) for the Planck TT $+$ TE $+$ EE $+$ BSH data set combination (BSH standing again for BAO, SN and local Hubble constraints).
- For the exponential EFT case: $\alpha _M^{\mathrm{today}} < 0.063$ and $\beta =0.87^{0.57}_{0.27}$ (95% confidence level) for the TT $+$ lowP data set combination and $\alpha _M^{\mathrm{today}} < 0.062$ and $\beta =0.92^{0.53}_{0.24}$ (95% confidence level) for the TT $+$ TE $+$ EE $+$ BSH data set combination.

With stringent bounds on the $\alpha _M$ and its time evolution index $\beta$ with the $\varLambda$CDM values of 0 and 1, respectively, within those constraints.

We discuss in Sect. 10 how the gravitational-wave (GW) event GW170817 and its electromagnetic counterpart GRB170817A constrained the speed of propagation of GW to be practically equal to the speed of light and thus strongly reduced the number of viable Horndeski models to generalized Brans–Dicke theories and cubic Galileons although the latter are ruled out by ISW observations (see Sect. 9.7). However, it was commented in Peirone et al. (2017b) that Horndeski models with non-trivial modifications to GR remain possible at the level of linear perturbations as they explored it using MG parameters. Furthermore, models that are beyond Horndeski and in particular the more general class of degenerate higher order scalar–tensor theories (DHOST) (see Sect. 7.3.1) provide a much more general class to look for further viable self-accelerating models (Crisostomi and Koyama 2017a). The latter study found DHOST models with late-time self-acceleration. They performed perturbations in the quasi-static limit and showed that the models can satisfy constraints from solar interior structure (Saito et al. 2015; Sakstein 2015a; Babichev et al. 2016) and the GW orbital decay of the Hulse–Taylor pulsar (Beltrán Jiménez et al. 2016b). Sakstein et al. (2017b) studied how the interior of astrophysical bodies and pulsations of stars can be used to test beyond horndeski models. They found that brown dwarfs and Cepheid stars are particularly sensitive to such tests. These beyond Horndeski models will be subject to full cosmological analyses once full CMB analysis tools will become available.

Kreisch and Komatsu (2017) performed a cosmological constraint analysis on Horndeski models that were not ruled out by the implication of the gravitational-wave event GW170817 and its electromagnetic counterpart GRB170817A (i.e., $c_{_{GW}=c}$ so $\alpha _T=0$). They used CMB data from Planck and the joint analysis of the BICEP2/Keck Array and Planck, galaxy clustering data SDSS LRGs, BOSS BAO data, and RSD measurements to constrain the remaining parameters. They modeled the evolution of each parameter with an amplitude (the parameter value today) and an index parameter for a scale factor power law as follows (see Sect. 7.3.1 for further discussion of the $\alpha _x$-parameterization):

- Running rate of the effective Planck mass, $M_*^2$,
  313 $$\begin{aligned} \alpha _{\mathrm{M}}=\frac{d\ln \left( M_*^2\right) }{d\ln a}, \end{aligned}$$
  which they evolved as
  314 $$\begin{aligned} \widetilde{M} = \widetilde{M}_0 a^{\beta } \implies \alpha _{\mathrm{M}} =\widetilde{M}_0 a^{\beta } \frac{\beta }{1+\widetilde{M}_0 a^{\beta }}, \end{aligned}$$
  where $M^2_*/m_0^2 = 1 + \widetilde{M}$ as implemented in the software EFTCAMB they used. $m_0^2$ is the Planck mass so $\widetilde{M}_0$ is the fractional deviation of $M_*^2$ from $m_0^2$ today.
- Kinecity of the scalar field due to the presence of its kinetic terms in the action
  315 $$\begin{aligned} \alpha _{\mathrm{K}} =\alpha ^{\mathrm{K}}_0 a^{\kappa } \end{aligned}$$
- Braiding/mixing of scalar and tensor terms
  316 $$\begin{aligned} \alpha _{\mathrm{B}} =\alpha ^{\mathrm{B}}_0 a^{\xi } \end{aligned}$$

They obtained the following results from using CMB and all the LSS data when fixing the kinecity to $\alpha _{K} = 0.1 a^3$: The friction $\alpha _0^{M}$ has an upper limit of 0.38 when $\alpha _0^{B} \ne 0$ and 0.41 when $\alpha _0^{B} = 0$ (all at the 95% confidence level). They found in the case $\alpha _0^{B} \ne 0$ that the bound excludes GR but in the $\alpha _0^{B} = 0$ case they attributed this to stability constraints imposed by fixing $\alpha _0^{K}=0.1$. They also conclude that the effects of Horndeski theory on primordial B-modes are constrained by CMB and LSS data to be insignificant with 95% confidence.

They caution though that making assumptions on some parameters in Horndeski models can cause dramatic changes in the results on other parameters, and fixing the kinecity is one case of this.

They use the Akaike information criterion (AIC) (Akaike 1974) to compare the two models $\alpha _0^{B} \ne 0$ versus $\alpha _0^{B} = 0$. They found that all the data sets prefer the model with $\alpha _0^{B} = 0$ where the data is then consistent with GR.

It is worth ending this sub-section with some useful remarks about constraining Horndeski models. Before the measurement of $\alpha _T \approx 0$ from double Neutron star merger event (i.e., the GW signal GRB170817 and its electromagnetic counterpart GRB170817A, see Sect. 10), there was too much freedom in the parameter space of the Horndeski models. They can produce a $\varLambda \mathrm {CDM}$ background and large-scale observables while providing self-acceleration with no need for a cosmological constant or dark energy component; see for example Lombriser and Taylor (2015). This degeneracy has now been broken by the GW170817/GRB170817A event as was anticipated in Lombriser and Taylor (2016) (see Fig. 4 therein). With the constraint $a_T=0$, a MG cosmic self-acceleration effect now must manifest itself in LSS observables. Indeed, Lombriser and Lima (2017) showed that a minimal signature such a model must produce in LSS provides a 3-$\sigma$ worse fit than $\varLambda \mathrm {CDM}$ model. They concluded that $\alpha _T=0$ will challenge the self-acceleration from a genuine scalar–tensor modification to GR (i.e., breaking the strong equivalence principle). In beyond-Hordeski models, other free functions are introduced and bring back further degeneracies between, for example, cosmic acceleration and LSS from $\alpha _M$. However, Lombriser and Taylor (2016) discussed how Standard Sirens are not affected by this degeneracy from $\alpha _M$ and should be able to test a self-acceleration at the 5-$\sigma$ level for both Horndeski and beyond-horndeski.

### Constraints on Brans–Dicke theory

The most stringent constraint on Brans–Dicke (BD) theory comes from solar system tests where the Cassini mission put the bound $\omega _{\mathrm{BD}}>40,000$ (Bertotti et al. 2003; Will 2014). However, as argued for example in Avilez and Skordis (2014), the theory can be a sub-category of a more general theory (e.g., Horndeski) that has a screening mechanism that makes it very close to GR at small scales and departs from it at cosmological scales. It is also interesting to obtain independent constraints on the theory at very different scales and times. Avilez and Skordis (2014) used CMB data from Planck, WMAP and SPT and ACT (Ade et al. 2014a; Komatsu et al. 2011; Schaffer et al. 2011; Dunkley et al. 2013), and constraints from Big-Bang Nucleosynthesis (BBN) light element abundances (Iocco et al. 2009). They use initial conditions on the scalar field such that the gravitational constant today on Earth is Newton’s constant, $G_N$. They find then $\omega _{\mathrm{BD}} > 692$ at the 99% CL. They also consider the case where the scalar is free and allowed to vary as a parameter. They find in this case, $\omega _{\mathrm{BD}} > 890$ and $0.981< G_{\mathrm{eff}}/G_N < 1.285$ at the 99% CL. This provided an order of magnitude improvement on previous measurements that were reported in the following analyses (Acquaviva et al. 2005; Wu et al. 2010; Wu and Chen 2010).

### Constraints on vector–tensor and generalized Einstein aether theories

Zuntz et al. (2010) conducted a thorough investigation of Einstein Aether theory finding that while in principle the vector field can source a dark matter component and also cause late-time cosmic acceleration, only the latter was found as a viable possibility. Indeed, they found that using the vector field effective effect as a substitute to dark matter does not fit large scale structure from SDSS DR6 (Adelman-McCarthy et al. 2008) and CMB WMAP7 data (Komatsu et al. 2011). They also found physical problems with such a possibility. On the other hand, an effective dark energy based on the vector field was found consistent with the data.

Some time earlier, Carroll and Lim (2004) expressed the gravitational constant appearing in the effective Friedmann equation of a Lorentz-Violating Vector Field theory as (we follow here the notation of Oost et al. 2018b)

317 $$\begin{aligned} G_{\mathrm{cos}}= \frac{G_{\mathrm{ae}}}{1+\frac{1}{2}(c_1+c_3+3 c_2)} \end{aligned}$$

where

318 $$\begin{aligned} G_{\mathrm{ae}}=G_N \left( 1-\frac{1}{2}(c_1+c_4)\right) \end{aligned}$$

This results in a decrease in the expansion rate with consequences during the big bang nucleosynthesis and will result in weak interactions freezing-out later. This leads to a lower freeze-out temperature and a decrease in the production of the primordial $^4 \mathrm{He}$ and a lower $^4 \mathrm{He}$-to-hydrogen mass ratio (Carroll and Lim 2004). This modifies the abundance of the primordial helium. In order to be consistent with current observations (see, e.g., Izotov et al. 2014; Aver et al. 2015), the cosmological gravitational constant must satisfy the constraint (Oost et al. 2018b)

319 $$\begin{aligned} \left| \frac{G_{\mathrm{cos}}}{G_N} -1 \right| \lesssim \frac{1}{8}. \end{aligned}$$

As we will discuss in Sect. 10, stringent constraints have been obtained on Einstein-Aether theories from the binary neutron star merger event GW170817 and GRB170817A by constraining $c_1=c_3$. Oost et al. (2018b) used higher order expansion of the $c_i$ parameter and combined this GW constraint with other theoretical and observational constraints in order to plot allowed regions in the $c_i$ parameter spaces. The constraints from GW170818/GRB170817B can be summarized as (see Oost et al. 2018a)

320 $$\begin{aligned} |c_1+c_3| < 10^{-15} \end{aligned}$$

and

321 $$\begin{aligned} 0 \lesssim c_1+c_4 \lesssim 2.5 \times 10^{-5},\,\,\,\,\,c_4 \lesssim 0,\,\,\,\,\, 0 \lesssim c_2 \lesssim 0.095. \end{aligned}$$

Other additional constraints from astrophysics and theory within these bounds can be found in Oost et al. (2018a).

De Felice et al. (2017) worked out perturbations for some specific Proca vector–tensor models and compared them to current CMB distance data, BAO, SN, and RSD growth rate data. They found the models to be consistent with the data used and equally (or slightly more) competitive than the $\varLambda$CDM. They found that models fit the expansion data with an effective dark energy equation of state of $w_{\mathrm{DE}}=-1-s$ with $s=0.254^{{}+ 0.118}_{{}-0.097}$ at 95 % confidence level (CL). When the growth data is added to the fit, they obtain, $s=0.16^{+0.08}_{-0.08}$ (95 % CL). It remains interesting to perform a full CMB and large scale structure analysis of the models.

### Constraints on massive gravity and bigravity

Koennig et al. (2014a) considered perturbations of bimetric massive gravity and identified a self-accelerating branch that is consistent with the expansion history and stable to linear perturbations. They call this the infinite-branch of bimetric gravity (IBB) based on the behavior of the ratio of the scale factor in the two metrics. They found that the only models with a stable cosmological evolution are the ones with non vanishing $\beta _0$, $\beta _1$ and $\beta _4$ parameters. Since $\beta _0$ is equivalent to a cosmological constant and they were rather interested in self-accelerating stable models, they restricted the analysis to the $\beta _1$ and $\beta _4$ IBB models. They compared the models, in the quasi-static approximation, to available growth rate data in the form of $f\sigma _8$ from 6dFGS (Beutler et al. 2012), LRG200, LRG60 (Samushia et al. 2012), BOSS (Tojeiro et al. 2012), WiggleZ (Blake et al. 2012), and VIPERS (de la Torre et al. 2013) surveys, as well as the Union 2.1 Compilation of SNe Ia data (Suzuki et al. 2012). They found that the IBB model fits the data with $\varOmega _m^0=0.18$ and an effective dark energy equation of state $w(z)=-0.79+0.21z/(1+z)$. They also found that growth rate of structure in IBB is well-approximated at late times by $f(z)\approx \varOmega _m^{.47}[1+0.21z/(1+z)]$. They found that the combination of growth and supernova data gives the IBB parameter constraints $\beta _1 = 0.48^{+0.05}_{-0.16}$ and $\beta _4 = 0.94^{+0.11}_{-0.51}$, although the strongest constraints come from the supernova data. They also find that the anisotropic stress MG parameter (or slip) in this model tends to 1 / 2 and the gravitational coupling parameter, as they defined it, tends to 4 / 3 at early times and they are different from the GR unity values. These MG parameters will be then a route to test and distinguish between these models and $\varLambda$CDM. Finally, they also found for these models that the usual ansatz used in GR for $f\approx \varOmega _m^{\gamma }$ does not work. It is rather a two-parameter form given by

322 $$\begin{aligned} f\approx \varOmega _{m}^{\gamma _{0}}\left( 1+\alpha \frac{z}{1+z}\right) , \end{aligned}$$

that provides good fit to the growth rate with $\gamma _{0}=0.47$ and $\alpha =0.21$ as best-fit values.

Geng et al. (2017) studied background and linear perturbation evolution for a minimum nontrivial case by setting only $\beta _0$ and $\beta _1$ non-zero so the models are not self-accelerating, see Sect. 7.3.4. As we reported earlier, they found an effective dark energy equation of state of a phantom type. They also compared the matter power obtained to SDSS LRG DR7 finding that it puts stringent constraints on $\bar{\beta }_1$ from Eq. (207) to be $\lesssim \mathcal {O}(10^{-2})$ at the linear perturbation level. Other seminal papers that compared bimetric theory to observations include Akrami et al. (2012) and Enander et al. (2015).

Bigravity models such the IBB above, and others, remain interesting to test using full CMB and large scale data analysis, however, it is worth mentioning that most of these solutions have been found to suffer from gradient or Higuchi instabilities in for example Könnig (2015) which compromises their viability.

### Constraints on *f*(*R*) models

Planck2015MG also constrained *f*(*R*) models in terms of the scalaron Compton wavelength today $B_0$ [see Eq. (118)]. When using Planck TT $+$ lowP $+$ BSH, they noted a degeneracy between $B_0$ and the optical depth $\tau$. This is removed when adding CMB lensing. They find at 95 % CL: $B_0< 0.12$ for the Planck TT $+$ lowP $+$ CMB Lensing and a very tight bound of $B_0 < 0.79 \times 10^{-4}$ when Planck TT $+$ lowP $+$ CMB Lensing $+$ BAO $+$ WL $+$ RSD is used. The result are thus close to that of a $\varLambda$CDM model and put very stringent limit on a departure toward *f*(*R*) gravity.

Although not among the most recent papers on the subject, Giannantonio et al. (2010) provided one of the most stringent and clear analysis on constraining *f*(*R*) models using WMAP5 CMB data (Nolta et al. 2009), ISW data from cross-correlating WMAP maps with six galaxy data sets in different bands (i.e., 2MASS, SDSS main galaxies, LRGs, and QSOs, NVSS, and HEAO) (Giannantonio et al. 2008). The data covers a redshift ranging from ${\bar{z}}= 0.1$ to ${\bar{z}}= 1.5$ and thus probes variations of the gravitational potentials over a large redshift range. They also added the union compilation of SN data from Kowalski et al. (2008) They used the parameterization $(\mu (a,k), \gamma (a,k))$ as in (90) and (91) with time and scale dependencies given by a refined version of (104) (Zhao et al. 2009). For f(R) models mimicking $\varLambda$CDM expansion, they obtained an upper bound of $B_0<0.4$ at the 95% C.L.

Okada et al. (2013) conducted an analysis using $f \sigma _8$ RSD data for redshift range $z = 0.06$–0.8 from WiggleZ, SDSS LRG, BOSS, and 6dFGRS. They tested the Hu–Sawicki’s *f*(*R*) model finding that only the parameter space that makes the model practically indistinguishable from $\varLambda$CDM is allowed at 95% CL. Dossett et al. (2014) combined large scale data from WiggleZ, BAO (from 6dF, SDSS DR7 and BOSS DR9), and Planck-2013 CMB data (and WMAP Polarization data) to find $\log _{10} < -4.07$ at 95% C.L. thus also putting a tight low bound on the Compton wavelength parameter $B_0$. They also found that *f*(*R*) models cannot explain the tension in the lens amplitude “parameter” of the CMB spectrum. Again, this reduces the allowed *f*(*R*) parameter space to be very close to $\varLambda$CDM. Further recent cosmological constraints on *f*(*R*) models can be found in Nunes et al. (2016), Pérez-Romero and Nesseris (2018) and Li and Shirasaki (2018).

Other very stringent limits on *f*(*R*) and other Chameleon theories came from astrophysical constraints using distance measurements in the nearby universe (Jain et al. 2013b). For example, this screening mechanism affects to different levels the enhanced gravitational force when using Cepheid stars versus when using tip of red giant branch stars to estimated distances. The screening mechanism leads to opposite effects on the inferred distances and offers the possibility to test such theories. The authors found no evidence for an enhancement of the gravitational force and put a constraint of $f_{_{R0}}\le 5\times 10^{-7}$ at 95% C.L. (Jain et al. 2013b). Finally, Sakstein et al. (2014) made the point that while our galaxy and similar ones are screened but less dense galaxies may be subject to less or no screening. In that case, stars in such dwarf galaxies must be hotter, brighter and pulsate with a shorter period. They used a samples of 25 unscreened galaxies and showed that the chameleon mechanism is practically ruled out. We note that using star interior physics has become a promising and effective probe of modification to gravity and associated screening mechanisms and we refer the reader to further works in Sakstein (2013, 2015a, b), Saltas et al. (2018) and references therein.

An excellent review of cosmological and astrophysical constraints on Chameleon fields and in particular *f*(*R*) models can be found in Lombriser (2014). The author provides a thorough compilation of bounds on $|f_{R0}|$ including relevant redshifts and scale as well as the measurements and probes used (see Table I there). Another very useful compilation (compendium) of constraints on Chameleon models including astrophysical and laboratory bounds can be found in Burrage and Sakstein (2016).

Most recently, Battye et al. (2017a) used the designer approach to *f*(*R*) models to compare them to Planck CMB temperature anisotropy, polarisation and lensing data as well as the BAO data from SDSS and WiggleZ. They showed that such approach based on the equation of state to the dark sector perturbations is numerically stable and provides analytical insights of the dynamics of such perturbations in the designer approach to *f*(*R*). They put stringent constraints on $B_0$ finding $B_0 < 0.006$ (95%CL) for the designer models with $w = -1$, $B_0 < 0.0045$ and $|w + 1| < 0.002$ (95%CL) for the designer models with $w \ne -1$. The authors discuss that their results indicate that for these models, *w* is strongly constrained to be $-1$, due to the strong dependence of $\sigma _8$ on *w*. They state that similar results were found in previous works of Raveri et al. (2014) and Hu et al. (2016) for the designer and Hu–Sawicki models using the Effective Field Theory (EFT) approach. They conclude that this hints for the fact that generic *f*(*R*) models with $w \ne -1$ can be ruled out from current cosmological data.

In sum, combining stringent cosmological constraints, astrophysical bounds and no-go theorems on Chameleon mechanism practically rules out *f*(*R*) models and in particular as a possible explanation for cosmic acceleration.

### Constraints on DGP models

Despite being plagued by the presence of ghost fields, the self-accelerating branch of the Dvali–Gabadadze–Porrati (sDGP) has been compared extensively to various astrophysical and cosmological observations. Using various distance measurements, Alcaniz (2002), Jain et al. (2002), Deffayet et al. (2002b) and Fairbairn and Goobar (2006) found that the characteristic scale does verify $r_c \sim H_0^{-1}$. Maartens and Majerotto (2006) used SN-Ia from Riess et al. (2004) and Astier et al. (2006), CMB shift parameter from Wang and Mukherjee (2006) and BAO data from Eisenstein et al. (2005) to constrain the self-accelerating branch finding it consistent with the data at the 2-$\sigma$ level but the $\varLambda$CDM provided a better fit to the data. Some stringent constraints came from Song et al. (2007b) using the angular diameter distance to surface of last scattering from WMAP Y3 (Spergel et al. 2007), SN data from Riess et al. (2004) and Astier et al. (2006) and local measurements of Hubble to show that the flat self-accelerating DGP model is inconsistent with the data at the 3-$\sigma$ level. They also found that the curved self-accelerating models remained consistent with the data but with a poorer fit than the $\varLambda$CDM. However, by using BAO data, growth data from the ISW and ISW-galaxy cross-correlations, they showed that any models with the same self-acceleration history as a wCDM model are strongly disfavored by such data. Fang et al. (2008) and Lombriser et al. (2009) used CMB data, galaxy-ISW cross-correlations data, and distance measurements, to show that both flat and curved self-accelerating DGP models are much disfavored by the data in comparison to $\varLambda$CDM.

While less appealing due to the lack of self-acceleration, the normal branch (nDGP) continues to be used to derive benchmark constraints, to run simulation and build mock data for MG studies in the nonlinear regime; see for example Barreira et al. (2016), Hellwing et al. (2017) and Bose et al. (2018).

### Constraints on Galileon models

Okada et al. (2013) used $f \sigma _8$ RSD data for redshift range $z = 0.06-0.8$ from WiggleZ, SDSS LRG, BOSS, and 6dFGRS. They tested covariant Galileon models with late-time acceleration and found that the model parameter space consistent with the observed background expansion is excluded by RSD data at more than $8-\sigma$ level. The models they considered have too strong of a growth rate and do not fit the data. However, they found that the extended Galileons of De Felice and Tsujikawa (2012) have solutions that are consistent with the RSD data within a 2-$\sigma$ level. As mentioned in Sect. 4.3, we recall that one needs to keep in mind that $f\sigma _8$ data points should be corrected for any assumptions of the $\varLambda \mathrm {CDM}$ model when reducing/calibrating the data. Most recently, Okumura et al. (2016) used their high redshift RSD data point at $z\sim 1.4$ from the FastSound survey using the Subaru telescope as well as lower-z previous measurements in order to constrain deviations from GR. They used covariant Galileon models as well as extended covariant Galileons but considered models with growth less strong than that of models used in Okada et al. (2013). While the models were found to be within the 1-$\sigma$ level at low redshifts, they deviate significantly from the GR-$\varLambda$CDM model at high redshift where they fall outside the 1-$\sigma$ bounds and possibly outside the 2-$\sigma$ for the covariant Galileons, see Fig. 3 from Okada et al. (2013). However, even more stringent constraints have been put now in the way of Galileons models from ISW measurements and from the gravitational-wave event GW170817 and its electromagnetic counterpart as we discuss below and further in Sect. 10.

Barreira et al. (2014a) analyzed cubic Galileon models and found that in the presence of massive neutrinos, the models provide a very good fit to CMB temperature, CMB lensing and BAO data. The authors used Planck-2013 (Ade et al. 2014a) temperature data, WMAP9 polarization data (Hinshaw et al. 2013), and Planck-2013 CMB Lensing (Ade et al. 2014d). They noted these as the PL data set. They added to these data sets, BAO measurements from the 6dF, SDSS DR7 and BOSS DR9 which they noted at the PLB data sets. They dubbed the models as ${\nu } {{\mathrm{Galileon}}}$ and ${\nu } \varLambda {{\mathrm{CDM}}}$ that each having seven cosmological parameters. ${\nu } \varLambda {{\mathrm{CDM}}}$ cosmic acceleration is due to a cosmological constant while that of ${\nu } {{\mathrm{Galileon}}}$ is due to a different coupling between curvature and sources. They found that while in the absence of massive neutrino $\varLambda$CDM is clearly favored by the data, the two models have close $\chi ^2$ when massive neutrinos are added to the analysis. That is $(\chi ^2_{P} ; \chi ^2_{L} ; \chi ^2_{B})=(9813.5\ ; 4.5\ ; 1.0)$ and $(9805.4\ ;8.7\ ; 1.4)$ for ${\nu } {{\mathrm{Galileon}}}$ and ${\nu } \varLambda {{\mathrm{CDM}}}$ respectively and with close total $\chi ^2$’s. They noted that the ${\nu } {{\mathrm{Galileon}}}$ best-fit model is also consistent with the local measurements of the Hubble constant, unlike the $\varLambda$CDM model. However, they noted that the ${\nu } {{\mathrm{Galileon}}}$ shows a negative ISW effect that is hard to reconcile with current observations. The models they considered are plotted against CMB, CMB Lensing and BAO data available at the time of their analysis in Fig. 4 that we reproduce here.

In a most recent analysis, Renk et al. (2017) (including some of the same authors as above) performed a further thorough analysis of self-accelerating Galileon models using CMB data from Planck-2015 temperature and polarization (Ade et al. 2016a) plus CMB lensing (Ade et al. 2016c), BAO (same as above), $H_0$ (Riess et al. 2016) and ISW data. For ISW, they used CMB temperature maps cross-correlated with foreground galaxies from the Wide-field Infrared Survey Explorer (WISE) survey (Wright et al. 2010). They found again that the Cubic Galileon models predict a negative ISW effect and thus is in a 7.8$\sigma$ tension with available data which rules the cubic models out. They also found that the ISW data constrain significantly the parameter spaces for the quartic and quintic Galileon models but leave regions of the parameter space where the models provide fits to the data comparable to the $\varLambda$CDM. However, this time the Galileon models are found in some 2-$\sigma$ tension with the BAO data. They concluded that the models are likely to be decisively constrained by future ISW and BAO data. In sum, while the cubic Galileons have been excluded by the ISW effect here, the quartic and quintic have been excluded by the gravitational-wave event GW170817 and its electromagnetic counterpart GRB170817A, as we discuss in Sect. 10.

Finally, Peirone et al. (2017a) investigated further the effect of neutrino masses and different mass hierarchies on fitting covariant Galileons to current data. They use the Planck 2015 temperature and polarization data, BAO from BOSS, local measurements of $H_0$, weak lensing from KiDS, and supernova JLA compilation. This analyses found that even with neutrinos and considering different mass hierarchies, the data considered rule out all covariant Galileons including the cubic, the quartic, and the quintic, in agreement with other previous results from ISW for the cubic and GW170817/GRB170817A for the quartic and quintic models.

### Constraints on TeVeS

Xu et al. (2015) used the galaxy velocity power spectrum from 6dF survey and the kinetic Sunayev Zel’dovich (kSZ) power spectrum from ACT/SPT (Hasselfield et al. 2013; Schaffer et al. 2011) to put constraints on TeVeS theory (see Sect. 7.3.3). They used these two particular probes in order to provide complementary constraints to those of $E_G$ from Reyes et al. (2010) (see Sect. 6.3) since the latter is insensitive to the amplitude of perturbations. For the TeVeS cosmology they also added one sterile neutrino and 3 massless neutrinos following the suggestion of Angus (2009) in order to fit observations of the CMB temperature power spectrum. They found that the linear kSZ power spectrum is consistent with upper limits of the ACT/SPT data. However, they found that the nonlinear kSZ TeVeS spectrum is ruled out by SPT observations and the ACT data put stringent constraints on the model parameters. They also constrained the models using Ade et al. (2014a) data and allowed for the three parameters $K_B,l_B$ and $\mu _0$ of the TeVeS model to vary as well as the neutrino physical energy density. The best fit cosmological parameter for the TeVeS models were found to be difficult to reconcile with other observations. Namely, the model gives a very small optical depth indicating that re-ionization would have ended at z=1.2 and an inferred value of the Hubble constant $H_0 < 50.8$ km $\mathrm s^{-1}$ Mpc$^{-1}$which is hard to reconcile with any other measurement of this constant. They also performed $\chi ^2$ goodness of the fit test and found that the TeVeS models have an excess of $\delta \chi ^2=501.36$ compared to the $\varLambda$CDM model and concluded that these results from Planck data rule out the TeVeS models.

This is a good example of how cosmological data can be used to rule out models that have evaded so far a number of tests such as solar system constraints and galaxy rotation curves without dark matter. This is a good example to show the promise of cosmological tests in constraining gravity theories and departures from GR at large scales.

However, the TeVeS theory structure remains an example of a complex theory that may have not said its last words as some other developments continue. For example, there is a general version of TeVeS in Skordis (2008) that has not been constrained in Xu et al. (2015). Also, the theory has been combined with a Galilean scalar field (Babichev et al. 2011), although with some continuing but less stringent challenges (Złośnik and Skordis 2017).

### Constraints on non-local gravity models

Full comparison of the ${ RR }$ Non-Local gravity model to CMB and other cosmological data has been performed in Dirian et al. (2014, 2015, 2016a) and Dirian (2017) with further model exploration in Belgacem et al. (2018b). Cosmological background and perturbation equations have been put into the Boltzmann-Einstein code CLASS by Dirian et al. (2016a) allowing for a full comparison to CMB and matter power spectra data.

We report here results from constraining ${ RR }$ non-local model from Dirian et al. (2016a), Dirian (2017) and Belgacem et al. (2018b). The authors used the following data sets. CMB from Planck-2015 (Adam et al. 2016) including: lowTEB data ($\ell \le 29$) and the high-$\ell$ TT,TE,EE ($\ell > 29$) of temperature and polarization spectra (Ade et al. 2016b); temperature plus polarization lensing data in the conservative range $\ell =40-400$ (Aghanim et al. 2016; Ade et al. 2016c). They also used Type Ia SN from the JLA data of SDSS-II/SNLS3 Betoule et al. (2014); and BAO data from Beutler et al. (2011), Ross et al. (2015) and Anderson et al. (2014).

First, Belgacem et al. (2018b) explained that the results found in Dirian et al. (2016a) favoring $\varLambda \mathrm {CDM}$ to ${ RR }$ minimal models is mainly due to fixing the $\sum _{\nu }m_{\nu }=0.06$ eV. When letting this parameter vary, the two models fit the data with practically equal $\chi$-squares and Bayes’ factors (Dirian 2017). Next, they stressed two particular results from their use of CMB $+$ BAO $+$ SN analysis. They find constraints on the Hubble constant of $H_0= 69.49\pm 0.80$ which is higher than the one from using the $\varLambda \mathrm {CDM}$. Compared to the local measurement of $H_0 = 73.24 \pm 1.74$ of Riess et al. (2016), this is only in $2.0\sigma$ tension compared to that which they find for the $\varLambda \mathrm {CDM}$, i.e., $3.1\sigma$. Second they find neutrino masses with the constraints $\sum _{\nu }m_{\nu }=0.219^{+0.083}_{-0.084}\mathrm{eV}$, which they remark falls within the window $0.06~{\mathrm{eV}}\,\lesssim \,\sum _{\nu }m_{\nu }\, \lesssim \, 6.6~\mathrm{eV}$ provided by oscillation and beta-decay experiments and is more consistent than the lower limit in the $\varLambda \mathrm {CDM}$. We refer the reader to Belgacem et al. (2018b) for result summary tables and more discussions.

Next, since the $H_0$ is not in significant tension with the ${ RR }$ minimal model, the authors of Belgacem et al. (2018b) added the local measurements to use CMB $+$ BAO $+$ JLA $+$ $H_0$. They found then $H_0=70.13_{-0.72}^{+0.76}$ and $\sum _{\nu }m_{\nu }=0.168_{-0.084}^{+0.078}\,\,\,\mathrm{eV}$ with a slightly better $\chi ^2$ for the ${ RR }$ model compared to $\varLambda \mathrm {CDM}$, although not statistically significant. However, the authors finish their analysis by considering comparison of the ${ RR }$ model and the $\nu \varLambda \mathrm {CDM}$ models to current data of the growth factor, $f\sigma _8$, data from 6dF GRS (Beutler et al. 2012), SDSS LRG (Oka et al. 2014), BOSS CMASS (Samushia et al. 2014), WiggleZ (Blake et al. 2012), VIPERS (de la Torre et al. 2013) and BOSS DR12 (Alam et al. 2017a). They found that $\chi ^2$ is lower in $\nu \varLambda \mathrm {CDM}$, compared to the minimal ${ RR }$ model with $\delta \chi ^2\simeq 2.01$. They state that when this is combined with the $\delta \chi ^2=-1.0$ from comparison with CMB $+$ BAO $+$ JLA $+\,H_0$ the models are then statistically equivalent. However, they also reported that when $H_0$ is not considered then overall the difference rises to $\delta \chi _{\mathrm{tot}}^2\simeq 4.95$ which favors weakly $\nu \varLambda$CDM over the ${ RR }$ models. This is certainly to be followed closely with incoming growth data.

Finally, the authors concluded their comparison of the ${ RR }$ minimal non-local gravity models by discussing the effect of the recent results from the GW event from the neutron star merger GW170817 and its electromagnetic counterpart GRB170817A. They showed that that gravitational waves in the ${ RR }$ model propagate at the speed of light and thus comply with the limit $c_T\approx 0$. However, they pointed out to the possibility of using standard sirens to distinguish between $\varLambda \mathrm {CDM}$ and the ${ RR }$ model using third-generation GW interferometers which they discussed in a companion paper Belgacem et al. (2017). As they stress there, one can define a “GW luminosity distance” which is different from the standard luminosity distance for electromagnetic signal. They take advantage of the predictivity of their ${ RR }$ model and provide a concrete prediction for the ratio of the GW and EM luminosity distances. They found that the effect due to modified GW propagation is more easily detectable, at future GW interferometers, than the effect from the dark energy equation of state (Belgacem et al. 2017). Furthermore, the authors give a much more detailed discussion of how their model can be tested with modified GW propagation in Belgacem et al. (2018a). The discussion is more general where they propose a parametrization of the effect of modified GW propagation that could be used for any modified gravity theory. They obtain some limits already by comparing the LIGO/Virgo measurement of $H_0$ using standard sirens with that from standard candles, and they compute in detail the sensitivity of the Einstein Telescope to the parameter related to modified GW propagation, in generic modified gravity theories. This will be very relevant to future GW detectors such as LISA and ET. We refer the reader to their papers for more on this new avenue.

## Constraints on deviations from GR and MG models from neutron star merger event GW170817/GRB170817A

The beginning of the 21st century will be remembered for the first detection of gravitational waves (GW) from compact objects. It all started when the Laser Interferometer Gravitational Observatory (LIGO) detected GW signals from the merger of black hole event which confirmed the existence of black holes and the prediction of GW (Abbott et al. 2016a). Almost two years later, LIGO and the VIRGO interferometer made the detection of GW from a merger of two neutron stars (GW170817) (Abbott et al. 2017a). Incidentally, the Fermi Gamma-ray Burst Monitor, and the Anti-Coincidence Shield for the Spectrometer for the International Gamma-Ray Astrophysics Laboratory observed a gamma-ray burst (GRB170817A) event within the following 1.7 seconds and in a close location to GW170817 (Goldstein et al. 2017; Savchenko et al. 2017). There were no doubts that GRB170817A was the electromagnetic counterpart of GW170817 (Abbott et al. 2017b). This was a consequential event to test some aspects of gravity at cosmological scales as one can confront the two completely different types of *astrophysical messengers*. That is exactly what was done immediately after the announcement of the event, see for example Baker et al. (2017), Creminelli and Vernizzi (2017), Ezquiaga and Zumalacárregui (2017), Sakstein and Jain (2017), Langlois et al. (2017b) and Amendola et al. (2017).

Indeed, the scientific community was well-prepared to exploit such an event since a number of papers had already studied the implications that can be drawn from comparing the propagation of gravitational and electromagnetic waves (Amendola et al. 2013b, 2014; Nishizawa and Nakamura 2014; Linder 2014; Raveri et al. 2015; Saltas et al. 2014; Beltrán Jiménez et al. 2016b; Bettoni et al. 2017; Sawicki et al. 2017; Lombriser and Taylor 2016; Lombriser and Lima 2017).

In particular, Lombriser and Taylor (2016) explicitly studied the implications for scalar–tensor gravity from an electromagnetic counterpart measurement to a LIGO/Virgo gravitational wave emitted by a neutron star merger. In their Fig. 4, they predicted a constraint that closely matches that of GW170817/GRB170817A further below. They also discussed the implications of such a measurement for Horndeski scalar–tensor gravity (and beyond), and estimated that such a simultaneous measurement should be anticipated within a few years from writing their paper. Their paper followed a previous analysis by Nishizawa and Nakamura (2014) which also made predictions close to the constraint below from GW170817/GRB170817A.

In GR, GWs travel at the speed of light, however in MG models, this is not always the case. As we discussed in Sect. 7.3.1, it is common to parametrize deviations of the speed of GW, $c_T$, from $c=1$ (keeping our notation convention) by using the tensor speed excess parameter $\alpha _T$ (Bellini and Sawicki 2014)

323 $$\begin{aligned} \alpha _T=c_T^2-1. \end{aligned}$$

Note that the first term on the RHS is actually $(c_{_T}/c)^2$ but we kept the notation convention of setting $c=1$.

Baker et al. (2017) discussed the implication of this event using the $\alpha _x$ (Sect. 7.3.1) parameterization while Creminelli and Vernizzi (2017) used directly the EFT formulation of dark energy and modified gravity theories (see Sect. 5.1). Following Baker et al. (2017), let us note the travel time of GW from GW170817 event to the GW detectors as

324 $$\begin{aligned} t_D-t_e= \frac{d_s}{c_T}, \end{aligned}$$

where $t_D$ is the merger time identified in the GW detectors, $t_e$ is the time of emission of GW and light from the event, and $d_s\simeq 40$ Mpc is the distance to the source event. It is worth mentioning that an Euclidean treatment of the distances is used here because of the relatively short distances involved. Similarly, we note the travel time of light from GRB170817A to the GRB light detectors as

325 $$\begin{aligned} t_L-t_e= d_{s}, \end{aligned}$$

where $t_L$ is the time of arrival (or peak brightness) measured at the GRB detectors. Taking the difference of the two above equations gives

326 $$\begin{aligned} t_G-t_L=d_s\left( \frac{1}{c_T}-1\right) . \end{aligned}$$

Using the arrival time difference of $t_D-t_L \simeq 1.7$ seconds and the value of $d_s$ into the above equation and translating the results to (323) gives the stringent bound

327 $$\begin{aligned} \left| \alpha _T\right| \lesssim 1\times 10^{-15}. \end{aligned}$$

It is worth mentioning here that one assumed here that the gamma-ray photons and GWs are released simultaneously. In reality, there could be a delay of order a few hours between these two events. Therefore, taking into account such a possible delay weakens the bound by a few orders of magnitude. However, $10^{-15}$ or $10^{-12}$ are both very tight constraints, leading to practically the same outcome.

Incidentally, the bound in (327) is consistent with the bound derived in Moore and Nelson (2001) and Kimura and Yamamoto (2012) from gravitational Cherenkov radiation which constrains GW speed to not exceed the speed of light, assuming a galactic origin for the high energy cosmic rays.

The bound (327) suggests that $\alpha _T\simeq 0$ so a number of papers studied the same consequences of assuming this is the case in order to constrain deviations from GR and MG models or, in other cases, propagating the stringent bound to constrain departures from GR.

### Implications for scalar–ensor theories

#### Implications for Horndeski models

The Horndeski class of MG models is a large class of scalar–tensor theories that was discussed in Sect. 7.3.1 and for which the gravitational action was given by (146). Baker et al. (2017) discussed that the constraint (327) can be realized by a highly tuned cancellation between the Horndenski action terms $G_{4,X}$, $G_{5,\phi }$ and $G_{5,X}$ that can all contribute to $\alpha _T$. However, as they stated, a more logical implication of $\alpha _T\simeq 0$ is that each of the three terms vanishes identically. Furthermore using the Bianchi identity, the Horndeski action then reduces, besides the potential term and the cubic term, to (Baker et al. 2017)

328 $$\begin{aligned} {\mathcal {L}}_4=f(\phi )R \end{aligned}$$

leaving only conformally coupled theories of the Jordan–Brans–Dicke (JBD) type. This then eliminates the quartic and quintic Galileons theories. These consequences on the Hordeski terms were also given prior to this event in McManus et al. (2016). See also Creminelli and Vernizzi (2017) for the same conclusions from GW170817/GRB170817A. Sakstein and Jain (2017) combined the constraint (327) with the lack of violation of the strong equivalence principle in the supermassive black hole in M87 in order to exclude the quartic Galileon model.

The JDB like models (328) can be divided in two sub-classes as discussed in Baker et al. (2017). The first sub-class is the generalized JDB where the scalar field does not evolve significantly on cosmic timescales. However, this sub-class of models requires the chameleon screening mechanism to pass solar-system tests of gravity and thus cannot be self-accelerating due to the no-go theorems discussed in Sect. 8.1 (Wang et al. 2012). In the second sub-class, the scalar field evolves significantly on cosmic timescales, for example as caused by terms in $G_2$ and $G_3$, producing self-acceleration. Thus the event does not exclude cubic Galileons, kinetic gravity braiding models (Deffayet et al. 2010) and k-essence models (Armendáriz-Picón et al. 2000, 2001). See more discussion in Baker et al. (2017).

Ezquiaga and Zumalacárregui (2017) considered the implications of the GW170817 and GRB170817A on MG models by starting from the covariant Galileon models and then moving to their generalizations to Horndeski and beyond Horndeski models. They translated the stringent bound (327) into bounds on the Galileon model coefficients and their generalizations. They arrived at similar conclusions as in Creminelli and Vernizzi (2017), Baker et al. (2017) and Langlois et al. (2017b). They tabulated models explicitly indicating that, in the Horndeski general class, Brans–Dicke, f(R), kinetic gravity braiding (Deffayet et al. 2010) are not affected, while quartic and quintic Galileons (Nicolis et al. 2009; Deffayet et al. 2009b), Fab Four (Charmousis et al. 2012), de Sitter Horndeski (Martin-Moruno et al. 2015), and $f(\phi )$Gauss–Bonnet (Nojiri et al. 2005) are all excluded.

#### Implications for Beyond Horndeski models

The beyond-Horndeski models of Gleyzes et al. (2015a, b) receive almost the same consequences as Horndeski models except for a specific combination of terms in the beyond-Horndeski action which can realize $\alpha _T=0$. The cosmology and motivation for such a specific combination remains to be explored and it is not clear if such models have any particular motivation (Baker et al. 2017). Sakstein and Jain (2017) also excluded the quartic beyond horndeski models. Ezquiaga and Zumalacárregui (2017) with their approach above found that beyond-Horndeski models with disformal tuning and the $A_1=0$ class of quadratic Degenerate Higher-Order Scalar–Tensor (DHOST) theories (Langlois and Noui 2016a) are not excluded, while quartic/quintic beyond-Horndeski models (Langlois and Noui 2016a), quadratic (with $A_1\ne 0$) (Langlois and Noui 2016a) and cubic DHOST models (Ben Achour et al. 2016a) are all excluded. They showed that only three alternatives (or their combination) are possible for scalar–tensor theories: (1) restricting Horndeski models to their minimum simplest terms that keep $c_T=1$; (2) applying a conformal transformation to these minimal Horndeski models which preserves the causal structure; (3) using Horndeski models but compensating the terms that modify the speed of GW to keep it luminal. This is done by a specific disformal factor to tune away the departure of the speed of GW from light speed. Langlois et al. (2017b) presented an analysis of the implications of the event GW170817/GRB170817A using the DHOST framework. For Horndeski and Beyond Horndeski theories, they came to the same conclusions discussed above from Creminelli and Vernizzi (2017), Baker et al. (2017) and Ezquiaga and Zumalacárregui (2017).

### Implications for vector–tensor theories

The constraints $\alpha _T=0$ imposes on Generalized Einstein-Aether theories (Jacobson and Mattingly 2001; Zlosnik et al. 2007) the condition $c_1=-c_3$ (see Sect. 7.3.2) which makes the effective Planck mass reduce to the GR value, while the cosmological background evolution remains different from GR (Baker et al. 2017). For the Generalized Proca theories (Tasinato 2014; Heisenberg 2014), the condition $\alpha _T=0$ imposes either a fine tuned cancellation of terms in the action or imposes that the terms related to $\alpha _T$ be all identically zero. The latter natural interpretation gives a branch with a cosmological evolution different from GR with a rescaled Planck mass in the modified Friedmann equation (Baker et al. 2017). Similar results were obtained in Oost et al. (2018b) about the Einstein-Aether theories. Since the Generalised Proca theory has a similar structure to Horndeski, the effects of $\alpha _T = 0$ on Generalised Proca is similar to the effects on Horndeski, i.e., the quartic and quintic terms are (effectively) ruled out. The same implication applies to the beyond Generalized Proca models of Heisenberg et al. (2016).

### Implications for massive gravity and bigravity theories

For massive gravity and bimetric gravity (de Rham et al. 2011; de Rham and Gabadadze 2010; Hassan and Rosen 2012a), the new results from GW170817 and GRB170817A have no significant cosmological consequences. For massive gravity, one can just obtain further weak constraints on the mass of the graviton. Similar to bounds obtained from previous Black Hole merger events (Abbott et al. 2016b), Baker et al. (2017) used the time delay of the GRB170817A electromagnetic counterpart to find $m \lesssim 10^{-22}$eV for the graviton mass. This is again much weaker than the solar system bound of the order of $m \lesssim 10^{-33}$ eV (e.g. de Rham et al. 2017) or galaxy cluster bound of order $m \lesssim 10-29$ eV (e.g. Desai 2018). This is an independent bound though. More relevant to our review, the local bounds obtained from GW propagation and the electromagnetic counterpart have no consequence on the cosmology of massive gravity and bigravity theory (Lagos and Ferreira 2014; Cusin et al. 2015a; De Felice et al. 2014; Narikawa et al. 2015; Max et al. 2017). However, see Brax et al. (2017) and Akrami et al. (2018) for constraints on doubly coupled metrics to matter models.

### Implications for ghost condensates and Ho**ř**ava–Lifshitz Gravity

For ghost condensates (Hamed et al. 2004), the modification of the GW speed is given by $c_T^2-1 \sim M^2_{GC}/M^2_{Pl}$, where $M_{GC}$ is the typical scale of the model. Now, experimental bounds on modifications of Newton law give $M_{GC}\le 10$ MeV. So it is not expected to see any significant changes in the speed of GW and the constraint (327) does not affect this theory (Creminelli and Vernizzi 2017). This is not the case for Hořava–Lifshitz theory as stated in Creminelli and Vernizzi (2017) where $C_T$ is expected to deviate from the speed of light.

Gümrükçüoǧlu et al. (2018) argued that the implications of the bound (327) are more subtle for HL parameters. As they explain, the theory has 3 independent IR parameters ($\alpha$, $\beta$ and $\gamma$ in their paper). Before the constraint on the speed of GW, the tightest constraints on HL in IR had come from ppN constraints where one assumes $\alpha = 2 \beta$. Papers then normally considered the 2-dimensional sub-region determined by ($\alpha =2\beta$ versus $\gamma$) in the parameter space. But the recent result from GW170817/GRB170817B set a constraints of $|\beta | < 10^{-15})$ so it is not justified to set $\alpha =2 \beta$ since it would require $\alpha$ and $\beta$ to be highly fine-tuned to the $10^-{15}$ level. Accordingly, they motivated in their paper to look at the ($\alpha$, $\gamma$) parameter sub-space, as the current constraints on $\alpha$ and $\gamma$ are orders of magnitudes looser compared to $\beta$ and focus on the $\beta =0$ plane. In the limit where HL and GR becomes indistinguishable (from IR perspective), HL becomes strongly coupled and loses its use as a perturbative alternative theory for GR. As they discussed in the paper, this puts a lower bound on $\alpha$ and $\gamma$ parameters set by experiments testing the validity of perturbative GR. This means that future tighter constraint on HL in IR regime combined with upper energy bounds on the validity of perturbative GR from future experiments could rule out HL as perturbatively renormalizable theory of gravity and make HL absolute. See Gümrükçüoǧlu et al. (2018) for a more detailed discussion.

### Implications for higher dimension models

As we discussed further above, in these models our universe is a 3$+$1 brane embedded in a higher dimensional space, for example 4$+$1 dimensional anti-de-Sitter space (see Sect. 7.5). In such a universe, gravity is the only force that propagates in the extra dimension (or the bulk space), while other forces are constrained to the brane hypersurface. As a consequence, GW and EM signals follow different paths leading to a time lag between the two signals propagating from a given point to another. GW170817 and GRB170817A can thus be used to put constraints on such models.

Visinelli et al. (2018) considered the setting where the GW and EM signals travel at the same speed but where the GW can take a shortcut in the bulk space and thus arrive ahead of the EM signal. This can be used to put a constraint on the radius of curvature, $\ell$, of the AdS5 bulk space. They used a $\varLambda \mathrm {CDM}$ model and performed a likelihood analysis to set an upper limit of $\ell \lesssim 0.535$ Mpc (68% CL). As the authors mention, this bound is not competitive with current Solar System constraints (e.g., Long et al. 2003; Tan et al. 2016), but is the first constraints from multi-messenger measurements.

Pardo et al. (2018) used the GW170817/GRB170817B result in a different way to put a constraint on the possible number of spacetime dimensions. They used the fact that in these higher dimension models, there is gravitational leakage into extra dimension leading to dumping of the amplitude of GW that reflects on the inferred distance to gravitational source. They used GW as standard sirens and extracted directly the luminosity distance, $d^{GW}_L$, to GW170817. They compared this distance with the inferred luminosity distance to the EM counterpart, $d^{EM}_L$. The latter is determined using the Hubble law at the small redshift from the source, i.e., $v_H=c z= H_0 d^{EM}_L$ (but taking into account the peculiar velocity of the host galaxy with respect of its galaxy-group precessing velocity). Following Deffayet and Menou (2007), they used a dumping parameter $\gamma$ to write $d^{GW}_L = (d^{EM}_L)^{\gamma }$. This parameter is related to the number of dimension, *D*, by $\gamma =\frac{D-2}{2}$. From the two distances as inferred above, they find $\gamma = 1.01^{+0.04}_{-0.05}$ at the 68% CL (using the local value of $H_0$) or $\gamma = 0.99^{+0.03}_{-0.05}$ (using Planck value of $H_0$). This in turn allowed them to put constraints on the spacetime dimension number as $D = 4.02^{+0.07}_{-0.10}$ (using local $H_0$) and $D = 3.98^{+0.07}_{-0.09}$ (using Planck $H_0$). They concluded that their results are in favor of the 3+1 dimensions of GR.

### Implications for results on MG parameters and large-scale-structure from GW170817 and GRB170817A

Interestingly, Saltas et al. (2014) and Sawicki et al. (2017) showed that there is a one-to-one relationship between modification to the propagation of GW and the gravitational slip parameter when the source is a perfect fluid matter. Amendola et al. (2017) noted that this result in combination with the constraint $c_T=c$ from GW170817/GRB170817A implies that the presence of a slip MG parameter in scalar–tensor theories can be attributed to only a conformal coupling to gravity. They also showed that the surviving vector–tensor theories cannot have any slip at all so detecting any slip parameter will rule all of them out. They demonstrated then that the growth rate in the surviving models must be at least as fast as that of GR except possibly for beyond Horndeski theories. Finally, they showed that if the slip parameter is to have any scale dependence at all then it should be in a way that the parameter reduces to the GR unity value at large scales with no-slip and so the model cannot be distinguished from GR at large scales.

In light of the implication of GW170817/GRB170817A (i.e., $\alpha _T=0$) and its consequences for the slip parameter (Saltas et al. 2014; Sawicki et al. 2017), the study Linder (2018) considered scalar–tensor models where the slip parameter is identically zero. Linder (2018) noted that with the vanishing of $\alpha _T$, the no slip criterion is simply given by $\alpha _B=-2 \alpha _M$. It was then shown that stability conditions for absence of ghosts and a positive sound speed squared for perturbations impose further restrictions on $(\alpha _B,\alpha _K)$ reducing the independent parameters to only one. So this no slip gravity can be characterized by one MG parameter. Using some guidance from cosmological evolution and stability requirements, some forms on the time evolution of the one parameter (e.g. $\alpha _M$) was proposed and studied. Linder (2018) then compared the growth rate data of $f\sigma _8$ to some of these models and found them to fit the data better than $\varLambda$CDM as they have a lower growth. It was noted that, unlike many other scalar–tensor theories, no-slip gravity predicts a weaker gravity than GR which explains the growth fit and, as the author states, could potentially inform the tension in the low amplitude found in weak lensing studies. The study concludes with forecasts of constraints from the DESI galaxy redshift survey showing that it could be distinguished from GR at the 3-$\sigma$ level.

Peirone et al. (2017b) Performed an extensive analytical and numerical analysis of the MG parameters $\varSigma$ and $\mu$ or equivalently $G_\mathrm{light}/G$ and $G_\mathrm{matter}/G$. They considered Horndeski models that are consistent with tests of gravity and the cosmic expansion history with late time acceleration. They also take into account the recent result from GW170817 and its counterpart GRB170817A, setting $c_T=c$. They confirmed a conjecture they made in their earlier work (Pogosian and Silvestri 2016) about MG parameters in Horndeski models. That is $(\sigma -1)(\mu -1)\ge 0$ must hold in viable Horndeski models. They also test the validity of the quasi-static approximation in Horndeski models finding that it holds well at small and intermediate scales but fails at $k\le 0.001$ h/Mpc. They concluded in their analysis that despite the stringent result from GW170817/GRB170817A, there remain Horndeski models with non-trivial modifications to gravity at the level of linear perturbations and large scale structure. They stressed the complementarity of different approaches to modifications to GR and the practicality of using the phenomenological $\varSigma$ and $\mu$ parameterization and their consistency relations, see also Pogosian and Silvestri (2016).

Finally, Battye et al. (2018) explored the results from GW170817/GRB170817A using an equation of state approach to modified gravity models. They confirmed the strong constraints found for Hordeski and Einstein-Aether models. They discuss how it is possible to construct MG models that evade GW170817/GRB170817A constraint but still provide cosmologically interesting modifications to gravity. These include *f*(*R*), non-local, and higher order derivative models.

### Implications for Vainshtein screening mechanism after GW170810 and GRB170817A

Crisostomi and Koyama (2017b) applied the implication of GW170817 and its counterpart GRB170817A to study the Vainshtein screening mechanism in the very general class of Degenerate Higher-Order scalar–tensor (DHOST) theories (including Horndeski and beyond-Horndeski models). They set $c_T=c$ and find that the Vainshtein mechanism generally works outside a matter source but it fails inside the matter. This then opens the door to test these theories using astrophysical observations inside matter sources such as stars, galaxies and clusters of galaxies and large scale structure. The formalism for such structures in this context depends on 3 parameters and some astrophysical constraints have already been derived on them (Koyama and Sakstein 2015; Saito et al. 2015; Sakstein 2015a, b; Sakstein et al. 2016, 2017a). Dima and Vernizzi (2017) found further implications and results on the Vainshtein screening mechanism from GW170817 and its counterpart that are consistent with the results above about the breaking of the Vainshtein screening inside astrophysical bodies. Finally, Langlois et al. (2017b) study the Vainshtein mechanism in the Degenerate Higher-Order Scalar–Tensor (DHOST) framework. They derive, for the DHOST theories satisfying $c_T=c$, the gravitational equations for inside and around a non-relativistic spherical object. Unlike outside the object, they found that gravity inside the object deviates from standard gravity. They also found that the deviation from standard gravity inside the object can be described by 3 parameters that satisfy consistency relations and can be constrained using present and future astrophysical data (Langlois et al. 2017b). This concurs with the findings above. It is also worth noting that the breaking of the Vainshtein screening mechanism inside matter has been discussed prior to GW170817 and GRB 170817B; see, for example Beltrán Jiménez et al. (2016b).

### Further notes or caveats on the implications of GW170817 and GRB170817A

Some caveats were raised in Baker et al. (2017) about the fact that the result $\alpha _T\simeq 0$ is based on a measurement at very low redshift ($z_s=0.01$) corresponding to practically the present time in cosmic history so it is possible, in principle, that this was not always the case. Another possible caveat is the limitation that can come from noting that cosmological gravitational waves have long wavelengths and propagate in a higher cosmological average density, while GW170817 has short wavelength and propagated to us in almost empty space. So it will be interesting to see/confirm if gravitational waves at cosmological scales would travel at the speed of light. See further discussions in Baker et al. (2017).

**Table 5:** Current status of some selected MG models

	Has self-acceleration	Passes ghost and other instability constraints	Screening mechanism type	Passes current cosmological, astrophysical, and GW170817	Otherfeatures, limitations, or constraints
*f*(*R*)	$\checkmark$	$\checkmark$	Chameleon	$\times$ (self-acc)	No-go theorem for self-acc.; some models inst. inside NS
				$\checkmark$ (non self-acc)	
sDGP	$\checkmark$	$\times$	Vainshtein	$\times$	
nDGP	$\times$	$\checkmark$	Vainshtein	$\checkmark$?	
Cubic (Galileons, Horndeski, B-H)	$\checkmark$	$\checkmark$	Vainshtein	$\times$	
Quartic (Galileons, Horndeski, B-H)	$\checkmark$	$\checkmark$	Vainshtein	$\times$	
Quintic (Galileons, Horndeski, B-H)	$\checkmark$	$\checkmark$	Vainshtein	$\times$	
Brans–Dicke (BD), Generalized BD	$\checkmark$	$\checkmark$	?	$\checkmark$?	BD very tightly constrained by solar syst.
				$$\checkmark $$	
Einstein-Aether, Generalized EA	$\times$?	$\checkmark$	C?, V	$\checkmark$?	Lorentz-violation
	$$\checkmark $$				
Proca and gene-ralized Proca	$\checkmark$	$\checkmark$	Vainshtein	Full CMB and LSS analysis not completed	
TeVeS	$\times$	$\checkmark$	Reduces to MOND at small scales	$\times$ but debated for some models	
Massive gravity	$\checkmark$	$\checkmark$?	Vainshtein	$\checkmark$?	No-FLRW sols. Higuchi inst.
Bimetric massive gravity	$\checkmark$	$\times$?	Vainshtein	$\checkmark$	Higuchi or gradient inst.
Horava–Lifshitz	$\times$	$\checkmark$	Close to GR at small scales	$\checkmark$ but narrow param. space	Lorentz-violation
Non-local *RR*	$\checkmark$	$\checkmark$	Reduces to GR	$\checkmark$	
Beyond-Horndeski, DHOST	$\checkmark$	$\checkmark$	C, V, W	Full CMB and LSS analysis not completed	Some models have gradient inst.?

Self-acceleration means that the models can have acceleration without a cosmological constant or other equivalent constant coming from the theory. BH stands for Beyond Horndeski models. For screening, C stands for Chameleon, V for Vainshtein, and W for weak coupling. NS stands for Neutron Stars. The reasoning here is that if a theory has specific models or branches that pass the constraint then we put a check. A question mark means that the point is still under debate or is unclear in the current literature

## Computer codes and packages for testing gravity at cosmological scales

A number of codes and software packages have been developed following the rapid development of the subject of testing GR and MG models at cosmological scales. Similar to the theoretical developments, codes have been developed according to two types. The first type is where a generic parametrization of deviations from GR is implemented using one of the generic parametrizations of Sect. 5.2. The second type is where the codes have focused on implementing a specific MG model or a broad class of models such as those described in Sect. 7.

It is worth noting that most codes that solve Einstein–Boltzmann equations are based on a modification of two popular codes that solve the Boltzmann and gravitational field equations to calculate CMB temperature and polarization power spectra as well as the matter power spectrum. The first is CAMB (Code for Anisotropies in the Microwave Background) and is available at http://camb.info/, see also Lewis et al. (2000). The second code is CLASS (Cosmic Linear Anisotropy Solving System) and is available at http://class-code.net/, see also Lesgourgues (2011) and Blas et al. (2011a). There are however other codes that are not based on these two systems such as for example DASh (Kaplinghat et al. 2002) and COOP (Huang 2012a) (available at http://cita.utoronto.ca/~zqhuang/coop/).

We describe further below two examples of codes of the first type for generic deviation from GR, i.e., ISitGR (Dossett et al. 2011b) and MGCAMB (Zhao et al. 2009; Hojjati et al. 2011). We also describe two examples of codes of the second type, i.e., hi_class (Zumalacárregui et al. 2017) and EFTCAMB (Hu et al. 2014a, b; Raveri et al. 2014) that both deal with broad classes of scalar–tensor MG models, and we refer the reader to the comparative study of Bellini et al. (2018) for a detailed list and description of other codes.

Codes of the second type include: Cosmology Object Oriented Package (COOP) (Huang 2012a, b) which implements an EFT approach to dark energy and modified gravity theories including the Horndeski broad class of scalar–tensor theories; Davis Anisotropy Shortcut Code (DASh) (Kaplinghat et al. 2002); CLASSig (Umiltà et al. 2015); a code used in Avilez and Skordis (2014) for Jordan–Bran–Dicke gravity; a modified version of CMBEASY (Doran 2005) for Einstein-Aether gravity (Zuntz et al. 2008); modified versions of CAMB (Lewis et al. 2000) for *f*(*R*) models (Dossett et al. 2014; Bean et al. 2007; Battye et al. 2016, 2017a; Battye and Pearson 2013); a modified version of CAMB (Lewis et al. 2000) for covariant Galileons (Barreira et al. 2012); CLASS-LVDM for Hořava–Lifshitz gravity (Ivanov 2017); and modified versions of CAMB and CLASS for models of nonlinear gravity with respective references (Barreira et al. 2014c) and (Dirian et al. 2016b).

We reproduce Table I from Bellini et al. (2018) (as Table 6 here) that provides a good list of such codes with tested models, to which we added the corresponding references.

Finally, we do not cover here N-body simulation codes for MG models or implementation of semi-analytical models but we refer the reader to Winther et al. (2015) (and references therein) for a recent comparative analysis of MG N-body codes. See also other recent works using the Comoving Lagrangian Acceleration (COLA) approach in Valogiannis and Bean (2017) and Winther et al. (2017). The presence of screening mechanisms in MG models makes the implementation of MG simulations more complicated. A parameterization for modified gravity on nonlinear cosmological scales was proposed in Lombriser (2016) and a fitting formula for *f*(*R*) Hu–Sawicki model has been derived in Zhao (2014).

**Table 6:** The codes used in the comparison by Bellini et al. (2018) along with the models tested

	$\alpha$ Param-	EFT Para-	JBD	Covariant	f(R)	Hořava	Non-Local
	etrization	metrization		Galileon	designer	Lifshitz	Gravity
EFTCAMB (Hu et al. 2014b)	$\checkmark$	$\checkmark$	$\checkmark$	$\checkmark$	$\checkmark$	$\checkmark$	$\times$
hi_class (Zumalacárregui et al. 2017)	$\checkmark$	$\checkmark$	$\checkmark$	$\checkmark$	$\times$	$\times$	$\times$
COOP (Huang 2012a)	$\checkmark$	$\times$	$\times$	$\times$	$\times$	$\times$	$\times$
GalCAMB (Barreira et al. 2012)	$\times$	$\times$	$\times$	$\checkmark$	$\times$	$\times$	$\times$
BD-CAMB (Avilez and Skordis 2014)	$\times$	$\times$	$\checkmark$	$\times$	$\times$	$\times$	$\times$
DashBD (Kaplinghat et al. 2002)	$\times$	$\times$	$\checkmark$	$\times$	$\times$	$\times$	$\times$
CLASSig (Umiltà et al. 2015)	$\times$	$\times$	$\checkmark$	$\times$	$\times$	$\times$	$\times$
CLASS_EOS_fR (Battye et al. 2017a)	$\times$	$\times$	$\times$	$\times$	$\checkmark$	$\times$	$\times$
CLASS-LVDM (Ivanov 2017)	$\times$	$\times$	$\times$	$\times$	$\times$	$\checkmark$	$\times$
NL-CLASS (Dirian et al. 2016b)	$\times$	$\times$	$\times$	$\times$	$\times$	$\times$	$\checkmark$
NL-CAMB (Barreira et al. 2014c)	$\times$	$\times$	$\times$	$\times$	$\times$	$\times$	$\checkmark$

As they note, the table shows only the models used in their paper, not all the models that each code can test. We added here to each line the references to corresponding papers. Reproduced with permission from Table I of Bellini et al. (2018), copyright by APS

### Integrated Software in Testing General Relativity (ISiTGR)

We start with ISiTGR (pronounced *Is it GR?*) that is publically available at http://www.utdallas.edu/~jdossett/isitgr/) and described in Dossett et al. (2011b). ISiTGR is an integrated set of modified modules for the publicly available packages CosmoMC (Cosmological Monte Carlo) (Lewis and Bridle 2002) and CAMB (Lewis et al. 2000). CosmoMC software uses a Markov-Chain Monte-Carlo (MCMC) approach to explore cosmological parameter spaces (see more information at http://cosmologist.info/cosmomc/).

ISiTGR introduces all the MG modifications to those two packages and combines them to a modified version of the Integrated Sachs–Wolfe (ISW)-galaxy cross correlations module of Ho et al. (2008) and Hirata et al. (2008) to test GR. It also includes a modified weak-lensing likelihood module for the refined Hubble Space Telescope (HST) Cosmic Evolution Survey (COSMOS) lensing tomography analysis as described in Schrabback et al. (2010) which has also been modified to test GR. It also includes a new baryon acoustic oscillation (BAO) likelihood module for the WiggleZ Dark Energy Survey BAO measurement data (Blake et al. 2011c). ISiTGR also has a version tailored specially to constrain *f*(*R*) models and is for example described and used in Dossett et al. (2014) and available at the same website above.

ISiTGR uses the modified growth parameters as described in Eqs. (86), (87), (88), and (89) as well as their time and scale evolution given by Eqs. (97), (98), (99), (100), (101), and (102), see also Table 2.

For ISiTGR and other codes discussed further below, it is worth noting that CAMB is written in the synchronous gauge and uses the metric potentials *h* and $\eta$ as described in Ma and Bertschinger (1995) instead of the potentials $\varPhi$ and $\varPsi$ of the conformal Newtonian gauge used in Sect. 5.2. In order to give a brief description of the implementation of ISiTGR (and other software further below), we will outline some common conversion and implementation steps using CAMB. The metric potentials in the two gauges are related to one another by, e.g., Ma and Bertschinger (1995)

329 $$\begin{aligned} {\varPhi }= & {} \eta -\mathcal {H}\alpha , \end{aligned}$$

330 $$\begin{aligned} \varPsi= & {} \dot{\alpha }+\mathcal {H} \alpha , \end{aligned}$$

where

331 $$\begin{aligned} k^2\alpha = \frac{\dot{h}}{2} +3\dot{\eta }. \end{aligned}$$

Now, CAMB evolves the metric potential $\eta$ (or $k\eta$) as well as the matter perturbations, $\delta _i$, heat flux, $q_i$, and the shear stress $\sigma _i$ for each matter species in the synchronous gauge according to the evolution equations given in Ma and Bertschinger (1995). Furthermore, CAMB uses two other variables noted $\sigma _{\mathrm{CAMB}}$ and $\mathcal {Z}$ that are defined and evaluated at each time step as follows

332 $$\begin{aligned} \sigma _{{\mathrm{CAMB}}} \equiv k\alpha = \frac{k(\eta -\varPhi )}{\mathcal {H}}, \end{aligned}$$

333 $$\begin{aligned} \mathcal {Z} \equiv \frac{\dot{h}}{2k} = \sigma _{{\mathrm{CAMB}}}- 3\frac{\dot{\eta }}{k}. \end{aligned}$$

The idea is that these variables allow CAMB to be written in such a way that the evolution of all other variables is changed simply by adjusting the evolution of the metric potential $\eta$. Thus it is important that one derives an equation for the evolution of $\eta$ consistent with the modified growth equations (86) and (88). As described in Dossett et al. (2011b), after some steps, one obtains

334 $$\begin{aligned} \dot{\eta }=\frac{-1}{2f_Q}\left\{ 2(\mathcal {H}^2-\dot{\mathcal {H}})k^2\alpha +\sum _i\bar{\rho _i}(a)\left[ \left( 2\mathcal {H}\left[ \mathcal {D}-Q\right] +\dot{Q}\right) \delta _i -Q(1+w_i)k^2\alpha - Q f_1\frac{q_i}{k} \right] \right\} , \nonumber \\ \end{aligned}$$

with

335 $$\begin{aligned} f_Q = k^2 + \frac{3}{2} Q\sum _i\bar{\rho _i}(1+w_i). \end{aligned}$$

Finally, the next necessary change is to redefine the derivatives of the Newtonian metric potentials, $\dot{\varPhi }+\dot{\varPsi }$, which go into evaluating the ISW effect in the CMB temperature anisotropy spectrum. This can be done quickly by observing that the quantities $\delta _i$ and $\sigma _i$ are invariant in transformations between the synchronous and conformal Newtonian gauges. Thus one can simply take the time derivative of (88) and sub in for $\dot{\delta }$ and $\dot{{\bar{\rho }}}_i$ to get:

336 $$\begin{aligned} \dot{\varPhi }+\dot{\varPsi }= & {} \frac{1}{k^2}\sum _i\bar{\rho _i}(a)\Bigg \{\left[ ((1+3w_i)Q+2\mathcal {D})\mathcal {H}-\dot{Q}\right] \frac{3(1+w_i)\sigma _i}{2} -\frac{3Q(1+w_i)\dot{\sigma }_i}{2} \nonumber \\&+(\mathcal {D}\mathcal {H}-\dot{\mathcal {D}})\delta _i+\mathcal {D}(1+w_i)\left( k^2\alpha -3\dot{\eta }\right) + \mathcal {D} f_1\frac{q_i}{k} \Bigg \}. \end{aligned}$$

Beside these changes other small adaptations, modifications and additions to both CAMB and CosmoMC are necessary to assure a smooth running and accurate output of modified CMB spectra according to the MG parameters. These can be found in Dossett et al. (2011b).

ISiTGR has been used or cited in over 50 papers. ISiTGR was used in the recent KiDS survey MG analyses (Joudaki et al. 2017) and KiDS+2dFLenS (Joudaki et al. 2018) as well as CFHTLenS+Planck data analysis including intrinsic alignment of galaxies as a systematic effect in Dossett et al. (2015).

### Modification of growth with CAMB (MGCAMB) and MGCosmoMC

MGCAMB provides a set of patches to the code CAMB in which the linearized Einstein equations were modified according to MG Eqs. (90) and (91). The software is publically available at http://aliojjati.github.io/MGCAMB/mgcamb.html and described in Hojjati et al. (2011) and Zhao et al. (2009). As described on its website, there was a major upgrade to MGCAMB in Hojjati et al. (2011) from the original version of Zhao et al. (2009), making it easier to use with CosmoMC and working for the entire redshift range. Similarly, Modified Gravity models with CosmoMC (MGCosmoMC) is a modified version of CosmoMC that allows one to fit modified gravity parameters to data sets in addition to other cosmological parameters.

The most recent versions of MGCAMB and MGCosmoMC include a wide range of parametrizations to accommodate MG models such as screened scalar–tensor theories as described in Brax et al. (2012), Symmetron parameterization, generalized Dilaton parametrization, Hu–Sawicki *f*(*R*) gravity, as well as the time and scale evolution parametrizations of MG parameters (90) and (91). MGCAMB has been used or cited in over 100 papers and has been used, in for example, Ade et al. (2016b).

### Horndeski in CLASS (hi_class)

hi_class (Zumalacárregui et al. 2017) is an extension to the Boltzmann solver code CLASS (Lesgourgues 2011; Blas et al. 2011a) to include modification to GR based on Horndeski models. hi_class inherits all the functionality of CLASS and can calculate cosmological distances, CMB, matter, and galaxy number count power spectra for this class of models. A publicly available version noted as hi_class teaser can be cloned or downloaded from the repository https://github.com/miguelzuma/hi_class_public or from the webpage http://miguelzuma.github.io/hi_class.html. This version is described in Zumalacárregui et al. (2017) and the latter websites.

The implementation of Horndeski in hi_class code is based on the EFT parameterization (see Sect. 5.1). CLASS and the hi_class extension are written in C programming language but use a class-structure and modularity similar to that of object-oriented languages such as C++ or Java in order to make the code more readable while easier to parallelize (see http://class-code.net/ for a discussion).

Since it encompasses a large class of models, hi_class has been used in a number of recent analyses including Bellini and Zumalacárregui (2015), Bellini et al. (2016, 2018), Renk et al. (2016, 2017), Alonso et al. (2017), Lorenz et al. (2017) and Ezquiaga and Zumalacárregui (2017).

### Effective field theory with CAMB (EFTCAMB) and (EFTCosmoMC)

EFTCAMB (Hu et al. 2014a, b) is a set of patches to the code CAMB which implements the EFT approach to dark energy and modified gravity models of cosmic acceleration as described in Sect. 5.1. The package comes along with a modified version of CosmoMC, called EFTCosmoMC, that allows one to use the software with cosmological data sets. The code description and download are available at http://eftcamb.org/index.html and the corresponding papers (Hu et al. 2014a, b). A useful flowchart of the code and models covered is also accessible at http://eftcamb.org/images/EFTCAMB_structure.pdf.

EFTCAMB implements the evolution of scalar and tensor perturbation equations including all the second order EFT operators. The implementation takes into account a consistent inclusion of more than one second order operator at a time and allows the use of a wide range of equation of state of dark energy for the background evolution. A number of options are made available to the user and can be found on the website and the flowchart above.

EFTCAMB has been used in a number of recent cosmological analyses including Bellini et al. (2018), Liu et al. (2017), Raveri et al. (2017), Peirone et al. (2017c), Hu et al. (2014a, b, 2015, 2016), Frusciante et al. (2016), Ade et al. (2016b) and Raveri et al. (2014).

### Recent comparison of Einstein–Boltzmann solver codes for testing general relativity

A recent careful comparative study of codes that solve Einstein–Boltzmann equations can be found in Bellini et al. (2018). Motivated by the high precision requirements from upcoming surveys such as LSST, WFIRST, Euclid, SKA, and Stage IV CMB experiments, the study aimed at finding at what level of accuracy such codes would agree with each other in calculating various CMB and matter power spectra.

The study compared codes of the second type as discussed in Sect. 11. They compared EFTCAMB, hi_class and COOP for general scalar–tensor theories. They found that CMB and matter power spectra from EFTCAMB and hi_class agree with one with another to a sub-percent level. They also found that COOP has the required accuracy and agrees with the two other at large scales but needs calibration to remain in agreement at scales below Mpc. Then they compared these three codes to the following six codes and found them in good agreement: DASh (Kaplinghat et al. 2002), BD-CAMB (Avilez and Skordis 2014) and CLASSig (Umiltà et al. 2015) that model Jordan–Brans–Dicke (JBD) gravity; GalCAMB (Barreira et al. 2012) for Galileon models; CLASS_EOS_fR (Battye et al. 2016, 2017a; Battye and Pearson 2013) for f(R) models; and CLASS-LVDM for Hořava–Lifshitz gravity (Ivanov 2017). Finally, they also compared the two codes NLCAMB (Barreira et al. 2014c) and NLCLASS (Dirian et al. 2016b) for non-local gravity and found them in good agreement.

While the comparison was done for some specific points in the cosmological parameter space, the authors stated that they expect that their comparison should hold for other models and parameters in view of the stability found for these codes. However, the authors clarify that future code comparisons should include more models, the nonlinear regime and the effect of screening mechanisms.

The authors conclude their analysis with a set of steps and warnings that a user should take into account when using these codes with various MG models to avoid any common possible sources of errors due to code versions, untested models, parameter conversion, initial conditions, and model-dependent precision requirements. We refer the interested reader to the full paper (Bellini et al. 2018) for detailed discussions and comparisons.

## Systematic effects in cosmological probes and degeneracies with modifications to GR

As we review in Sect. 13, constraining decisively modifications to GR will depend on how well ongoing and future surveys and experiments can control and mitigate systematic effects in the data. First, uncertainties on MG parameters will become soon systematic-error dominated rather than statistical-error dominated. So the precision needed to distinguish between MG and GR will depend on how well systematic uncertainties will be mitigated down. Second, some systematic effects can mimic physical effects on observables and therefore introduce a bias (shift) in the corresponding cosmological parameters including MG parameters, causing them to deviate from their GR values. We describe below some of these systematic effects taking weak gravitational lensing and intrinsic alignments as an illustrative example and refer the reader to corresponding reviews and papers for other probes and effects.

Weak gravitational lensing is a promising probe for measuring MG parameters to a one-percent precision level as forecast studies show in the next section. However, in order to reach this potential, one needs to get rid of some systematic effects such as galaxy intrinsic alignments, baryonic effects, and photometric redshift uncertainties, see for example the reviews Hoekstra and Jain (2008), Troxel and Ishak (2015), Kirk (2015), Eifler et al. (2015) and Mandelbaum (2017).

For example, Intrinsic alignments (IA) of galaxies have been recognized as one of the most serious contaminants to weak gravitational lensing and the cosmological constraints obtained from it. For example, Bridle and King (2007) found a 50% bias due to IA on determining the dark energy equation of state from weak lensing. There are two types of IA correlations. The first is the intrinsic ellipticity correlation, also known as the II signal, and is due to the fact that two physically close galaxies could be aligned by the tidal force field of the same dark matter structure surrounding them. The second type of alignment has been pointed out more recently by Hirata and Seljak (2004) and is due to the fact that if a matter structure causes the alignment of a nearby galaxy and also contributes to the lensing signal of a background galaxy, then it produces an (anti-)correlation between gravitational lensing and intrinsic ellipticities, also known as the GI signal. The GI 2-point signal has been measured in SDSS, MegaZ-LRG and other samples by various groups including Mandelbaum et al. (2006), Hirata et al. (2007), Okumura et al. (2009) and Faltenbacher et al. (2009). The 3-point IA correlations follow the same mechanisms and are known as III, GGI, and GII correlations. While the II and III correlations of IA can be, in principle, greatly reduced with photo-z’s by using cross-spectra of galaxies in two different redshift bins, so that the galaxies are separated by large enough distances to assure that the tidal effect is weak, this does not work for the GI, GGI, and GII types which happen between galaxies at different redshifts and large separations. Proposed mitigation methods for IA include parametrization–marginalization Heymans et al. (2013) and Krause et al. (2016), nulling techniques (Joachimi and Schneider 2008, 2009), or self-calibration methods (Zhang 2010a, b; Troxel and Ishak 2012a, b, c).

Laszlo et al. (2012) conducted a forecast analysis to study the disentanglement of cosmic tests of gravity from weak lensing systematics. They considered ongoing and upcoming photometric stage III surveys such as DES and stage IV such as Euclid, LSST and WFIRST. They found that using galaxy bias and intrinsic alignment models that depend on the redshift give figures of merit in constraining modifications to gravity that are a factor of four weaker than when no redshift dependence is assumed. This reflects the fact that not accounting for systematics or not properly modeling them can lead to overestimating the constraints on MG. They also found that adding Planck CMB data helps in adding a number of parameters to model systematic effect in lensing without loss of constraining power.

**Fig. 17:**
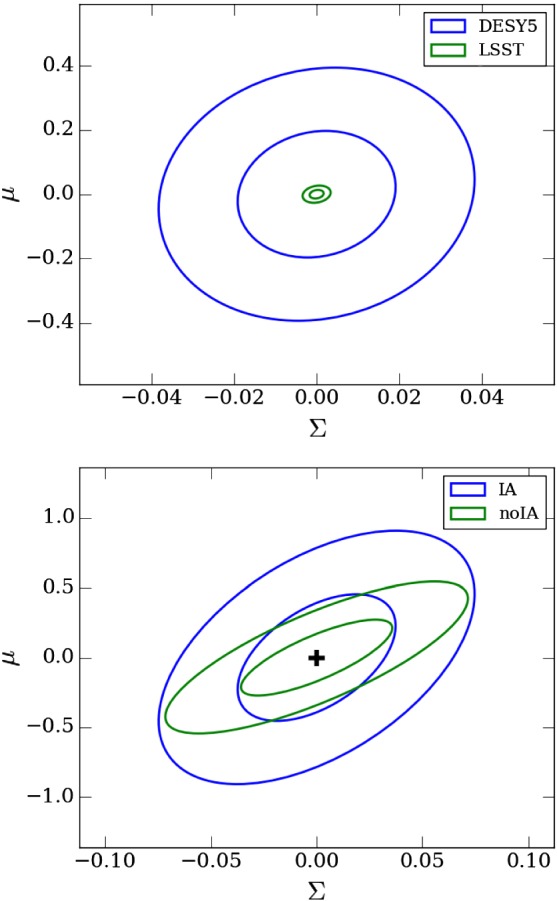
Left: Forecasted 68% and 95% confidence contours on MG parameters $\varSigma$ and $\mu$ for a future DES-Y5 survey in blue and for an LSST-like survey in green. No intrinsic alignment (IA) systematics are assumed here. While DES-Y5 promises to make tight constrains on $\varSigma$ as expected from a weak lensing survey, LSST will provide an order magnitude further improvement on the two parameters. Right: A comparison between uncertainties for DES-Y5 survey when ignoring IA in green and taking them into account in blue. This means that ignoring IA leads to underestimating the uncertainties on the parameters. This also shows that these uncertainties will be systematic-error dominated. Figure reproduced with permission from Ferté et al. (2017)

Ferté et al. (2017) constrained MG parameters $\mu$ and $\varSigma$ using weak lensing data from CFHTLenS and DES-SV, RSD data from BOSS DR 12 and the 6dF galaxy survey, and CMB data from Planck (see Sect. 6). They included three lensing systematics in their analysis. First, the shape measurement error that they model with a multiplicative factor. Second, the calibration bias of the photometric redshift distribution that they model with another parameter. Third, the intrinsic alignments that they use with a one amplitude parameter for the IA nonlinear model of Hirata and Seljak (2004) and Bridle and King (2007). They marginalized in their analysis over these three systematic parameters and compared the effect of ignoring one systematic at a time. They found that ignoring the effect of calibration bias or photometric redshift bias does affect significantly the constraints on MG parameters. However, ignoring intrinsic alignments shifts the constraints toward lower values of $\varSigma$. They found thus a degeneracy between the amplitude of IA and the $\varSigma$ MG parameter leading to higher values of $\varSigma$ when IA is included. Similar shifts in the dark energy equation of state parameters as caused by including or not including IA systematics have been studied in Krause et al. (2016) and Yao et al. (2017). Furthermore, Ferté et al. (2017) also found when forecasting constraints on MG, using 5 years data of DES, that including IA increases the uncertainties on MG parameters as shown in their figure 11 (right panel of Fig. 17 here). This shows that ignoring IA leads to overestimating MG parameter constraints from lensing.

Dossett et al. (2015) performed a constraint analysis on MG parameters using binned, functional and hybrid parameterizations including intrinsic alignment systematic effect. They used data from Planck temperature anisotropies, the galaxy power spectrum from WiggleZ survey, weak lensing tomography shear-shear cross correlations from the CFHTLenS survey, Integrated Sachs Wolfe-galaxy cross correlations, and baryon acoustic oscillation data. They found that the constraints on the amplitude of intrinsic alignment depend on the MG parametrization used but the correlation parameters between MG parameters and IA amplitude are weak to moderate.

The lesson to take from this illustrative example is that systematic effects in cosmological probes of gravity can be degenerate with MG parameters and also limit the precision that one can reach in constraining these parameters. This is the case also for other systematics such as baryonic feedback effects that can enhance growth of structure and be degenerate with some modifications to gravity as reflected on the matter power spectrum at smaller scales (Puchwein et al. 2013). The scale dependence of the $\beta$ parameter in redshift space distortion measurements, if ignored, can also introduce bias on determining the growth factor of structure leading to incorrect constraints on MG theories (Okumura and Jing 2011). We refer the reader to the following review articles including systematic effects in cosmological probes and their effect on dark energy or modified gravity models: e.g., Weinberg et al. (2013), Mandelbaum (2017), Nishizawa (2014) and references therein.

## Future cosmological constraints on GR and MG parameter forecasts

There are a number of promising cosmological surveys of large scale structure, CMB and distance probe experiments that are being built or planned such as (AdvACT, eBOSS, DESI, Euclid, HSC/PFS, LSST, POLARBEAR, SPT-3G, WFIRST and others). These will provide an overwhelming large amount of data with high precision. As we discussed above, huge efforts are also being made to develop and advance the mitigation of systematic effects to allow these surveys to reach their full constraining potential.

We will here provide a brief overview of some parameter forecast analyses that examined how well we will be able to constrain MG parameters using these future surveys. The commonly used formalism for such forecasts is the Fisher formalism (Fisher 1935) or the Markov Chain Monte-Carlo (MCMC) simulated spectra and likelihoods (Metropolis et al. 1953).

The fisher matrix $\mathbf {F}$ can be determined from the theoretical observable functions and specifications of a survey, e.g., Vogeley and Szalay (1996) and Tegmark et al. (1997). It can provide a forecasted covariance matrix $\mathbf {C}$ since $\mathbf {C}=\mathbf {F^{-1}}$. This allows one to forecast uncertainties on individual cosmological parameters $\sigma (p^i)=\sqrt{C_{ii}}$. This also allows one to calculate the correlations between parameters as $P_{ij}=C_{ij}/\sqrt{C_{ii}C_{jj}}$. Although, the Fisher formalism has shortcomings, as for example, it does not cover non-Gaussian constraints, it has been used extensively in the literature for cosmological parameter forecasts. Another quantity that is worth mentioning here is the Figure of Merit (FoM) that can be used to determine the constraining power of probes or combinations of probes, e.g., Albrecht et al. (2006, 2009). FoM is often defined to be proportional to the reciprocal of the square root of the determinant of the covariance matrix, i.e., $\sqrt{\mathrm{det}(\mathbf {C})}$ since the latter is proportional to the super-volume of the super-ellipsoides in the parameter hyperspace. Various constants of proportionality have been used including unity. As the constraints get tighter, the ellipsoid volumes get smaller and the FoM get stronger: $\mathrm {FoM}= (\det \, C)^{-1/2}$ or $\mathrm {FoM} = -\frac{1}{2} \ln (\det (\mathbf {C}))$. FoM has been used for the dark energy equation of state constraints in, e.g., Albrecht et al. (2006, 2009), Acquaviva and Gawiser (2010), Mortonson et al. (2010), Wang et al. (2010b) and for MG parameter constraints in, e.g., Dossett et al. (2011a), Laszlo et al. (2012) and Casas et al. (2017).

A second approach to parameter forecasting is to use simulated likelihoods using Markov-Chain-Monte-Carlo methods. This allows one to go beyond the Gaussian assumptions in the Fisher formalism. While the Fisher analyses can in principle provide accurate estimates in the vicinity of the best fit points in parameter space, it becomes less accurate away from such regions and in particular in higher dimensional spaces where systematic effect parameters are added to the analysis. MCMC simulation methods can be computationally intensive and have been used for dark energy equation of state forecasts with or without systematic effects, see for example Upadhye et al. (2005) and Krause et al. (2016).

Ferté et al. (2017) added to their paper a parameter forecast analysis including the two MG parameters $[1 + \mu (a)]$ and $[1 + \varSigma (a)]$ in the Poisson and weak lensing equations taking 0 values in GR. They used the full five-year DES survey and an LSST-like survey. They marginalized over five other cosmological parameters as defined previously $\{A_S, n_s, \varOmega _m, \varOmega _b, h_0\}$ and assumed a $\varLambda$CDM fiducial model. They used a Fisher analysis and used specifications for DES-5Y and LSST-like in the respective order: 5,000 and 18,000 square degrees of sky coverage; 5 and 10 redshift bins; 10 and 55 galaxies per arc-minutes squared; 0.25 and 0.20 for the intrinsic ellipticity standard deviation; $0.05(1+z)$ and $0.05(1+z)$ for the standard deviation of the photo-z estimation as a function of the redshift *z*. They first derive results for DES-Y5 and LSST-like without any use of intrinsic alignment systematics. We display their Fig. 10 (left panel of Fig. 17 here) showing forecasted 68% and 95% confidence contours on ($\varSigma$,$\mu$) around their GR values for both surveys. They give the projected uncertainties as

337 $$\begin{aligned} \sigma _{\varSigma } = 0.019, \;\;\;\;\;\;\; \sigma _{\mu } = 0.20, \end{aligned}$$

for DES-Y5, and

338 $$\begin{aligned} \sigma _{\varSigma } = 0.0017, \;\;\;\;\;\;\; \sigma _{\mu } = 0.013, \end{aligned}$$

for an LSST-like survey.

An ideal case without IA systematics, the uncertainties on $\varSigma$ from DES seem already significant while more uncertainty will persist on $\mu$. However, LSST would provide a further order of magnitude improvement on the errors and constraining even $\mu$ down to a decisive bound. However, as the authors show, including IA systematics changes significantly these forecasts. They show the effect only for constraints from DES-Y5 using an IA nonlinear model that they represent by adding an IA amplitude parameter in each of the 5 bins, so bringing the number of parameters to 12. Their Fig. 11 (right panel of Fig. 17 here) shows a comparison between constraints with and without IA systematics. It indicates that ignoring IA leads to under-estimating uncertainties. It could also be pointed out that ignoring IA can also falsely shift the best fit MG parameters in a real-data analysis. Here, the analysis did not even include complementary probes such as RSD, CMB and BAO and yet the statistical errors are of the order of a percent or sub-percent level. Clearly, the future of constraining MG models to such a level will be systematic error dominated. The analysis showed the promise of future weak lensing surveys in providing decisive answers on any modification of GR at cosmological scales.

Casas et al. (2017) performed an extended forecast analysis of MG parameters for the weak lensing and galaxy clustering surveys of Euclid, Square Kilometer Array 1 (SKA1), SKA2, and the dark energy spectroscopic instrument (DESI). They also combined the above surveys to a prior covariance matrix from Planck CMB constraints. They used the MG parameters $\mu (a)$, $\eta (a)$ and $\varSigma (a)$ as defined in Eqs. (90), (91) and (92) of Sect. 5.2.

First, they employed a binning method where they divided the redshift range $0 \le z \le 3$ into six bins with some smoothed transitions. They did not consider any scale dependence and assumed that the two parameters take their GR values of unity at redshift above 3. They assumed that the background has a flat $\varLambda \mathrm {CDM}$ evolution and considered 15 parameters $\theta =\{\varOmega _{m},\varOmega _{b},h,\ln 10^{10} A_{s},n_{s},\{\mu _{i}\},\{\eta _{i}\}\}$, with $i=1..5$. All $\eta _i$ and $\mu _i$ are equal to 1 for GR.

Second, they considered a functional time parametrization for the 2 MG parameters following Ade et al. (2016b), with no scale dependence again. They considered two sub-cases:

- case-1 where the time evolution of MG parameters is parameterized via the effective dark energy density parameter. They call this the late-time parameterization since it reduces to GR at early times
  339 $$\begin{aligned}&\mu (a,k)\equiv 1+E_{\mathrm{11}}\varOmega _{\mathrm{DE}}(a), \end{aligned}$$
  340 $$\begin{aligned}&\eta (a,k)\equiv 1+E_{\mathrm{22}}\varOmega _{\mathrm{DE}}(a); \end{aligned}$$
- case-2 where the time dependence is parameterized directly using a Taylor series in the scale factor. They call this the early-time parameterization since it allows for modifications to GR even at early times:
  341 $$\begin{aligned}&\mu (a,k)\equiv 1+E_{\mathrm{11}}+E_{\mathrm{12}}(1-a), \end{aligned}$$
  342 $$\begin{aligned}&\eta (a,k)\equiv 1+E_{\mathrm{21}}+E_{\mathrm{22}}(1-a). \end{aligned}$$
  See Casas et al. (2017) and Ade et al. (2016b) for more discussion about these parametrizations.

In addition to the five standard cosmological parameters, they added $E_{11}$ and $E_{22}$ for the late-time parametrization and $E_{11},E_{12},E_{21},E_{22}$ for the early-time case. The $E_{ij}$s are then used to reconstruct $\mu (a)$, $\eta (a)$ and $\varSigma (a)$. Here all $E_{ij}$s are zero in GR.

The fiducial model values used for the binned and the two functional parametrizations were taken as the best fit values to the data *Planck*+BAO+SNe+H$_{0}$ (BSH) as used in Ade et al. (2016b) (unlike the analysis discussed right above where the fiducial values were taken as the GR ones).

The results of Casas et al. (2017) are summarized in three Tables 7, 8, and 9 that we reproduce from Casas et al. (2017). They give results for weak lensing, galaxy clustering for Euclid, SKA1 and SKA2 separating constraints when using linear and nonlinear scales as well as with and without the combination to Planck CMB constraint covariance matrix. They used two semi-analytical approximations to include nonlinear regimes for lensing and clustering. They find that using nonlinear scales reduces and even breaks degeneracies between MG parameters in different bins and also with the overall amplitude of the matter power spectrum. They also show in the tables how much gain is obtained when adding constraints from nonlinear scales. In sum, they find that future surveys Euclid, SKA1, SKA2 and DESI (in combination with Planck priors) can constrain the present values (i.e., at $z=0$) of the parameters $\eta$, $\mu$, and $\varSigma$ to 2–3% when only linear scales are used but these reduce to about 1% or less when nonlinear scales are included. Clearly again, the determining factor for these future surveys in obtaining decisive answers on deviation from GR will be the successful mitigation of systematic effects.

**Table 7:** Forecasted uncertainties for the MG binned parameterization from Casas et al. (2017)

Euclid (Redbook)	$\mu _{1}$	$\mu _{2}$	$\mu _{3}$	$\mu _{4}$	$\mu _{5}$	$\eta _{1}$	$\eta _{2}$	$\eta _{3}$	$\eta _{4}$	$\eta _{5}$	Rel. MG
Fiducial	1.108	1.027	0.973	0.952	0.962	1.135	1.160	1.219	1.226	1.164	FoM
GC (lin)	119%	159%	183%	450%	1470%	509%	570%	586%	728%	3390%	0
GC (nl-HS)	7.0%	6.7%	10.9%	27.4%	41.1%	20%	24.3%	19.9%	38.2%	930%	19
WL (lin)	165%	2210%	4150%	13100%	22500%	2840%	3140%	8020%	29300%	39000%	$-$ 27
WL (nl-HS)	188%	255%	419%	222%	206%	330%	488%	775%	8300%	9380%	$-$ 10
GC $+$ WL (lin)	5.8%	10%	19.2%	282%	469%	7.9%	9.6%	16.1%	276%	2520%	12
GC $+$ WL $+$ *Planck* (lin)	3.4%	4.8%	7.8%	9.3%	13.1%	6.2%	7.7%	9.1%	12.7%	23.6%	27
GC $+$ WL (nl-HS)	2.2%	3.3%	8.2%	24.8%	34.1%	3.6%	5.1%	8.1%	25.4%	812%	24
GC $+$ WL $+$ *Planck* (nl-HS)	1.8%	2.5%	5.8%	7.8%	10.3%	3.2%	4.1%	5.9%	9.6%	19.5%	33
GC $+$ WL $+$ *Planck* (nl-Halofit)	2.0%	2.4%	5.1%	7.4%	10.2%	3.5%	4.1%	5.8%	9.2%	18.9%	33

$1-\sigma$ marginalized errors expressed as percentage of the corresponding fiducial value on parameters for Euclid Galaxy Clustering (GC) and Weak Lensing (WL) surveys used separately and combined. Results with the addition of the Planck-2015 prior covariance are also presented. Results are also presented using linear spectra (lin) and nonlinear prescription (nl-HS). For the GC survey, they set the cutoff to $k_{\max }=0.15$ h/Mpc in the linear case and $k_{\max }=0.5$ h/Mpc in the nonlinear case. For WL, they set the maximum cutoff in the linear case at $\ell _{\max } = 1000$, while setting it in the nl-HS case at $\ell _{\max } = 5000$. The last row is for combined GC $+$ WL $+$ *Planck* using the Halofit nonlinear fitting formula for power spectra. The last column shows the increase of Figure of Merit (FoM) (see top of Sect. 13) with respect to the reference GC linear case. Just as the FoM, a larger relative increase indicates more constraining power for the probe. One can see that each time nonlinearities are added, considerable improvements are obtained. As the authors pointed out, showing errors on $\mu$ and $\eta$ make WL look unfairly poor at constraining parameters. But when these errors are converted into errors on $\varSigma$, the constraints on $\varSigma _{1,2,3}$ are slightly better, of the order of 40% for WL(nl-HS). This is due to the fact that WL is more sensitive to the parameter $\varSigma$. The FoM itself is almost unaffected by the choice of the parameter pair because the area of a 2D ellipse is invariant under rotation. Table reproduced with permission from Casas et al. (2017), copyright by Elsevier

**Table 8:** Forecasted uncertainties for the MG late-time functional parameterization from Casas et al. (2017)

	$\varOmega _{c}$	$\varOmega _{b}$	$n_{s}$	$\ell \mathcal {A}_{s}$	*h*	$\mu$	$\eta$	$\varSigma$	MG FoM
Fiducial	0.254	0.048	0.969	3.060	0.682	1.042	1.719	1.416	Relative
GC(nl-HS)									
Euclid	0.9%	2.5%	1.3%	0.8%	1.7%	1.7%	475%	291%	2.9
SKA1-SUR	5%	15.3%	8.7%	3.8%	10.8%	18.1%	165%	108%	1.7
SKA2	0.5%	1.3%	0.4%	0.4%	0.8%	0.7%	86.8%	53.2%	5.5
DESI-ELG	1.6%	4.1%	2.3%	1.3%	2.9%	3.3%	899%	552%	1.8
WL(nl-HS)									
Euclid	6.3%	20.7%	4.6%	5.8%	13.8%	23.3%	40.9%	4.6%	4.5
SKA1	30.8%	109%	35%	36.5%	77.6%	220%	405%	36.8%	0.5
SKA2	6%	22.5%	5.9%	6.8%	15.9%	19%	33.2%	3.7%	4.9
GC $+$ WL(lin)									
Euclid	1.8%	5.9%	2.8%	2.3%	4.2%	7.1%	10.6%	2%	6.6
SKA1	10.1%	47.6%	25.4%	21.7%	40.4%	26.4%	28.8%	13.6%	3.7
SKA2	1.2%	4.5%	2.2%	1.9%	3.3%	4.1%	5.5%	1.6%	7.5
GC $+$ WL(lin) $+$ *Planck*									
Euclid	1.0%	0.7%	0.4%	0.4%	0.4%	6.2%	9.8%	1.5%	6.9
SKA1	2.4%	1.2%	0.4%	1.2%	0.7%	12%	19.8%	3.8%	5.3
SKA2	0.7%	0.6%	0.3%	0.4%	0.3%	3.6%	5.2%	1.2%	7.8
GC $+$ WL(nl-HS)									
Euclid	0.8%	2.2%	0.8%	0.7%	1.5%	1.6%	2.4%	1.0%	8.7
SKA1	4.7%	14.3%	6.2%	3.6%	9.6%	12.8%	11%	7.3%	5.5
SKA2	0.4%	1.3%	0.3%	0.4%	0.8%	0.7%	0.9%	0.6%	10.3
GC $+$ WL(nl-HS) $+$ *Planck*									
Euclid	0.7%	0.6%	0.2%	0.2%	0.3%	1.6%	2.4%	0.9%	8.9
SKA1	2.0%	1.0%	0.4%	0.8%	0.6%	3.5%	6%	2.7%	6.9
SKA2	0.4%	0.5%	0.2%	0.1%	0.2%	0.6%	0.9%	0.5%	10.3

1$\sigma$ marginalized errors expressed as percentage of the corresponding fiducial value on parameters for Galaxy Clustering (GC) and Weak Lensing (WL) surveys used separately and combined for Euclid, SKA1 and SKA2. Results with and without Planck-2015 prior covariance. Results are also presented using linear spectra (lin) and nonlinear prescription (nl-HS). Last column shows the FoM for each probe for MG parameters relative to the Euclid GC linear base case (not shown here). One can see that in general, SKA2 is the most powerful survey, followed by Euclid and SKA1. The authors note that in the case of GC alone, DESI-ELG is more constraining than SKA1-SUR. As expected, the GC survey would only constrain $\mu$ with a high accuracy, while a WL survey would constrain $\varSigma$ with a very good accuracy. The combination of both breaks the degeneracy and provides much more powerful constraints than each probe alone. However, as the authors noted, adding *Planck*  priors in the last row improves considerably the constraints on the base $\varLambda \mathrm {CDM}$ parameters but has almost no effect on the MG parameters. This is also indicated by the almost constant MG FoM. Table reproduced with permission from Casas et al. (2017), copyright by Elsevier

**Table 9:** Forecasted uncertainties for the MG early-time functional parameterization from Casas et al. (2017)

	$\varOmega _{c}$	$\varOmega _{b}$	$n_{s}$	$\ell \mathcal {A}_{s}$	*h*	$\mu$	$\eta$	$\varSigma$	MG FoM
Fiducial	0.256	0.0485	0.969	3.091	0.682	0.902	1.939	1.326	Relative
GC(nl-HS)									
Euclid	1.1%	2.3%	1.3%	0.7%	1.6%	1.8%	7.9%	4.8%	6.6
SKA1-SUR	7.9%	14.2%	13.4%	4.2%	11%	12.6%	82.7%	52.6%	2.2
SKA2	0.6%	1.3%	0.7%	0.4%	0.9%	0.9%	3.4%	1.8%	8.3
DESI-ELG	2.0%	4.3%	2.7%	1.4%	3.0%	8.2%	32%	28.6%	4.3
WL(nl-HS)									
Euclid	6.5%	21.9%	6.6%	5.9%	15.8%	2.8%	8.0%	3.4%	6.6
SKA1	32%	106%	37.2%	33%	79.3%	13.1%	37.1%	16.4%	3.4
SKA2	5.9%	22.1%	6.7%	6.1%	16.1%	2.4%	7.0%	2.9%	6.9
GC $+$ WL(lin)									
Euclid	1.8%	6.6%	3.4%	5.6%	5.2%	3.0%	6.8%	3.4%	6.4
SKA1	10.3%	46.4%	24.2%	33.6%	40.2%	14.4%	29.6%	15.5%	3.3
SKA2	1.3%	4.9%	2.5%	4.2%	3.9%	2.5%	5.7%	2.7%	6.8
GC $+$ WL(lin) $+$ *Planck*									
Euclid	1.2%	0.9%	0.6%	2.3%	0.4%	2.4%	6.5%	2.8%	6.8
SKA1	2.5%	1.5%	0.8%	2.9%	0.8%	8.8%	22.2%	8.5%	4.5
SKA2	0.9%	0.7%	0.6%	2.1%	0.3%	2.1%	5.4%	2.3%	7.2
GC $+$ WL(nl-HS)									
Euclid	1.0%	2.2%	1.2%	0.7%	1.6%	1.3%	4.4%	1.9%	8.1
SKA1	7.1%	13.4%	10.7%	4%	10%	8.2%	24.4%	10.5%	4.4
SKA2	0.6%	1.3%	0.7%	0.4%	0.9%	0.8%	2.7%	1.3%	8.8
GC $+$ WL(nl-HS) $+$ *Planck*									
Euclid	0.8%	0.7%	0.3%	0.3%	0.3%	1.3%	4.4%	1.9%	8.1
SKA1	2.1%	1.3%	0.5%	0.9%	0.7%	7%	20.8%	8.2%	4.9
SKA2	0.5%	0.5%	0.3%	0.2%	0.2%	0.8%	2.7%	1.3%	8.8

1$\sigma$ marginalized errors expressed as percentage of the corresponding fiducial value on parameters for Galaxy Clustering (GC) and Weak Lensing (WL) surveys used separately and combined for Euclid, SKA1 and SKA2. Results with the addition of the Planck-2015 prior covariance are also presented. Results are also presented using linear spectra (lin) and nonlinear prescription (nl-HS). Last column shows the FoM for each probe for MG parameters relative to the Euclid GC linear base case (not shown here). Also, adding Planck to the last combination does not provide any additional improvements in MG parameters. The authors note that in this parameterization, a GC survey alone is able to constrain both $\mu$ and $\varSigma$ to a good level for all surveys, better than with the late time parameterization, more often used in literature. Of course, WL still does better on $\varSigma$. The combination of GC $+$ WL is however less constraining in the early time parametrization than in the late time parameterization one. The nonlinear forecast for GC $+$ WL $+$ *Planck* would yield, for Euclid and SKA2, contraints at the 1–2% accuracy level on $\mu$, $\varSigma$, while for SKA1 the contraints would be at the 8% level. Table reproduced with permission from Casas et al. (2017), copyright by Elsevier

Other relatively recent MG parameter constraint forecast studies include Alonso et al. (2017), where the authors considered scalar–tensor theories and the $\alpha _x$ parameterization. They focused on Stage IV CMB-S4 and photometric surveys such as LSST, and SKA1. They also used a Fisher analysis and the FoM metric. The analysis was not restricted to the quasi-static approximation and included relativistic effects. They showed how combinations of probes can constrain redshift and scale evolution. They found that combination of probes can constrain the MG parameters down to a few percent level as well. It is even more optimistic to note that these should only improve now that the event GW170817/GRB170817A has constrained $\alpha _T$ to practically zero. Harrison et al. (2016) determined dark energy and MG parameter constraint forecasts for weak lensing surveys from SKA1 and SKA2. They find that SKA1 can provide constraints similar to stage-II experiments such as DES while SKA2 can provide tighter constraints than stage-IV experiments such as LSST, WFIRST and Euclid. Further MG parameters or $f\sigma _8$ forecast studies can be found in Spurio Mancini et al. (2018), Beutler et al. (2012) and Majerotto et al. (2012).

## Concluding remarks and outlook

Cosmological surveys and experiments are increasing in number and sophistication. Interesting ideas with new theoretical developments in gravity theories continue to emerge. In the midst of this buildup, general relativity continues to be so far prosperous and consistent with various cosmological tests and observations. It is worth noting though that while relativity is found to be consistent with all current data sets, the constraints are still too large to exclude some other possible theories.

There are some small tensions that appear between different data sets when the $\varLambda \mathrm {CDM}$ model of general relativity is being used as an underlying theoretical model. While these tensions are likely due to systematic effects in various data sets, it is worth following closely how they will evolve with upcoming and future more precise data.

Constraints on modified gravity parameters are quickly tightening up due to increasing statistical power in the data. However, this shows that for upcoming and planned surveys, the uncertainty in testing general relativity at cosmological scales will be rather systematic-error dominated. Therefore, understanding and mitigating systematic effects in cosmological probes of gravity will play a major role in obtaining decisive answers from observations.

Progress is also needed in working on modified gravity numerical simulations in order to exploit nonlinear regimes where probes such as weak lensing and galaxy clustering can reach more constraining power.

Astrophysical tests at galactic and stellar levels are found to be complementary to cosmological tests of gravity and will prove to be very useful in testing screening mechanisms of modified gravity models.

There are some interesting proposed viable theories of gravity that are still consistent with some cosmological observations and have luminal speed of gravitational waves (see Table 5). It will be useful to develop frameworks to test them against full large-scale structure and CMB data.

Finally, in the next decade or so, upcoming and future surveys or experiments (e.g. AdvACT, DES, DESI, Euclid, HSC/PFS, LiteBIRD, LSST, PIXIE, SKA, SPT-3G, WFIRST and others) along with ongoing efforts in mitigating systematic effects promise to tighten the constraints on MG parameters and provide conclusive answers on gravity physics at cosmological scales.

## Footnotes

1 Expanding Eq. (75) shows how each term relates to the respective power spectra under the assumptions of linearity in the density, velocity and galaxy bias. That is  where $\theta =\nabla .\mathbf v$ is the divergence of the peculiar velocity field and where $P_{gg}(k)$, $P_{g\theta }(k)$, $P_{\theta \theta }(k)$, are the galaxy–galaxy, galaxy–$\theta$ and $\theta$–$\theta$ power spectra respectively.
